# Comparing robotic and open partial nephrectomy under the prism of surgical precision: a meta-analysis of the average blood loss rate as a novel variable

**DOI:** 10.1007/s11701-024-02060-z

**Published:** 2024-08-07

**Authors:** Sotirios Artsitas, Dimitrios Artsitas, Irene Koronaki, Konstantinos G. Toutouzas, George C. Zografos

**Affiliations:** 1 Geniko Nosokomeio Athenon Ippokrateio, Vasilisis Sofias str. 114, 11527 Athens, Greece; 2 1st Propaedeutic Department of Surgery, School of Medicine, National and Kapodistrian University of Athens (NKUA), Mikras Asias str. 75, 11527 Athens, Greece; 3 2nd Department of Orthopaedics, KAT Attica General Hospital, Nikis str. 2, Kifissia, 14561 Athens, Greece; 4 National Technical University of Athens (NTUA), Zografou Campus, Heroon Polytechniou str. 9, 15780 Athens, Greece; 5 Laboratory of Applied Thermodynamics, School of Mechanical Engineering, National Technical University of Athens (NTUA), Heroon Polytechniou str. 9, 15780 Athens, Greece; 6 National and Kapodistrian University of Athens (NKUA), Mikras Asias str. 75, 11527 Athens, Greece

**Keywords:** Partial nephrectomy, Nephron-sparing surgery, Robotic surgery, Robot-assisted surgery, Surgical precision

## Abstract

Exploration of surgical precision in robotic procedures is extensive, yet lacks a unified framework for comparability. This study examines tissue handling precision by comparing the per-minute blood loss rate between robotic and open partial nephrectomy. A literature search from August 2022 to June 2024 identified 43 relevant studies providing data on estimated blood loss and procedure duration. The expected values and standard errors of these variables were used to compute the per-minute blood loss rate (*Q*). Meta-analytical methods estimated pooled and subgroup-level mean differences, favoring robotic surgery (MD_Q_ = − 1.043 ml/min, CI_95%_ = [− 1.338; − 0.747]). Subgroup analyses by publication year, patient matching, referral center count, and ROBINS-I status consistently supported this advantage. Sensitivity analyses confirmed the above benefit in studies with increased accuracy in reported results (MD_Q_ = *– *0.957 ml/min, CI_95%_ = [*– *1.269; *– *0.646]), low risk of bias involving matched comparisons (MD_Q_ = *– *0.563 ml/min, CI_95%_ = [*– *0.716; *– *0.410]), large sample sizes and increased statistical power (MD_Q_ = *– *0.780 ml/min, CI_95%_ = [*– *1.134; *– *0.425]), and multicenter analyses with patient matching (MD_Q_ = *– *0.481 ml/min, CI_95%_ = [*– *0.698; *– *0.263]). The subsequent analysis of correlation between the original variables suggested a slight reduction in the robotic advantage when the latter were proportionally related. Multilevel meta-regression at both temporal and qualitative scales consistently indicated a comparative benefit of the robotic approach. Potentially, lower per-minute blood loss compared to open surgery suggests that robotic partial nephrectomy demonstrates enhanced precision in tissue handling.

**Supplementary Information:**

The online version contains supplementary material available at 10.1007/s11701-024-02060-z.

## Background

Partial nephrectomy (PN) represents the prevailing technique for addressing small kidney tumors, with open partial nephrectomy (OPN) continuing to be widely acknowledged as the current standard. Whereas laparoscopic partial nephrectomy (LPN) and robot-assisted partial nephrectomy (RAPN) both provide minimally invasive alternatives, the constrained acceptance of the former is primarily attributed to its challenging learning curve. Conversely, RAPN shows potential in overcoming the technical intricacies associated with LPN. Overall, the primary goal of PN involves the attainment of the “Trifecta” outcome, and optimization of the “Margin, Ischemia, and Complication” (MIC) score, concerning factors such as margin status, ischemia duration, and complication rates [1]. Assessment of surgical margin status and application of the Clavien–Dindo classification scale are pivotal for evaluating tumor removal efficacy and postoperative complications grading, respectively. Additionally, the Comprehensive Complication Index (CCI) proves invaluable for supplementary comparison of complications among patients with diverse comorbidities [2]. Preservation of renal function depends on key determinants, including tumor volume, ischemia time (IT), and the magnitude of decline in glomerular filtration rate (GFR) [3]. In light of these considerations, approaches like selective clamping and zero-ischemia PN strive to reduce ischemic damage, emphasizing the pivotal role of surgical precision in sustaining postoperative renal function. Ultimately, robotic partial nephrectomy (RPN), facilitated by advanced surgical robotic platforms, has become the preferred modality, largely attributed to its heightened surgical capabilities, enhanced field visualization, and improved ergonomics [4, 5].

This study delves into the realm of surgical precision within the context of PN, with a specific emphasis on per-minute blood loss as a critical parameter in comparing RAPN and OPN. Recognizing the well-established ergonomic benefits associated with RAPN, this research aims to fill the existing gap by providing a comprehensive theoretical analysis on tissue handling precision, a facet that has received limited direct comparison in current studies. Existing challenges include variations in determination methods, essentially hindering comparability. Standard methodologies typically involve resection margin evaluation [6] or deterministic volumetric analyses [7, 8]. In this study, a novel approach integrates the kidney's physiological characteristics into a streamlined model. The latter accounts for the specific tissue handling requirements during PN procedures, and the inherent perfusion dynamics of the kidney, acknowledging that some degree of bleeding may occur despite effective hemostatic measures during tumor excision. Our principal aim was to assemble an exhaustive dataset for the comparative analysis of the average blood loss rate between RAPN and OPN. To accomplish this objective, we applied an enhanced meta-analytical methodology to determine the overall mean difference between the above surgical approaches.

## Materials and methods

### Literature search

The literature search spanned from August 15, 2022, to June 25, 2024, and involved the relevant comparative literature pertaining to RPN/RAPN vs. OPN through targeted keyword utilization, including “robotic”, “robot-assisted”, “open”, and “partial nephrectomy”. The study protocol, identified by the ID: CRD42022353403, incorporates the search strategy defining the individual parameters for the inclusion–exclusion process. The above information is available within the respective repository, publicly accessible on the Prospero website at: https://www.crd.york.ac.uk/PROSPEROFILES/353403_STRATEGY_20220813.pdf [9]. The investigation involved an extensive research across commonly utilized databases, such as “PubMed”, “Scopus”, “ScienceDirect”, “Google Scholar”, and Cochrane’s “CENTRAL”. Additionally, monthly alerts were employed to ensure comprehensive coverage throughout the entire spectrum of available publications.

### Study selection

To manage the substantial volume of data resulting from the initial search, rigorous eligibility criteria were exclusively applied to titles. Upon completing the systematic search for each database, unique study sets were methodically imported into the Sysrev environment [10]. In subsequent steps, the criteria for inclusion were meticulously applied, centering on English language records, non-duplicated content, and relevant investigations supplying appropriately formatted data for “RPN vs. OPN" or “RAPN vs. OPN” comparisons. These select studies were employed to furnish group-specific data for estimated blood loss (EBL) in milliliters (ml) and operative time (OT) in minutes (min). The above enabled the calculation of the average rate of blood loss per minute, considered as a crucial metric in evaluating the precision of tissue handling during tumor resection in PN procedures. Non-comparative analyses or studies lacking statistically assessable data were systematically excluded. Moreover, studies that presented results exclusively for one arm of the comparison were also omitted. Three notable modifications were introduced following the initial protocol drafting. The first entailed refining the study title, the second involved expanding the scope of databases under investigation, while the third comprised the rigorous enforcement of secondary inclusion criteria. These updates were implemented to enhance content clarity and facilitate a more comprehensive exploration of the existing literature.

The transparent application of the inclusion criteria for individual studies was performed as part of a relevant project on the Sysrev online platform, accessible via the following URL: https://sysrev.com/p/119881. Within Sysrev, studies underwent an initial assessment based on several qualitative factors, including language, duplicability, eligibility, text & data availability, and study type. Specific labels were subsequently assigned, aligning with distinct patient populations or outcomes relevant to their respective fields of interest. These meticulous processes were performed by a single member of the authoring team ([SA]). Following the initial evaluation, additional criteria for study exclusion were implemented, specifically addressing the concomitant provision of comparative data for both EBL and OT. Despite meeting the aforementioned requirement, specific categories of analyses were also ruled out. These encompassed supplements or conference presentations, along with studies primarily focused on cost analyses. Moreover, studies involving special patient populations were deemed inappropriate for inclusion to preserve homogeneity. Such populations involved individuals with solitary kidneys, highly complex renal masses, or solely hilar tumors. Additionally, studies that exclusively adopted a retroperitoneal approach, applied cold ischemia, or included patient groups with chronic kidney disease (CKD) were also eliminated. The final set of studies for analysis was curated through the collaborative efforts of two investigators ([SA], [DA]). The precise process of study exclusion or inclusion will be visually delineated in an appropriately constructed PRISMA flowchart, to be presented in the forthcoming “Results” section.

### Outcomes

The key goal of this research was to evaluate through statistical estimation, the per-minute blood loss in PN, quantified as the composite outcome “Q” and expressed in ml/min. Specifically, this variable was physiologically perceived as inversely correlated with the degree of surgical precision. In addition, two secondary outcomes were pursued, encompassing the average blood loss in milliliters (ml) and the procedure duration in minutes (min), collectively referred to as “original variables”. The aforementioned outcomes are widely acknowledged in the relevant literature comparing RPN/RAPN and OPN, and their individual analysis was deemed indispensable for comprehending their relative impacts on the derived quotient. To uphold adherence to the established protocol and effectively manage the substantial volume of data, no additional outcomes beyond those specified above were sought. Nevertheless, a thorough analysis was undertaken to detect possible disparities in comparability across the integrated studies, with particular attention to significant deviations in baseline characteristics among the specific patient populations being compared. The latter are concisely outlined in an appropriate study table (Table 1) introduced within the “Results” section.

### Qualitative assessment

Following the compilation of numerical data, design features, and methodological profiles of the included studies, three reviewers ([DA], [IK], and [KT]) undertook the assessment and application of two qualitative classification scales. The initial implementation of the Newcastle—Ottawa Scale (NOS) resulted in a broad assessment, with the subsequent use of the ROBINS-I tool ensuring a more in-depth classification into specific risk of bias (ROB) clusters [11, 12]. During the rigorous tabulation of records and simultaneous acquisition of numerical data, specific metadata concerning various study characteristics were systematically captured. The aforementioned metadata encompassed essential features, including the author's name, publication year, comparison protocol, patient matching, confounding factors, study durations, single- or multicenter types of analysis, NOS evaluation, ROBINS-I clustering, and other pertinent information. To address missing data, a predetermined strategy was implemented for their exclusion from both the experimental (RPN/RAPN) and control populations (OPN). The above measure facilitated the estimation of standard errors for the average per-minute blood loss.

### Evidence acquisition

The stratification of studies within the Sysrev environment involved three hierarchical stages. The initial two levels, as detailed in the “Study selection” subsection, focused on fundamental binary parameter assessment and field of interest labeling. These stages were primarily overseen by two team members ([SA] and [DA]). At the third level, each study underwent detailed examination for data pertaining to patient group averages and standard deviations (SD) of the original variables, within the context of RPN/RAPN vs. OPN comparison. Concretely, two reviewers ([DA] and [IK]) initially collaborated and subsequently worked independently, ultimately organizing the eligible studies into a “.csv” file for systematic analysis. The above-described 3-stage process culminated in the tabulation of both the included studies and the provided or derived data in a structured and intuitive manner.

Regarding the newly established quantitative measure, denoted as "Q" and represented by the "EBL / OT" quotient, its expected value (EV_Q_) and standard error (SE_Q_) were derived assuming a normal distribution. Summary statistics, including the means ${(}\overline{{{\text{EBL}}}} {, }\overline{{{\text{OT}}}} {) }$ and standard deviations ${\text{(SD}}_{{{\text{EBL}}}} {\text{, SD}}_{{{\text{OT}}}} {) }$ of the original variables, were utilized to estimate Q for each study and arm, addressing any gaps in data within the compared populations. The expected value and standard error of Q (EV_Q_ and SE_Q_ respectively) were computed using the estimator functions (1–3), following the methodology delineated by van Kempen and van Vliet in a relevant investigation of ratio estimators [13]. The above functions were operated by assuming that every study contributed a singular sample for each comparison group (*n* = 1). Additionally, to expedite the computational process, the original variables were required to be in the “mean—SD” format, expressed in milliliters (ml) and minutes (min), respectively. For non-standard formatted data, a uniform transformation was applied using the “rule of thumb” (mean ≈ median, and SD ≈ interquartile range/1.35 or SD ≈ range/4) assuming a normal distribution [14, 15]. These approximations were practically applied to ensure homogeneity, as the data provision was inconsistent between the formats: "median—interquartile range (IQR)" and “median—range”. The methodology described above facilitated a consistent and robust treatment of data for analysis.

1 $${\text{EV}}_{{\text{Q}}} {\text{ = EV}}\left\{ {\frac{{\overline{{{\text{EBL}}}} }}{{\overline{{{\text{OT}}}} }}} \right\} \, \approx \, \frac{{\overline{{{\text{EBL}}}} }}{{\overline{{{\text{OT}}}} }}{ + }\frac{{1}}{{\text{n}}}\left[ {{\text{SD}}_{{{\text{OT}}}}^{{2}} \, \frac{{\overline{{{\text{EBL}}}} }}{{\overline{{{\text{OT}}}}^{{3}} }}{ - }\frac{{{\text{cov}}\left( {\text{EBL,OT}} \right)}}{{\overline{{{\text{OT}}}}^{{2}} }}} \right]$$

2 $${\text{SE}}_{{\text{Q}}} {\text{ = SE}}\left\{ {\frac{{\overline{{{\text{EBL}}}} }}{{\overline{{{\text{OT}}}} }}} \right\} \, \approx \, \left[ {\frac{{1}}{{\text{n}}}\left( {\frac{{{\text{SD}}_{{{\text{EBL}}}}^{{2}} }}{{\overline{{{\text{OT}}}}^{{2}} }}{ + }\frac{{\overline{{{\text{EBL}}}}^{{2}} {\text{ SD}}_{{{\text{OT}}}}^{{2}} }}{{\overline{{{\text{OT}}}}^{{4}} }}{ - }\frac{{{2 }\overline{{{\text{EBL}}}} {\text{ cov}}\left( {\text{EBL,OT}} \right)}}{{\overline{{{\text{OT}}}}^{{3}} }}} \right)} \right]^{{{\raise0.7ex\hbox{${1}$} \!\mathord{\left/ {\vphantom {{1} {2}}}\right.\kern-0pt} \!\lower0.7ex\hbox{${2}$}}}}$$

3 $${\text{cov}}\left( {\text{EBL,OT}} \right){\text{ = r SD}}_{{{\text{EBL}}}} {\text{ SD}}_{{{\text{OT}}}}$$

The estimator functions (1–2) incorporate a fourth parameter in the calculation of EV_Q_ and SE_Q_, represented by the covariance between EBL and OT (${\text{cov}}\left( {\text{EBL, OT}} \right)$), as defined within Eq. (3). Estimating this additional parameter involves determining the correlation between EBL and OT through the Pearson’s coefficient (r), which quantifies the extent to which one outcome varies in relation to the other [16, 17]. To effectively approximate the corresponding coefficient values, appropriate Monte Carlo simulations were executed for each study and comparison arm, by leveraging the expected values and standard errors of the original variables [18]. The clinical relevance of r pertains to ${\text{cov}}\left( {\text{EBL, OT}} \right)$, presuming that the original variables adhere to a joint normal distribution, while the conceivable spectrum of *r*-values theoretically spans between − 1 and + 1 [19]. To elaborate, markedly positive *r*-values signify that the original variables move in tandem, suggesting simultaneous increases or decreases. The former scenario suggests more complex interventions, where increased EBL requires an extended OT for effective hemostatic management. The latter implies more manageable cases of PN, where the procedure is completed in a brief timeframe with efficient hemostasis. On the other hand, for highly negative *r*-values the original variables move in opposite directions. The first scenario involves increased bleeding within constrained procedure duration, possibly indicating aggressive tissue handling without meticulous control of small bleeding foci. Conversely, the second reflects reduced bleeding within a prolonged operative timeframe, suggesting the need for additional OT for appropriate hemostatic measures to be applied. These extreme r-values are likely to be associated with the surgeon's skill, experience, and ability to implement intraoperative hemostasis effectively, given the common occurrence of bleeding during tumor resection in PN. Following the computation of Q, the resulting data along with those pertaining to the original variables, were collectively re-tabulated in a dedicated “.csv” file by three reviewers ([SA], [DA], and [IS]).

### Statistical analysis

Building upon the above-articulated theoretical foundation, the newly introduced “Q” variable was considered a suitable measure inversely representing the level of surgical precision during PN, specifically addressing the blood loss rate. To evaluate the overall impact in the contrast between robotic and open PN, a range of meta-analytical techniques were utilized, employing the mean difference in Q (MD_Q_) as the effect size. Due to the widespread integration of studies and the extensive provision of comparative data, a random effects model was implemented, incorporating the Hartung and Knapp (H–K) adjustment to obtain the respective MD_Q_-estimates [20, 21].

The outcomes of the meta-analysis (MA) were graphically represented through appropriate forest plots, detailing both overall and individual effects, supplemented by the corresponding weights from each included study. These plots provided a comprehensive overview of the comparative effect, accompanied by labels indicating its direction. The forthcoming quantitative results will be presented in a dedicated table (Table 2) within the relevant section of the analysis. In subsequent meta-regression visualizations, a conventional graphical representation portrays each incorporated study as a circle, of size directly proportional to the precision of the reported outcomes. More specifically, linear regression models were applied, utilizing the restricted maximum likelihood (REML) method, to visually illustrate the trend of the MD_Q_ effect [22, 23]. In the aforementioned analysis, two moderators were employed, with the first corresponding to the publication year, while the second pertaining to the NOS grading, quantitatively expressed as the quality star count.

To examine the potential presence of publication bias (PB), conventional funnel and radial plots were systematically employed, complemented by the application of Egger's test, to bolster the assessment’s robustness. Additionally, a comprehensive exploration of small-study effects (SSEs) was conducted using meticulously configured funnel plots. These plots graphically incorporate a regression curve to highlight the direction of the effect, with its significance being assessed through the magnitude of the curvature, as a deviation from the vertical, using Q-Q' plots [24, 25]. Heterogeneity was evaluated using the inter-study variation (τ^2^), and relevant statistical parameters, including Higgins I^2^ and Cochran's Q [26, 27]. Subgroup analysis (SGA) was conducted to identify further origins of heterogeneity, taking into account factors such as publication time-points, population matching, engagement of multiple referral centers, and ROBINS-I stratification. The cutoff point for publication year was pragmatically set at 2018, establishing the last 5-year period of RAPN’s utilization. Additionally, meta-regression analysis (MRA) scrutinized variations in the overall effect based on both the year of publication and NOS quality rating. To comprehensively explore heterogeneity at both the pooled data level and within the context of SGA, MRA was conducted for the entire study set and for each of the above subgroups, respectively.

Conclusively, a 5-level sensitivity analysis (SA) was conducted to validate the findings emerging from both the pooled meta-analysis and subgroup analysis. The first level entailed the curation of a subset of studies to ensure consistency in the accuracy of the derived Q-estimates. This subset was formed by applying a predefined threshold to the range of the 95% confidence interval (CI_95%_) accompanying the MD_Q_ from each study within the pooled data, ensuring sufficient homogenization of the results. Subsequently, at the second level, a subset of matched analyses was generated, concurrently categorized as “ROBINS-I: Low”. The primary objective of this sub-analysis was to extract conclusions from the most dependable sources, considering their comparative design and ROB profile. Advancing to the third level, the criterion focused on the patient populations included in each instance. Specifically, this sub-analysis incorporated studies with large sample sizes, defined as those exceeding the average total patient population within the original study set. The overarching goal was to estimate the effect within a subset that enhances statistical power, thereby effectively reducing Type 2 errors related to falsely insignificant findings. Proceeding to the fourth level, attention was redirected towards shaping a subset distinguished by enhanced reliability. This was accomplished by introducing two criteria intricately linked to the design of each study, which required the simultaneous adoption of patient matching while adhering to a multicenter type of analysis. The fifth and last stage of the SA entailed scrutinizing a sequence of consecutive r-values. This process impacted the calculation of Q, involving the stepwise computation of the covariance between the original variables. The examination covered the incremental progression of the correlation coefficient (r) from − 0.99 to + 0.99 in steps of 0.1. The analysis aimed to ascertain the trajectory of the overall effect between robotic and open PN, as the coefficient varies across the above range. The interpretation of results was conducted by taking into account the assumptions made earlier regarding the extreme positive or negative coefficient values. More specifically, the highest positive *r*-values were assumed to be associated with procedures where *Q* is primarily driven by inherent intraoperative challenges in conducting the procedure (e.g., prior abdominal surgery, reoperations, large retroperitoneal lesions, extremely complex renal tumors, extensive local recurrences, history of radiation therapy, etc.). Conversely, the most negative *r*-values were considered to be associated with the surgeon's skill level, experience, and intraoperative decision-making capacity during tissue dissection and hemostatic management.

### Reporting of results and data availability

The analysis of original and derived data was performed by utilizing the R programming language, specifically in version 4.3.2 [28]. Graphical representation for qualitative assessment utilized the statistical package "robvis" within the aforementioned context [29]. The current investigation followed the pertinent guidelines outlined on the PRISMA website (http://prisma-statement.org/Extensions/Protocols), ensuring alignment with the most recent checklist published in 2020. The presentation of results follows the format: “MD_Q_–CI_95%_”. To ensure methodological transparency, the entire dataset of outcomes, accompanied by the analytical code, is publicly accessible on GitHub [30]. The relevant repository is available at: https://github.com/sotbike/Q.git.

## Results

### Study retrieval

**Fig. 1:**
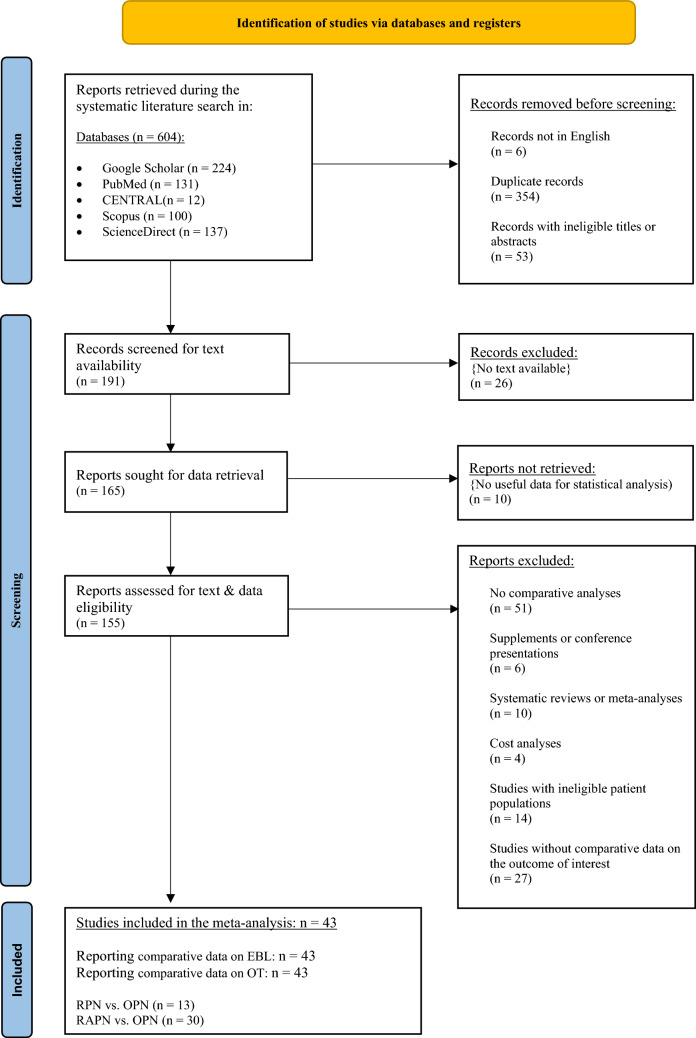
Flowchart of studies according to the Preferred Reporting Items for Systematic Reviews and Meta-Analyses (PRISMA). From: Page MJ, McKenzie JE, Bossuyt PM, Boutron I, Hoffmann TC, Mulrow CD, et al. The PRISMA 2020 statement: an updated guideline for reporting systematic reviews. BMJ 2021;3 72:n71. https://doi.org/10.1136/bmj.n71

The systematic exploration of international literature is visually outlined in Fig. 1, presenting the relevant PRISMA flowchart. Commencing with an initial identification of 604 studies, subsequent steps involved the elimination of non-English papers, duplicates, and manuscripts with non-eligible titles or abstracts, resulting in 191 records for text and data screening. From the resulting study set, 26 records were excluded due to unavailability of their full text, while an additional 10 were eliminated as they did not contain usable data for analysis. This process resulted in a comprehensive assessment of text and data eligibility for the remaining 155 studies. Upon closer examination, 51 records lacking comparative data were excluded from consideration. Additionally, six records constituting supplements or conference presentations, along with 10 comparisons related to systematic reviews or meta-analyses, and four studies centered around cost analyses were deemed ineligible and thus excluded. Fourteen records featuring special patient populations were also omitted, to preserve homogeneity and comparability across the analyses within the resulting study set. Among these, three studies exclusively involved patients with solitary kidneys, six with highly complex renal tumors, one with hilar masses, one with reoperations, and one with CKD already present at diagnosis. Furthermore, in one study, the retroperitoneal approach was exclusively applied, while in another, only cold ischemia was employed. Despite initial consideration for eligibility, an additional 27 studies were removed due to the absence of concurrent data provision concerning the original variables. Following the meticulous filtering process outlined above, a refined cohort of 43 studies was identified as suitable for in-depth analysis. In this definitive dataset, 13 records explored the comparison of RPN vs. OPN, and 30 delved into that of RAPN vs. OPN. The collective study set encompassed data from 18,179 patients, distributed across 9743 in the robotic or robot-assisted PN group and 8436 in the open surgery group. Notably, missing data affected 76 patients in total, with 60 and 16 in robotic and open surgery arms, respectively.

### Study characteristics

**Fig. 2:**
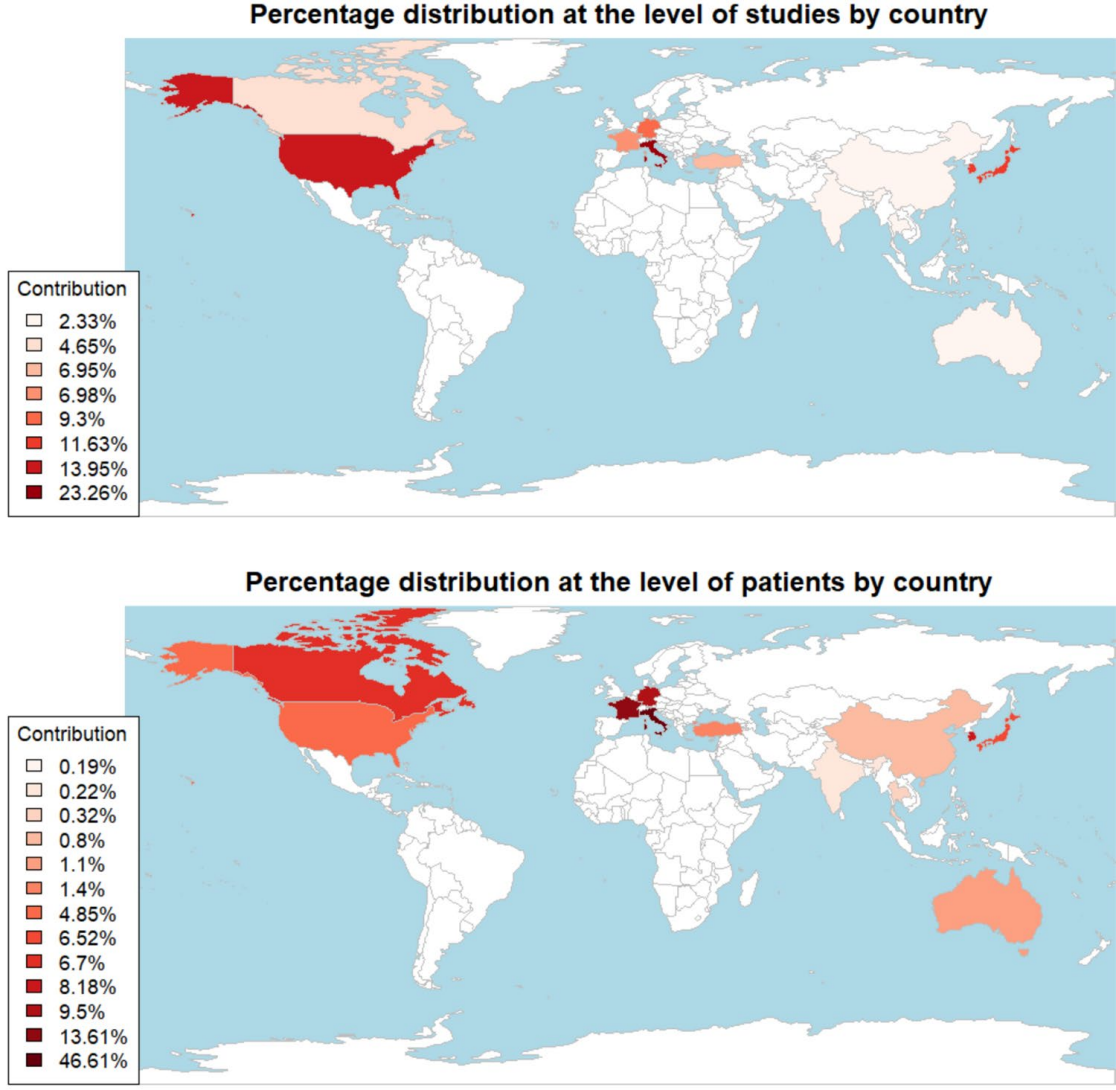
Comprehensive chart featuring an embedded map displaying the percentage distributions of studies and patients by their respective countries of origin

This subsection provides demographic insights relevant to the studies incorporated in the analysis. Primary data sources, at both the study and patient levels, originated predominantly from the USA, Korea, and Japan, with substantial contributions from Italy, Germany, and France within continental Europe, ensuring global coverage. A thorough analysis of these data was conducted to systematically capture any potential deviations in their geographic distributions. The respective percentages of data sources at the study and patient levels are visually depicted in Fig. 2, with additional detailed information provided in the corresponding pie charts within Supplementary Fig. 1 (available as supplementary material in Supplementary file 2).

**Fig. 3:**
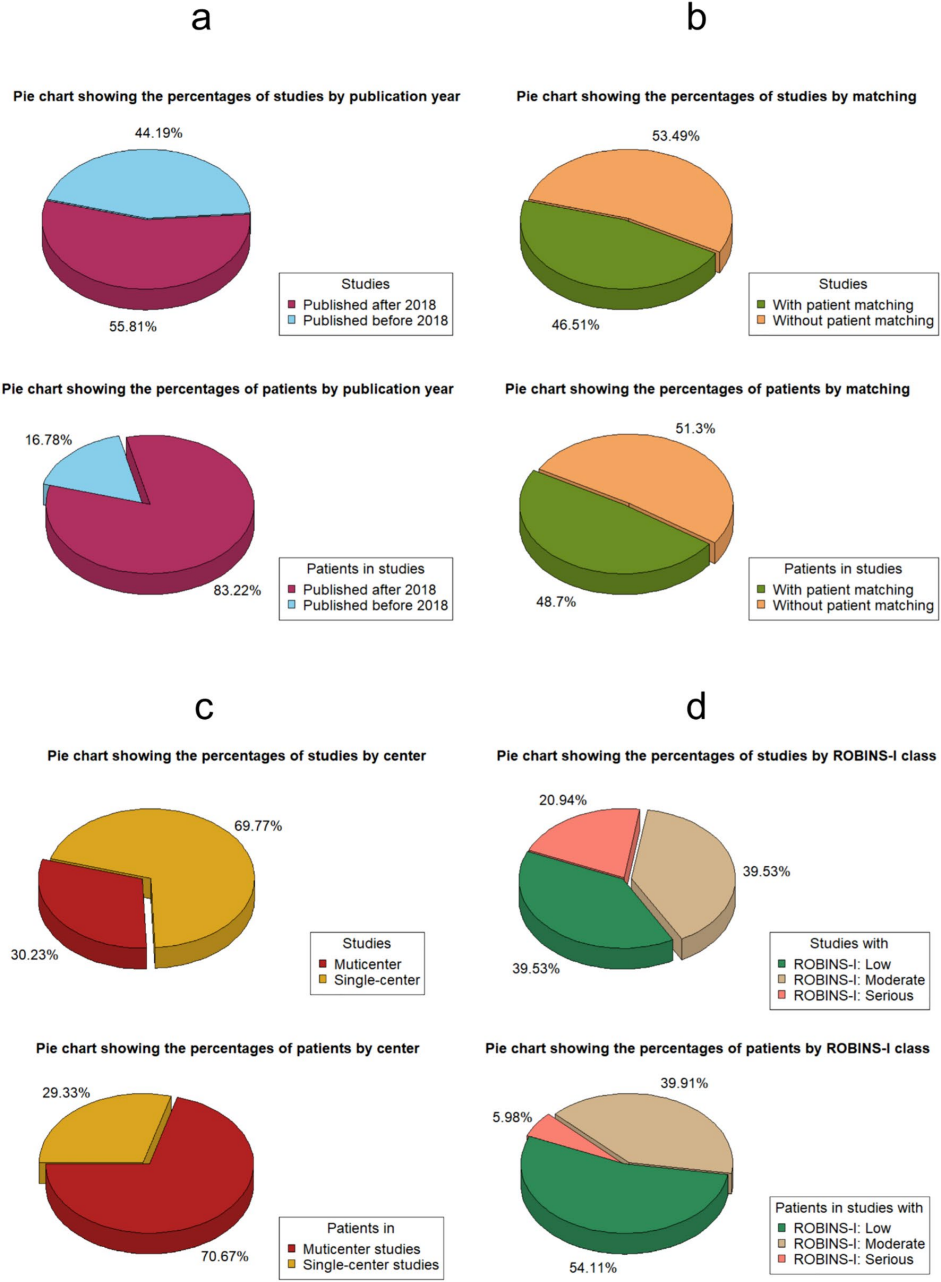
Pie charts depicting the percentage distributions of available data for studies and patients, respectively, categorized by publication year (**a**), adoption of patient matching (**b**), number of referral centers involved (**c**) and ROBINS-I class (**d**)

The temporal allocation of study activity reveals that the majority was predominantly conducted between 2010 and 2020 and is visually represented in Supplementary Fig. 2. This graph also delineates the duration of studies according to their risk of bias clustering as per the ROBINS-I tool. Supplementary Figs. 3 and 4 provide complementary details on patient matching implementation, and the adoption of single- or multicenter analysis, respectively. Overall examination of these charts reveals no significant variations in study durations across the investigated subgroups. In terms of publication year, almost 56% of the studies (representing 83% of the patient data) were published after 2018. Furthermore, patient matching was implemented in approximately 46.5% of the overall study count and nearly half of the cumulative patient population. The above distributions are illustrated in the relevant pie charts of Fig. 3a and b, respectively. Concerning referral centers, approximately 30% of the studies (encapsulating just over 70% of the patient data) were part of multicenter analyses. Finally, with respect to the ROBINS-I classification, 39.5% of the studies (encompassing almost 54% of the patient data) were designated as having a low risk of bias (ROB). On the other hand, the same percentage of studies, representing 40% of the total patient population, were classified as having an intermediate ROB, with the corresponding percentages for high-risk studies being 21% and 6%, respectively. The above results are presented in more detail within Fig. 3c and d.

Overall, Table 1 furnishes a comprehensive summary of the characteristics spanning the entire array of included studies. It presents design details such as publication time points, application of patient group matching, adoption of multi- or single-center analyses, study durations, NOS ratings, ROBINS-I strata, and variations in baseline features within the final study set. Key points of disparity included older individuals with renal tumors of increased size and complexity, predominantly within the OPN group, a fact associated with the adoption context of robotic approach in current surgical practice.

**Table 1:** Table of included studies, detailing title, author, publication year, methodological design, duration of activity, quality assessment, patient population in each arm of the comparison, and baseline differences between the compared populations

Title	Author	Study design	Population	Duration	Quality	Baseline deviations
Comparison of the Trifecta outcomes of robotic and open nephron-sparing surgeries performed in the robotic era of a single institution	Acar et al. 2015 (Turkey) [58]	Single-center without patient matching (RAPN vs. OPN)	*N*_exp_ = 59 *N*_ctrl_ = 74 (Total: 133)	1705 days (1/5/2010–31/12/2014)	NOS: 7 ROBINS-I: Moderate	OPN: older pts, higher RENAL & PADUA scores
Prediction of significant renal function decline after open, laparoscopic, and robotic partial nephrectomy: External validation of the Martini's nomogram on the RECORD2 project cohort	Antonelli et al. 2022 (Italy) [59]	Multicenter without patient matching (RAPN vs. OPN)	*N*_exp_ = 981 *N*_ctrl_ = 886 (Total: 1867)	1460 days (1/1/2013–31/12/2016)	NOS: 8 ROBINS-I: Low	OPN: retroperitoneal approach RAPN: enucleation, on-clamp technique
Clinical and oncological outcomes of open partial nephrectomy versus robot assisted partial nephrectomy over 15 years	Audige et al. 2022 (France) [60]	Multicenter without patient matching (RAPN vs. OPN)	*N*_exp_ = 201 *N*_ctrl_ = 204 (Total: 405)	4747 days (1/1/2007–31/12/2019)	NOS: 7 ROBINS-I: Moderate	OPN: older pts, higher CCI, lower RENAL score
Nephrometry score matched robotic vs. laparoscopic vs. open partial nephrectomy	Banapour et al. 2018 (USA) [61]	Multicenter with patient matching (RPN vs. OPN)	*N*_exp_ = 163 *N*_ctrl_ = 176 (Total: 339)	2921 days (1/1/2007–31/12/2014)	NOS: 6 ROBINS-I: Moderate	OPN: older pts, higher CCI (matched for RENAL score)
Comparison of surgical outcomes of open, laparoscopic, and robotic partial nephrectomy	Boylu et al. 2015 (Turkey) [49]	Single-center without patient matching (RAPN vs. OPN)	*N*_exp_ = 46 *N*_ctrl_ = 20 (Total: 66)	1825 days (1/1/2009–31/12/2013)	NOS: 7 ROBINS-I: Moderate	RAPN: higher ASA score OPN: higher RENAL score
Perioperative outcomes of open, laparoscopic, and robotic partial nephrectomy: A prospective multicenter observational study (The RECORd 2 Project)	Bravi et al. 2019 (Italy) [62]	Multicenter without patient matching (RPN vs. OPN)	*N*_exp_ = 789 *N*_ctrl_ = 682 (Total: 1471)	1460 days (1/1/2013–31/12/2016)	NOS: 9 ROBINS-I: Low	OPN: older pts, higher PADUA score, higher rate of solitary kidneys
The IRON study: Investigation of robot-assisted versus open nephron-sparing surgery	Bravi et al. 2023 (Italy) [63]	Multicenter with patient matching (RAPN vs. OPN)	*N*_exp_ = 2404 *N*_ctrl_ = 1063 (Total: 3467)	5478 days (1/1/2004–31/12/2018)	NOS: 8 ROBINS-I: Low	OPN: older pts, lower preoperative eGFR, higher rate of solitary kidneys
Functional and oncologic outcomes of open, laparoscopic and robotic partial nephrectomy: A multicenter comparative matched-pair analysis with a median 5 years follow up	Chang et al. 2018 (Korea) [64]	Multicenter with patient matching (RAPN vs. OPN)	*N*_exp_ = 122 *N*_ctrl_ = 122 (Total: 244)	2556 days (1/1/2006–31/12/2012)	NOS: 8 ROBINS-I: Low	No special population
						No differences
Morphometric profile of the localized renal tumors managed either by open or robot-assisted nephron-sparing surgery: The impact of scoring systems on the decision-making process	Esen et al. 2013 (Turkey) [65]	Single-center without patient matching (RAPN vs. OPN)	*N*_exp_ = 32 *N*_ctrl_ = 23 (Total: 55)	791 days (1/5/2010–30/6/2012)	NOS: 7 ROBINS-I: Moderate	OPN: older pts, higher RENAL & PADUA scores
A multicenter matched-pair analysis comparing robot-assisted versus open partial nephrectomy	Ficarra et al. 2014 (Italy) [66]	Multicenter with patient matching(RPN vs. OPN)	*N*_exp_ = 200 *N*_ctrl_ = 200 (Total: 400)	760 days (1/1/2009–31/1/2011)	NOS: 8 ROBINS-I: Low	No special population
						No differences
Purely off-clamp partial nephrectomy: Robotic approach better than open using a Pentafecta outcome with propensity score matching	Gandi et al. 2022 (Italy) [67]	Single-center with patient matching (RAPN vs. OPN)	*N*_exp_ = 71 *N*_ctrl_ = 71 (Total: 142)	2190 days (1/1/2014–31/12/2019)	NOS: 8 ROBINS-I: Low	Clampless technique
						No differences
Robotic partial nephrectomy for clinical T2a renal mass is associated with improved Trifecta outcome compared to open partial nephrectomy: A single surgeon comparative analysis	Ghali et al. 2019 (USA) [47]	Single-center without patient matching (RPN vs. OPN)	*N*_exp_ = 59 *N*_ctrl_ = 91 (Total: 150)	2921 days (1/7/2008–30/6/2016)	NOS: 6 ROBINS-I: Moderate	cT2a renal tumors
						No differences
Achieving the “Trifecta” with open versus minimally invasive partial nephrectomy	Ghavimi et al. 2021 (Canada) [68]	Multicenter without patient matching (RPN vs. OPN)	*N*_exp_ = 284 *N*_ctrl_ = 746 (Total: 1030)	2860 days (1/1/2011–31/10/2018)	NOS: 7 ROBINS-I: Moderate	OPN: larger tumor size, lower preoperative eGFR
Comparison of peri- and intraoperative outcomes of open vs robotic-assisted partial nephrectomy for renal cell carcinoma: A propensity-matched analysis	Hoeh et al. 2023 (Germany) [69]	Single-center with patient matching (RAPN vs. OPN)	*N*_exp_ = 134 *N*_ctrl_ = 481 (Total: 615)	6605 days (1/1/2003–31/1/2021)	NOS: 7 ROBINS-I: Moderate	No special population
						OPN: older pts, higher RENAL score, larger tumors, more pts with previous abdominal surgery
Comparison of open and robotic-assisted partial nephrectomy approaches using multicentric data (UroCCR-47 study)	Ingels et al. 2022 (France) [70]	Multicenter without patient matching (RPN vs. OPN)	*N*_exp_ = 1409 *N*_ctrl_ = 560 (Total: 1969)	1276 days (1/1/2014–30/6/2017)	NOS: 8 ROBINS-I: Moderate	No special population
						No differences
Comparison of robot-assisted and open partial nephrectomy for completely endophytic renal tumors: A single center experience	Kara et al. 2016 (USA) [71]	Single-center without patient matching (RAPN vs. OPN)	*N*_exp_ = 87 *N*_ctrl_ = 56 (Total: 143)	1856 days (1/1/2011–31/1/2016)	NOS: 6 ROBINS-I: Moderate	Endophytic tumors
						OPN: higher rate of solitary kidneys
A single surgeon's experience with open, laparoscopic, and robotic partial nephrectomy	Klaassen et al. 2014 (USA) [72]	Single-center without patient matching (RPN vs. OPN)	*N*_exp_ = 35 *N*_ctrl_ = 23 (Total: 58)	2037 days (1/8/2006–28/2/2012)	NOS: 6 ROBINS-I: Serious	OPN: higher RENAL score, lower preoperative GFR
Robotic-assisted versus conventional open partial nephrectomy (Robocop): A propensity score-matched analysis of 249 patients	Kowalewski et al. 2021 (Germany) [2]	Single-center with patient matching (RAPN vs. OPN)	*N*_exp_ = 83 *N*_ctrl_ = 166 (Total: 249)	3286 days (1/1/2010–31/12/2018)	NOS: 8 ROBINS-I: Moderate	No special population
						No differences
Randomized controlled feasibility trial of robot-assisted versus conventional open partial nephrectomy: The ROBOCOP II study	Kowalewski et al. 2023 (Germany) [73]	Single-center with patient matching (RAPN vs. OPN)	*N*_exp_ = 25 *N*_ctrl_ = 25 (Total: 50)	731 days (1/1/2020–1/1/2022)	NOS: 9 ROBINS-I: Low	No special population
						No differences
Transition from open and laparoscopic to robotic partial nephrectomy: Learning curve and outcomes	Kumar et al. 2024 (India) [74]	Single-center without patient matching (RAPN vs. OPN)	*N*_exp_ = 25 *N*_ctrl_ = 15 (Total: 40)	2464 days (1/6/2015–28/2/2022)	NOS: 6 ROBINS-I: Serious	No special population
						OPN: higher RENAL score, larger tumors
Open versus robot-assisted partial nephrectomy: Effect on clinical outcome	Lee et al. 2011 (Korea) [75]	Single-center without patient matching (RPN vs. OPN)	*N*_exp_ = 69 *N*_ctrl_ = 234 (Total: 303)	2801 days (1/5/2003–31/12/2010)	NOS: 6 ROBINS-I: Serious	No special population
						RPN: higher BMI
Open partial nephrectomy vs. robot-assisted partial nephrectomy for a renal tumor larger than 4 cm: A propensity score matching analysis	Lee et al. 2021 (Korea) [46]	Single-center with patient matching (RAPN vs. OPN)	*N*_exp_ = 67 *N*_ctrl_ = 67 (Total: 134)	5082 days (1/6/2003–30/4/2017)	NOS: 8 ROBINS-I: Low	Tumors larger than 4 cm
						No differences
A comparison of robotic, laparoscopic and open partial nephrectomy	Lucas et al. 2012 (USA) [76]	Single-center with patient matching (RPN vs. OPN)	*N*_exp_ = 27 *N*_ctrl_ = 54 (Total: 81)	2556 days (1/1/2004–31/12/2010)	NOS: 7 ROBINS-I: Moderate	No special population
						No differences
Robotic-assisted partial nephrectomy provides better operative outcomes as compared to the laparoscopic and open approaches: Results from a prospective cohort study	Luciani et al. 2017 (Italy) [77]	Single-center with patient matching (RAPN vs. OPN)	*N*_exp_ = 110 *N*_ctrl_ = 73 (Total: 183)	4198 days (1/1/2005–30/6/2016)	NOS: 6 ROBINS-I: Serious	RAPN: higher preoperative Hb
Robotic and open partial nephrectomy for localized renal tumors larger than 7 cm: A single-center experience	Malkoc et al. 2017 (USA) [78]	Single-center without patient matching (RPN vs. OPN)	*N*_exp_ = 54 *N*_ctrl_ = 56 (Total: 110)	2524 days (1/1/2009–30/11/2015)	NOS: 7 ROBINS-I: Serious	Tumors larger than 7 cm
						No differences
Robotic-assisted laparoscopic partial nephrectomy vs. laparoscopic and open partial nephrectomy: A single-site, two-surgeon, retrospective cohort study	Masoumi-Ravandi et al. 2024 (Canada) [79]	Single-center without patient matching (RAPN vs. OPN)	*N*_exp_ = 82 *N*_ctrl_ = 106 (Total: 188)	2372 days (1/2/2015–31/7/2021)	NOS: 6 ROBINS-I: Serious	No special population
						OPN: younger pts, higher preoperative Cr, larger tumors
A prospective comparison of the pathologic and surgical outcomes obtained after elective treatment of renal cell carcinoma by open or robot-assisted partial nephrectomy	Masson-Lecomte et al. 2013 (France) [5]	Single-center without patient matching (RAPN vs. OPN)	*N*_exp_ = 42 *N*_ctrl_ = 58 (Total: 100)	1095 days (1/1/2008–31/12/2010)	NOS: 7 ROBINS-I: Moderate	No special population
						OPN: higher RENAL score
Margin and complication rates in clampless partial nephrectomy: A comparison of open, laparoscopic and robotic surgeries	Mearini et al. 2016 (Italy) [1]	Single-center without patient matching (RAPN vs. OPN)	*N*_exp_ = 31 *N*_ctrl_ = 80 (Total: 111)	3651 days (1/1/2006–31/12/2015)	NOS: 6 ROBINS-I: Serious	RAPN: higher ASA score, higher CCI
Robotic-assisted versus open partial nephrectomy: A prospective multicenter comparison study of perioperative outcomes (AGILE project)	Minervini et al. 2013 (Italy) [48]	Multicenter without patient matching (RAPN vs. OPN)	*N*_exp_ = 104 *N*_ctrl_ = 198 (Total: 302)	729 days (1/1/2010–31/12/2011)	NOS: 8 ROBINS-I: Moderate	OPN: larger tumor size RAPN: higher CCI
Early single-center experience with robotic partial nephrectomy using the da Vinci Xi: Comparative assessment with conventional open partial nephrectomy	Motoyama et al. 2019 (Japan) [80]	Single-center with patient matching (RAPN vs. OPN)	*N*_exp_ = 37 *N*_ctrl_ = 37 (Total: 74)	2618 days (1/7/2010–31/8/2017)	NOS: 7 ROBINS-I: Low	No special population
						OPN: retroperitoneal approach
Comparison of robotic and open partial nephrectomy: Single-surgeon matched cohort study	Oh et al. 2014 (Korea) [81]	Single-center with patient matching (RPN vs. OPN)	*N*_exp_ = 100 *N*_ctrl_ = 100 (Total: 200)	3683 days (1/5/2003–31/5/2013)	NOS: 8 ROBINS-I: Low	No special population
						No differences
Partial nephrectomy in clinical T1b renal tumors: Multicenter comparative study of open, laparoscopic and robot-assisted approach (the RECORd Project)	Porpiglia et al. 2016 (Italy) [82]	Multicenter without patient matching (RAPN vs. OPN)	*N*_exp_ = 95 *N*_ctrl_ = 133 (Total: 228)	1825 days (1/1/2009–31/12/2013)	NOS: 9 ROBINS-I: Low	No special population
						OPN: older pts
Comparative analysis of robotic-assisted partial nephrectomy versus open partial nephrectomy during the initial robotic learning curve: Does the end justify the means?	Saoud et al. 2017 (Lebanon) [4]	Single-center without patient matching (RAPN vs. OPN)	*N*_exp_ = 15 *N*_ctrl_ = 19 (Total: 34)	760 days (1/7/2013–31/7/2015)	NOS: 5 ROBINS-I: Serious	OPN: higher RENAL score
Comparative analysis of perioperative outcomes between robot-assisted partial nephrectomy and open partial nephrectomy: A propensity-matched study	Sawada et al. 2021 (Japan) [50]	Single-center with patient matching (RAPN vs. OPN)	*N*_exp_ = 58 *N*_ctrl_ = 58 (Total: 116)	5843 days (1/1/2005–31/12/2020)	NOS: 7 ROBINS-I: Low	No special population
						No differences
Comparative outcomes and predictive assessment of Trifecta in open, laparoscopic, and robotic-assisted partial nephrectomy cases with renal cell carcinoma: A 10-year experience at Ramathibodi hospital	Soisrithong et al. 2021 (Thailand) [83]	Single-center without patient matching (RAPN vs. OPN)	*N*_exp_ = 41 *N*_ctrl_ = 18 (Total: 59)	3651 days (1/1/2009–31/12/2018)	NOS: 7 ROBINS-I: Serious	No special population
						No differences
Lower incidence of postoperative acute kidney injury in robot-assisted partial nephrectomy than in open partial nephrectomy: A propensity score-matched study	Tachibana et al. 2020 (Japan) [84]	Multicenter with patient matching (RAPN vs. OPN)	*N*_exp_ = 411 *N*_ctrl_ = 411 (Total: 822)	5478 days (1/1/2004–31/12/2018)	NOS: 8 ROBINS-I: Low	No special population
						No differences
A propensity score-matched comparison of surgical precision obtained by using volumetric analysis between robot-assisted laparoscopic and open partial nephrectomy for T1 renal cell carcinoma: A retrospective non-randomized observational study of initial outcomes	Takagi et al. 2016 (Japan) [8]	Single-center with patient matching (RAPN vs. OPN)	*N*_exp_ = 48 *N*_ctrl_ = 48 (Total: 96)	1095 days (1/1/2012–31/12/2014)	NOS: 7 ROBINS-I: Low	cT1 renal tumors
						No differences
Perioperative and long-term functional outcomes of robot-assisted versus open partial nephrectomy: A single-center retrospective study of a Japanese cohort	Takahara et al. 2022 (Japan) [85]	Single-center with patient matching (RAPN vs. OPN)	*N*_exp_ = 39 *N*_ctrl_ = 39 (Total: 78)	3713 days (1/8/2007–30/9/2017)	NOS: 7 ROBINS-I: Low	No special population
						No differences
Comparison of perioperative, renal and oncologic outcomes in robotic-assisted versus open partial nephrectomy	Tan et al. 2018 (Australia) [86]	Single-center without patient matching (RAPN vs. OPN)	*N*_exp_ = 145 *N*_ctrl_ = 55 (Total: 200)	2556 days (1/1/2010–31/12/2016)	NOS: 8 ROBINS-I: Moderate	OPN: older pts, higher preoperative eGFR, lower RENAL score
Open versus robotic-assisted partial nephrectomy: A multicenter comparison study of perioperative results and complications	Vittori et al. 2014 (Italy) [44]	Multicenter without patient matching (RAPN vs. OPN)	*N*_exp_ = 105 *N*_ctrl_ = 198 (Total: 303)	729 days (1/1/2010–31/12/2011)	NOS: 9 ROBINS-I: Low	OPN: larger tumor size RAPN: higher CCI
A propensity-score matched comparison of perioperative and early renal functional outcomes of robotic versus open partial nephrectomy	Wu et al. 2014 (China) [87]	Single-center with patient matching (RPN vs. OPN)	*N*_exp_ = 51 *N*_ctrl_ = 94 (Total: 145)	1825 days (1/1/2009–31/12/2013)	NOS: 8 ROBINS-I: Low	No special population
						No differences
Predictors of renal function after open and robot-assisted partial nephrectomy: A propensity score-matched study	Yu et al. 2019 (Korea) [88]	Single-center with patient matching (RPN vs. OPN)	*N*_exp_ = 303 *N*_ctrl_ = 303 (Total: 606)	4837 days (1/2/2004–30/4/2017)	NOS: 7 ROBINS-I: Moderate	No special population
						No differences
Open versus robot-assisted partial nephrectomy: A longitudinal comparison of 880 patients over 10 years	Zeuschner et al. 2021 (Germany) [51]	Single-center with patient matching (RAPN vs. OPN)	*N*_exp_ = 500 *N*_ctrl_ = 313 (Total: 813)	4382 days (1/1/2007–31/12/2018)	NOS: 6 ROBINS-I: Moderate	OPN: larger tumor size, higher PADUA score

*RAPN* robot-assisted partial nephrectomy, *OPN* open partial nephrectomy, *NOS* Newcastle–Ottawa Scale, *ROBINS-I* Risk of Bias in Non-randomized Studies of Interventions, *N*_*exp*_ patient population in experimental arm (RPN/RAPN), *N*_*ctrl*_ patient population in control arm (OPN)

**Fig. 4:**
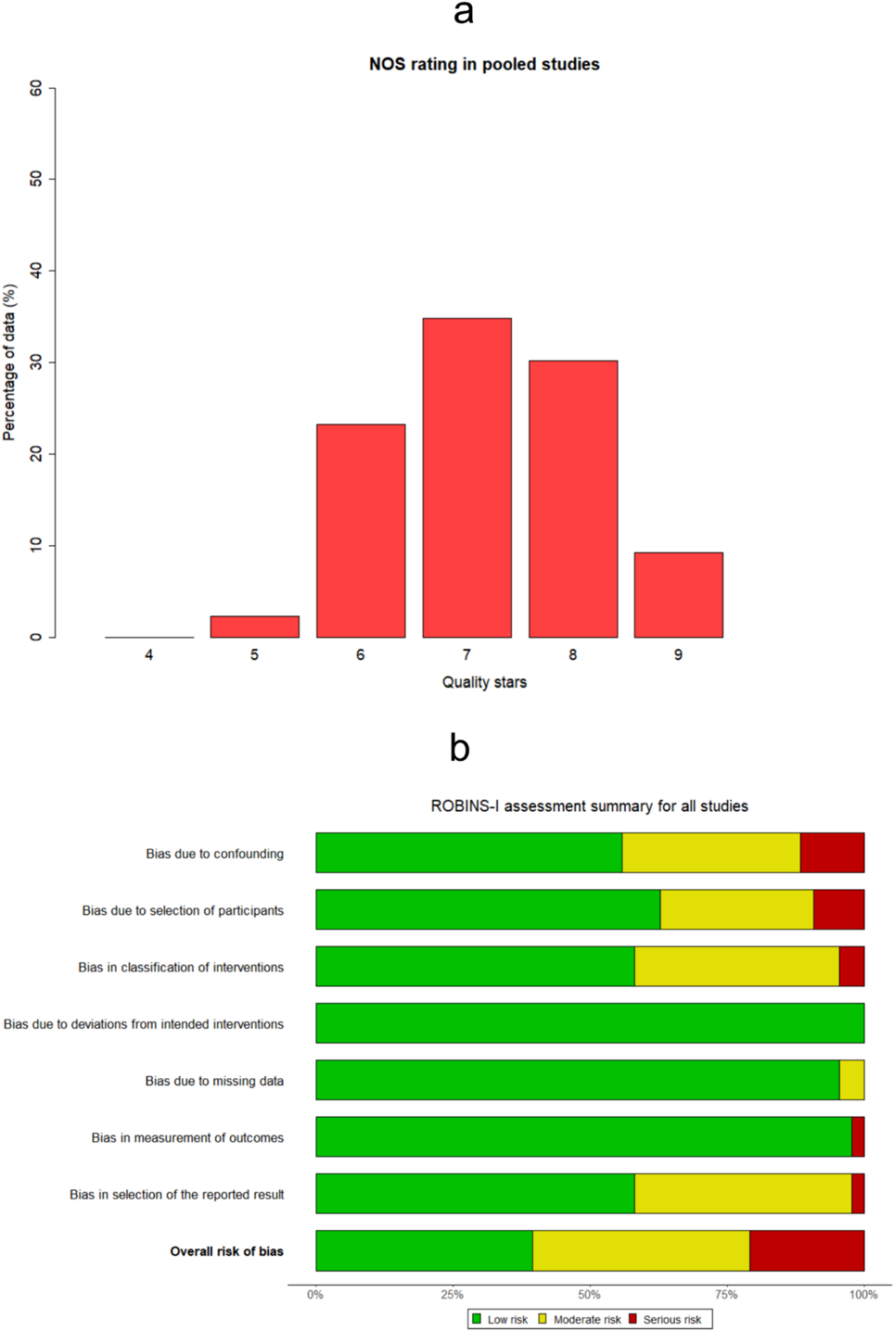
Histogram illustrating the percentages of pooled studies corresponding to each quality grading class according to the Newcastle–Ottawa Scale (**a**). Summary plot illustrating the evaluation of all incorporated studies using the ROBINS-I tool, presenting their percentages stratified by the risk of bias level within each of the seven domains (**b**)

With regard to the NOS qualitative grading, Fig. 4a illustrates the histogram of percentages over the total number of records for each category, based on the count of quality stars assigned. From this graph, a bell-like distribution emerged among studies that received ratings from 5 to 9, with the majority (approximately one-third of the data at the study level) being awarded 7 quality stars. Supplementary Fig. 5 provides the corresponding histograms for the explored subsets. Upon careful inspection of the above diagrams, a relatively higher level of quality was evident for studies published during the last 5 years, those employing patient matching, and those constituting multicenter analyses. More specifically, the clearest qualitative preeminence was observed in matched comparisons, underscoring the significance of the results derived from this particular subset in terms of credibility. In conjunction, concerning the ROBINS-I assessment, the pertinent evaluation forms are concisely presented in the accompanying supplementary material (Supplementary ROBINS-I forms in Supplementary file 3). Particularly, the summary plot pertaining to the pooled data is presented in Fig. 4b, succinctly displaying the percentages over the total number of studies for each ROB category, within each of the seven domains of the tool. Remarkably, no significant deviations from intended interventions were observed. Furthermore, confounding, classification of interventions, and selection bias emerged as the most prevalent risk factors, being present in approximately 40% of the included studies. The relevant SGA indicated a somewhat higher quality status in studies published during the last 5-year period, and a clear advantage in those incorporating group matching, as well as those conducted across multiple centers, in line with the previously presented NOS rating distributions. For further insights, additional summary plots for the subgroups, and the full set of traffic light plots for the pooled analysis and SGA, are provided within Supplementary Figs. 6, 7, 8 and Supplementary Figs. 9, 10, 11, 12, 13, 14, 15, respectively.

### Correlation between EBL & OT

In the preliminary stage of data analysis, we centered on assessing the computational and physiological relevance of the correlation between the original variables (EBL, OT). This evaluation began within the aggregated data, utilizing expected values from all studies in robotic and open PN arms, respectively. Subsequently, a separate examination was conducted for each study arm, using Monte Carlo simulations to indirectly estimate the corresponding r-values [31, 32].

**Fig. 5:**
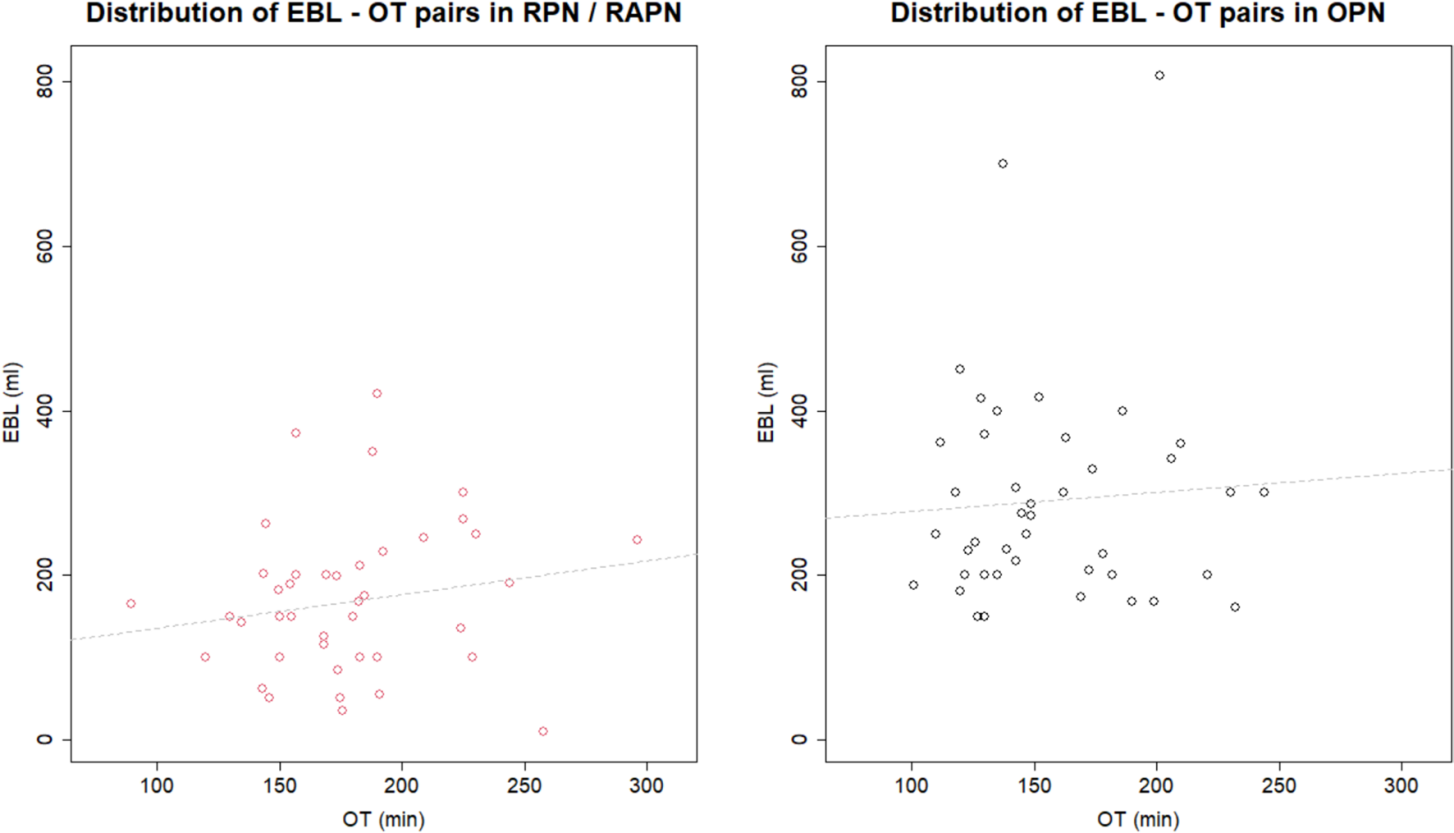
Scatter plots depicting the EBL–OT pairs for the two compared surgical approaches (RPN/RAPN vs. OPN). Each diagram is accompanied by the corresponding correlation, represented by a dashed regression line. It is noted that the plots were constructed using the expected values for each variable from each arm of every included study

The scatterplots presented in Fig. 5 illustrate the EBL—OT pairs for each comparison arm, revealing an increased EBL for the same OT in OPN compared to RAPN. This observation provides insight into the potential disparity in the Q quotient between the two approaches. The overall correlation coefficients between the expected EBL (EV_EBL_) and OT (EV_OT_) in the pooled studies, were 0.1792 for RPN/RAPN and 0.0646 for OPN. Despite variations in the overall r-values between the robotic and open groups in the above diagrams, the difference observed between the two approaches was statistically insignificant (*z*-value = 0.5209, *p*-value = 0.6025). Additionally, the corresponding deviations from zero did not yield statistical significance as well (*t*-value = 1.1459, *p*-value = 0.2585 vs. *t*-value = 0.4093, *p*-value = 0.6845, respectively). Subsequently, sequential Monte Carlo simulations of 10^3^ repetitions were executed to derive an r-value for each comparison arm of every study. This involved the integration of corresponding patient populations, average EBL and OT values, and their respective standard deviations. The objective was to establish a bivariate normal distribution for EBL and OT, ultimately determining the most probable correlation coefficient. Following this process, a dedicated dataset was generated, featuring coefficients at the scale of 10^–3^, which were credibly considered negligible.

The findings presented above suggest a lack of significant interconnection between EBL and OT, essentially reflecting their independence. However, it is important to note that this apparent ‘non-correlation status’ is not universally applicable; it can fluctuate depending on patient selection, the adoption of a multicenter analysis model, surgical experience, annual patient volume, and the implementation of specific treatment protocols. Nevertheless, this observation implies that the complexity of the surgical procedure (indicated by the most positive r-values) and the skills and dexterity of the operating surgeon (indicated by the most negative r-values) contribute almost equally to the formation of the composite “Q” outcome in PN. In light of these findings, it becomes evident that the theoretical framework outlined in the “Materials and methods” section, significantly contributes to elucidating the physiological underpinnings of the computationally derived r-values.

### Meta-analysis of EBL, OT and Q

In the ensuing subsection, the outcomes of the meta-analysis (MA) for EBL, OT, and their quotient *Q* are delineated, employing a random effects model adjusted via the Hartung and Knapp (H–K) method. This analysis relies on the r-values derived from the previously described Monte Carlo simulations, as the coefficients computed collectively for each compared arm exhibited no significant difference from zero or from each other. The dataset under analysis included combined data on EBL and OT from 18,103 patients, of which 9683 underwent a robotic procedure and 8420 received open surgery.

Initially, the effects of the two surgical approaches were separately evaluated regarding each original variable. In terms of EBL, its expected value (EV_EBL_) across all studies and in the RPN/RAPN group was 157 ml, with a CI_95%_ of [131.9; 182]. Conversely, in the OPN group, EV_EBL_ was 278.8 ml, with CI_95%_ = [241.8; 315.9]. As anticipated from existing literature, the divergence was statistically significant, indicating that open surgery is associated with higher average blood loss. The forest plots outlining the respective results from the two approaches are included in Supplementary Figs. 16 and 17. Concerning the OT variable, in the RPN/RAPN group, its expected value (EV_OT_) was 175.9 min, with CI_95%_ = [163.9; 187.9], while in the OPN group, EV_OT_ was 155.9 min, with CI_95%_ = [144.5; 167.3]. In this instance, the difference in procedure duration appears marginally significant, suggesting a slight advantage in terms of brevity on the part of the open approach, aligning with previously available literature data as well. The relevant forest plots, presenting the aforementioned results for robotic and open PN, are provided in Supplementary Figs. 18 and 19, respectively. The outlined findings indicate that EBL is the primary original variable driving the difference in the EBL/OT quotient (MD_Q_) in ml/min. This inference is supported by the notable divergence observed specifically in the overall EV_EBL_ between the robotic and open approaches. The above observation lies in accordance with our previously discussed theoretical framework, indicating that Q inversely reflects surgical precision. This is because aggressive tissue handling in PN typically leads to hemorrhagic adverse effects, without necessarily affecting the procedure duration.

Furthermore, the mean differences in EBL and OT were similarly examined in the context of RPN/RAPN vs. OPN, as respectively, illustrated in the forest plots of Supplementary Figs. 20 and 21. Specifically, the mean difference in average blood loss (MD_EBL_) was − 117.6 ml, with a CI_95%_ of [− 153.4; − 81.7]. In terms of operative duration, the mean difference (MD_OT_) was 19.2 min, with CI_95%_ = [9.1; 29.4]. These findings substantiate our initial hypothesis as for the individual contributions from each original variable to the formulation of Q. Roughly, while RAPN requires an additional 19 min of OT, OPN results in an excess of 118 ml in EBL, implying a tenfold divergence in magnitude (10^1^), which notably impacts their ratio.

Finally, the derived Q-estimates were analyzed within a framework similar to that employed for the original variables. For the expected value of Q in the RAPN group, it was EV_Q_ = 1.03 ml/min, with CI_95%_ = [0.86; 1.20], whereas in the OPN group, it was EV_Q_ = 2.12 ml/min, with CI_95%_ = [1.80; 2.43]. These findings complement the initial framework roughly visualized in the EBL—OT scatterplots of Fig. 5, offering additional insights into MD_Q_. Essentially, they suggest a substantial magnitude in the superiority of robotic PN over open surgery in terms of per-minute blood loss, with this assertion remaining to be further confirmed quantitatively. The corresponding results are depicted in the forest plots of Supplementary Figs. 22 and 23.

### Meta-analysis of MD_Q_

**Fig. 6:**
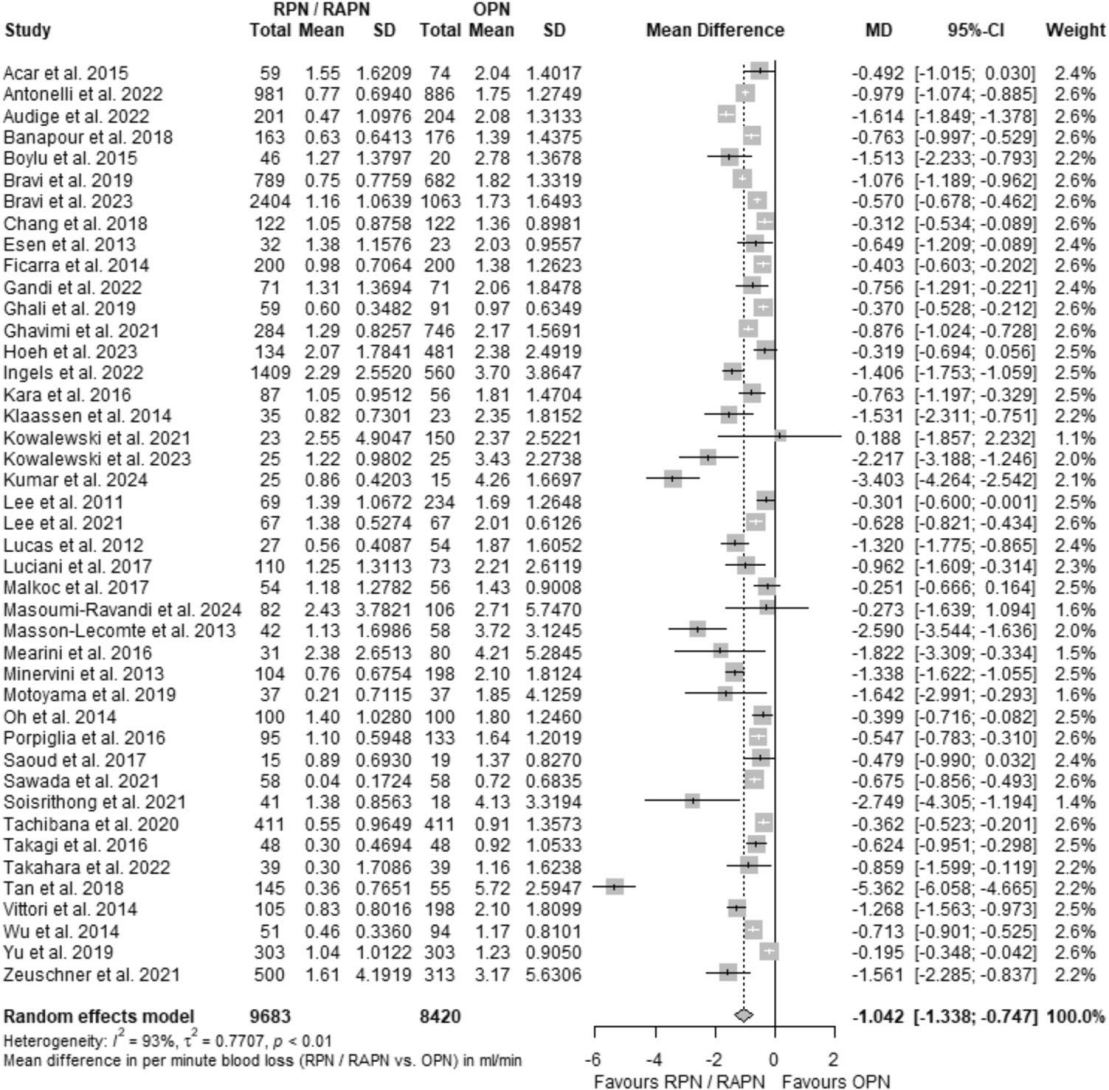
Forest plot showing the comparative effect as the mean difference in Q (MD_Q_) between RPN/RAPN and OPN for all included studies

This section delves into the results of the meta-analysis (MA) for MD_Q_, our primary metric in comparing RAPN and OPN. The analysis was conducted by applying a random effects model and incorporating the Hartung and Knapp (H–K) adjustment, for enhanced accuracy and robustness. The overall effect across the entire cohort of analyzed studies yielded MD_Q_ = − 1.04 ml/min, with a CI_95%_ of [− 1.34; − 0.75]. This result indicates a significant preeminence on the part of robotic over open PN concerning the average blood loss rate, and inductively tissue handling precision as modeled via Q, supporting our initial hypothesis. The forest plot depicting this outcome is presented in Fig. 6.

Nevertheless, it is essential to appreciate the substantial degree of observed heterogeneity (*τ*^2^ = 0.77 with CI_95%_ = [0.52; 1.44], Cochran’s *Q* = 573.36 with *p*-value < 0.0001, and Higgins *I*^2^ = 92.7% with CI_95%_ = [91%; 94%]). Multiple factors contribute to the above observation. Firstly, the inclusion of a considerable number of individual analyses is anticipated to introduce variations, leading to spikes in intra-study variability. Moreover, the use of ratio estimators typically results in reduced standard errors compared to standard deviations within individual patient populations, further accentuating inter-study variability (*τ*^2^). Lastly, the predominant inclusion of non-randomized comparisons with a substantive contribution from small studies (of limited accuracy in their reported outcomes), also adds to the above-observed level of heterogeneity.

**Fig. 7:**
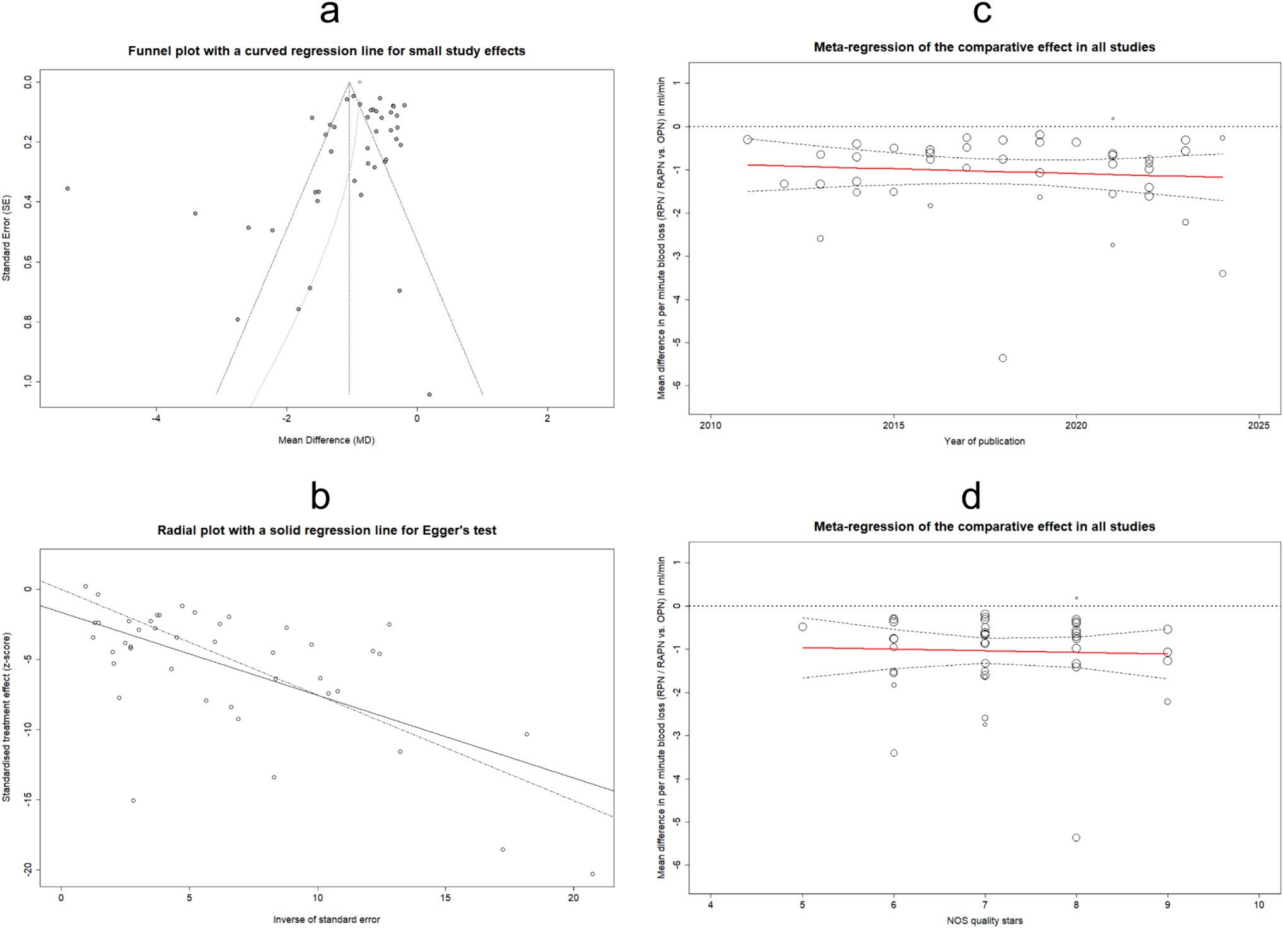
Funnel plot presenting the publication bias assessment in all the examined studies, incorporating a curved regression line to investigate for small study effects (**a**). Radial plot complemented with an integrated regression line, to assess the significance of publication bias using the Egger's test (**b**). Meta-regression analysis plots showing the change in the comparative effect (MD_Q_) between RPN/RAPN vs. OPN, along with the CI_95%_, in the aggregated studies, using as moderator the publication year (**c**) and the score in quality stars based on the NOS scale (d)

Figure 7a displays the funnel plot evaluating publication bias (PB) and the resultant regression curve utilized for modeling small-study effects (SSEs). The plot illustrates a marginal asymmetry toward studies with intermediate and low accuracy in reported outcomes, as estimated through the inverse variance method. A similar funnel plot enhanced with contours indicating specific levels of significance, is available in Supplementary Fig. 24. Additionally, Egger’s test was employed to quantitatively assess the above asymmetry, as illustrated in Fig. 7b, resulting in a magnitude of bias (MB) of − 1.65, with a standard error (SE) of 0.93. Regarding statistical inference, the linear regression resulted in: *t*-value = − 1.78, and p-value = 0.08, essentially indicating marginally non-significant asymmetry. Consequently, the impact of PB appears to be quite minimal, a conclusion also visually supported in the relevant radial plot by the slight divergence between the regression lines corresponding to the captured data (dashed line) and the modeled data via Egger’s test (solid line). Nonetheless, this observation underscores the impact of SSEs (Q-Q' = 41.02, *p*-value < 0.0001), a facet to be further scrutinized in the subsequent “Sensitivity analysis” subsection.

In addition to the aforementioned, a subgroup analysis (SGA) was carried out, considering the publication time point, adoption of patient group matching, number of centers conducting the analysis, and bias risk status. More specifically, for publications after 2018, the findings revealed that MD_Q_ was − 1.18 ml/min, with a CI_95%_ of [− 1.68; − 0.67]. In contrast, for previous publications, MD_Q_ was − 0.84 ml/min, with CI_95%_ = [− 1.10; − 0.58]. Studies incorporating patient matching resulted in an MD_Q_ of − 0.64 ml/min, with CI_95%_ = [− 0.83; − 0.45], while studies without matching exhibited an MD_Q_ of − 1.33 ml/min, with CI_95%_ = [− 1.85; − 0.82]. Additionally, in multicenter studies, MD_Q_ was − 0.88 ml/min, with CI_95%_ = [− 1.14; − 0.62], and in single-center studies, MD_Q_ was − 1.14 ml/min, with CI_95%_ = [− 1.57; − 0.71]. Lastly, studies labeled as “ROBINS-I: Low” exhibited an MD_Q_ of − 0.71 ml/min, with CI_95%_ = [− 0.90; − 0.52], “ROBINS-I: Moderate” studies demonstrated an MD_Q_ of − 1.24 ml/min, with CI_95%_ = [− 1.87; − 0.61], and "ROBINS-I: Serious" studies revealed an MD_Q_ of − 1.23 ml/min, with CI_95%_ = [− 2.11; − 0.35]. The SGA consistently indicated a statistically significant superiority of RAPN over OPN concerning MD_Q_, with no substantial deviations among the investigated subgroups, except for when it pertained to group matching (Cochran’s *Q* for subgroup differences: 6.95, *p*-value = 0.008). The most conservative results were observed in comparisons utilizing patient matching, whereas the least conservative stemmed from studies that omitted the application of a similar protocol, with the former also demonstrating the lowest level of heterogeneity (*I*^2^ = 75.9% with CI_95%_ = [62.8%; 84.3%]). Visual representations of these outcomes are provided in the sequential forest plots of Supplementary Figs. 25, 26, 27, 28. For a concise summary of these analyses, Table 2 compiles the pertinent quantitative outcomes.

Finally, a meta-regression analysis (MRA) delved into both aggregated and subgroup data, utilizing an REML model to establish regression lines for the respective overall effects. Figure 7c illustrates the pooled comparative impact from RAPN and OPN on MD_Q_ concerning the year of publication. Similarly, Fig. 7d displays the corresponding analysis concerning the quality evaluation scores derived from the NOS grading. In both depictions, the effect remains relatively homogenous, irrespective of the temporal or qualitative moderator, showcasing a consistent trend around the overall MD_Q_-estimate from the pooled data. The MRA also encompassed the previously defined subgroup levels. Overall, the comparative effect exhibited a consistent pattern concerning publication year and quality level, with two distinct observations. Firstly, it emerged evidence of a discernible increase in the advantage provided by RAPN with an advancing publication year, particularly noticeable in the subgroups of studies without matching, and those labeled as “ROBINS-I: Serious”. Secondly, the subgroups that provided higher-quality results regarding the accuracy of their findings, as represented by the width of the CI_95%_, pertained to matched analyses, multicenter studies, and comparisons characterized by low ROB. Supplementary Figs. 29, 30, 31 encompass the results from the relevant investigation presented above. Specifically, in the MRA plot focusing on subgroups based on ROB, an increase in result accuracy is highlighted with decreasing bias risk, thus confirming the quality assessment process using the ROBINS-I tool.

### Sensitivity analysis

**Fig. 8:**
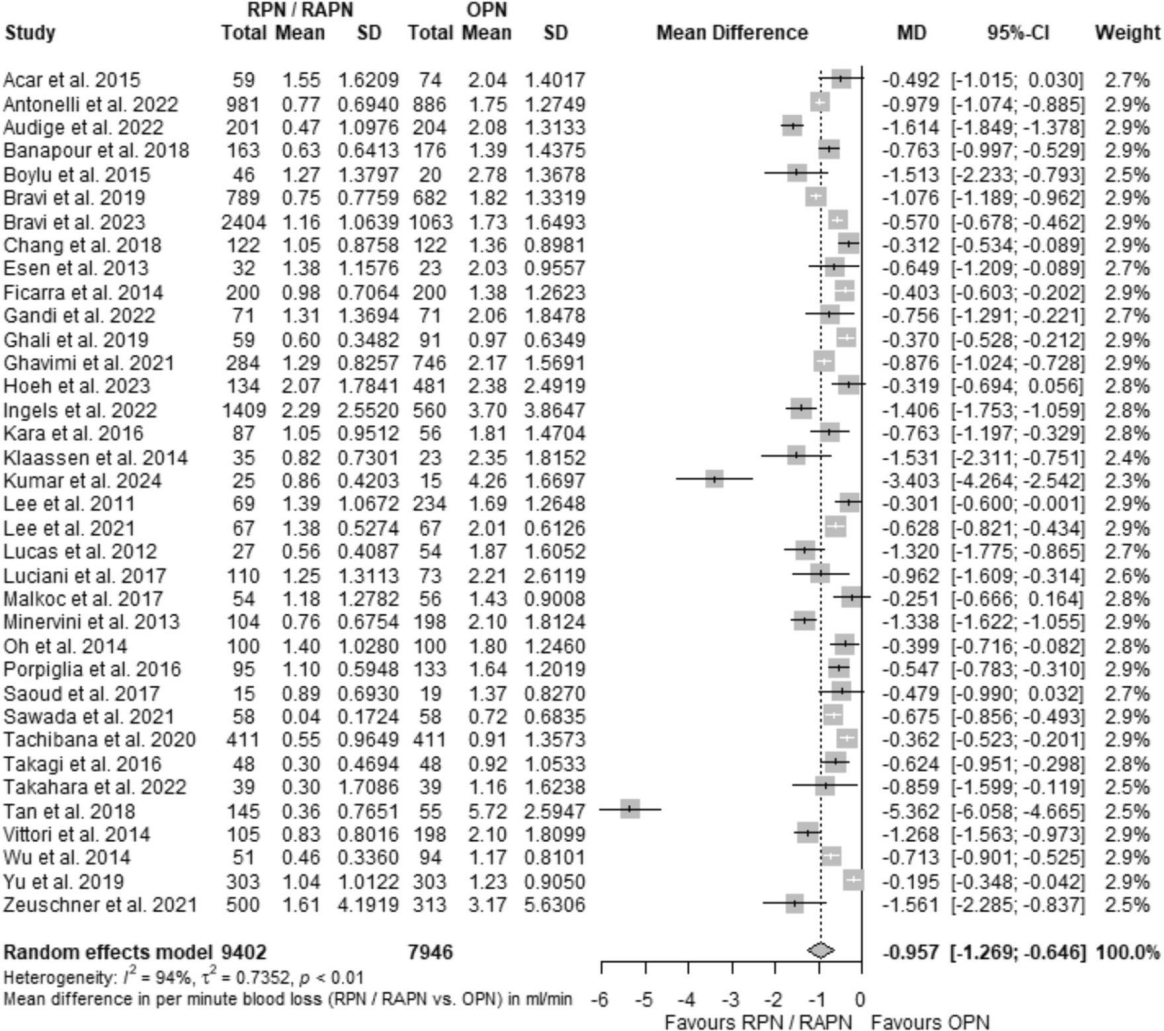
Forest plot showing the comparative effect as the mean difference in Q (MD_Q_) between RPN/RAPN and OPN in the subset of studies with increased accuracy of reported results,  isolated at the first level of the sensitivity analysis

**Fig. 9:**
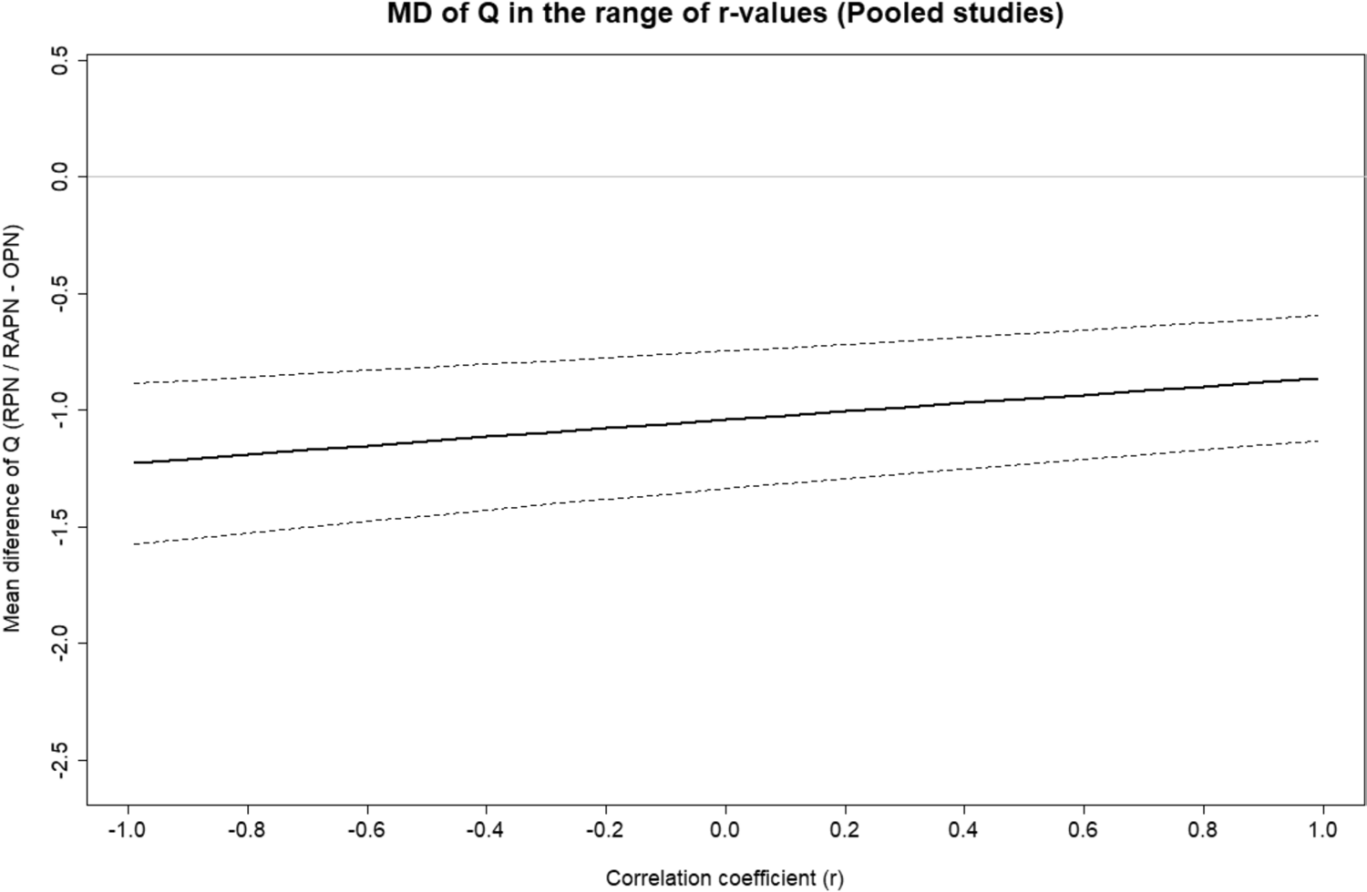
Funnel plot presenting the publication bias assessment in the subset of studies corresponding to the first level of the sensitivity analysis, that incorporates a curved regression line to investigate for small study effects (**a**). The respective radial plot with an embedded regression line, to assess the significance of publication bias using the Egger's test (**b**). Meta-regression analysis plots showing the change in the comparative effect (MD_Q_) between RPN/RAPN vs. OPN, along with the CI_95%_, in the subset of studies corresponding to the first level of the sensitivity analysis using as moderator the publication year (**c**) and the score in quality stars based on the NOS scale (**d**)

Concluding the “Results” section, we elaborate on the findings obtained from a five-tiered SA, as detailed previously in the “Statistical analysis” subsection. Initially, our baseline dataset underwent a refinement wherein seven comparisons, displaying diminished accuracy in reported outcomes, were excluded. The exclusion criterion involved the isolation of studies with a CI_95%_ range up to two standard deviations from the respective pooled ranges in the original study set. This process yielded a subset of 36 records characterized by enhanced accuracy in their reported results. The data pertained to a total of 17,348 patients, of whom 9402 underwent robotic surgery, and 7946 received open intervention. In this refined analysis, the overall estimate for MD was − 0.96 ml/min, with a CI_95%_ of [− 1.27; − 0.65]. Figure 8 illustrates the pertinent forest plot, reaffirming the superior blood loss rate maintenance by the robotic approach compared to open surgery in this specific subset. Despite the aforementioned adjustments, considerable heterogeneity persisted (*τ*^2^ = 0.74 with CI_95%_ = [0.49; 1.46], Cochran's Q = 538.98 with *p*-value < 0.0001, and Higgins *I*^2^ = 93.5% with CI_95%_ = [91.9%; 94.8%]), implying a potential association with the methodology employed in retrieving data for the original variables, ultimately enabling the formulation of the “Q” outcome. Further discussion on the above observation will be detailed in the subsequent section addressing the strengths and limitations of this study. Figure 9a presents the corresponding funnel plot for PB assessment, revealing a substantial restoration of symmetry. Supplementary Fig. 32 provides a similar diagram with contours of statistical significance. The Egger’s test for quantitative PB evaluation, illustrated in Fig. 9b, did not yield statistically significant results (MB = − 1.63 with SE = 1.23, *t*-value = − 1.33, and *p*-value = 0.19); however, the SSEs remained notable (Q-Q' = 26.69, *p*-value < 0.0001).

The first level (L_1_) of SA was broadened to include the subgroups examined earlier. Among records published post-2018, the comparative effect was: MD_Q_ = − 1.13 ml/min, with CI_95%_ = [− 1.72; − 0.54], whereas for studies released before the last 5-year period, it was: MD_Q_ = -0.76 ml/min, with CI_95%_ = [− 0.97; − 0.54]. Studies incorporating patient matching demonstrated the effect: MD_Q_ = -0.59 ml/min, with CI_95%_ = [− 0.75; − 0.43], while those without the implementation of a similar protocol showed: MD_Q_ = − 1.25 ml/min, with CI_95%_ = [− 1.83; − 0.67]. In multicenter studies, the effect was: MD_Q_ = − 0.88 ml/min, with CI_95%_ = [− 1.14; − 0.62], while in single-center studies, it was: MD_Q_ = − 1.02 ml/min, with CI_95%_ = [-1.51; − 0.53]. Additionally, for studies stratified as of low ROB, the effect was: MD_Q_ = − 0.67 ml/min, with CI_95%_ = [− 0.82; − 0.52], whereas for the moderate ROB stratum, it was: MD_Q_ = − 1.22 ml/min, with CI_95%_ = [− 1.89; − 0.54], and for the serious ROB cluster, it was: MD_Q_ = − 1.11 ml/min, with CI_95%_ = [− 2.35; 0.13]. As observed in the pooled analysis, studies that implemented patient matching tended to report outcomes more conservatively, simultaneously demonstrating the lowest heterogeneity (*I*^2^ = 75% with CI_95%_ = [59.8%; 84.4%]), whereas those categorized as "ROBINS-I: Moderate" showed the least conservatism in their MD_Q_-estimates. In summary, this first tier of SA displayed a somewhat more conservative MD_Q_ than our initial approach, and effectively mitigated the influence of small-scale studies on the overall effect. The above findings are deemed more statistically robust, maintaining significance in favor of RAPN over OPN in restraining the Q metric. The complete set of the SA forest plots is presented in Supplementary Figs. 33, 34, 35, 36.

To complement the above subanalysis, a multileveled MRA was conducted in a similar stepwise manner. Regarding the aggregated studies, Fig. 9c presents the evolution of MD_Q_ relative to the year of publication, while Fig. 9d displays the respective change concerning the NOS quality star count. From the aforementioned diagrams, a chronologically and qualitatively consistent advantage emerged in favor of the RPN/RAPN group. At the subgroup level, the MRA revealed a temporally homogeneous effect as well, particularly noticeable in comparisons with patient matching and a multicenter type of analysis. On the other hand, a slight extension of the benefits demonstrated by RAPN was observed in non-matched and single-center studies, both over time and with improving source quality. Detailed results are available through the corresponding MRA plots of Supplementary Figs. 37, 38, 39.

At the second level (L_2_) of SA, the specific subset of studies with low ROB simultaneously applying patient matching was assessed. The study set included 13 records, encompassing a total of 5968 patients, of whom 3633 underwent RPN/RAPN and 2335 underwent OPN. In this sub-analysis, the pooled effect was calculated at MD_Q_ = − 0.56 ml/min, with a CI_95%_ of [− 0.72; − 0.41]. The relevant forest plot is presented in Supplementary Fig. 40a, highlighting the maintenance of significance in favor of robotic interventions regarding the average blood loss rate. It is noteworthy to highlight the efficient mitigation of observed heterogeneity, which was minimized at this point (*τ*^2^ = 0.02 with CI_95%_ = [0.01; 0.59], Cochran’s Q = 33.32 with *p*-value = 0.0009, and Higgins *I*^2^ = 64% with CI_95%_ = [34.7%; 80.1%]); however, its impact remained significant. Furthermore, when examining PB, the generated funnel plots are shown in Supplementary Figs. 40b and 41a, indicating sufficient symmetry around the overall MD_Q_-estimate. Also in this case, the Egger’s test excluded a significant effect from PB (MB = − 1.40 with SE = 0.86, *t*-value = − 1.64, and *p*-value = 0.13), and its output is graphically presented in the radial plot of Supplementary Fig. 41b. On the other hand, the SSEs were somewhat confined in this instance, with their statistical significance formally remaining (Q-Q' = 6.53, *p*-value = 0.01). Finally, the MRA revealed a consistent pooled MD_Q_ both over the years and across the range of NOS grading, as shown in Supplementary Figs. 41c and d, respectively.

In the third level (L_3_), a more compact subset of studies with large sample sizes was isolated. In this case, the selection criterion required that the total number of patients in each individual comparative analysis exceed the average study population in the initial dataset. The final subset comprised 9 studies and included data from 12,660 individuals (approximately 70% of the total population in the initial study set), of which 7215 underwent robotic intervention and 5445 received open surgery. At this level, the overall effect was MD_Q_ = − 0.78 ml/min with a CI_95%_ of [− 1.13; − 0.43], as depicted in the corresponding diagram in Supplementary Fig. 42a. Therefore, the significance of RAPN’s superiority in limiting the average per minute blood loss was maintained in this case as well. However, despite the application of the aforementioned threshold, the impact of heterogeneity remained significant (*τ*^2^ = 0.18 with CI_95%_ = [0.07; 0.82], Cochran’s Q = 163.82 with *p*-value < 0.0001, and Higgins *I*^2^ = 95.1% with CI_95%_ = [92.6%; 96.8%]). Funnel plots generated during the examination of PB are presented in Supplementary Figs. 42b and 43a, with symmetry deemed satisfactory upon inspection. Complementarily, the resultant radial plot graphically displaying the application of Egger’s test is included in Supplementary Fig. 43b, supporting the earlier conclusion regarding the relative absence of PB (MB = 0.60 with SE = 3.63, *t*-value = 0.17, and p-value = 0.87). On the subject of SSEs, in this instance they were practically eliminated due to the predominant inclusion of large-scale studies (Q-Q' = 0.64, *p*-value = 0.42). Finally, as evidenced by the MRA plots in Supplementary Figs. 43c and d, the aforementioned comparative effect between RAPN and OPN emerged as uniformly significant both chronologically and qualitatively.

The fourth level (L_4_) of the SA focused on including only studies conducted by multiple referral centers that also implemented patient matching. The aim was to ensure maximum credibility of the data sources, given the association of these study features with increased NOS scores, as demonstrated in the “Study characteristics” subsection. The emerging study set comprised 5 comparative analyses encapsulating data from a population of 5272 patients, with 3300 in the RPN/RAPN group and 1972 in the OPN group. In this case, the pooled effect was MD_Q_ = − 0.48 ml/min with a CI_95%_ of [− 0.70; − 0.26], remaining statistically significant, while the graphical representation of the respective outcome is included in Supplementary Fig. 44a. At this level, a substantial reduction in heterogeneity was achieved, albeit with its statistical significance persisting (*τ*^2^ = 0.02 with CI_95%_ = [0.00; 0.27], Cochran’s *Q* = 12.93 with *p*-value = 0.01, and Higgins *I*^2^ = 69.1% with CI_95%_ = [20.6%; 87.9%]). In evaluating PB's influence on shaping the overall effect, we generated appropriate funnel plots, as depicted in Supplementary Figs. 44b and 45a, revealing notable symmetry. The quantification of PB through Egger’s test, graphically depicted in Supplementary Fig. 45b, statistically confirmed the absence of a significant impact in this sub-analysis as well (MB = 1.32 with SE = 3.00, *t*-value = 0.44, and *p*-value = 0.69). The SSEs at this level were further constrained to become statistically non-significant (Q-Q' = 0.78, *p*-value = 0.38). Subsequently, MRA in the temporal domain demonstrated a consistently stable and significant comparative effect favoring the robotic approach across the examined time series. Conversely, qualitative MRA indicated a statistically significant advantage through RAPN, with an increasing NOS rating in the included analyses. The relevant diagrams depicting these outcomes are included in Supplementary Figs. 45c and d, respectively. A summary of the results from the aforementioned stages of SA is comprehensively presented in Table 2.

**Table 2:** Meta-analysis results for the pooled studies and subgroups, including the first four levels of the sensitivity analysis

Level of analysis	Level of data	No of studies	Effect in ml/min (MD_Q_ – CI_95%_)	Heterogeneity (*I*^2^–CI_95%_)	PB (MB – SE – *p*-value)	SSEs (Q-Q'–*p*-value)
Pooled analysis (involves all available studies)	Pooled studies	*n* = 43	*– *1.0425 [*– *1.3378; *– *0.7472]	92.7% [91.0%; 94.0%]	MB = − 1.6474 SE = 0.9269 *p* = 0.0829	Q-Q' = 41.02 *p* < 0.0001
	Studies published after 2018	*n* = 24	*– *1.1766 [*– *1.6825; *– *0.6707]	95.2% [93.8%; 96.2%]		
	Studies published before 2018	*n* = 19	*– *0.8401 [*– *1.0999; *– *0.5803]	80.9% [71.1%; 87.4%]		
	Studies with patient matching	*n* = 20	*– *0.6399 [*– *0.8262; *– *0.4536]	75.9% [62.8%; 84.3%]		
	Studies without patient matching	*n* = 23	*– *1.3332 [*– *1.8465; *– *0.8199]	93.9% [92.1%; 95.4%]		
	Multicenter studies	*n* = 13	*– *0.8769 [*– *1.1373; *– *0.6165]	94.0% [91.4%; 95.8%]		
	Single-center studies	*n* = 30	*– *1.1392 [*– *1.5659; *– *0.7124]	91.4% [88.9%; 93.4%]		
	“ROBINS-I: Low” studies	*n* = 17	*– *0.7114 [*– *0.8993; *– *0.5235]	89.1% [84.1%; 92.5%]		
	“ROBINS-I: Moderate” studies	*n* = 17	*– *1.2405 [*– *1.8743; *– *0.6068]	95.6% [94.1%; 96.7%]		
	“ROBINS-I: Serious” studies	*n* = 9	*– *1.2296 [*– *2.1122; *– *0.3471]	87.5% [78.4%; 92.8%]		
Level 1 of sensitivity analysis (involves studies with increased accuracy of reported outcomes based on their CI_95%_ range)	Pooled studies	*n* = 36	*– *0.9574 [*– *1.2690; *– *0.6457]	93.5% [91.9%; 94.8%]	MB = − 1.6340 SE = 1.2277 *p* = 0.1920	Q-Q' = 26.69 *p* < 0.0001
	Studies published after 2018	*n* = 19	*– *1.1313 [*– *1.7184; *– *0.5441]	96.1% [94.9%; 97.0%]		
	Studies published before 2018	*n* = 17	*– *0.7576 [*– *0.9719; *– *0.5432]	79.3% [67.5%; 86.8%]		
	Studies with patient matching	*n* = 17	*– *0.5902 [*– *0.7458; *– *0.4346]	75.0% [59.8%; 84.4%]		
	Studies without patient matching	*n* = 19	*– *1.2479 [*– *1.8251; *– *0.6707]	94.8% [93.1%; 96.1%]		
	Multicenter studies	*n* = 13	*– *0.8769 [*– *1.1373; *– *0.6165]	94.0% [91.4%; 95.8%]		
	Single-center studies	*n* = 23	*– *1.0182 [*– *1.5113; *– *0.5251]	92.6% [90.2%; 94.5%]		
	“ROBINS-I: Low” studies	*n* = 15	*– *0.6702 [*– *0.8248; *– *0.5156]	89.7% [84.7%; 93.0%]		
	“ROBINS-I: Moderate” studies	*n* = 15	*– *1.2161 [*– *1.8901; *– *0.5420]	96.0% [94.6%; 97.0%]		
	“ROBINS-I: Serious” studies	*n* = 6	*– *1.1100 [*– *2.3485; 0.1284]	90.8% [82.7%; 95.1%]		
Level 2 of sensitivity analysis (involves studies of low ROB with patient matching)	Pooled studies	*n* = 13	*– *0.5628 [*– *0.7157; *– *0.4100]	64.0% [34.7%; 80.1%]	MB = − 1.4029 SE = 0.8564 *p* = 0.1296	Q-Q' = 6.53 *p* = 0.0106
Level 3 of sensitivity analysis (involves studies with large sample sizes based on their total patient populations)	Pooled studies	*n* = 9	*– *0.7797 [*– *1.1343; *– *0.4251]	95.1% [92.6%; 96.8%]	MB = 0.6004 SE = 3.6310 *p* = 0.8733	Q-Q' = 0.64 *p* = 0.4247
Level 4 of sensitivity analysis (involves multicenter studies with patient matching)	Pooled studies	*n* = 5	*– *0.4806 [*– *0.6983; *– *0.2630]	69.1% [20.6%; 87.9%]	MB = 1.3158 SE = 3.0001 *p* = 0.6906	Q-Q' = 0.78 *p* = 0.3775

*MD*_*Q*_ mean difference in Q, *CI*_95%_: 95% confidence interval, *ROB* risk of bias according to the ROBINS-I tool, *ROBINS-I* Risk of Bias in Non-randomized Studies of Interventions, *I*^2^ Higgins statistic for heterogeneity assessment, *PB* publication bias, *MB* magnitude of publication bias, *SE* standard error, *SSEs* small study effects, *Q-Q*' deviation of the quantile–quantile plot from the vertical axis due to small study effects, *ml*: milliliters, min: minutes. Note that non-significant findings are denoted in gray font

The final level of the SA (L_5_) delved into the trajectory of MD_Q_ across the theoretical spectrum of values obtained by the correlation coefficient (r), as previously outlined in the “Correlation between EBL & OT” subsection. This examination holds particular relevance in assessing the estimated r-values derived from the Monte Carlo simulation, a process essentially foundational to this subanalysis. Overall, the relevant estimations hinged on the assumption that both the original variables adhere to a bivariate normal distribution. In practical terms, r-values can widely fluctuate among individual studies and between diverse comparison groups. According to the established theoretical framework on the procedural implications of the coefficient, our findings indicate that the relative impacts of “operative intricacy” and “surgical dexterity” on per-minute blood loss (Q), exhibit substantial variability, contingent upon the selected approach (RPN/RAPN vs. OPN) or the study type (e.g., single-center vs. multicenter analyses, examinations of matched vs. non-matched patient groups, comparative studies conducted on the general population vs. specific patient groups, etc.).

**Fig. 10:**
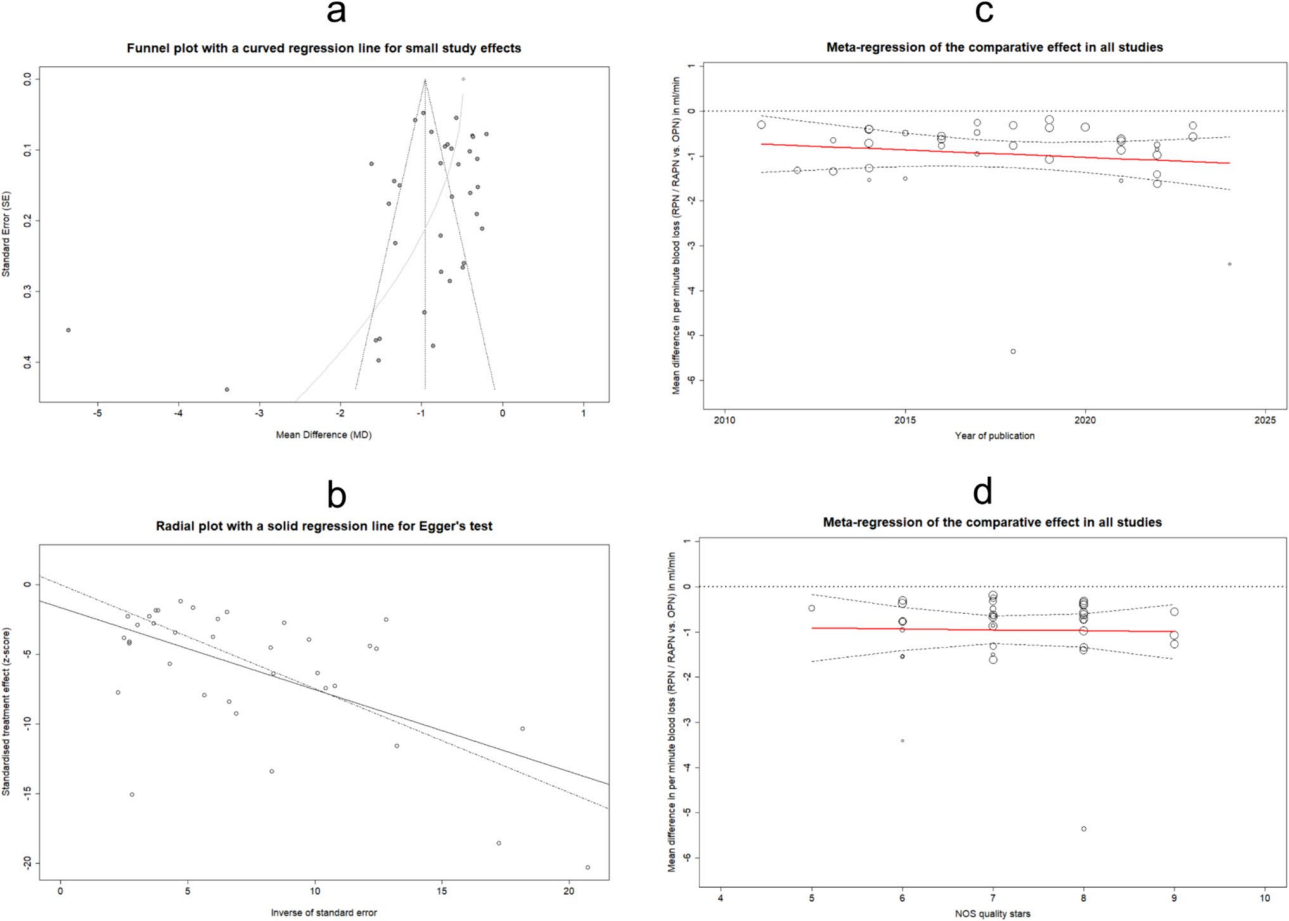
Plot depicting the variation in the comparative effect (MD_Q_) between RPN/RAPN and OPN, along with its CI_95%_, for consecutive r-values, at the  fifth level of the sensitivity analysis and for the total of included studies

The computationally utilized coefficient values spanned from − 0.99 to + 0.99, and Fig. 10 illustrates the variation of MD_Q_ for consecutive r-values within this spectrum, across the pooled data. This graph unveils a gradual narrowing of the statistically significant advantage for RAPN as r progresses from the most negative to the most positive ends of the spectrum. While the difference in MD_Q_ between the extremes of the coefficient does not reach statistical significance, aligning with earlier conceptualizations of their physiological context, it does, however, support a distinct and recognizable trend. Specifically, RAPN demonstrates a seemingly enhanced advantage over OPN in terms of tissue handling precision as modeled by Q, especially when the configuration of the latter is predominantly influenced by the surgeon's dexterity and experience levels, corresponding to the most negative r-values. This finding underscores the potential for robotic systems to boost surgical precision from a technical standpoint, particularly in the realms of surgical skill and operational capacity.

The SA plots generated at the subgroup level (Supplementary Figs. 46 and 47) mirrored the trends observed in Fig. 10. In the above graphs, studies published post-2018, those without comparative matching, those conducted within multiple centers, and those of moderate ROB demonstrated the most precise MD_Q_-estimates. The relevant findings from the pooled analysis and the SGA are provided in the form of forest plots as supplementary material (Sensitivity Analysis forest plots in Supplementary file 4). A detailed and systematic investigation ensued, conducted in a stepwise manner for each r-value. The outcomes of this process are presented in animated plots, also accessible as supplementary material (Supplementary Animated plots in Supplementary file 1). The above analysis involved the estimation of MD_Q_ across the entire range of coefficients (progressing stepwise from *r* = − 0.99 to *r* =  + 0.99 in increments of 0.1), as previously detailed in the “Statistical analysis” subsection. Supplementary animated plot 1 illustrates the funnel plot for PB assessment, including a curved regression line to evaluate the effects from small-scale studies. It illustrates a gradual recession in asymmetry and a decrease in the magnitude of the relevant SSEs, as r-values move towards the most positive end of the spectrum. Additionally, Supplementary animated plot 2 illustrates the respective radial plot with the application of Egger’s test. In this diagram, no significant deviation is observed between the PB regression line for the available studies (dashed line) and that of the Egger’s test (solid line), suggesting that the impact from PB on shaping the overall effect is not particularly substantial.

Concluding the sub-analyses discussed above, Supplementary animated plot 3 illustrates the evolution of the comparative effect in MRA across sequentially ascending r-values, with the publication year as moderator and encompassing all available studies. A detailed examination of this plot reveals a gradual homogenous constriction in MD_Q_ as r approaches + 0.99, consistent with the pattern explained previously in Fig. 10. Similar diagrams within the context of SGA are provided in Supplementary animated plots 4–10. Increased precision in the presented results and a more distinct significance in the advantage provided by RAPN arose from matched comparisons, multicenter analyses, as well as studies of low ROB. This occurrence serves to bolster the credibility of the aforementioned findings. A parallel trend is evident in Supplementary animated plot 11, where the number of quality stars according to NOS is employed as the moderator. This progressive shrinkage of the advantage provided by RAPN over OPN, observed in the pooled analysis, is also discernible in subgroup analyses with the NOS grading serving as the moderator, as demonstrated in Supplementary animated plots 12–15. In this case, the most cohesive results originated from the subset of multicenter studies.

## Discussion

### Discussion of results

The objective of the present study was to define a novel parameter that assesses surgical precision in PN, by reflecting the average blood loss per minute. Initial evaluation of the original variables revealed a correlation between RAPN and reduced blood loss, whereas OPN was only marginally associated with shorter operative durations. In their overall content, these interconnections align with the majority of the available comparative literature between the two approaches [33, 34]. In the preliminary data exploration, the MDs for each original variable were computed across all eligible studies. The mean difference in average blood loss (MD_EBL_) favored RAPN by 118 ml, while in terms of operative time (MD_OT_), OPN was favored by 19 min. The respective deviation between these outcomes falls within the order of magnitude of 10, indicating that blood loss predominantly influences the EBL / OT ratio, represented by the newly introduced Q variable. A similar conclusion can be drawn from our assessment of the correlation between the original variables used to estimate Q. Figure 5 highlighted that, when controlling for OT, EBL is higher in OPN procedures. These findings intuitively support the hypothesis that Q potentially represents surgical precision in PN procedures, by inversely expressing the combined influence of surgical skills and dissection demands primarily on blood loss, with a secondary impact on operative time. With respect to the Q variable, we initially conducted a quantitative approximation of its expected value (EV_Q_) separately for each surgical approach. Subsequently, we conducted a multilevel meta-analysis with the aim to estimate the mean difference (MD_Q_) between RAPN and OPN.

Having established that EBL is the primary driver of the Q quotient, at this point we will endeavor to emphasize the implications arising from a macroscopic extrapolation of Q- and MD_Q_-estimates in theoretical transfusion needs. This pertains to the blood volumes required for a precisely balanced replenishment of the predicted EBL, as indicated below with the asterisk symbol. Firstly, by rearranging the Q quotient, we are able to directly predict the total blood loss in each comparison arm ${\text{(EBL}}^{*} )$, by utilizing the Q-estimates and expected procedure durations (EV_OT_) in RPN/RAPN and OPN groups respectively, according to Eq. (4). In the same manner, it is possible to predict the average difference in EBL between the two approaches $\left( {{\Delta }\left\{ {{\text{EBL}}^{*} } \right\}} \right)$ by utilizing Eq. (5). This prediction lies on the basis of the resulting MD_Q_ estimates for the typical duration of a robotic or open PN, with the latter being standardized as the average ${\text{(OT}}_{{{\text{std}}}} {)}$. The transformation becomes feasible due to the clinically marginal MD_OT_ between the two approaches, estimated at around 11% & 12% of the total OT in RPN/RAPN & OPN, respectively. The above-described predictions aim to quantify the “Macroscopic” disparity between the compared modalities, focusing on the primary driver of the effect. This involves estimating the impact from Q and MD_Q_ in the context of surgical precision through dimension reduction, with EBL considered a more tangible parameter for optimal interpretation.

4 $${\text{EBL}}^{*} {\text{ = Q }} \cdot {\text{ EV}}_{{{\text{OT}}}}$$

5 $$\Delta \left\{ {EBL^{*} } \right\}{\text{ = MD}}_{{\text{Q}}} \, \cdot {\text{OT}}_{{{\text{std}}}}$$

Further engaging with the aforementioned, in the “Meta-analysis of EBL, OT & Q” subsection, the expected Q-estimates (EV_Q_) for RAPN and OPN were 1.03 ml/min and 2.12 ml/min, respectively. Additionally, the expected operative durations (EV_OT_) were 175.9 min and 155.9 min, respectively. Consequently, regarding the prediction of EBL for each approach according to Eq. (4), blood loss volumes $${\text{(EBL}}^{*} )$$ of 181.3 ml and 330 ml are predicted for the robotic and open surgery groups respectively. By converting the above quantities to packed red blood cells (PRBC: approximately 350 ml per unit) and whole blood (WB: approximately 500 ml per unit) [35], we can macroscopically demonstrate the disparity induced by the achieved surgical precision in each approach, based upon the expected duration of each procedure. Thus, concerning RAPN, the predicted blood loss volume clinically translates into $${\raise0.7ex\hbox{$1$} \!\mathord{\left/ {\vphantom {1 2}}\right.\kern-0pt} \!\lower0.7ex\hbox{$2$}}$$ PRBC and $${\raise0.7ex\hbox{$1$} \!\mathord{\left/ {\vphantom {1 3}}\right.\kern-0pt} \!\lower0.7ex\hbox{$3$}}$$ WB units, while in OPN, the aforementioned transformations correspond to approximately 1 PRBC and $${\raise0.7ex\hbox{$2$} \!\mathord{\left/ {\vphantom {2 3}}\right.\kern-0pt} \!\lower0.7ex\hbox{$3$}}$$ WB units.

Furthermore, in terms of the predicted difference in EBL between RAPN and OPN $$\left( {\Delta \left\{ {{\text{EBL}}^{*} } \right\}} \right)$$, upon analyzing the available data we observed a statistically significant disparity in Q, evident at both pooled and subgroup levels. In the pooled analysis, this difference was estimated at: MD_Q_ = − 1.04 ml/min. At this point, considering the mean PN durations for RAPN (EV_OT_ = 175.9 min) and OPN (EV_OT_ = 155.9 min), we can infer the average expected OT for both approaches at: OT_std_ = 165.9 min. Based on the above MD_Q_, an additional 173 ml of blood loss is predicted for OPN integrated over a standard-duration robotic or open PN. This difference clinically corresponds to $${\raise0.7ex\hbox{$1$} \!\mathord{\left/ {\vphantom {1 2}}\right.\kern-0pt} \!\lower0.7ex\hbox{$2$}}$$ PRBC and $${\raise0.7ex\hbox{$1$} \!\mathord{\left/ {\vphantom {1 3}}\right.\kern-0pt} \!\lower0.7ex\hbox{$3$}}$$ WB units. Consequently, the magnitude of this adjusted difference strongly supports the differentiation in surgical precision between the robotic and open approaches, as represented by the Q metric. The most conservative findings were derived from studies utilizing patient matching, which simultaneously demonstrated the lowest level of heterogeneity in the SGA of the initially retrieved data (Higgins *I*^2^ = 75.9%), with an MD_Q_ of − 0.64 ml/min. When translated to blood loss over the standard PN duration, this amounts to 106.2 ml of excessive volume in OPN, representing almost $${\raise0.7ex\hbox{$1$} \!\mathord{\left/ {\vphantom {1 3}}\right.\kern-0pt} \!\lower0.7ex\hbox{$3$}}$$ PRBC and $${\raise0.7ex\hbox{$1$} \!\mathord{\left/ {\vphantom {1 5}}\right.\kern-0pt} \!\lower0.7ex\hbox{$5$}}$$ WB units. In contrast, the least conservative findings were observed in studies lacking comparative matching, with MD_Q_ = − 1.33 ml/min, reflecting 221.2 ml more blood loss in OPN, which corresponds to approximately $${\raise0.7ex\hbox{$2$} \!\mathord{\left/ {\vphantom {2 3}}\right.\kern-0pt} \!\lower0.7ex\hbox{$3$}}$$ PRBC and $${\raise0.7ex\hbox{$2$} \!\mathord{\left/ {\vphantom {2 5}}\right.\kern-0pt} \!\lower0.7ex\hbox{$5$}}$$ WB units. Therefore, even under the most conservative scenario, the notable divergency between RAPN and OPN substantially reinforces the former's apparent superiority in the context of surgical precision, as indicated by the Q variable when viewed from a broader perspective.

In the initial stage of SA (L_1_), the analysis focused on studies with enhanced accuracy in reported outcomes. At this level, the MD_Q_ was − 0.96 ml/min, indicating an additional volume of 158.8 ml in blood loss for OPN within a standard PN duration, accounting for about $${\raise0.7ex\hbox{$2$} \!\mathord{\left/ {\vphantom {2 5}}\right.\kern-0pt} \!\lower0.7ex\hbox{$5$}}$$ PRBC and $${\raise0.7ex\hbox{$1$} \!\mathord{\left/ {\vphantom {1 3}}\right.\kern-0pt} \!\lower0.7ex\hbox{$3$}}$$ WB units. The most conservative results, observed under minimal heterogeneity (Higgins *I*^2^ = 75%), were once again obtained from patient-matched analyses. These studies displayed an MD_Q_ of − 0.59 ml/min, equivalent to 97.9 ml of additional blood loss for OPN, practically approximating $${\raise0.7ex\hbox{$3$} \!\mathord{\left/ {\vphantom {3 {10}}}\right.\kern-0pt} \!\lower0.7ex\hbox{${10}$}}$$ PRBC and $${\raise0.7ex\hbox{$1$} \!\mathord{\left/ {\vphantom {1 5}}\right.\kern-0pt} \!\lower0.7ex\hbox{$5$}}$$ WB units. In this case as well, the least conservative findings emerged from studies with unmatched populations, registering an MD_Q_ of − 1.25 ml/min, essentially resulting in 207 ml more blood loss for OPN, and representing $${\raise0.7ex\hbox{$3$} \!\mathord{\left/ {\vphantom {3 5}}\right.\kern-0pt} \!\lower0.7ex\hbox{$5$}}$$ PRBC and $${\raise0.7ex\hbox{$2$} \!\mathord{\left/ {\vphantom {2 5}}\right.\kern-0pt} \!\lower0.7ex\hbox{$5$}}$$ WB units. In L_2_ of the SA, the investigation was conducted within the subset of low ROB analyses where patient matching was applied, to enhance the credibility of the derived estimates. In this subset, where heterogeneity was compressed to its lowest levels (Higgins *I*^2^ = 64%), the pooled MD_Q_ emerged at − 0.56 ml/min, translating to an additional 93.4 ml of predicted blood loss for OPN in a standard-duration PN, corresponding to almost $${\raise0.7ex\hbox{$1$} \!\mathord{\left/ {\vphantom {1 4}}\right.\kern-0pt} \!\lower0.7ex\hbox{$4$}}$$ PRBC and $${\raise0.7ex\hbox{$1$} \!\mathord{\left/ {\vphantom {1 5}}\right.\kern-0pt} \!\lower0.7ex\hbox{$5$}}$$ WB units. Subsequently, in L_3_ of the SA, the sub-analysis focused on studies with large sample sizes, with the pooled MD_Q_ estimated at -0.78 ml/min. Based on this difference, we can predict an additional blood loss from open surgery amounting to 129.4 ml over OT_std_, equivalent to just over $${\raise0.7ex\hbox{$1$} \!\mathord{\left/ {\vphantom {1 3}}\right.\kern-0pt} \!\lower0.7ex\hbox{$3$}}$$ PRBC and $${\raise0.7ex\hbox{$1$} \!\mathord{\left/ {\vphantom {1 4}}\right.\kern-0pt} \!\lower0.7ex\hbox{$4$}}$$ WB units. Finally, in L_4_ of the SA, the estimation of MD_Q_ was conducted in the subset of patient-matched studies employing a multicenter analysis approach, aimed at bolstering the robustness of conclusions and mitigating the initially observed heterogeneity. At this level, the difference between RAPN and OPN was estimated at -0.48 ml/min, predicting an additional 79.7 ml for the latter during OT_std_, which translates to approximately $${\raise0.7ex\hbox{$1$} \!\mathord{\left/ {\vphantom {1 4}}\right.\kern-0pt} \!\lower0.7ex\hbox{$4$}}$$ PRBC and $${\raise0.7ex\hbox{$1$} \!\mathord{\left/ {\vphantom {1 6}}\right.\kern-0pt} \!\lower0.7ex\hbox{$6$}}$$ WB units.

From the perspective of coherence, driven by the degree of observed heterogeneity as indicated in Table 2, the most reliable MD_Q_-estimates (based on study features) emerged from the matched comparisons, as well as in the L_2_ & L_4_ stages of the SA. In particular, the observed differences in Q led to the prediction of a decrease in modeled transfusion needs by 20–30% in PRBC units, and 15–20% in WB units, during the standardized duration of either a robotic or open intervention. The aforementioned proportions were considered sufficiently substantial to justify a clinically impactful difference in Q-based surgical precision between RPN/RAPN and OPN. The intention behind the above calculations and transformations was not solely to highlight the EBL differences between the two approaches, as a significant portion of existing comparative literature centers on this specific aspect. Rather, the aim was to underscore the implications of the significant divergence in Q between RAPN and OPN, factored over the average OT between both approaches. These impactful findings regarding the primary Q driver (EBL) serve as a benchmark for evaluating surgical precision, affirming the clinical relevance of this novel variable. More specifically, they indicate that the discrepancy in per-minute blood loss between robotic and conventional open PN holds tangible physiological implications and practical ramifications. The relevant results from the aforementioned predictions of blood loss and transformations into transfusion volumes are summarized in Table 3.

**Table 3:** Conversion of findings regarding Q and MD_Q_ into macroscopic estimations of blood loss. In the first part of the table, the transformations of Q-estimates derived from the analysis of pooled data for each approach, are presented as percentages of PRBC and WB units according to the expected OT in each case. In the second part, the transformations of MD_Q_-estimates at each level of analysis and data, are highlighted as differences in blood loss between RPN/RAPN and OPN, expressed as percentages of PRBC and WB units based on the average OT between the two approaches

Level of analysis	Framework of comparison	Q- & MD_Q_-estimates (ml/min)	Operative time (min)	Predicted blood loss (ml)	Percentages of PRBC and WB units (%)	
Conversion of Q-estimates into macroscopic blood loss for each surgical approach (based on OT_exp_ and OT_ctrl_)						
Pooled analysis (Involves all available studies)	Surgical Approach	EV_Q_	EV_OT_	EBL^*^	PRBC_350 ml_	WB_500 ml_
	RPN/RAPN	1.031	175.867	181.319	51.80%	36.30%
	OPN	2.116	155.939	329.967	94.30%	66.00%
Conversion of MD_Q_-estimates into macroscopic blood loss difference between RAPN and OPN (based on OT_std_)						
Pooled analysis (Involves all available studies)	Level of Data	MD_Q_	OT_std_	Δ{EBL^*^}	Δ{PRBC_350 ml_}	Δ{WB_500 ml_}
	Pooled studies	*– *1.0425	165.903	*– *172.954	*– *49.40%	*– *34.60%
	Studies published after 2018	*– *1.1766	165.903	*– *195.201	*– *55.80%	*– *39.00%
	Studies published before 2018	*– *0.8401	165.903	*– *139.375	*– *39.80%	*– *27.90%
	Studies with patient matching	*– *0.6399	165.903	*– *106.161	*– *30.30%	*– *21.20%
	Studies without patient matching	*– *1.3332	165.903	*– *221.182	*– *63.20%	*– *44.20%
	Multicenter studies	*– *0.8769	165.903	*– *145.48	*– *41.60%	*– *29.10%
	Single-center studies	*– *1.1392	165.903	*– *188.997	*– *54.00%	*– *37.80%
	“ROBINS-I: Low” studies	*– *0.7114	165.903	*– *118.023	*– *33.70%	*– *23.60%
	“ROBINS-I: Moderate” studies	*– *1.2105	165.903	*– *200.826	*– *57.40%	*– *40.20%
	“ROBINS-I: Serious” studies	*– *1.2296	165.903	*– *203.994	*– *58.30%	*– *40.80%
Level 1 of sensitivity analysis (Involves studies with increased accuracy of reported outcomes based on their CI_95%_ range)	Pooled studies	*– *0.9574	165.903	*– *158.836	*– *45.40%	*– *31.80%
	Studies published after 2018	*– *1.1313	165.903	*– *187.686	*– *53.60%	*– *37.50%
	Studies published before 2018	*– *0.7576	165.903	*– *125.688	*– *35.90%	*– *25.10%
	Studies with patient matching	*– *0.5902	165.903	*– *97.916	*– *28.00%	*– *19.60%
	Studies without patient matching	*– *1.2479	165.903	*– *207.03	*– *59.20%	*– *41.40%
	Multicenter studies	*– *0.8769	165.903	*– *145.48	*– *41.60%	*– *29.10%
	Single-center studies	*– *1.0182	165.903	*– *168.922	*– *48.30%	*– *33.80%
	“ROBINS-I: Low” studies	*– *0.6702	165.903	*– *111.188	*– *31.80%	*– *22.20%
	“ROBINS-I: Moderate” studies	*– *1.2161	165.903	*– *201.755	*– *57.60%	*– *40.40%
	“ROBINS-I: Serious” studies	*– *1.11	165.903	*– *184.152	*– *52.60%	*– *36.80%
Level 2 of sensitivity analysis (Involves studies of low ROB with patient matching)	Pooled studies	*– *0.5628	165.903	*– *93.37	*– *26.70%	*– *18.70%
Level 3 of sensitivity analysis (Involves studies with large sample sizes based on their total patient populations)	Pooled studies	*– *0.7797	165.903	*– *129.355	*– *37.00%	*– *25.90%
Level 4 of sensitivity analysis (Involves multicenter studies with patient matching)	Pooled studies	*– *0.4806	165.903	*– *79.733	*– *22.80%	*– *15.90%

*RPN* robotic partial nephrectomy, *RAPN* robot-assisted partial nephrectomy, *OPN* open partial nephrectomy, *MD*_*Q*_ mean difference in Q, *EV* expected value, *OT* operative time, *EBL* estimated blood loss, EBL^*^ predicted volume of blood loss via Q or MD_Q_, *PRBC* packed red blood cells (approximately: 350 ml/unit), *WB* whole blood (approximately: 500 ml/unit), OT_std_: average EV_OT_ between RPN/RAPN and OPN groups, *ROBINS-I* Risk of Bias in Non-randomized Studies of Interventions, *ml* milliliters, *min* minutes. Note that the symbol "Δ" indicates the difference between the robotic and open approaches in terms of predicted blood loss volumes

In L_5_ of the SA, we probed into the impact of the interconnection between EBL and OT on MD_Q_. In particular, we noticed a trend where the maximum advantage from RAPN (in terms of Q restriction) was amplified for the most negative r-values. Based on the theoretical groundwork of the coefficient’s physiological implications, these extreme values correspond to procedures where the surgeon's skill and experience predominantly affect the per-minute blood loss [36, 37]. However, the lack of statistical significance in the deviation between the two poles of the coefficient’s spectrum, hints at the concomitant influence from parameters linked to the complexity of tissue dissection. These computational outcomes resonate with extensive literature supporting the pivotal role of robotic technology in improving ergonomics and facilitating intricate intracorporeal procedures [38–41]. As a result, the above observation suggests that robotic platforms may provide a more pronounced benefit in cases requiring a relative enhancement in surgeon's dexterity, rather than those inherently involving heightened complexity in performing the procedure, such as reoperations, concurrent retroperitoneal conditions, and highly complex renal tumors. The aforementioned perspective is supported by relevant meta-analyses comparing the two approaches in patient populations with high complexity tumors. In these instances, characterized by increased difficulty in managing renal parenchyma, no significant differences were observed in terms of EBL, which constitutes the driving factor for the Q quotient. Conversely, the contradictory outcomes concerning OT, also align with our hypothesis regarding its secondary influence on shaping the novel variable [42, 43]. Finally, in terms of the MRA findings observed in both the pooled data and subgroups, they consistently indicated a favorable comparative effect on the part of RAPN on MD_Q_, with respect to the year of publication and the NOS quality status.

### Discussion on existing evidence

In an overall evaluation of the RPN/RAPN vs. OPN comparison, Vittori et al. conducted a multicenter study in Italy, contrasting patients undergoing robotic procedures with those assigned to receive open surgery. Their analysis revealed that RPN was linked to longer OT, reduced EBL, and shorter hospital stays. Moreover, the observed variations in positive surgical margin (PSM) and intraoperative complication rates revealed no significant distinctions between the two groups. RPN was independently related with a more favorable risk of postoperative complications, offering considerable benefits, particularly in minimizing blood loss volumes and mitigating specific adverse events such as urinary fistula and postprocedural bleeding compared to OPN [44]. Within the realm of technical aspects among minimally invasive modalities, several studies comparing RAPN and LPN have confirmed the superiority of the former, citing a more favorable learning curve and optimal ergonomics [45]. Furthermore, in the context of the most commonly explored outcomes, Lee et al. compared RAPN and OPN in patients with renal masses larger than 4 cm in their maximum diameter. Despite the fact that RAPN exhibited longer operative and ischemic times, it resulted in a shorter length of stay (LOS). Notably, average EBL, pain assessment scores, and complication rates were comparable across the groups, however, RAPN demonstrated a superior 6-month estimated glomerular filtration rate (e-GFR). The observed differences regarding recurrence-free and cancer-specific survival were statistically insignificant, and the study concluded that RAPN is a safe and feasible alternative to OPN for treating larger renal tumors, showing comparable surgical, functional, and oncological outcomes [46]. In the scope of tissue handling quality, Takagi et al. comparatively explored the surgical outcomes of RAPN vs. OPN, focusing on surgical precision by leveraging volumetric data in a propensity score-matched study. In their investigation, 100 patients underwent robotic PN, while 179 received open surgery. Their findings indicated no notable distinctions in overall postoperative renal function and viable parenchymal volume preservation. Both groups displayed similar rates of urological complications and PSM, but RAPN exhibited lower EBL and a shorter LOS than OPN [8]. In assessing operative efficiency, Ghali et al. conducted a comparison between OPN and RPN for cT2a renal masses. Their main focus was on the composite outcome of Trifecta, which represents the quality level of tumor resection in PN from a therapeutic perspective. Their study identified a higher accomplishment rate of the Trifecta outcome in the RPN group, indicating improved surgical quality. RPN was also linked to a lower incidence of major complications, reduced EBL, shorter LOS, and comparable PSM rates and functional recovery compared to OPN. The authors proposed that RPN might serve as a primary option for select patients with cT2a renal masses, especially in high-volume referral centers [47]. Additionally, within the framework of oncological outcomes, previous studies comparing RPN and OPN for cT2 tumors have cited similar results, with RPN demonstrating substantial short-term superiority regarding LOS. Moreover, RPN has shown progressive improvements in limiting EBL and the overall suppression of complication rates over the years [48].

In fact, numerous comparative studies underscore the advantages of RAPN over OPN, and a substantial body of literature consistently reveals that the robotic approach exhibits a pattern of lower EBL, slightly extended OT, and shorter LOS, as previously noted [49]. In a relevant study by Sawada et al., the researchers conducted a thorough investigation of perioperative outcomes between RAPN and OPN, adjusting for patient and tumor characteristics through propensity score matching. Their analysis indicated that the former excelled in terms of EBL, IT, and LOS, while insignificant differences were observed with respect to perioperative complications and PSM incidence. Additionally, RAPN exhibited a superior profile in preserving postoperative renal function, and demonstrated lower rates of CKD upstaging. The preservation of renal function was attributed to reduced nephron loss and a shorter WIT, indicating a relative advantage in surgical precision with respect to tissue handling and ischemia application, respectively [50]. Zeuschner et al. conducted a study contrasting 500 RAPN with 313 OPN procedures, performed at a single center between 2007 and 2018. Their results suggested that RAPN had lower complication rates, less EBL, and shorter LOS. While OPN was correlated with a shorter OT, this study strongly asserts the overall superiority of RAPN. However, it acknowledged that the full benefits of robotic surgery may take time to manifest due to the associated learning curve, implying a significant impact from the surgeon's technical skill level [51]. This collective body of research consistently upholds the notion that RAPN yields outcomes at least comparable to OPN, irrespective of the complexity of renal tumors. The above advocates that the robotic approach is not only a viable option but also provides specific advantages, such as improved visualization, optimized ergonomics, and possibly superior surgical precision. Consequently, this extensive exploration of EBL and OT within the main body of comparative literature sparked our interest in investigating their ratio, representing the per-minute blood loss. Utilization of this quotient (Q) proved effective in offering a more detailed insight into the inherent characteristics of each approach with respect to the degree of precision in renal parenchyma dissection. Moreover, it enabled us to assess the combined impact of factors associated with the treating surgeon's technical expertise and the inherent challenges of conducting PN, in shaping the attainable level of tissue handling quality.

### Strengths and limitations

The present exploration showcases both assets and constraints while constituting a unique contribution to the field. A notable strong-point lies in its innovative approach, introducing the novel variable of per-minute blood loss (Q), previously absent in the initial dataset. The primary objective was to reveal the comparative impact of RAPN and OPN on this metric, suggesting its potential as an indicator of surgical precision in PN. This conceptualization is grounded on the vascular-rich nature of the kidney, where any procedure involving excision of parenchymatous segments inevitably leads to blood loss, since renal blood flow ranges between 20 and 25% of the total cardiac output [52, 53]. Acknowledging the demand for increased delicacy during intricate maneuvers adjacent to the renal hilum, the study recognizes that precision may be inversely reflected in the average rate of blood loss in this specific case as well. Another commendable aspect lies in the incorporation of a concise triad of outcomes (EBL, OT, and Q) for a comprehensive analysis. The inclusion of an extensive dataset sourced from a wide body of the relevant international literature enhances the study's robustness, while the meticulous database search and inclusion criteria application underscore transparency in its methodology. Furthermore, rigorous data analysis, covering both pooled data and diverse subgroups, strengthens the inferred results. Finally, the use of MRA and SA in a multilevel fashion aims to identify factors contributing to excessive heterogeneity, thereby enhancing the overall credibility of the findings.

Despite the aforementioned strengths, the study is subject to several limitations associated with its computationally intensive methodology and estimator-based design. The primary constraint arises from the prevalence of non-randomized studies and the incorporation of relatively small-scale analyses. As detailed in the “Results” section, the aggregation of a substantial number of records characterized by heightened intra-study variation led to an also elevated degree of heterogeneity. Another limitation concerns the application of estimator functions and the assumption that the data in each study arm were normally distributed. This approach resulted in a relative shrinkage of individual standard errors, contributing to an increase in inter-study variation (*τ*^2^). While initially concerning, this observation may be inherent to the nature of the computations performed, within the analytical approach employed to obtain the Q- and MD_Q_-estimates [54]. Moreover, a third limitation emerges from the assessment of the Pearson’s correlation coefficient between the original variables (EBL, OT). Due to the absence of individual data for direct computation, the analysis employed Monte Carlo simulations to approximate r for each comparison arm of every included study. Regardless of this constraint, the results were considered consistent with the physiological underpinnings of the interconnection between the original variables. The above limitations underscore the need for cautious interpretation and emphasize the importance of considering these factors in the broader context of the research.

### Future potential

Accounting for its inherent limitations, this analysis significantly contributes to understanding the clinical relevance of the newfound Q variable in the context of RAPN and OPN comparisons. The discernible disparity observed between the two approaches, within the standard duration of each procedure, raise important considerations for future investigations. Therefore, it is suggested that future studies jointly elucidate summary statistics of EBL, OT, and Q for each compared patient population. Such an approach could enhance the determination of the correlation coefficient (r), optimizing the theoretical groundwork and strengthening the robustness of estimations for EV_Q_ and SE_Q_. Moreover, this study implies the potential utility of Q in predicting total blood loss in robotic and open PN, based on the estimated procedure duration. Achieving accuracy in this prediction could prove beneficial in clinical practice, facilitating a direct estimation of the impending blood loss volume. Future studies are encouraged to consider incorporating the Q-estimates derived, to formulate predictive models for EBL, with the aim of optimizing intraoperative transfusions in both RAPN and OPN. Furthermore, as previously discussed, a notable point of interest in this study concerns its synthetic methodology, which involves the retrograde formation of composite variables from retrospective original data. This approach may prove particularly valuable as a feasibility analysis, offering prior distributions in the Bayesian framework for the prospective design of randomized controlled trials (RCTs), with the goal of enhancing statistical power and reducing the costs linked to the incorporation of multiple outcomes [55, 56]. Having meticulously recognized that the Q quotient may not conclusively model the multifaceted concept of tissue handling delicacy in PN, in a recent study we specifically explored the precision aspect related to the proportion of ischemia [57]. Consequently, we advocate for additional studies in this field to investigate surgical precision within the common methodological framework established, potentially integrating conventional data from volumetric analyses.

## Conclusions

In the current study, we systematically compared RAPN and OPN, placing particular emphasis on per-minute blood loss by leveraging available literature data. In this context, we introduced a novel variable denoted as Q, representing the ratio between the average blood loss volume (EBL) and the mean procedural duration (OT). This metric was considered inversely associated with the level of surgical precision in PN. Utilizing summary statistics from original comparative outcomes (EBL and OT), we captured the required statistical parameters to approximate their quotient (Q) through statistical estimation. Subsequently, we employed an appropriate meta-analytical methodology to compute the overall mean difference between the two approaches. The data analysis revealed a statistically significant advantage for RAPN in maintaining lower Q-estimates. The examination of effects from study characteristics demonstrated consistent homogeneity in terms of statistical inference, suggesting that the observed advantage on the part of RAPN may be inherent. The subsequent sensitivity analysis indicated a trend of widening in the comparative advantage offered by the robotic approach, especially concerning the skill level of the treating surgeon. However, further studies will be necessary to explore additional aspects of this comparison, and to determine Q within the framework of a prospective analysis protocol.

## Supplementary Information

Below is the link to the electronic supplementary material.

Supplementary file1 (DOCX 47266 KB)

Supplementary file2 (DOCX 6396 KB)

Supplementary file3 (DOCX 459 KB)

Supplementary file4 (DOCX 4983 KB)

## Data Availability

All the data utilized and statistical code developed are available on GitHub at the following link: https://github.com/sotbike/Q.git.

## Abbreviations

AKI: Acute kidney injury
CCI: Comprehensive complication index
CENTRAL: Cochrane Central Register of Controlled Trials
CI_95%_: 95% Confidence interval
CKD: Chronic kidney disease
cov: Covariance
EBL^*^: Predicted EBL based on Q in an average duration robotic or open PN
EBL: Estimated blood loss
eGFR: Estimated glomerular filtration rate
EV: Expected value
GFR: Glomerular filtration rate
H–K: Hartung and Knapp (adjustment)
IQR: Interquartile range
IT: Ischemia time
LOS: Length of (Hospital) stay
LPN: Laparoscopic partial nephrectomy
MA: Meta-analysis
MB: Magnitude of (publication) bias
MD: Mean difference
MIC: Margin, ischemia, complications
min: Minutes
ml: Milliliters
MRA: Meta-regression analysis
NOS: Newcastle–Ottawa scale
OPN: Open partial nephrectomy
OT: Operative time
OT_std_: Normalized (average) OT between RAPN and OPN
PB: Publication bias
PBRC: Packed red blood cells
PN: Partial nephrectomy
PRISMA: Preferred reporting items for systematic reviews and meta-analyses
PSM: Positive surgical margins
Q: Per minute blood loss (Q = EBL / OT)
r: Pearson’s correlation coefficient (between EBL & OT)
RAPN: Robot-assisted partial nephrectomy
RCT: Randomized controlled trial
REML: Restricted maximum likelihood
ROB: Risk of bias
ROBINS-I: Risk of bias in non-randomized studies of interventions
RPN: Robotic partial nephrectomy
SA: Sensitivity analysis
SD: Standard deviation
SE: Standard error
SGA: Subgroup analysis
SSEs: Small-study effects
URL: Uniform resource locator
WB: Whole blood
WIT: Warm ischemia time
