# Supplementary material for: Comparing robotic and open partial nephrectomy under the prism of surgical precision: a meta-analysis of the average blood loss rate as a novel variable
Source: J Robot Surg. 2024 Aug 7;18(1):313. doi: 10.1007/s11701-024-02060-z (PMC11306375; doi:10.1007/s11701-024-02060-z)
Supplement: Supplementary file 1 — Supplementary file1 (DOCX 47266 KB) [file 11701_2024_2060_MOESM1_ESM.docx]

SUPPLEMENTARY ANIMATED PLOTS

Supplementary Animated Plot 1: Animated funnel plot with an embedded curved regression line, to assess the significance of small study effects, for successive r-values from -0.99 to +0.99. (Double-click on the plot to play & press ESC to exit)

Supplementary Animated Plot 2: Animated radial plot with an integrated solid regression line, to assess the significance of publication bias through the Egger's test, for successive r-values from -0.99 to +0.99. (Double-click on the plot to play & press ESC to exit)

Supplementary Animated Plot 3: Animated meta-regression plot showing the change in the comparative effect (MD_Q_) between RPN/RAPN vs. OPN along with the CI_95%_, in pooled studies, using as moderator the publication year, for successive r-values from -0.99 to +0.99. (Double-click on the plot to play & press ESC to exit)

Supplementary Animated Plot 4: Animated meta-regression plot showing the change in the comparative effect (MD_Q_) between RPN/RAPN vs. OPN along with the CI_95%_, in studies with patient matching, using as moderator the publication year, for successive r-values from -0.99 to +0.99. (Double-click on the plot to play & press ESC to exit)

Supplementary Animated Plot 5: Animated meta-regression plot showing the change in the comparative effect (MD_Q_) between RPN/RAPN vs. OPN along with the CI_95%_, in studies without patient matching, using as moderator the publication year, for successive r-values from -0.99 to +0.99. (Double-click on the plot to play & press ESC to exit)

Supplementary Animated Plot 6: Animated meta-regression plot showing the change in the comparative effect (MD_Q_) between RPN/RAPN vs. OPN along with the CI_95%_, in multicenter studies, using as moderator the publication year, for successive r-values from -0.99 to +0.99. (Double-click on the plot to play & press ESC to exit)

Supplementary Animated Plot 7: Animated meta-regression plot showing the change in the comparative effect (MD_Q_) between RPN/RAPN vs. OPN along with the CI_95%_, in single-center studies, using as moderator the publication year, for successive r-values from -0.99 to +0.99. (Double-click on the plot to play & press ESC to exit)

Supplementary Animated Plot 8: Animated meta-regression plot showing the change in the comparative effect (MD_Q_) between RPN/RAPN vs. OPN along with the CI_95%_, in studies with ROBINS-I: Low, using as moderator the publication year, for successive r-values from -0.99 to +0.99. (Double-click on the plot to play & press ESC to exit)

Supplementary Animated Plot 9: Animated meta-regression plot showing the change in the comparative effect (MD_Q_) between RPN/RAPN vs. OPN along with the CI_95%_, in studies with ROBINS-I: Moderate, using as moderator the publication year, for successive r-values from -0.99 to +0.99. (Double-click on the plot to play & press ESC to exit)

Supplementary Animated Plot 10: Animated meta-regression plot showing the change in the comparative effect (MD_Q_) between RPN/RAPN vs. OPN along with the CI_95%_, in studies with ROBINS-I: Serious, using as moderator the publication year, for successive r-values from -0.99 to +0.99. (Double-click on the plot to play & press ESC to exit)

Supplementary Animated Plot 11: Animated meta-regression plot showing the change in the comparative effect (MD_Q_) between RPN/RAPN vs. OPN along with the CI_95%_, in pooled studies, using as moderator the number of NOS quality stars, for successive r-values from -0.99 to +0.99. (Double-click on the plot to play & press ESC to exit)

Supplementary Animated Plot 12: Animated meta-regression plot showing the change in the comparative effect (MD_Q_) between RPN/RAPN vs. OPN along with the CI_95%_, in studies with patient matching, using as moderator the number of NOS quality stars, for successive r-values from -0.99 to +0.99. (Double-click on the plot to play & press ESC to exit)

Supplementary Animated Plot 13: Animated meta-regression plot showing the change in the comparative effect (MD_Q_) between RPN/RAPN vs. OPN along with the CI_95%_, in studies without patient matching, using as moderator the number of NOS quality stars, for successive r-values from -0.99 to +0.99. (Double-click on the plot to play & press ESC to exit)

Supplementary Animated Plot 14: Animated meta-regression plot showing the change in the comparative effect (MD_Q_) between RPN/RAPN vs. OPN along with the CI_95%_, in multicenter studies, using as moderator the number of NOS quality stars, for successive r-values from -0.99 to +0.99. (Double-click on the plot to play & press ESC to exit)

Supplementary Animated Plot 15: Animated meta-regression plot showing the change in the comparative effect (MD_Q_) between RPN/RAPN vs. OPN along with the CI_95%_, in single-center studies, using as moderator the number of NOS quality stars, for successive r-values from -0.99 to +0.99. (Double-click on the plot to play & press ESC to exit)
