# Supplementary material for: Comparing robotic and open partial nephrectomy under the prism of surgical precision: a meta-analysis of the average blood loss rate as a novel variable
Source: J Robot Surg. 2024 Aug 7;18(1):313. doi: 10.1007/s11701-024-02060-z (PMC11306375; doi:10.1007/s11701-024-02060-z)
Supplement: Supplementary file 2 — Supplementary file2 (DOCX 6396 KB) [file 11701_2024_2060_MOESM2_ESM.docx]

SUPPLEMENTARY FIGURES


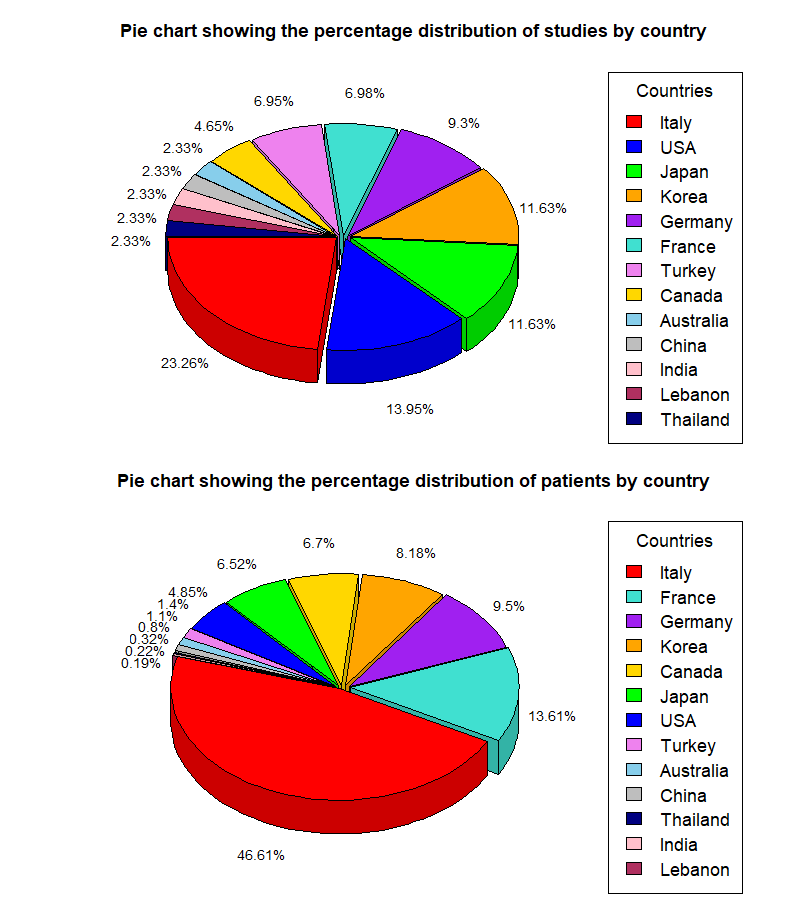


Supplementary Figure 1: Pie charts displaying the percentage distributions of studies and patients by their respective countries of origin.


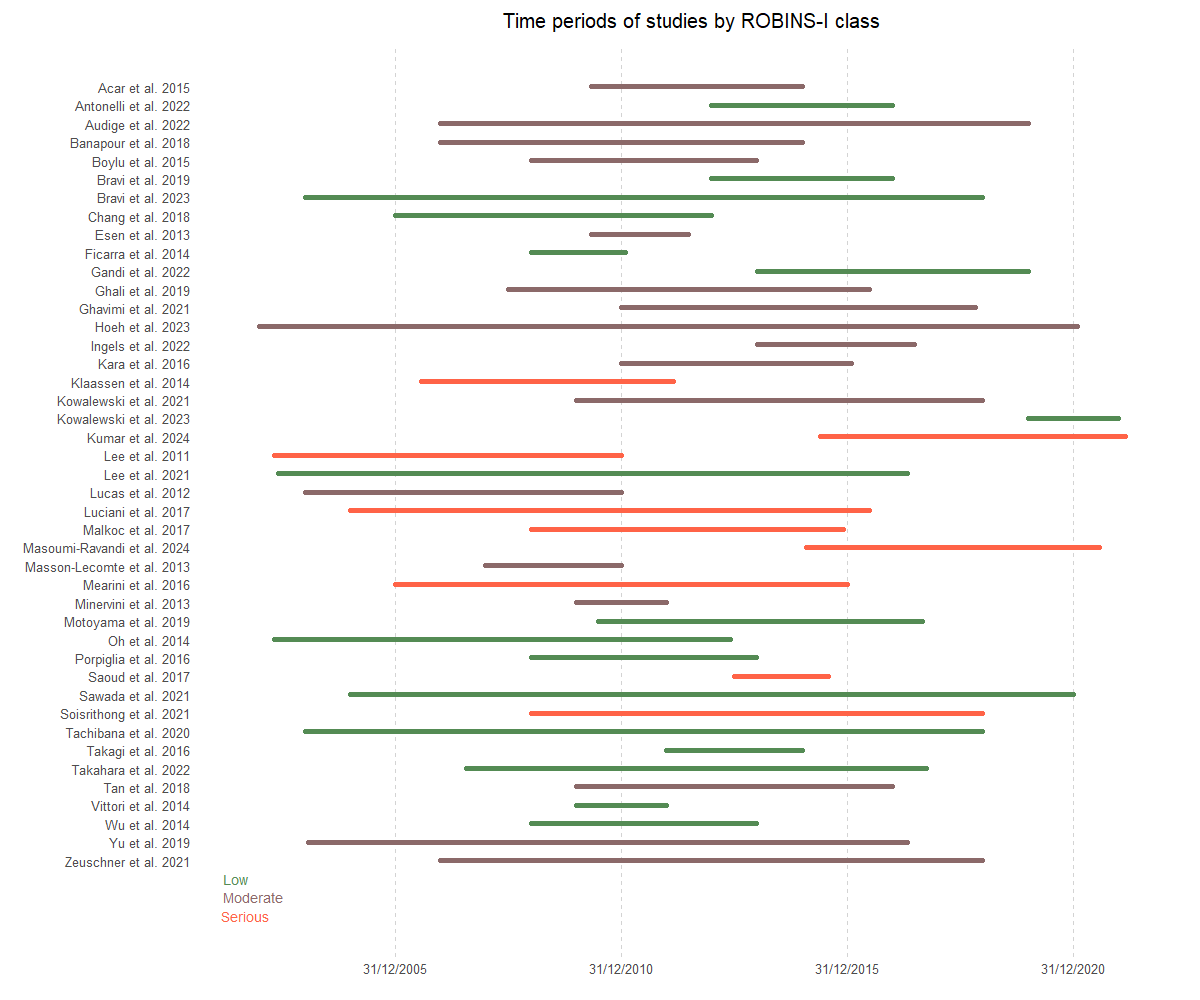


Supplementary Figure 2: Diagram illustrating the temporal activity intervals of the included studies by ROBINS-I class.


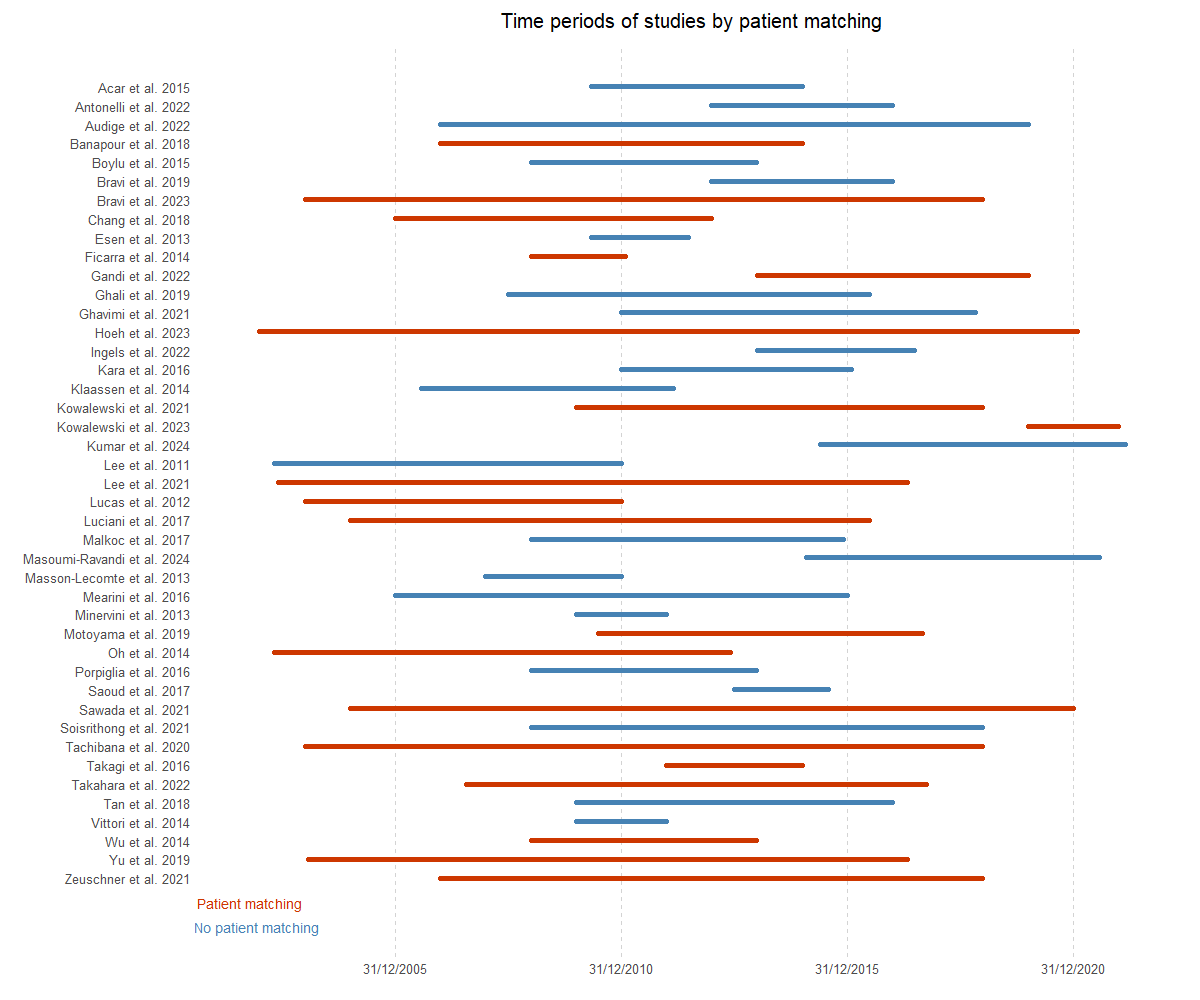


Supplementary Figure 3: Diagram illustrating the temporal activity intervals of the included studies by patient matching.


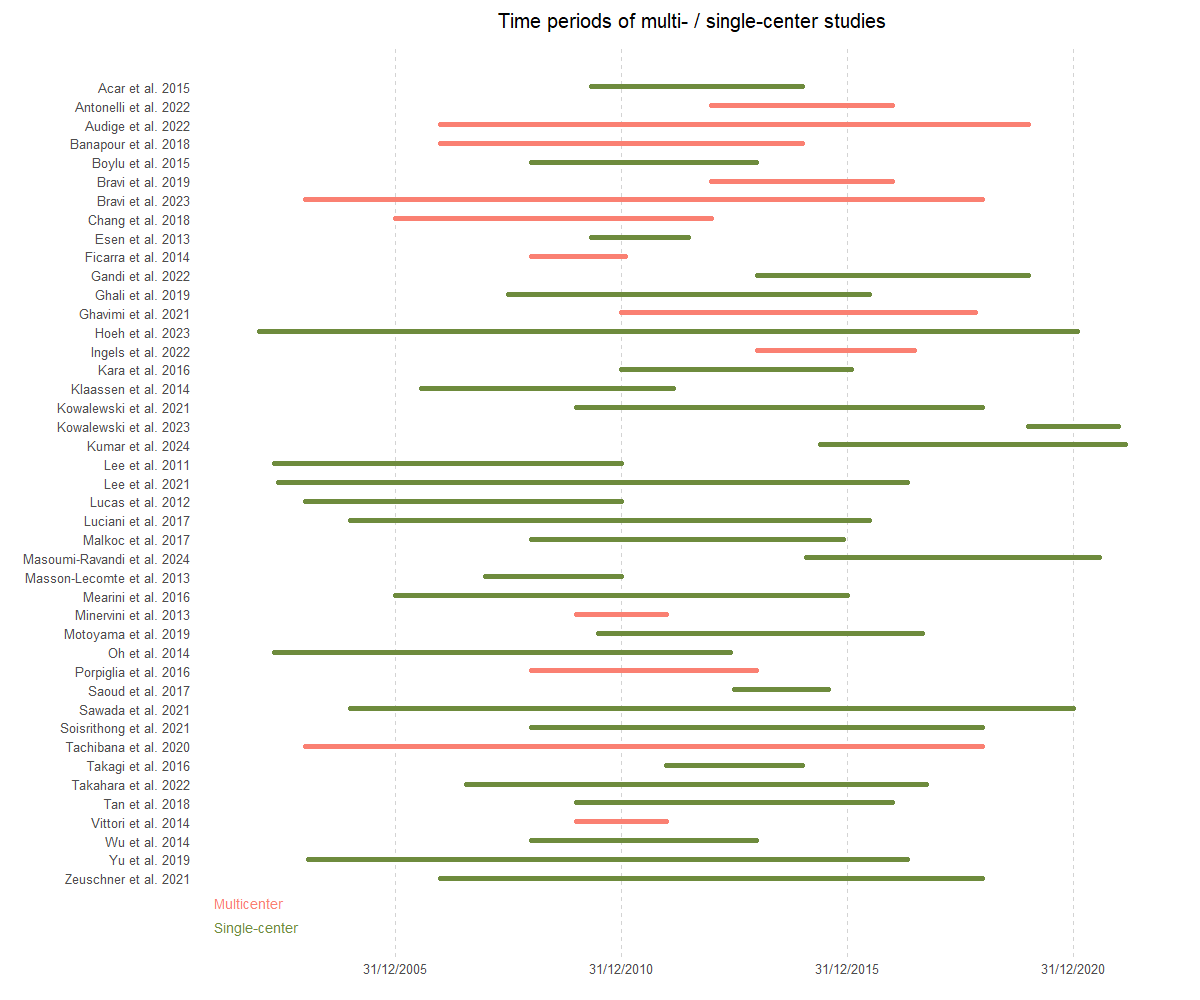


Supplementary Figure 4: Diagram illustrating the temporal activity intervals of the included studies by the number of referral centers.


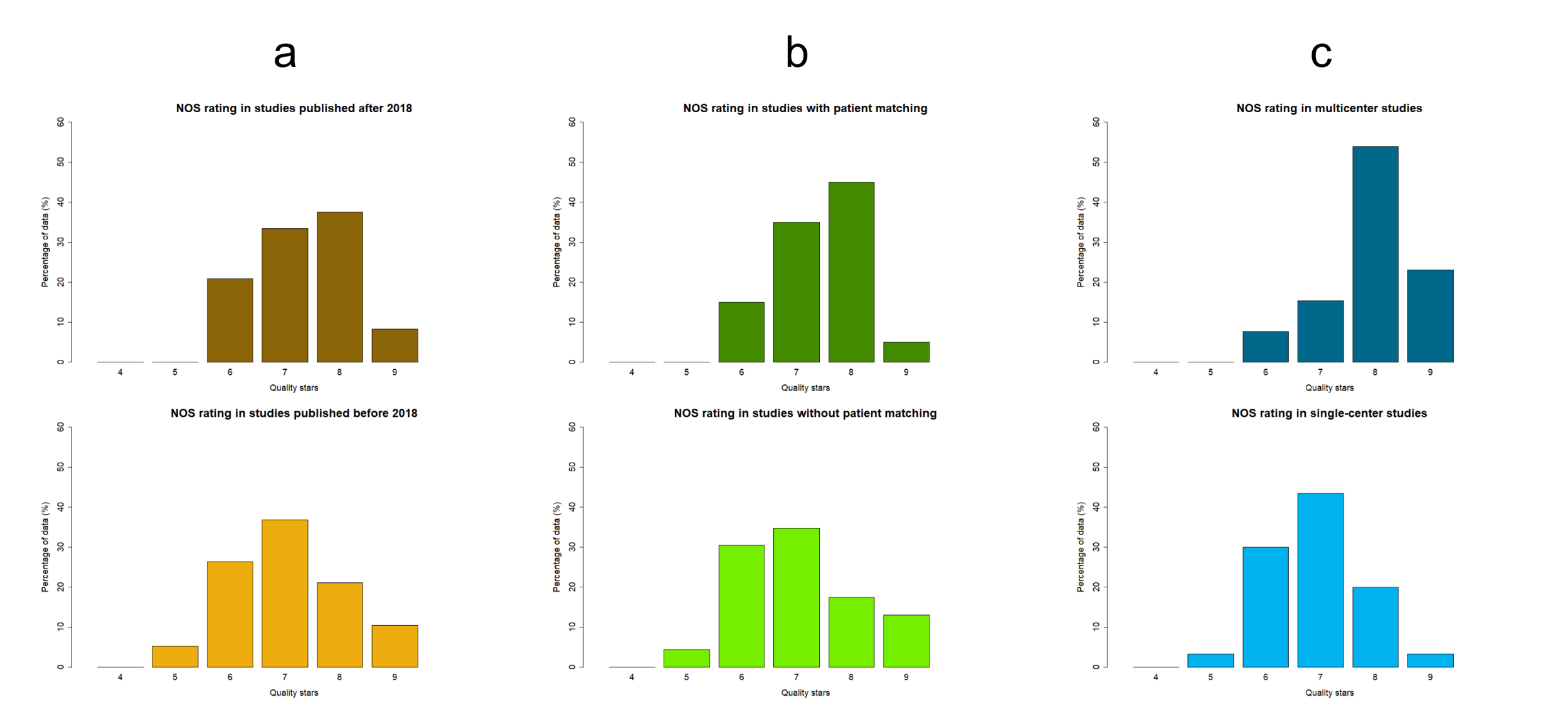


Supplementary Figure 5: Histograms illustrating the respective percentages for each quality level based on the Newcastle - Ottawa Scale (NOS), across the total of studies included in each subgroup, with respect to the year of publication (a), patient matching implementation (b), and the number of referral centers involved (c).


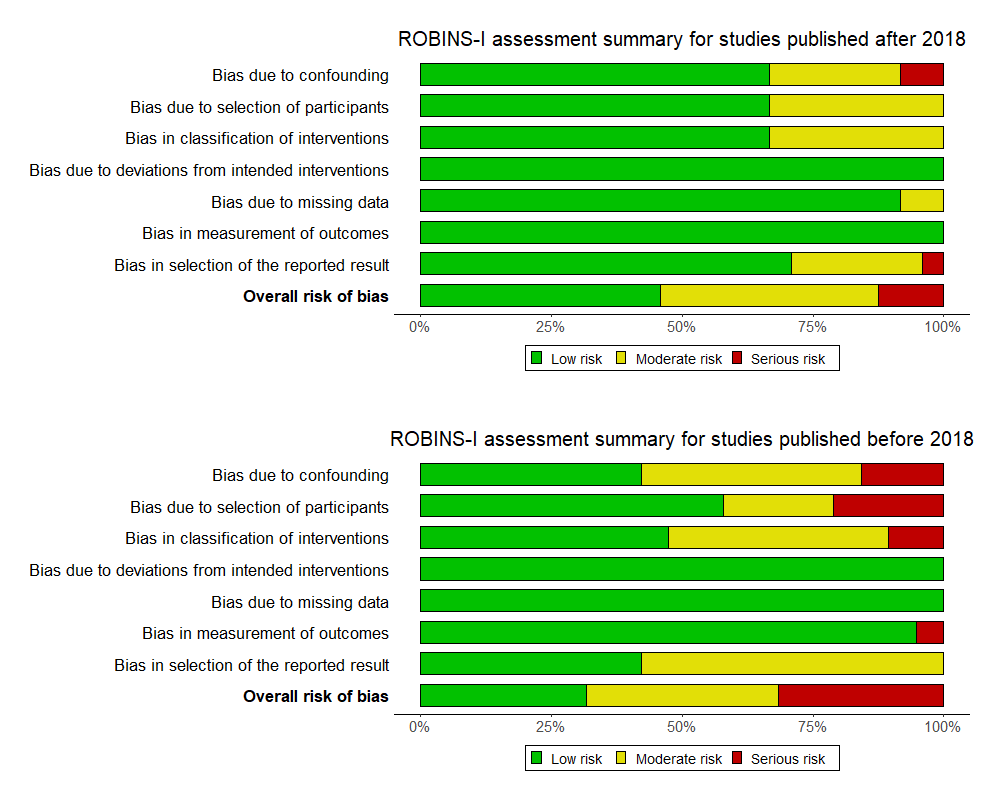


Supplementary Figure 6: Summary plot illustrating the evaluation of subgroups by publication year, using the ROBINS-I tool, presenting their percentages stratified by the risk level within each of the seven domains.

*
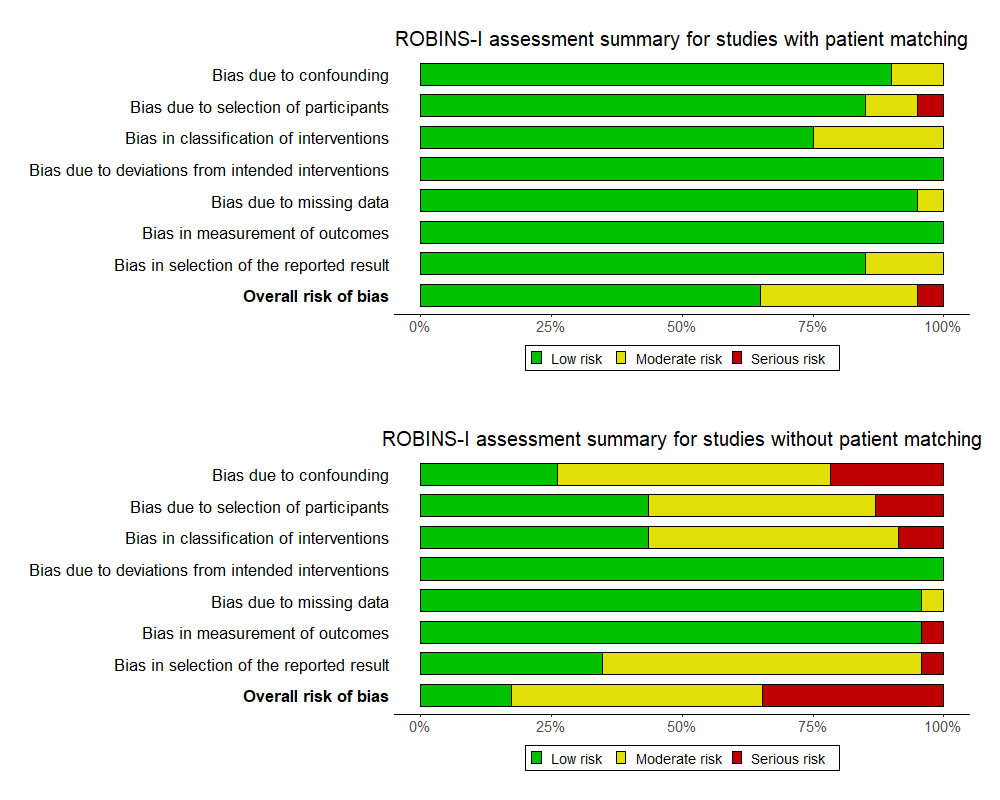
*

Supplementary Figure 7: Summary plot illustrating the evaluation of subgroups by patient matching, using the ROBINS-I tool, presenting their percentages stratified by the risk level within each of the seven domains.


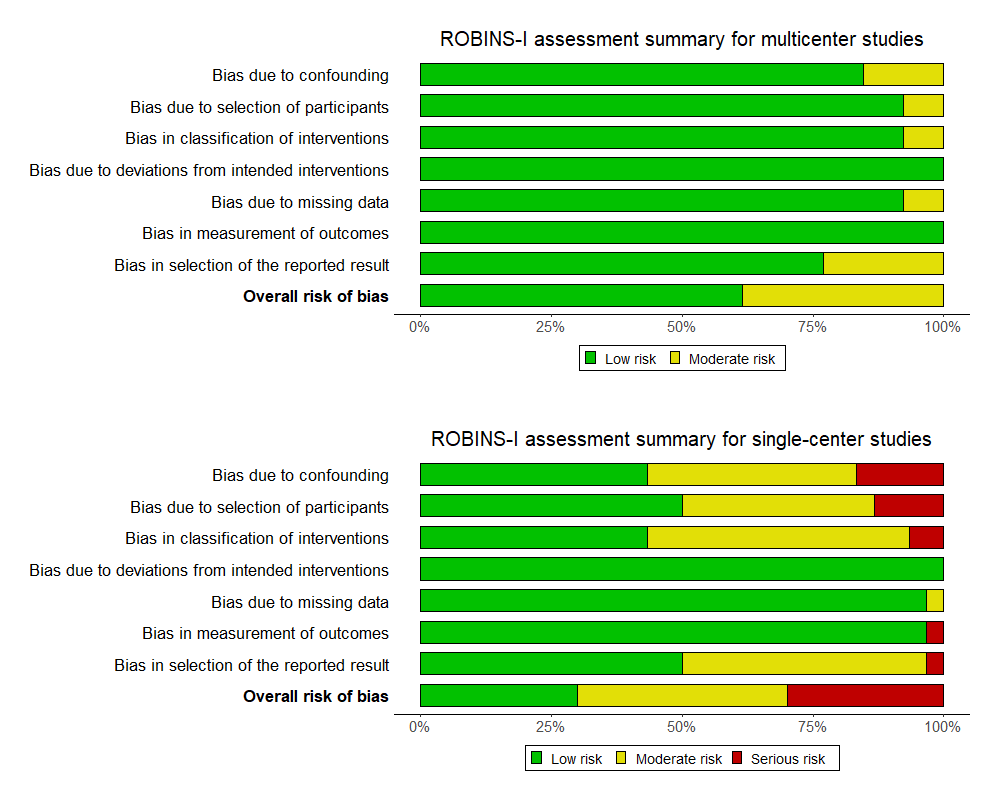


Supplementary Figure 8: Summary plot illustrating the evaluation of subgroups by the number of referral centers involved, using the ROBINS-I tool, presenting their percentages stratified by the risk level within each of the seven domains.


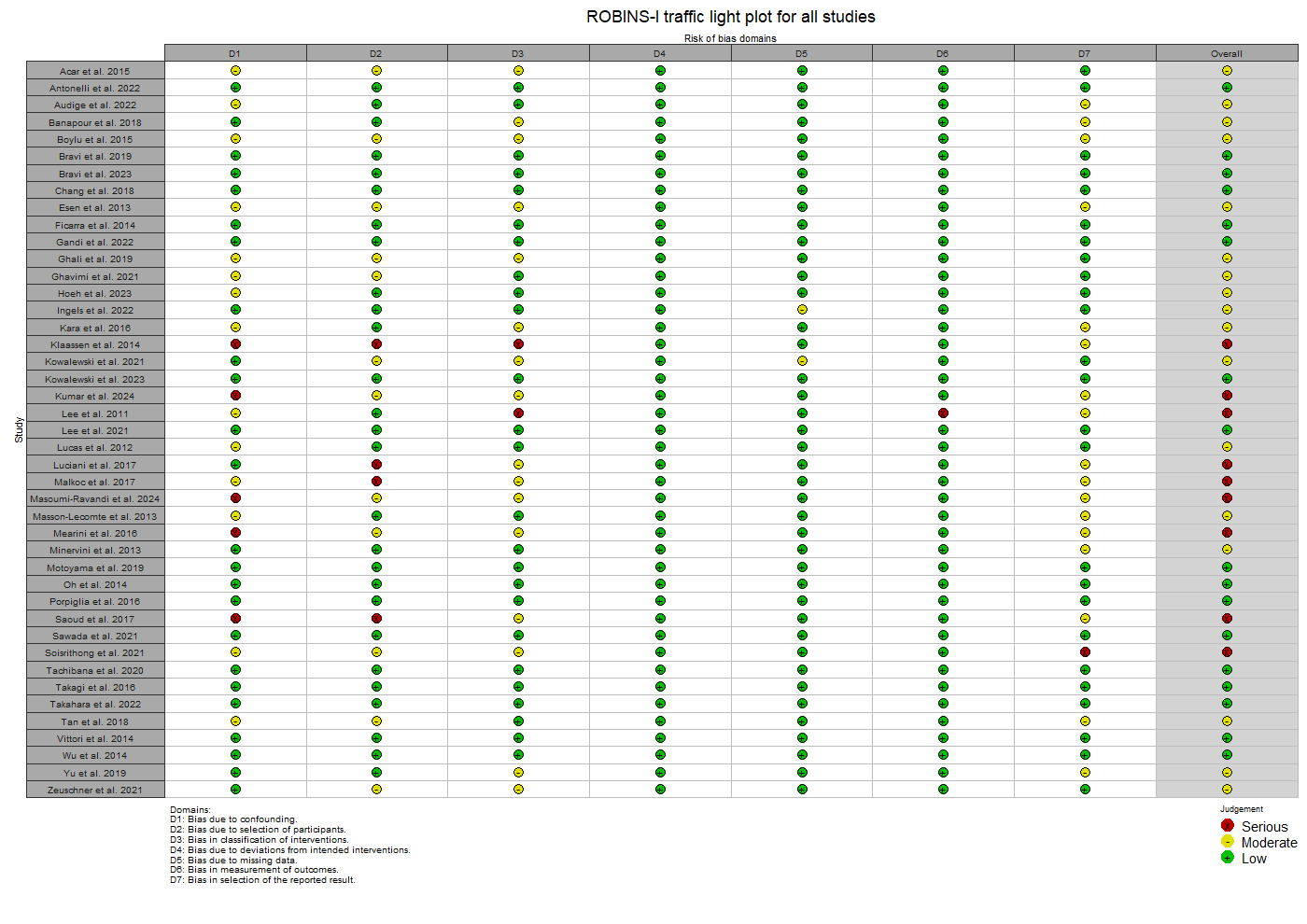


Supplementary Figure 9: Traffic light plot illustrating the evaluation of all included studies, using the ROBINS-I tool, presenting their risk level within each of the seven domains.


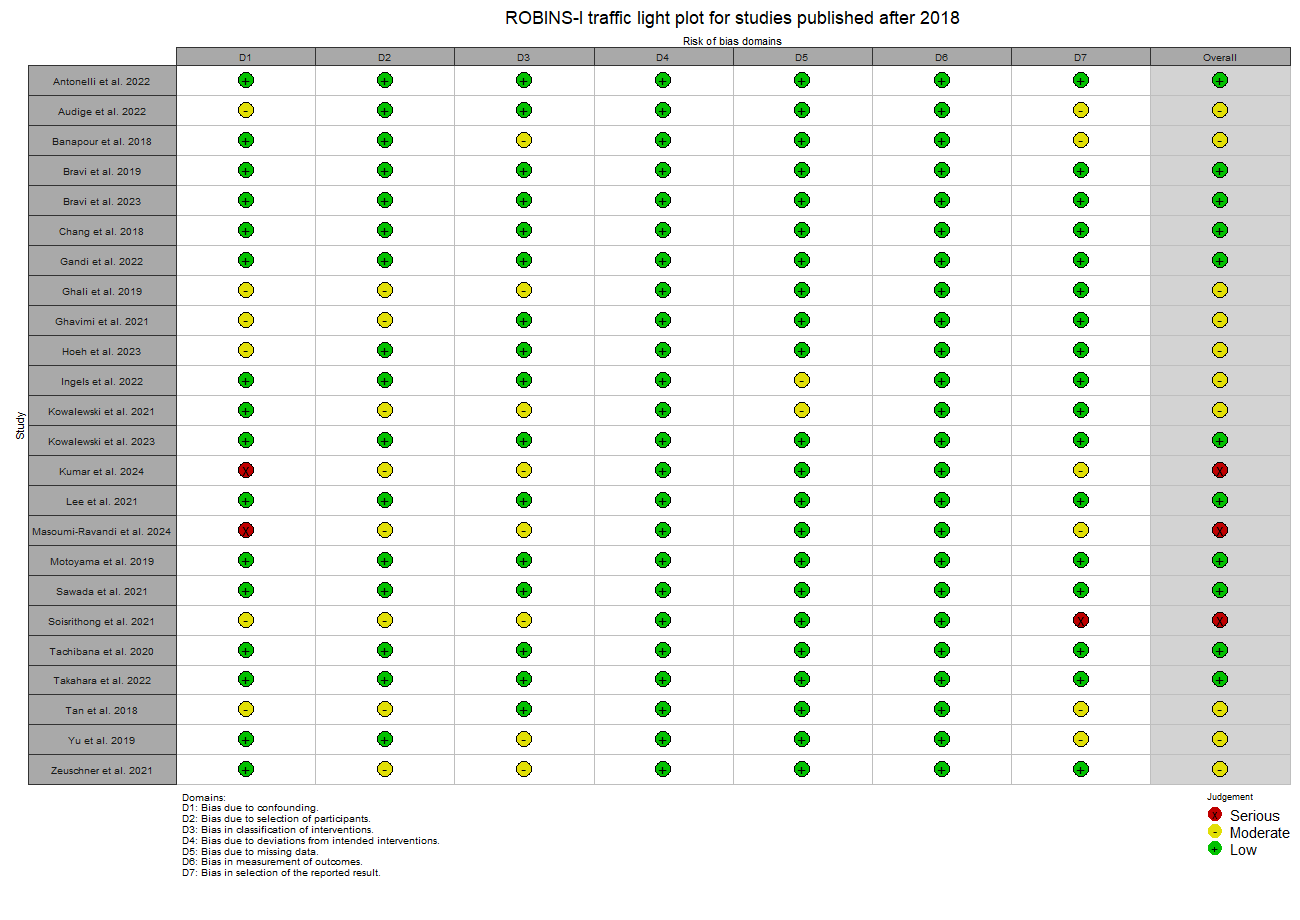


Supplementary Figure 10: Traffic light plot illustrating the evaluation of studies published after 2018, using the ROBINS-I tool, presenting their risk level within each of the seven domains.


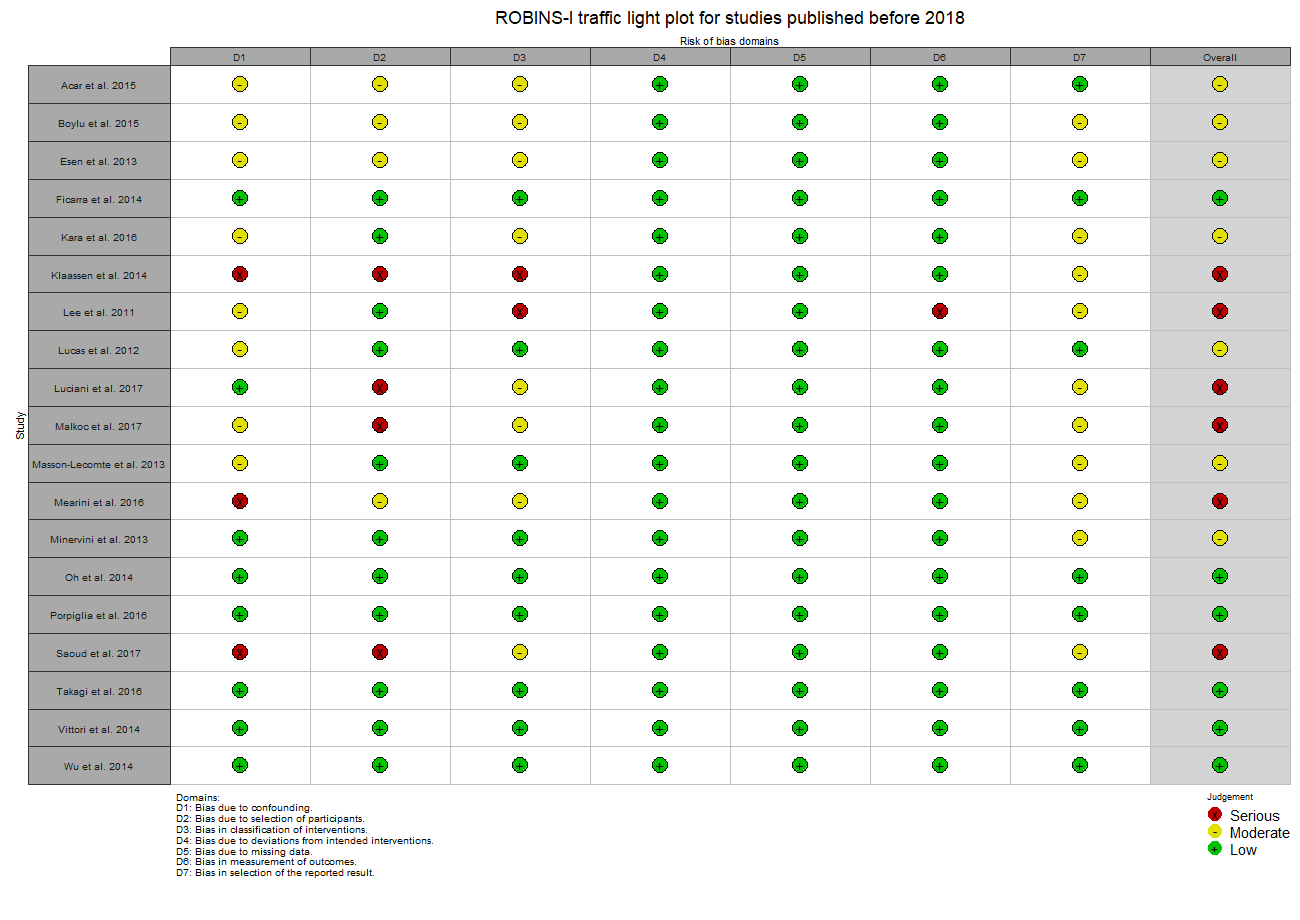


Supplementary Figure 11: Traffic light plot illustrating the evaluation of studies published before 2018, using the ROBINS-I tool, presenting their risk level within each of the seven domains.


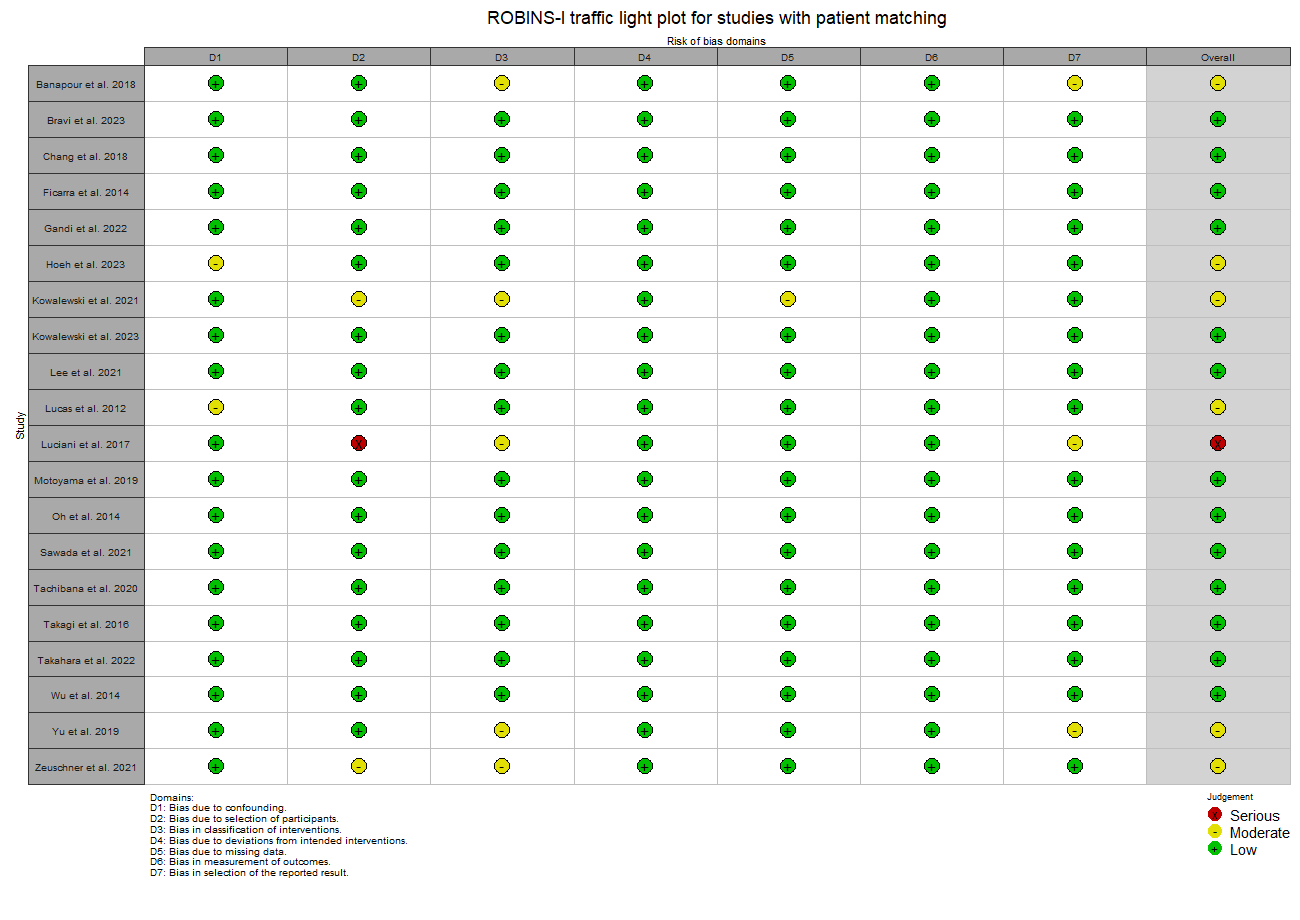


Supplementary Figure 12: Traffic light plot illustrating the evaluation of studies with patient matching, using the ROBINS-I tool, presenting their risk level within each of the seven domains.


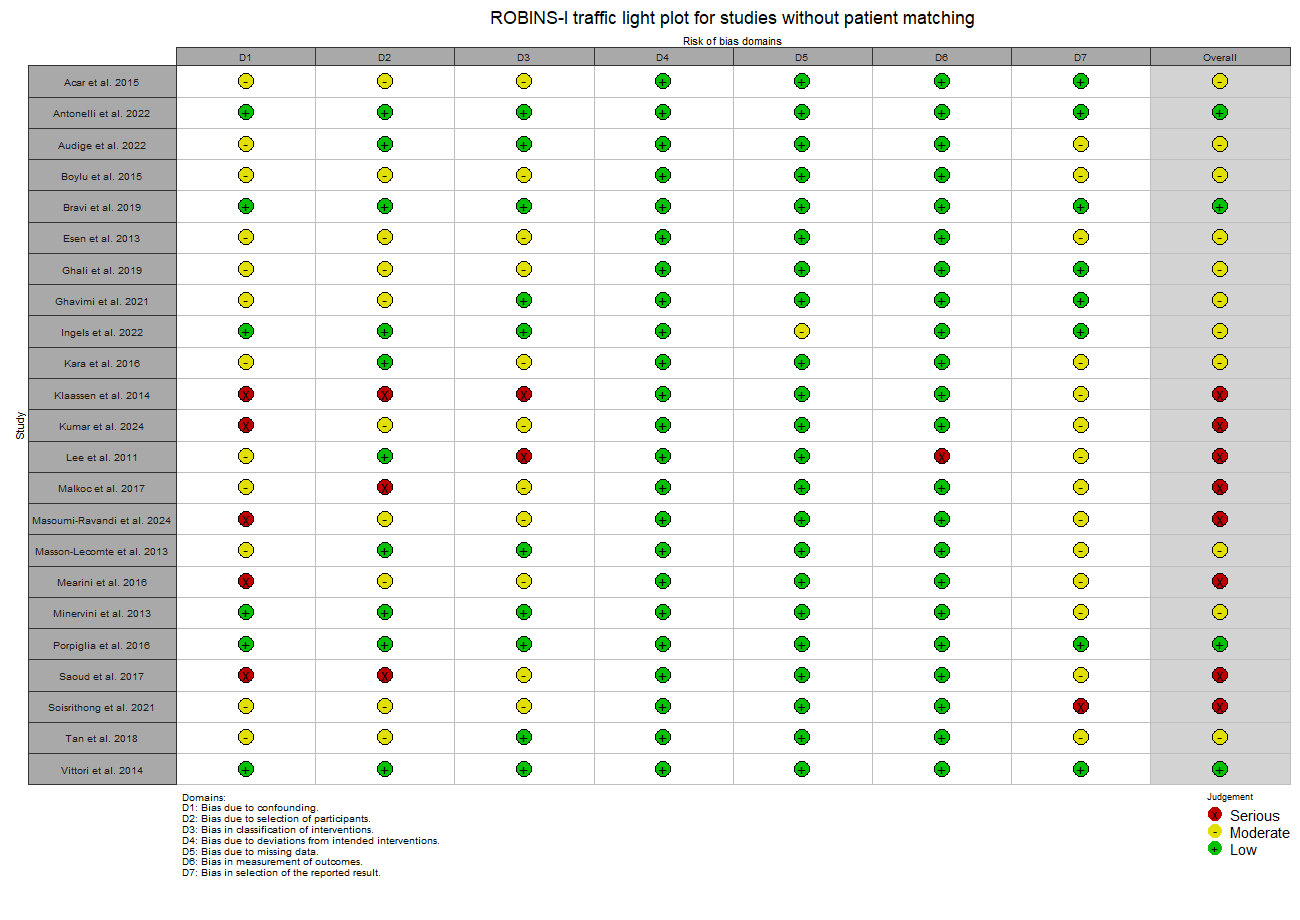


Supplementary Figure 13: Traffic light plot illustrating the evaluation of studies without patient matching, using the ROBINS-I tool, presenting their risk level within each of the seven domains.


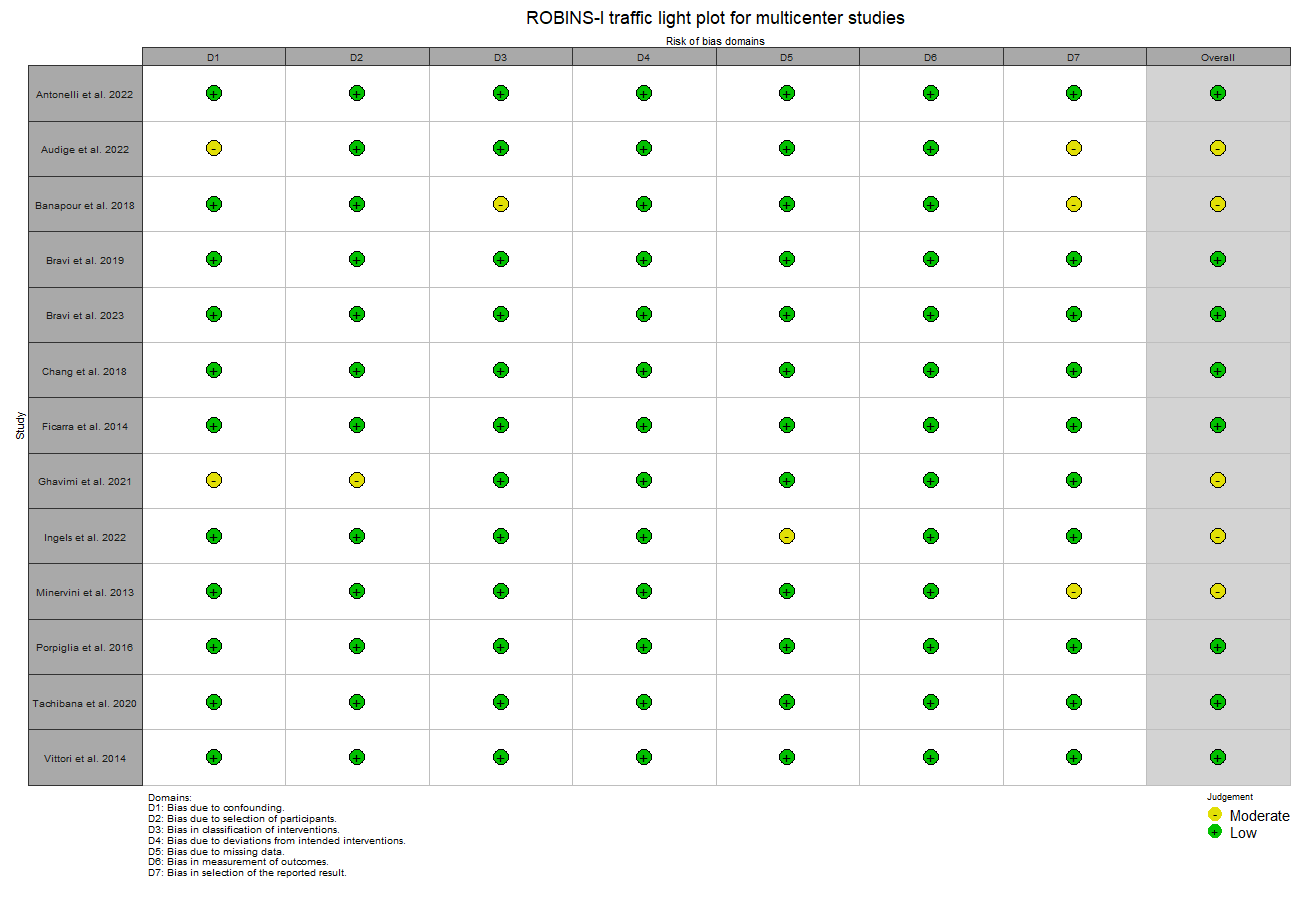


Supplementary Figure 14: Traffic light plot illustrating the evaluation of multicenter studies, using the ROBINS-I tool, presenting their risk level within each of the seven domains.


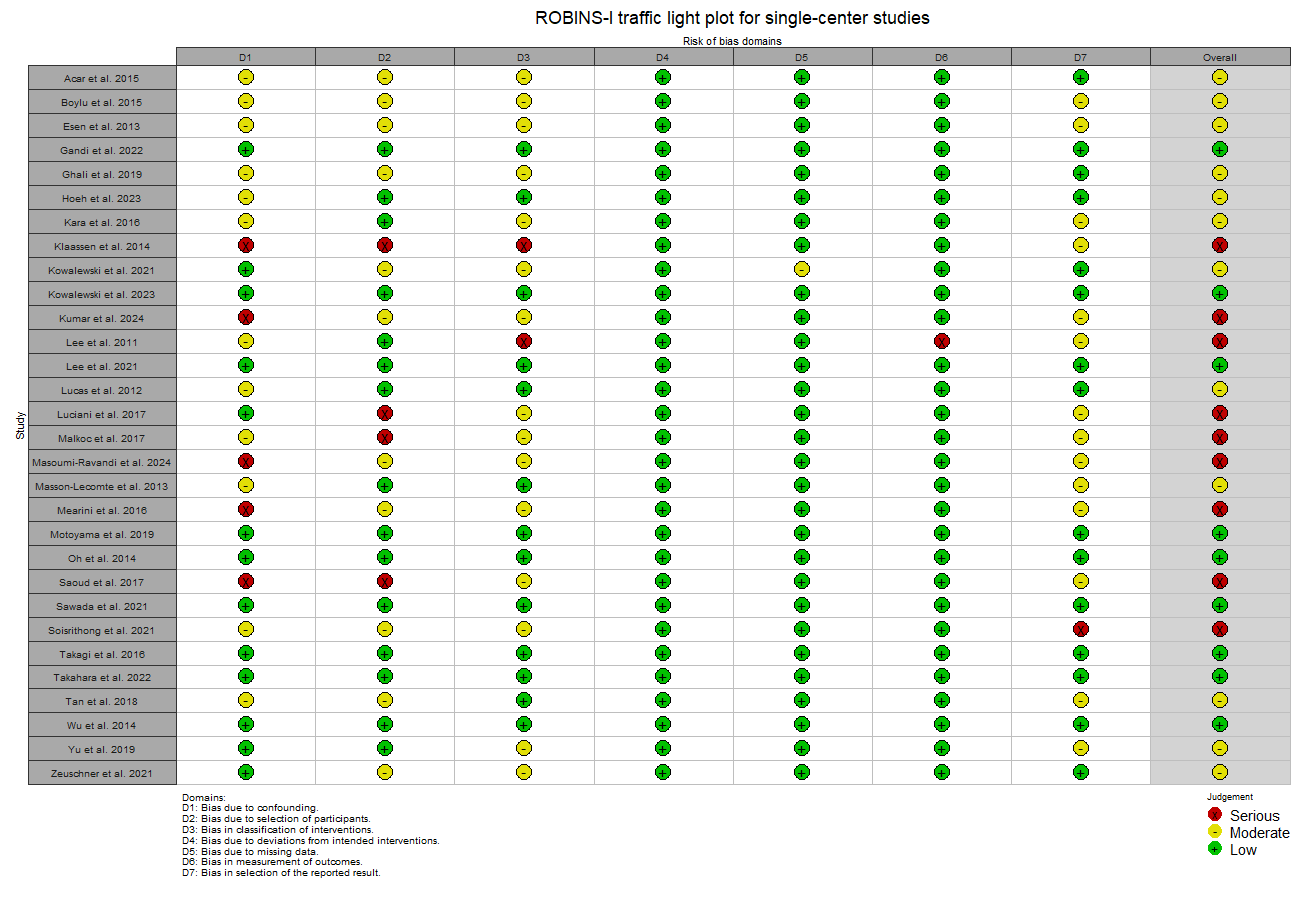


Supplementary Figure 15: Traffic light plot illustrating the evaluation of single-center studies, using the ROBINS-I tool, presenting their risk level within each of the seven domains.


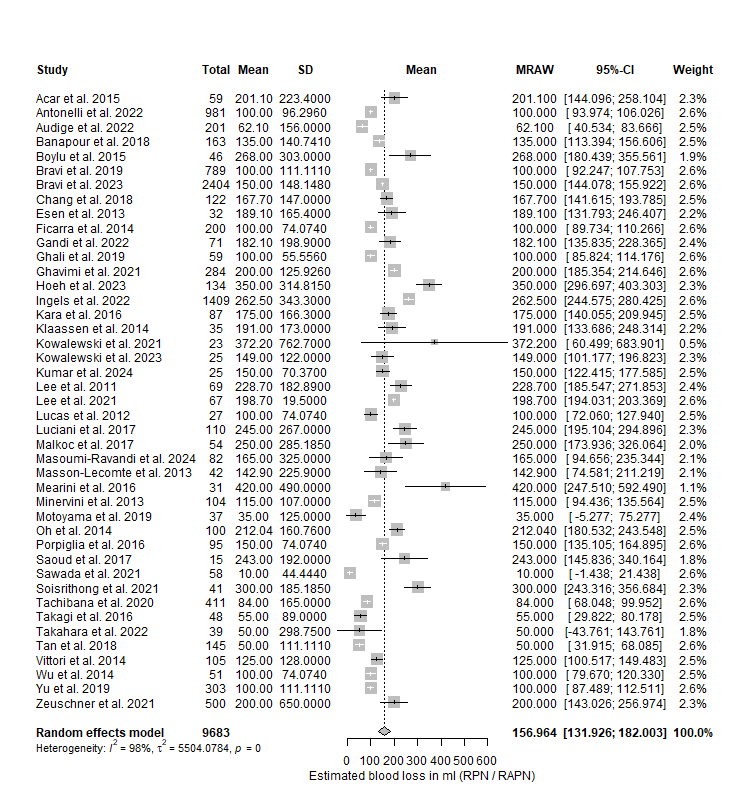


*
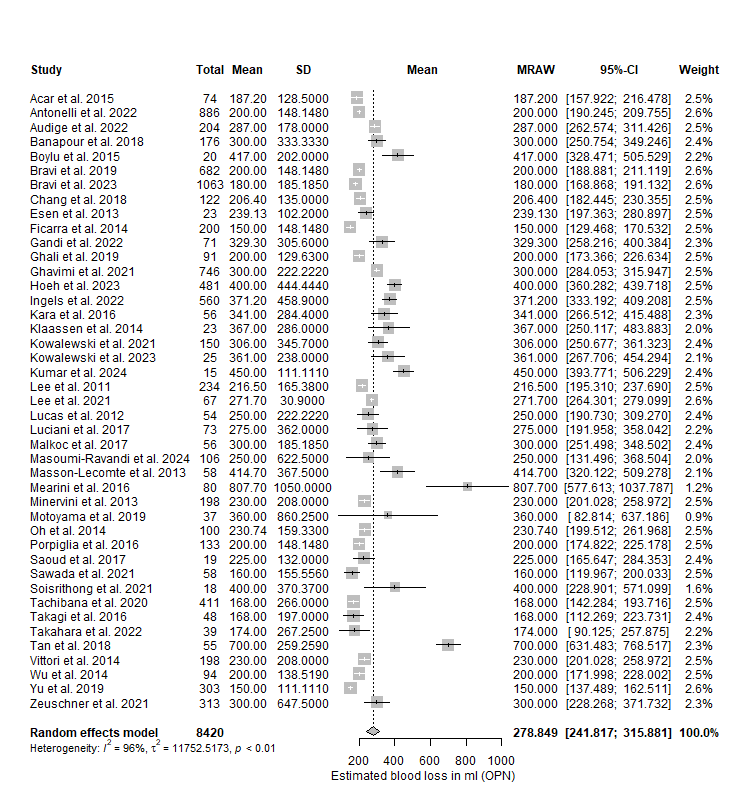

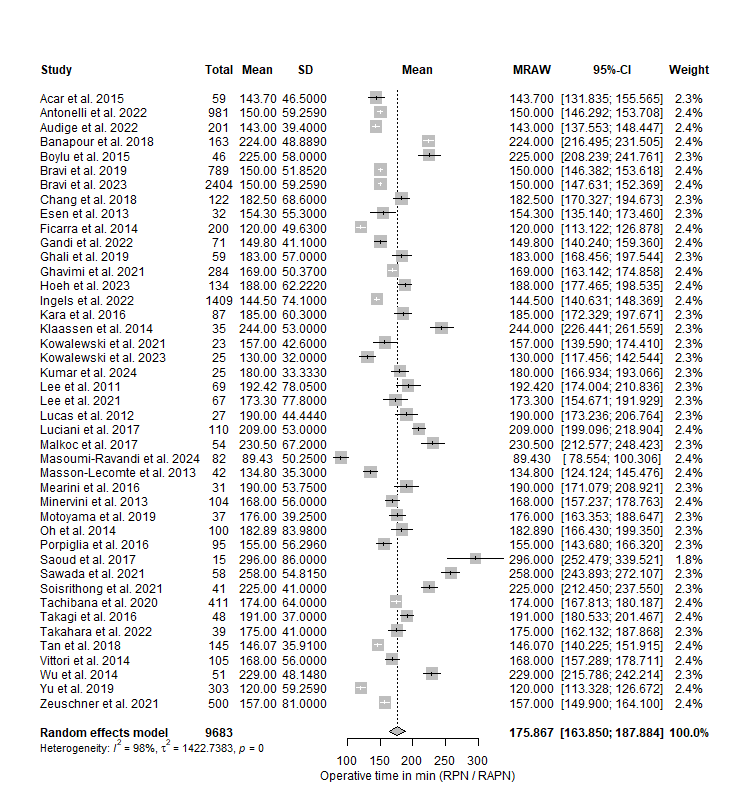

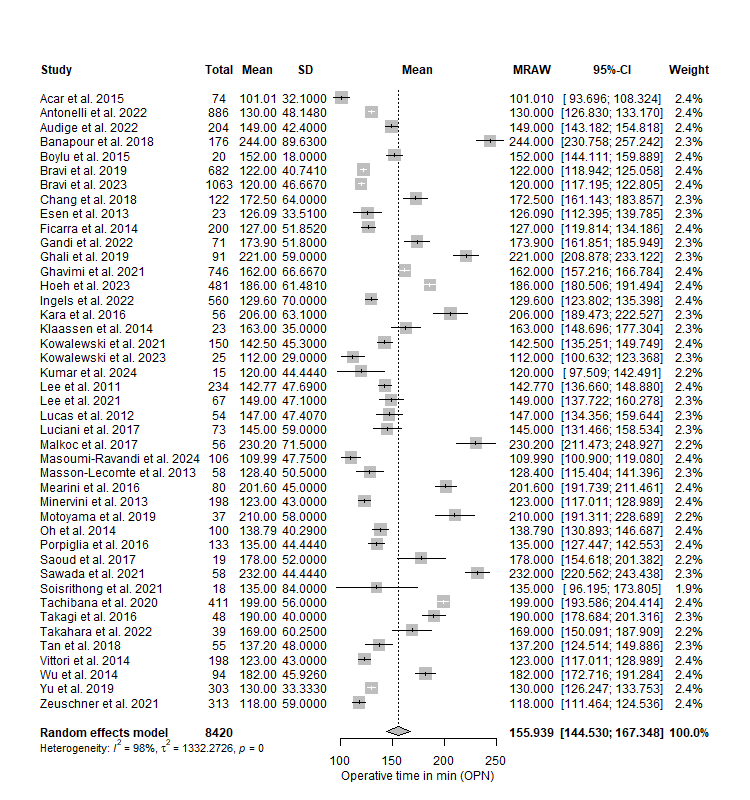

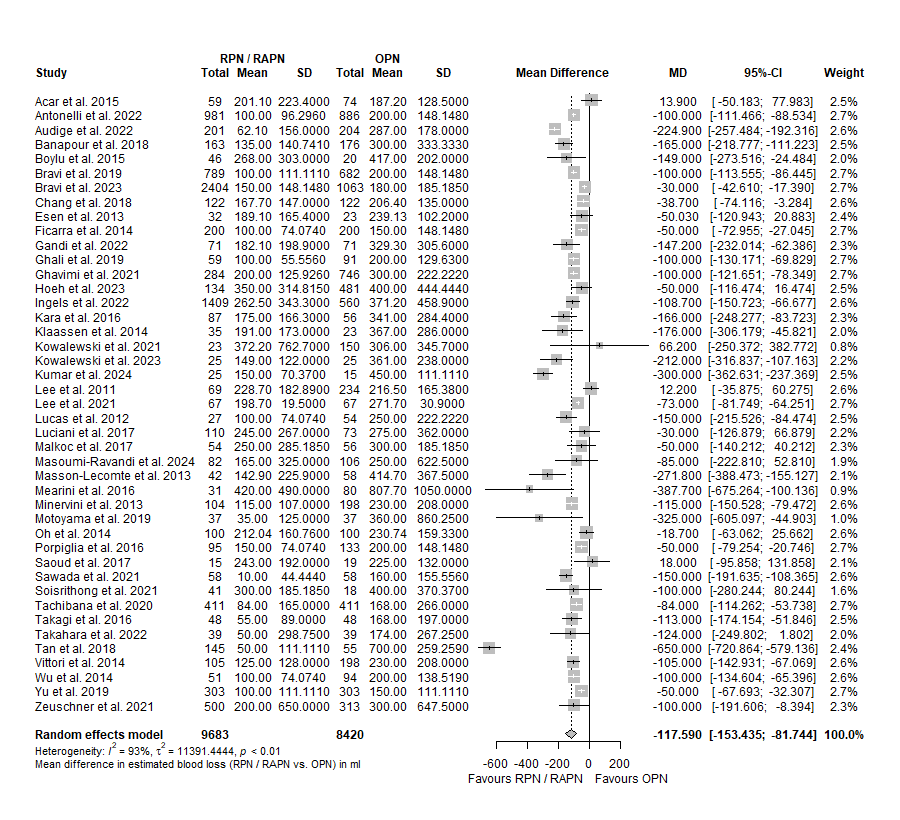

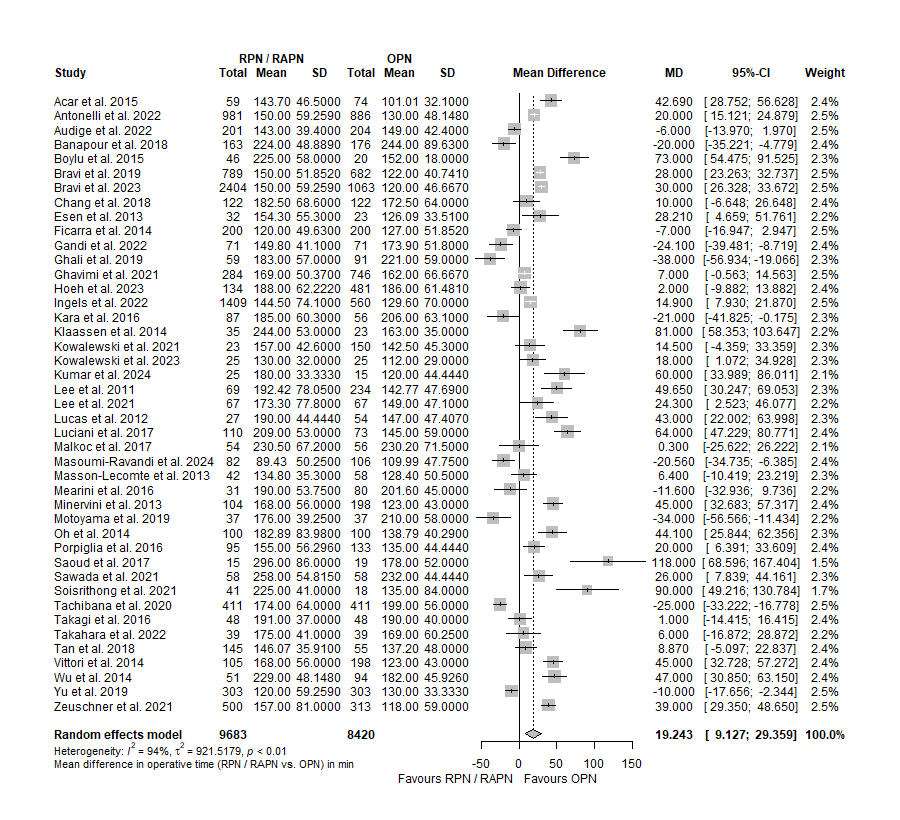
*

Supplementary Figure 16: Forest plot showing the estimation of the expected value of EBL (EV_EBL_) along with its CI_95%_ in RPN / RAPN group, for all included studies.

Supplementary Figure 17: Forest plot showing the estimation of the expected value of EBL (EV_EBL_) along with its CI_95%_ in OPN group, for all included studies.

Supplementary Figure 18: Forest plot showing the estimation of the expected value of OT (EV_OT_) along with its CI_95%_ in RPN / RAPN group, for all included studies.

Supplementary Figure 19: Forest plot showing the estimation of the expected value of OT (EV_OT_) along with its CI_95%_ in OPN group, for all included studies.

Supplementary Figure 20: Forest plot showing the estimation of the mean difference in EBL (MD_EBL_) along with its CI_95%_ in RPN/RAPN vs. OPN, for all included studies.

Supplementary Figure 21: Forest plot showing the estimation of the mean difference in OT (MD_OT_) along with its CI_95%_ in RPN/RAPN vs. OPN, for all included studies.


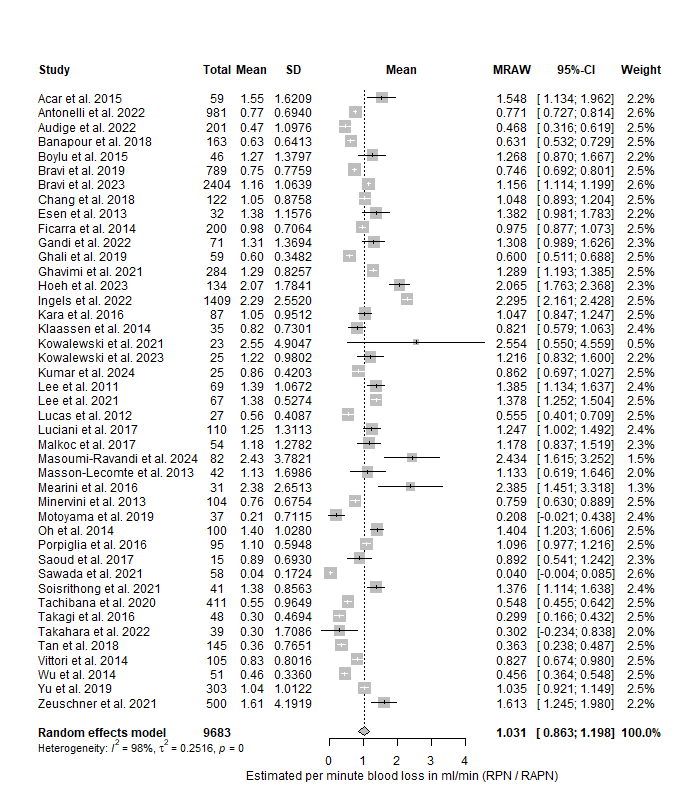


Supplementary Figure 22: Forest plot showing the estimation of the expected value of Q (EV_Q_) along with its CI_95%_ in RPN / RAPN group, for all included studies.


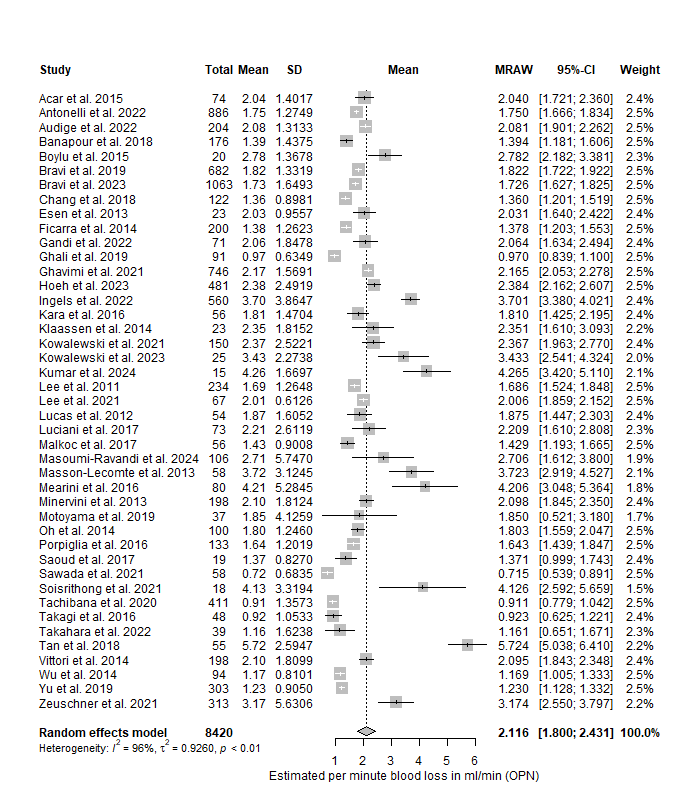


Supplementary Figure 23: Forest plot showing the estimation of the expected value of Q (EV_Q_) along with its CI_95%_ in OPN group, for all included studies.


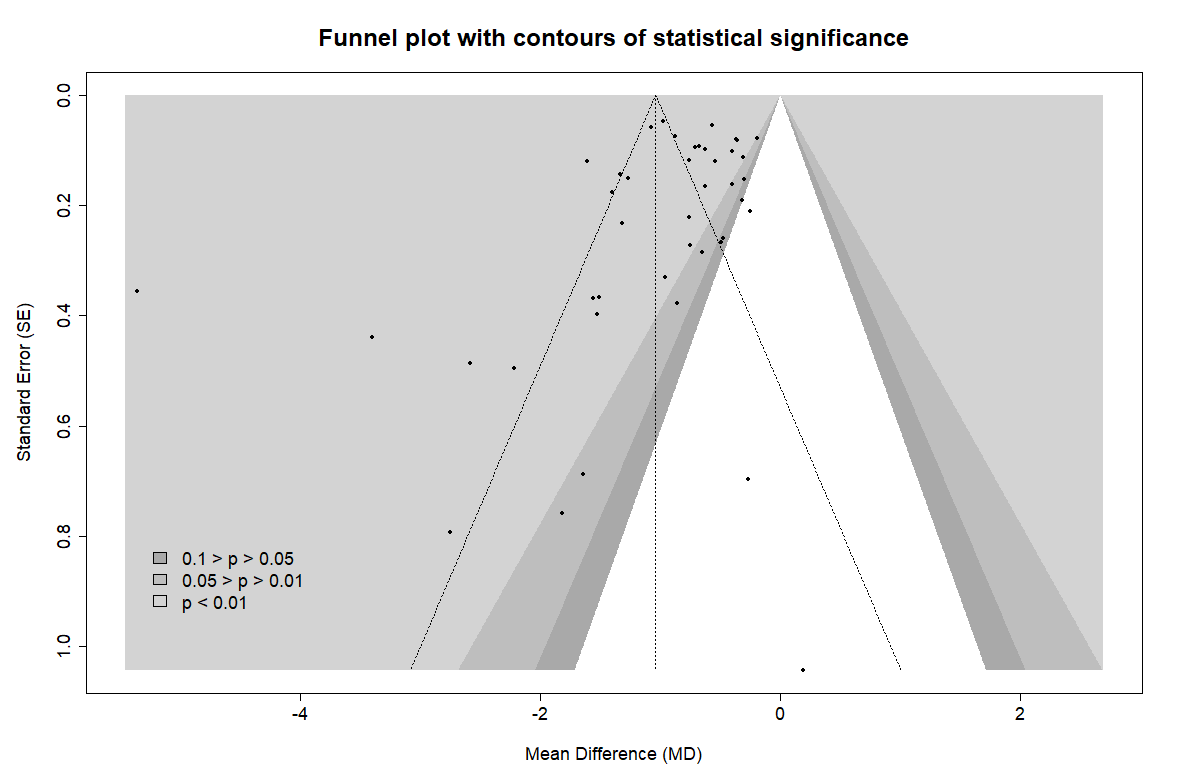


Supplementary Figure 24: Funnel plot with contours of statistical significance, for the assessment of publication bias in all available studies.


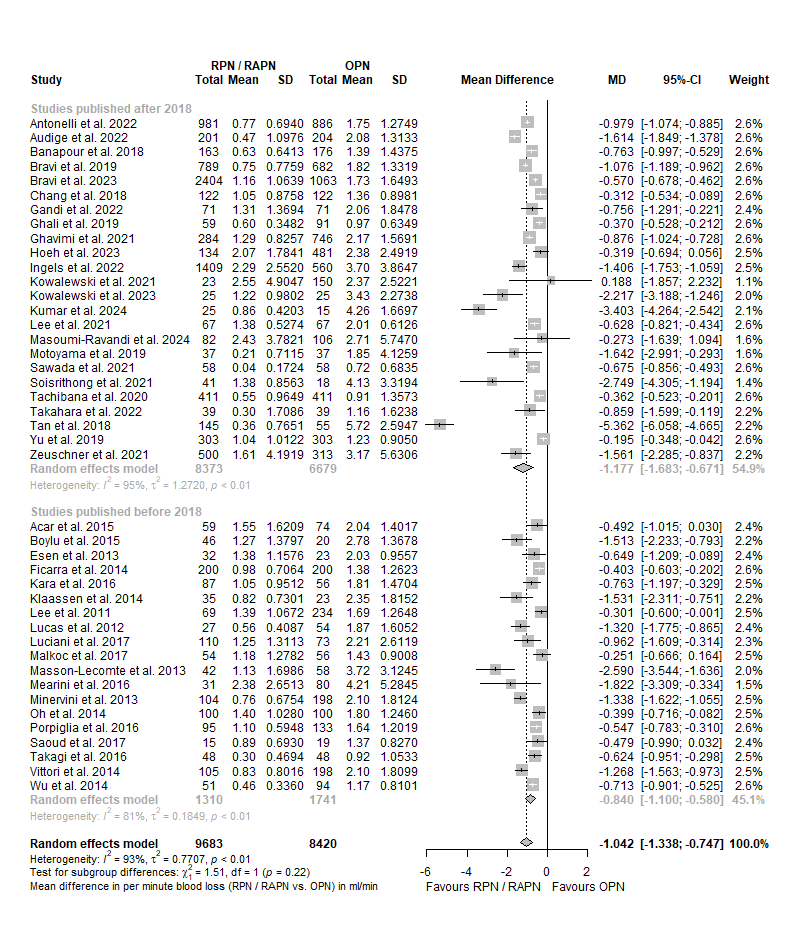


Supplementary Figure 25: Forest plot showing the estimation of the mean difference in Q (MD_Q_) along with its CI_95%_ in RPN/RAPN vs. OPN, for subgroups according to publication year.


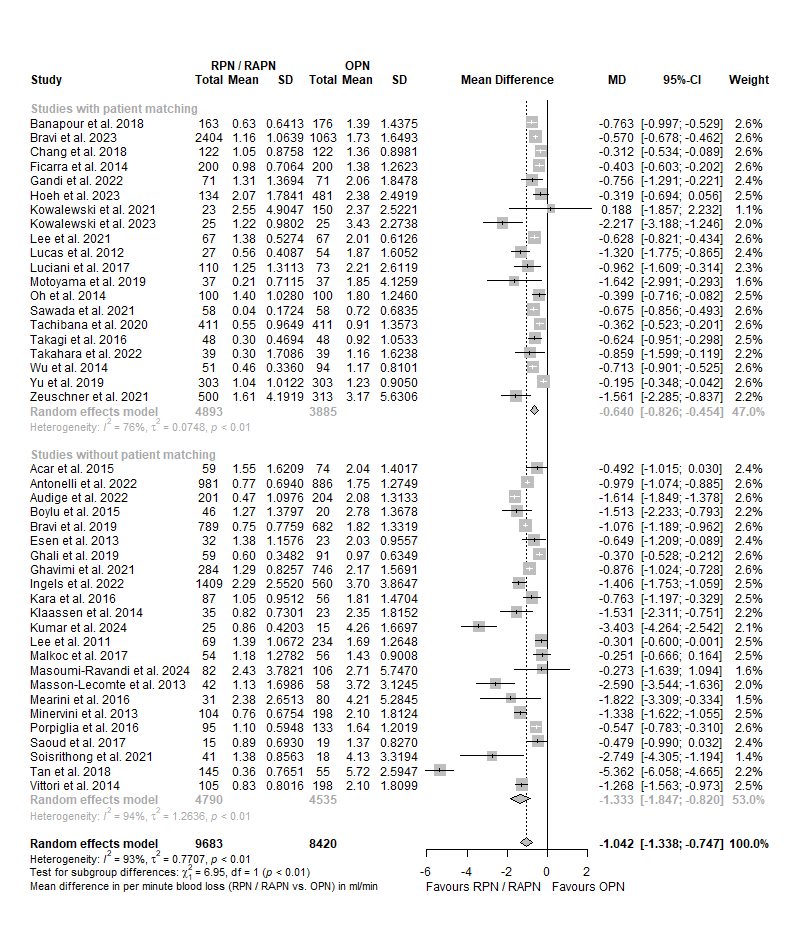


*
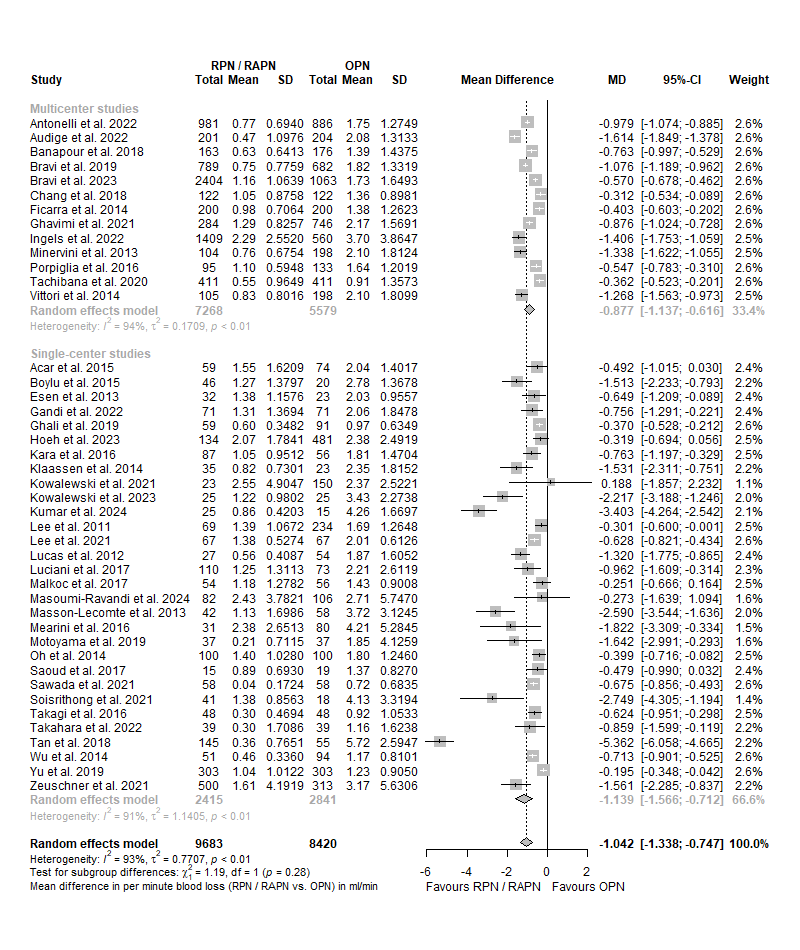

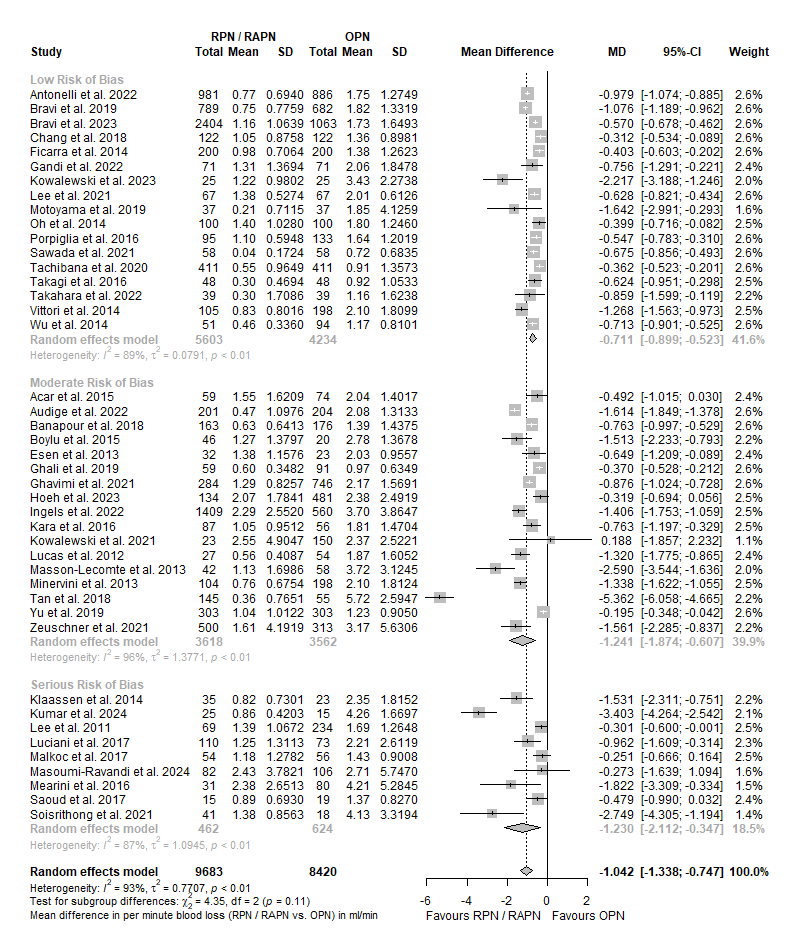
*

Supplementary Figure 26: Forest plot showing the estimation of the mean difference in Q (MD_Q_) along with its CI_95%_ in RPN/RAPN vs. OPN, for subgroups according to patient matching.

Supplementary Figure 27: Forest plot showing the estimation of the mean difference in Q (MD_Q_) along with its CI_95%_ in RPN/RAPN vs. OPN, for subgroups according to the number of referral centers involved.

Supplementary Figure 28: Forest plot showing the estimation of the mean difference in Q (MD_Q_) along with its CI_95%_ in RPN/RAPN vs. OPN, for subgroups according to ROBINS-I class.


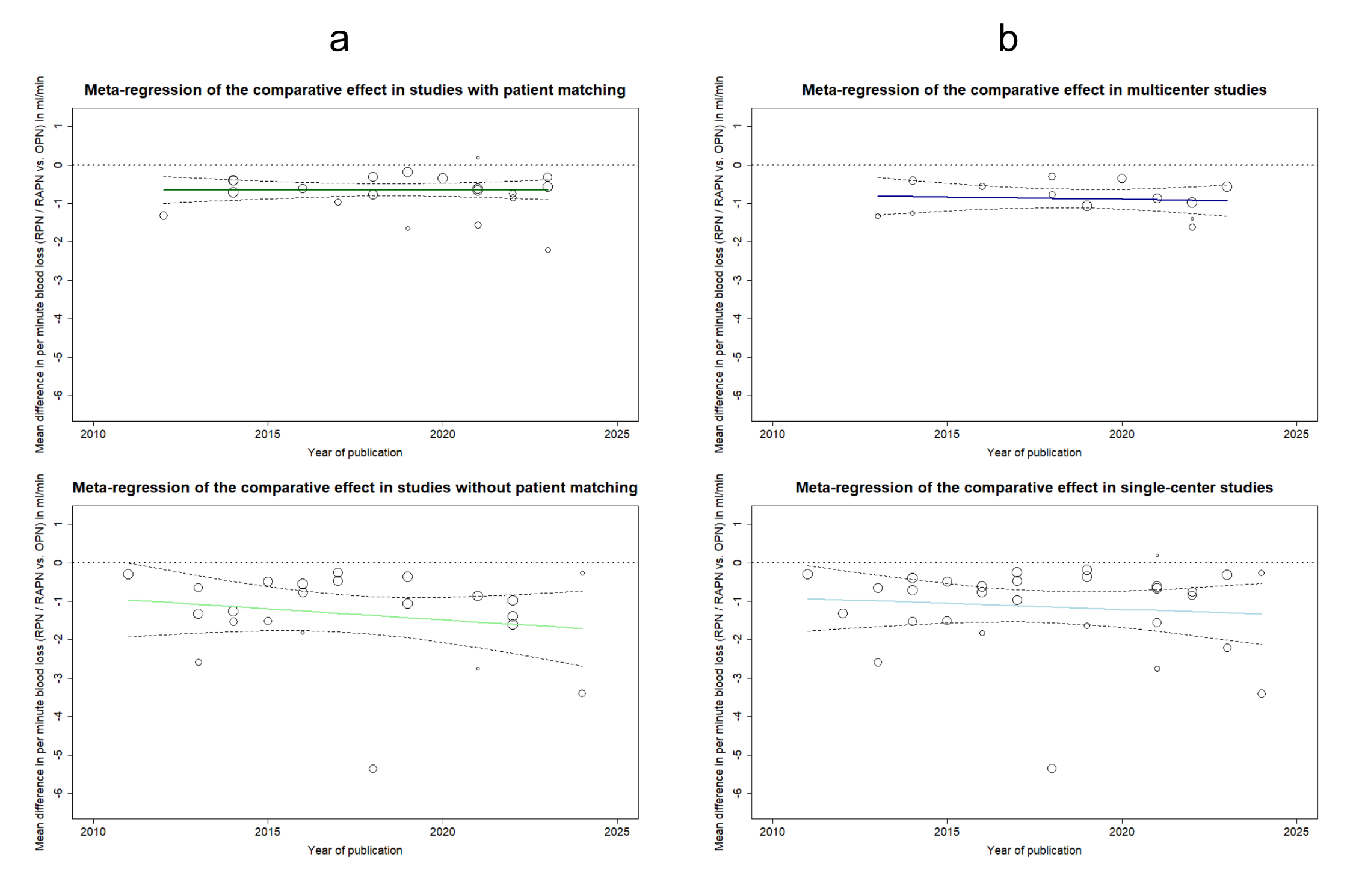


Supplementary Figure 29: Meta-regression analysis plots showing the change in the comparative effect (MD_Q_) between RPN/RAPN vs. OPN, along with its CI_95%_, in studies according to patient matching (a), and number of referral centers (b), using as moderator the year of publication.


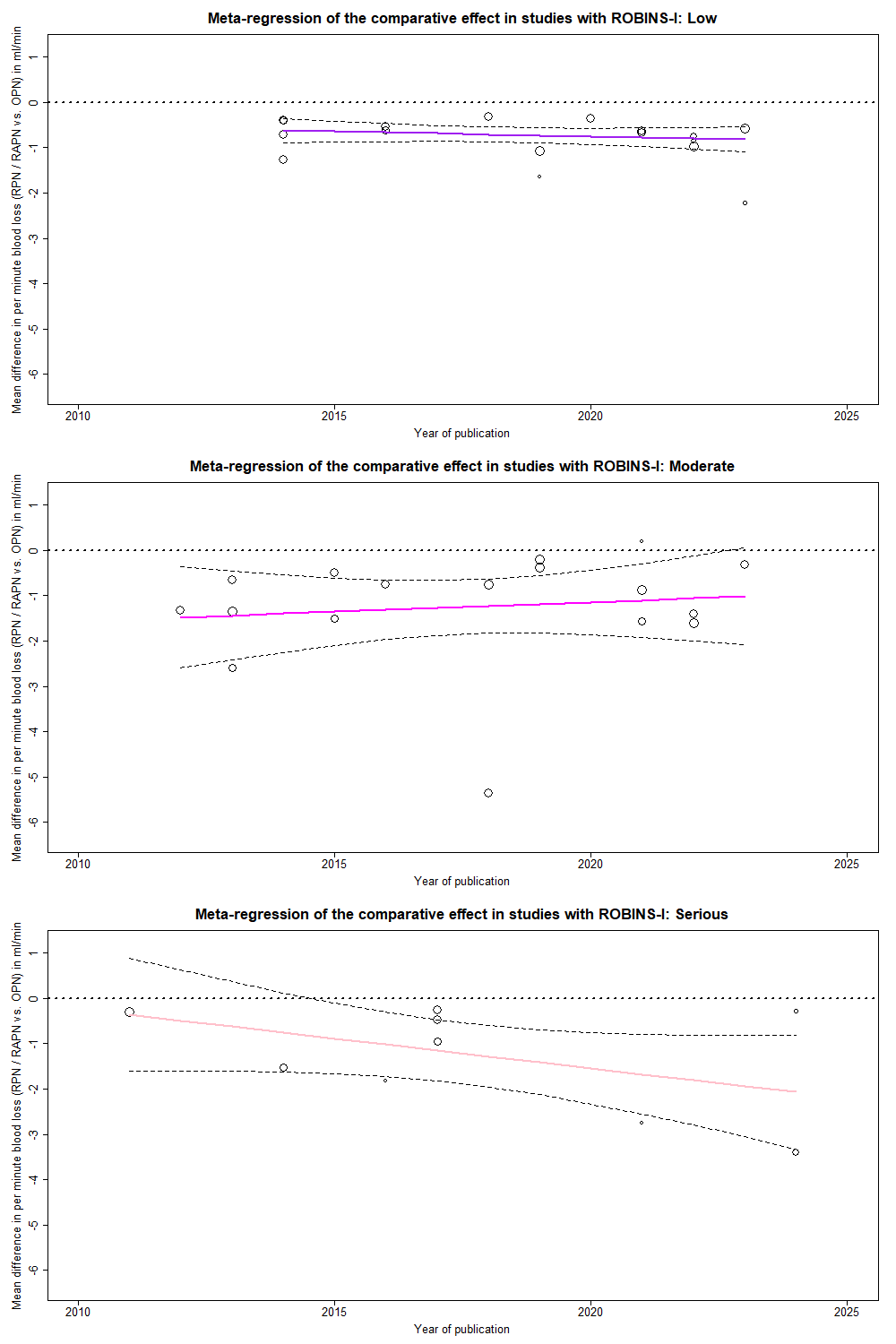


Supplementary Figure 30: Meta-regression analysis plots showing the change in the comparative effect (MD_Q_) between RPN/RAPN vs. OPN, along with its CI_95%_, in studies according to their ROBINS-I class.


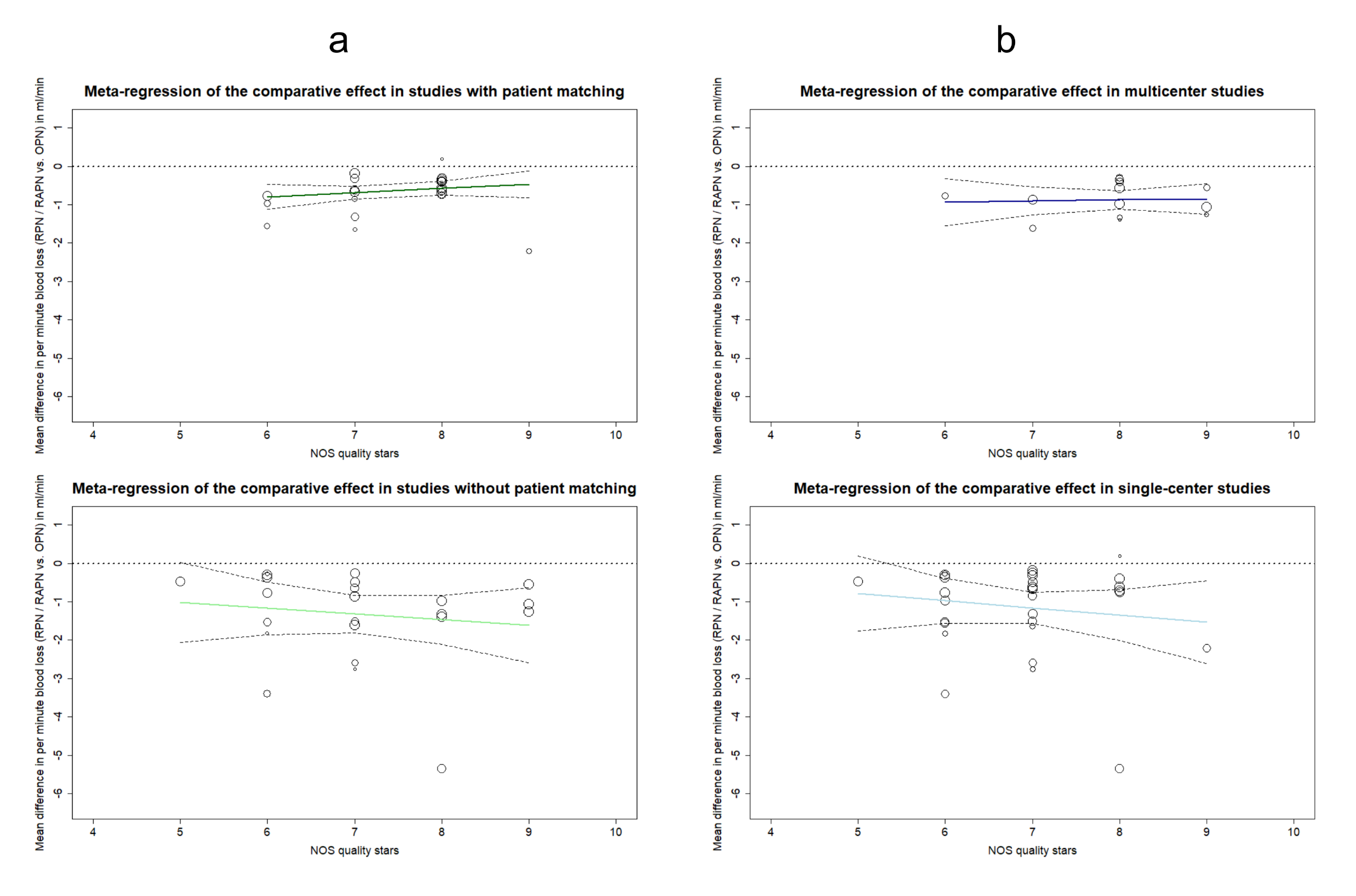


Supplementary Figure 31: Meta-regression analysis plots showing the change in the comparative effect (MD_Q_) between RPN/RAPN vs. OPN, along with its CI_95%_, in studies according to patient matching (a), and number of referral centers (b), using as moderator the number of quality stars from NOS.


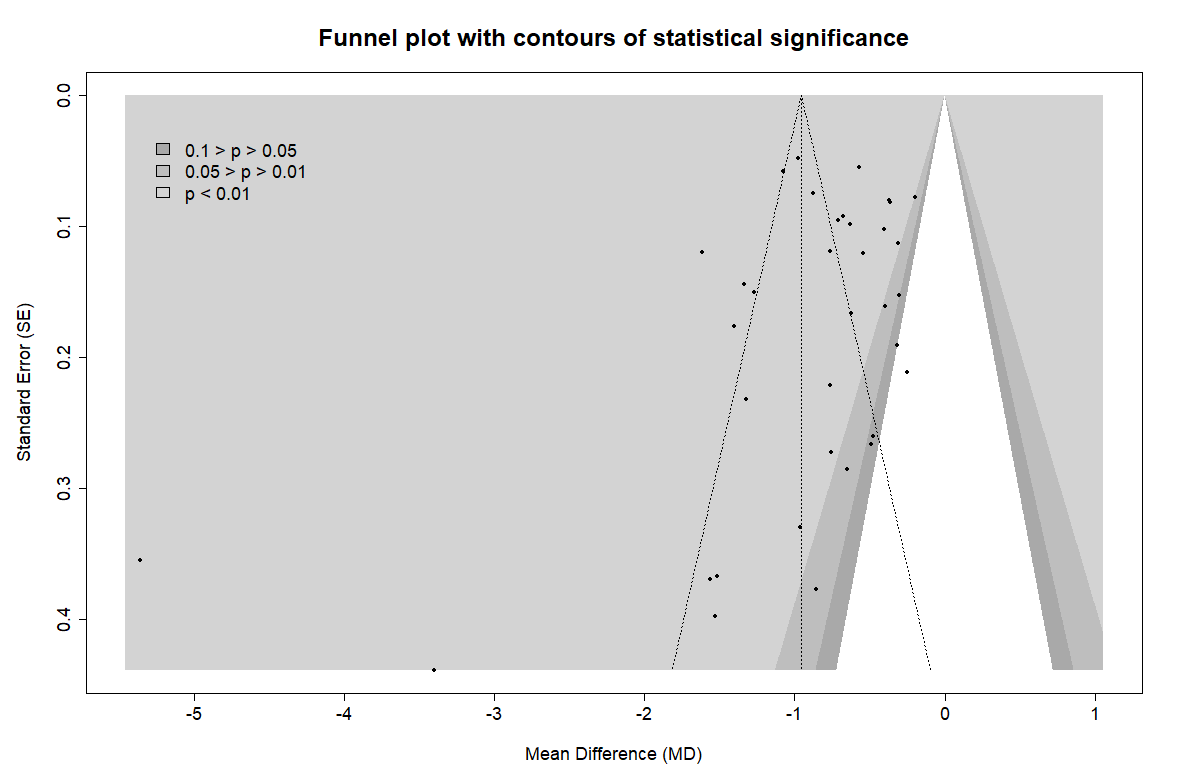


Supplementary Figure 32: Funnel plot with contours of statistical significance, for the assessment of publication bias in the subset of studies isolated at the first level of the sensitivity analysis.


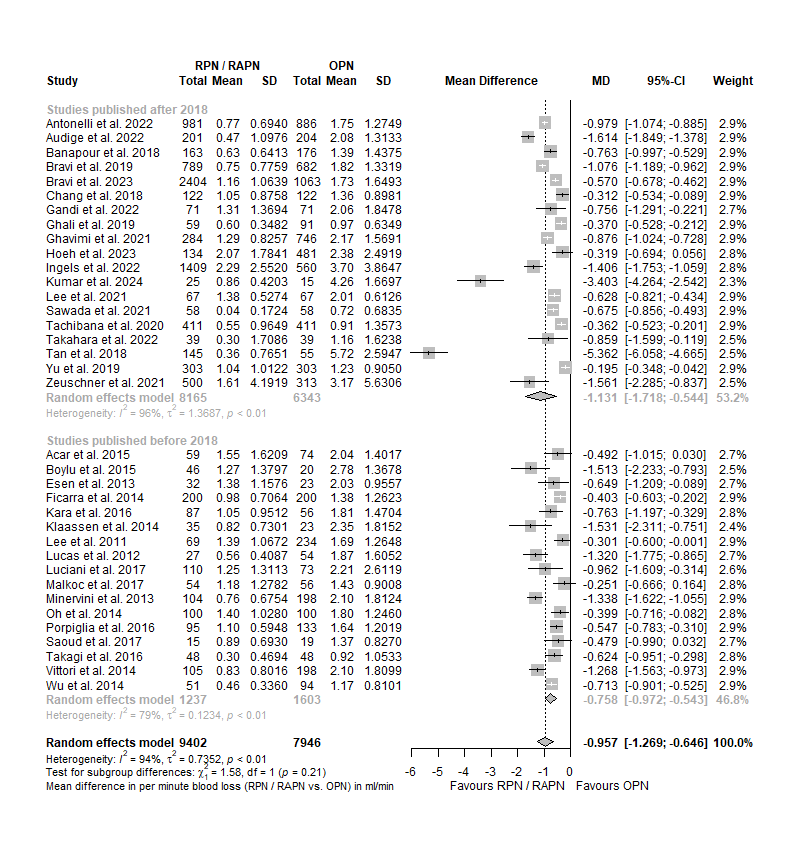


Supplementary Figure 33: Forest plot showing the estimation of the mean difference in Q (MD_Q_) along with its CI_95%_ in RPN/RAPN vs. OPN, for subgroups according to publication year and for those studies with increased accuracy of reported results, isolated at the first level of the sensitivity analysis.


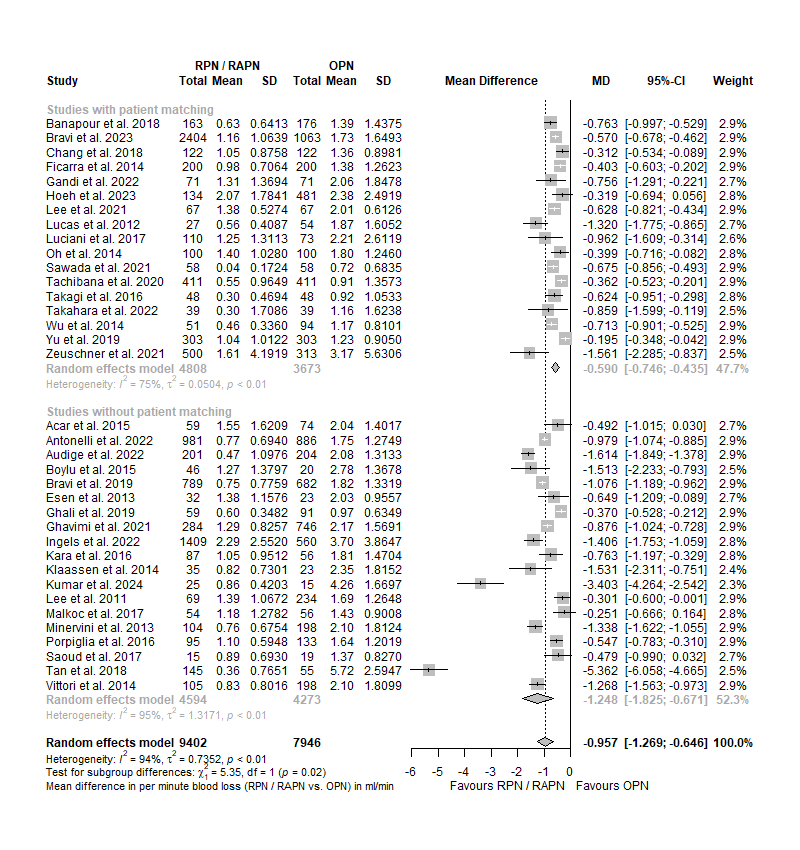


Supplementary Figure 34: Forest plot showing the estimation of the mean difference in Q (MD_Q_) along with its CI_95%_ in RPN/RAPN vs. OPN, for subgroups according to patient matching and for those studies with increased accuracy of reported results, isolated at the first level of the sensitivity analysis.


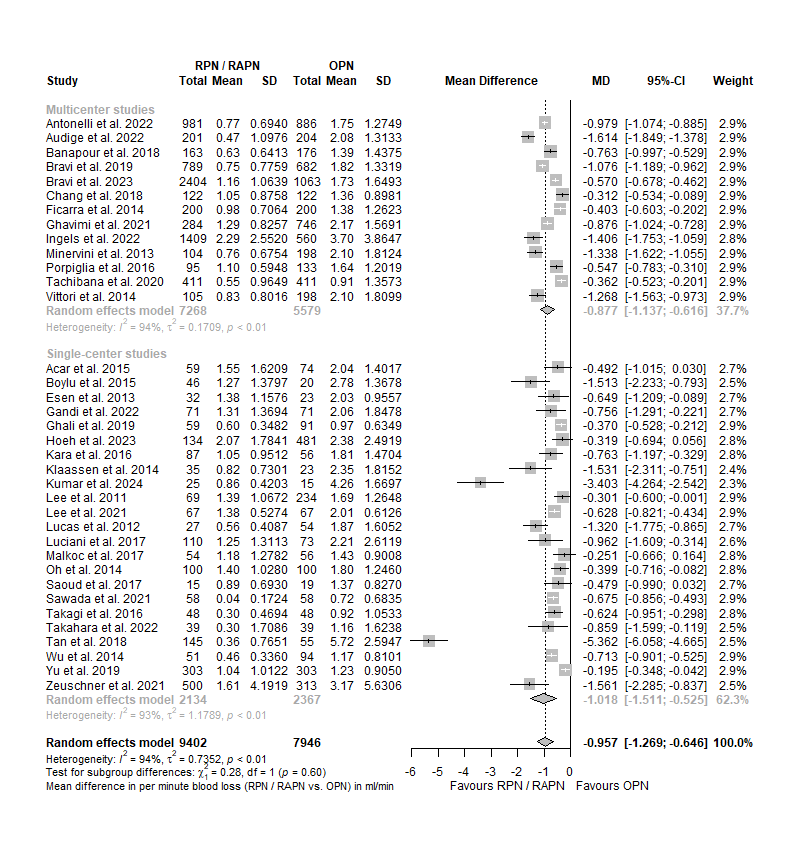


Supplementary Figure 35: Forest plot showing the estimation of the mean difference in Q (MD_Q_) along with its CI_95%_ in RPN/RAPN vs. OPN, for subgroups according to the number of referral centers involved, and for those studies with increased accuracy of reported results, isolated at the first level of the sensitivity analysis.


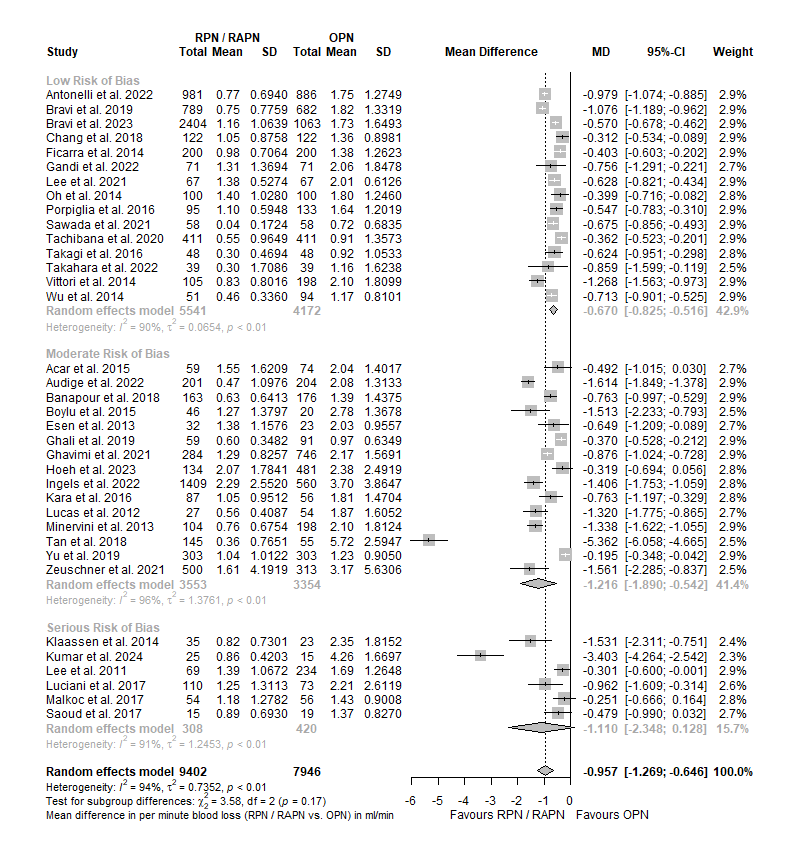


Supplementary Figure 36: Forest plot showing the estimation of the mean difference in Q (MD_Q_) along with its CI_95%_ in RPN/RAPN vs. OPN, for subgroups according to ROBINS-I class and for those studies with increased accuracy of reported results, isolated at the first level of the sensitivity analysis.


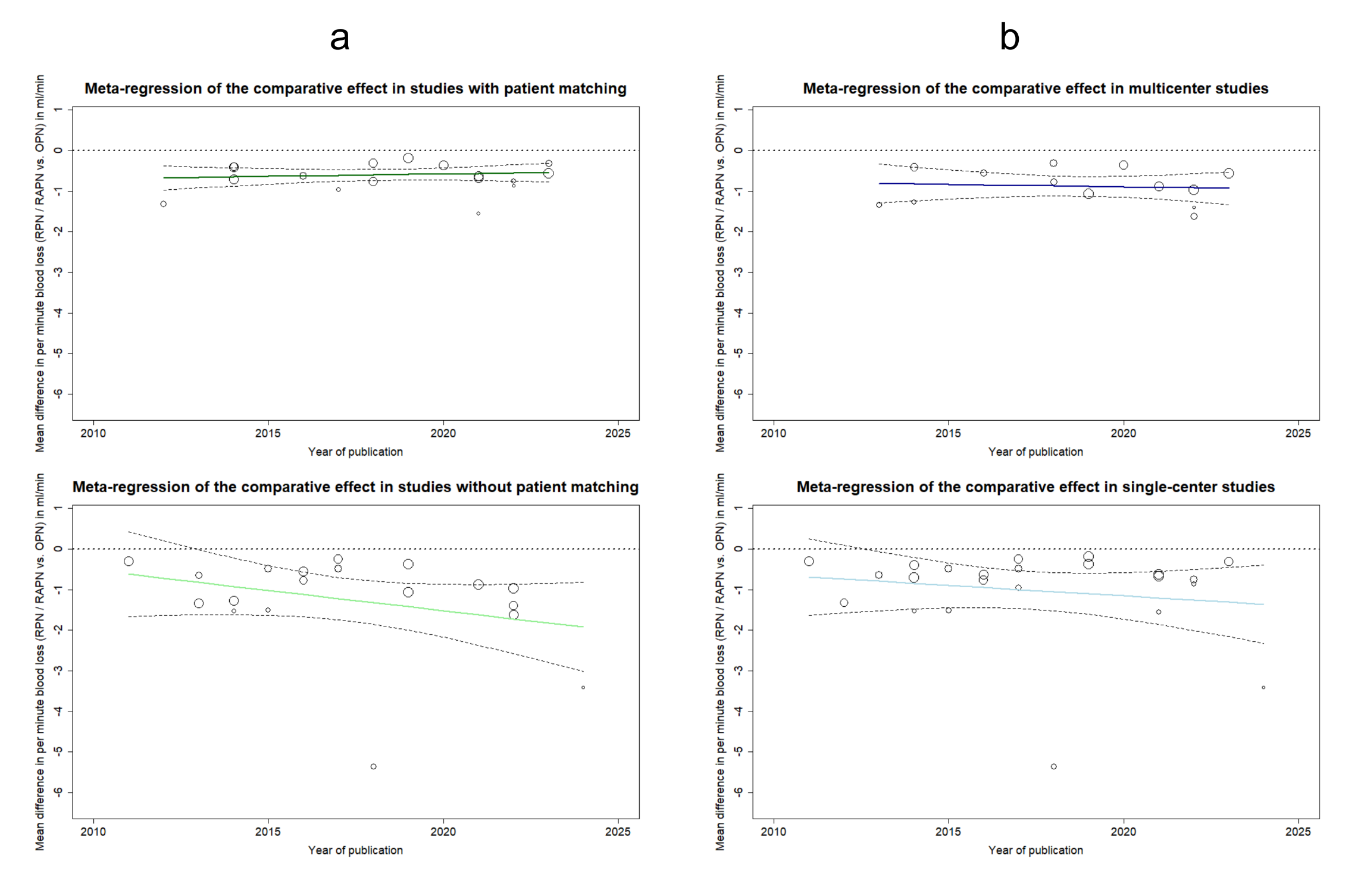


Supplementary Figure 37: Meta-regression analysis plots showing the change in the comparative effect (MD_Q_) between RPN/RAPN vs. OPN, along with its CI_95%_, in studies with increased accuracy in their reported results, according to patient matching (a), and number of referral centers (b), using as moderator the publication year.


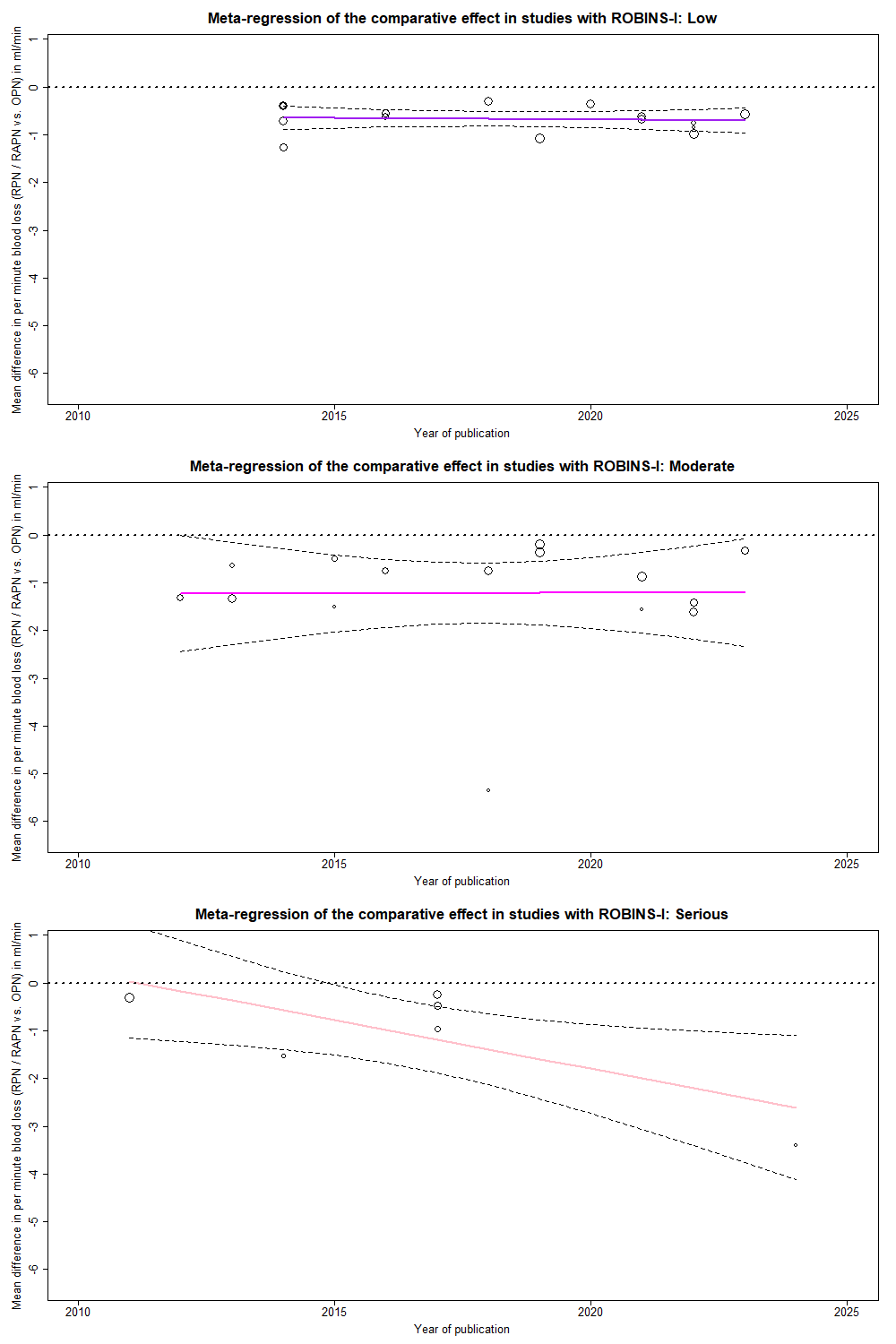


Supplementary Figure 38: Meta-regression analysis plots showing the change in the comparative effect (MD_Q_) between RPN/RAPN vs. OPN, along with its CI_95%_, in studies with increased accuracy in reported results, according to their ROBINS-I class.


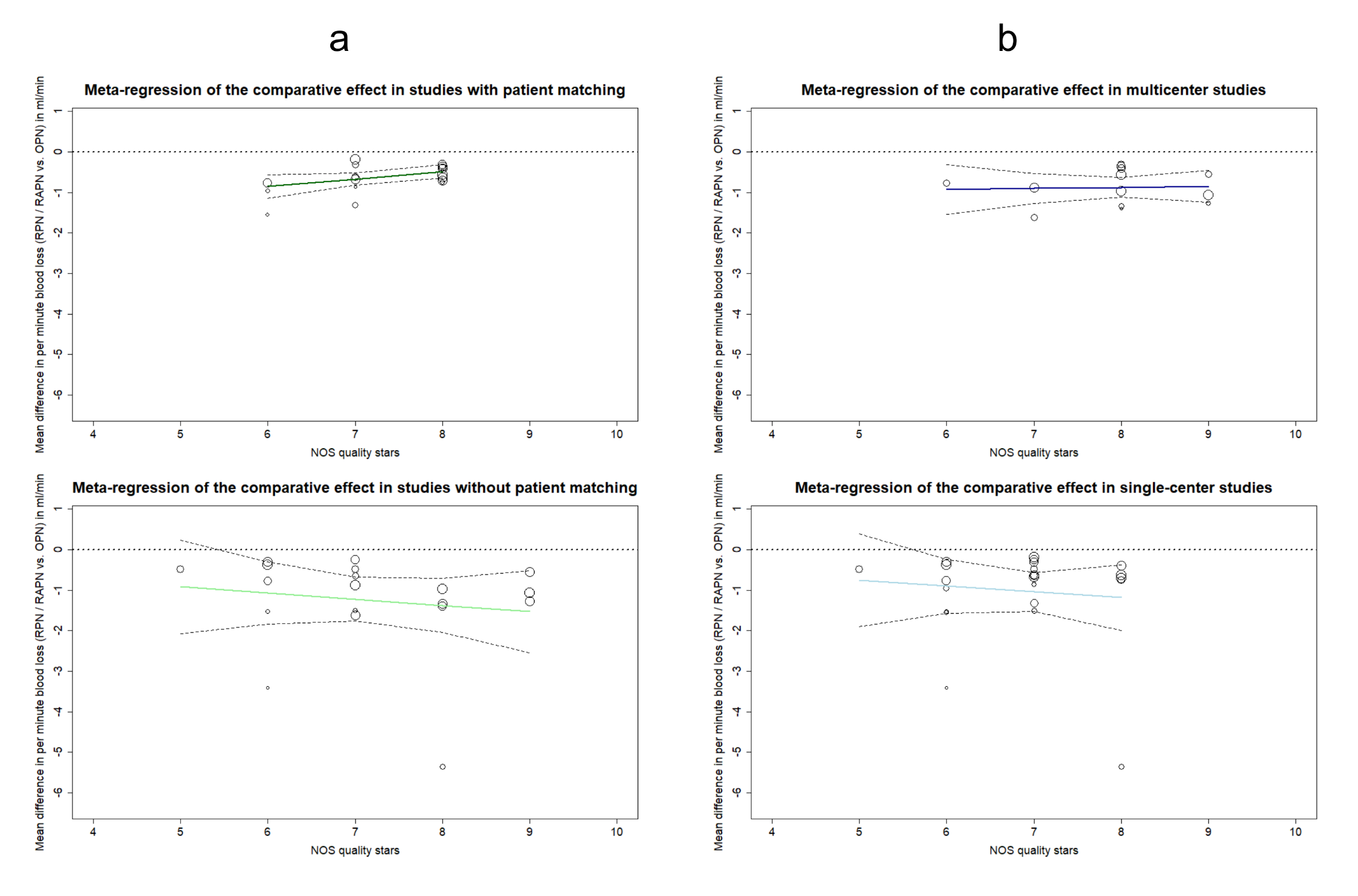


Supplementary Figure 39: Meta-regression analysis plots showing the change in the comparative effect (MD_Q_) between RPN/RAPN vs. OPN, along with its CI_95%_, in studies with increased accuracy in their reported results, according to patient matching (a), and number of referral centers (b), using as moderator the number of quality stars from NOS.


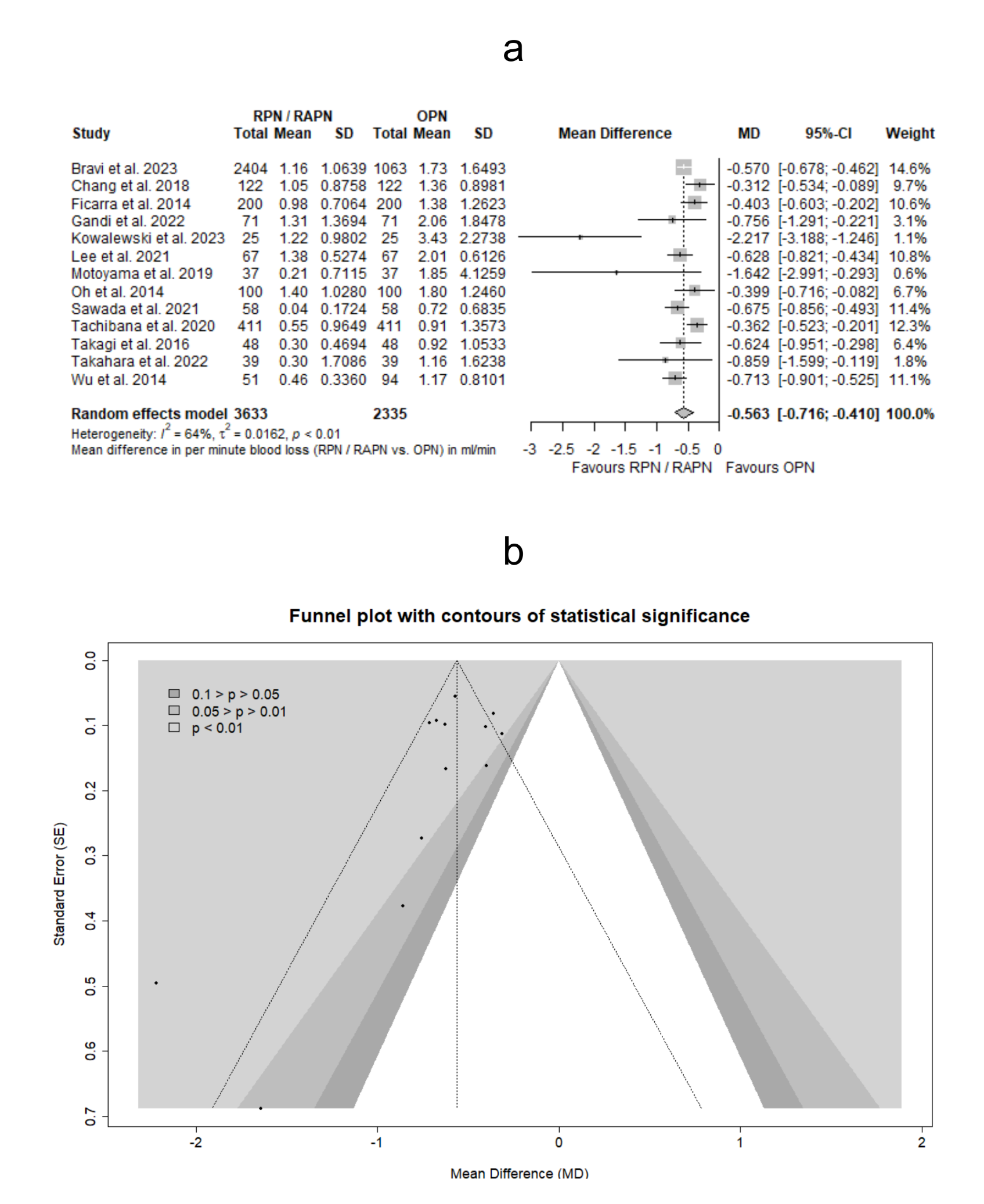


Supplementary Figure 40: Forest plot showing the estimation of the mean difference in Q (MD_Q_) along with its CI_95%_ in RPN/RAPN vs. OPN, for studies of low risk of bias with patient matching, isolated at the second level of the sensitivity analysis (a). Funnel plot with contours of statistical significance, for the assessment of publication bias in the respective study set (b).


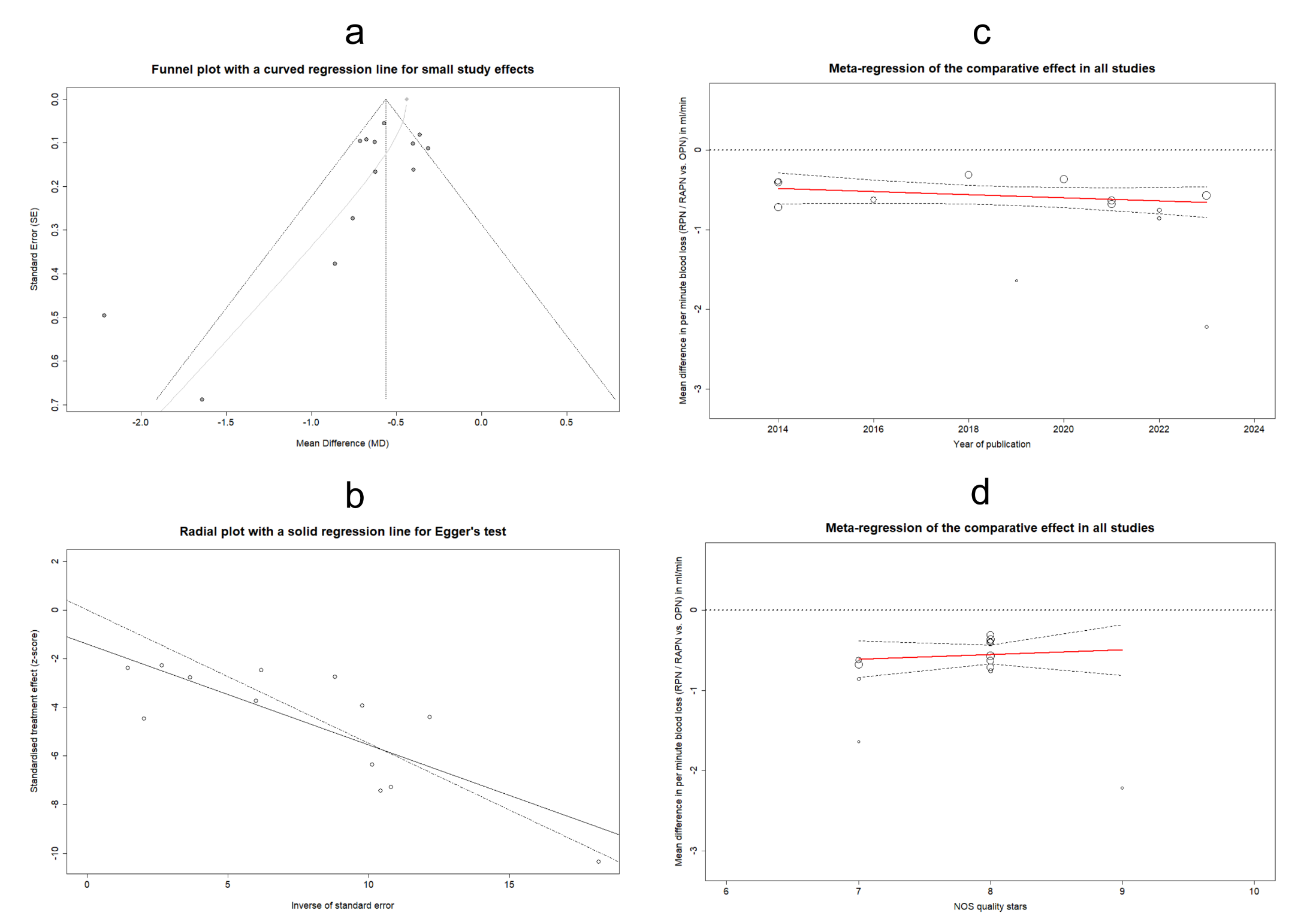


Supplementary Figure 41: Funnel plot presenting the publication bias assessment in matched analyses with low risk of bias, incorporating a curved regression line to investigate for small study effects (a). Radial plot complemented with an integrated regression line, to assess the significance of publication bias using the Egger's test (b). Meta-regression analysis plots showing the change in the comparative effect (MD_Q_) between RPN/RAPN vs. OPN, along with the CI_95%_, in the respective study set, using as moderator the publication year (c) and the NOS quality score (d).


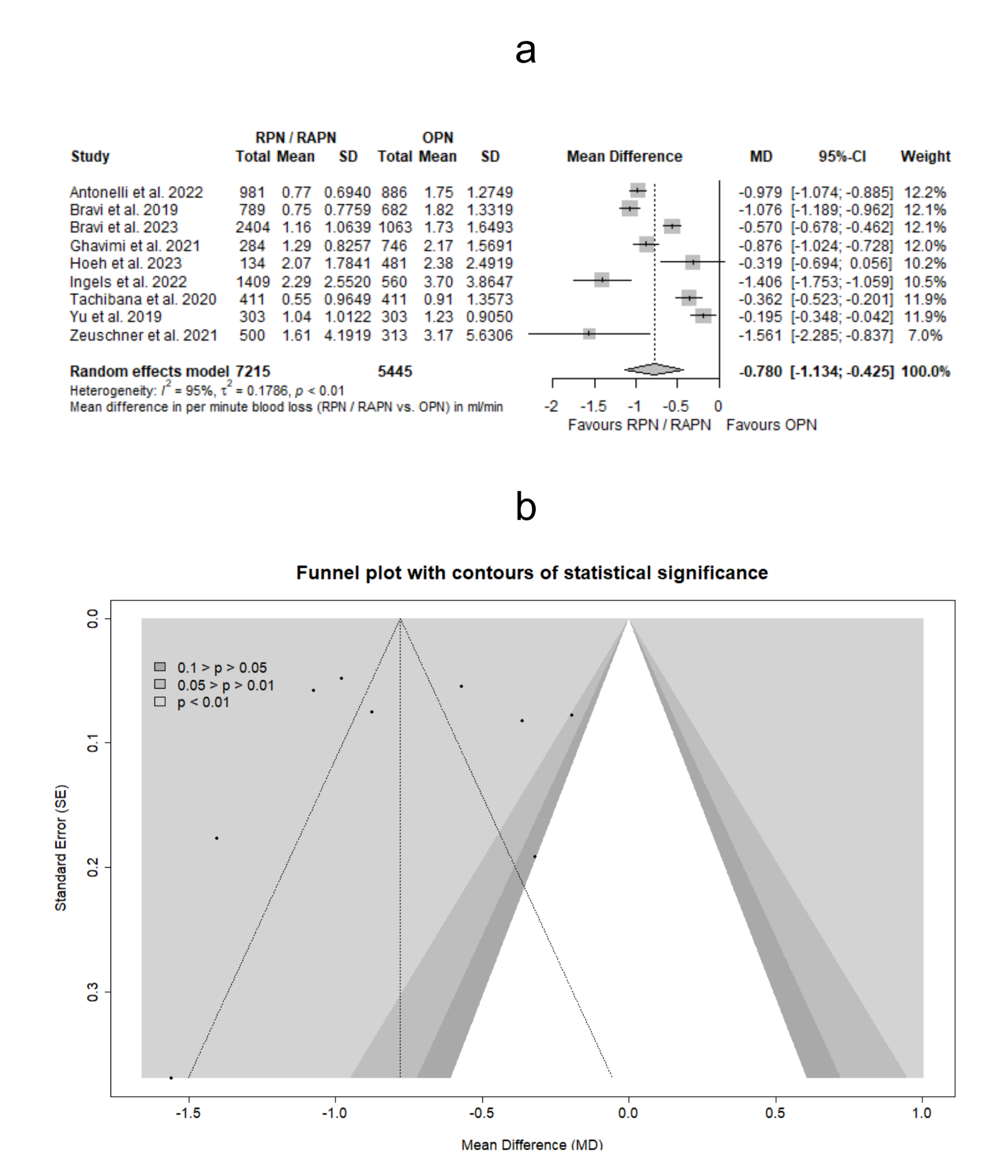


Supplementary Figure 42: Forest plot showing the estimation of the mean difference in Q (MD_Q_) along with its CI_95%_ in RPN/RAPN vs. OPN, for studies with large sample sizes, isolated at the third level of the sensitivity analysis (a). Funnel plot with contours of statistical significance, for the assessment of publication bias in the respective study set (b).


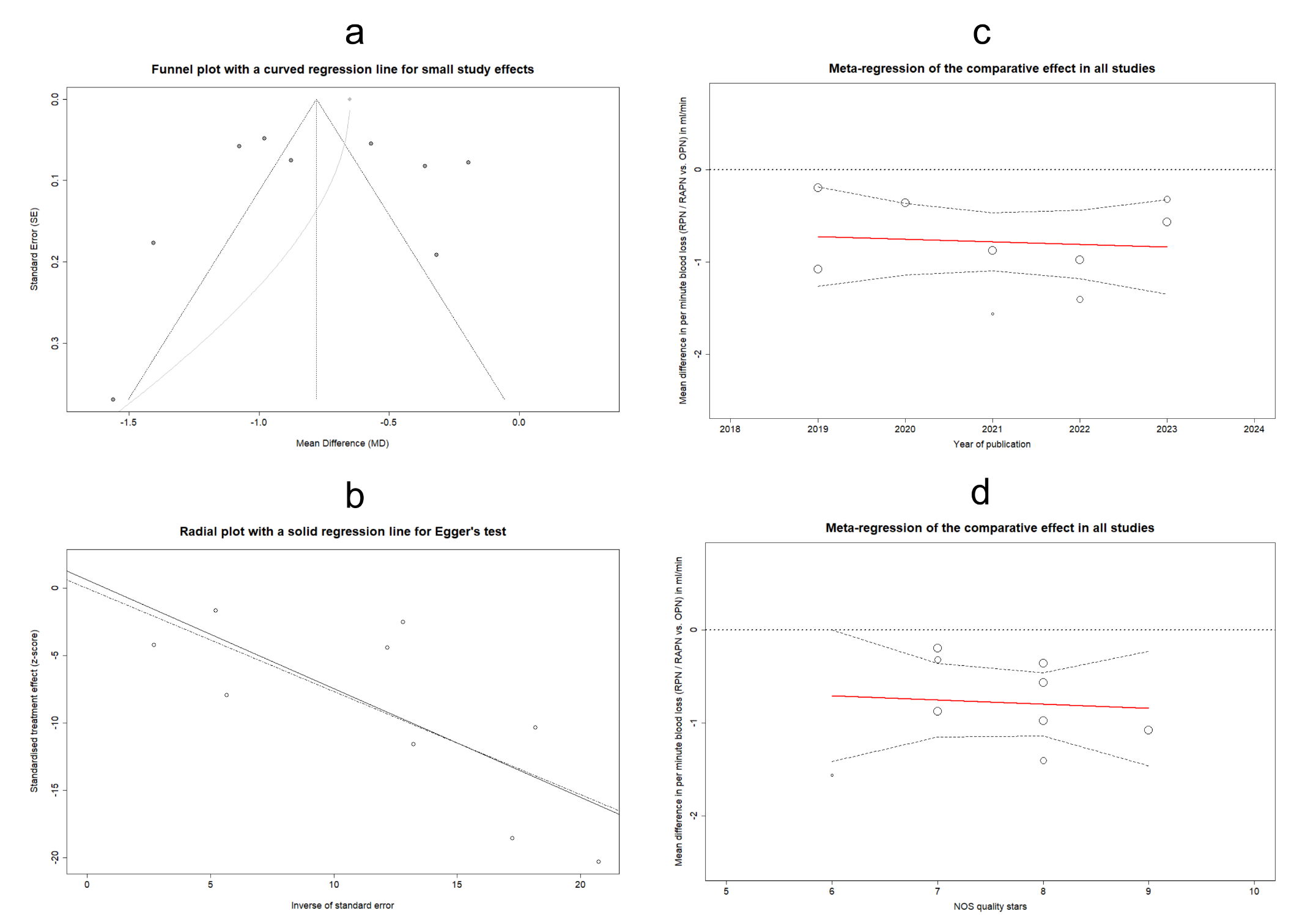


Supplementary Figure 43: Funnel plot presenting the publication bias assessment in studies with large sample sizes, incorporating a curved regression line to investigate for small study effects (a). Radial plot complemented with an integrated regression line, to assess the significance of publication bias using the Egger's test (b). Meta-regression analysis plots showing the change in the comparative effect (MD_Q_) between RPN/RAPN vs. OPN, along with the CI_95%_, in the respective study set, using as moderator the publication year (c) and the NOS quality score (d).


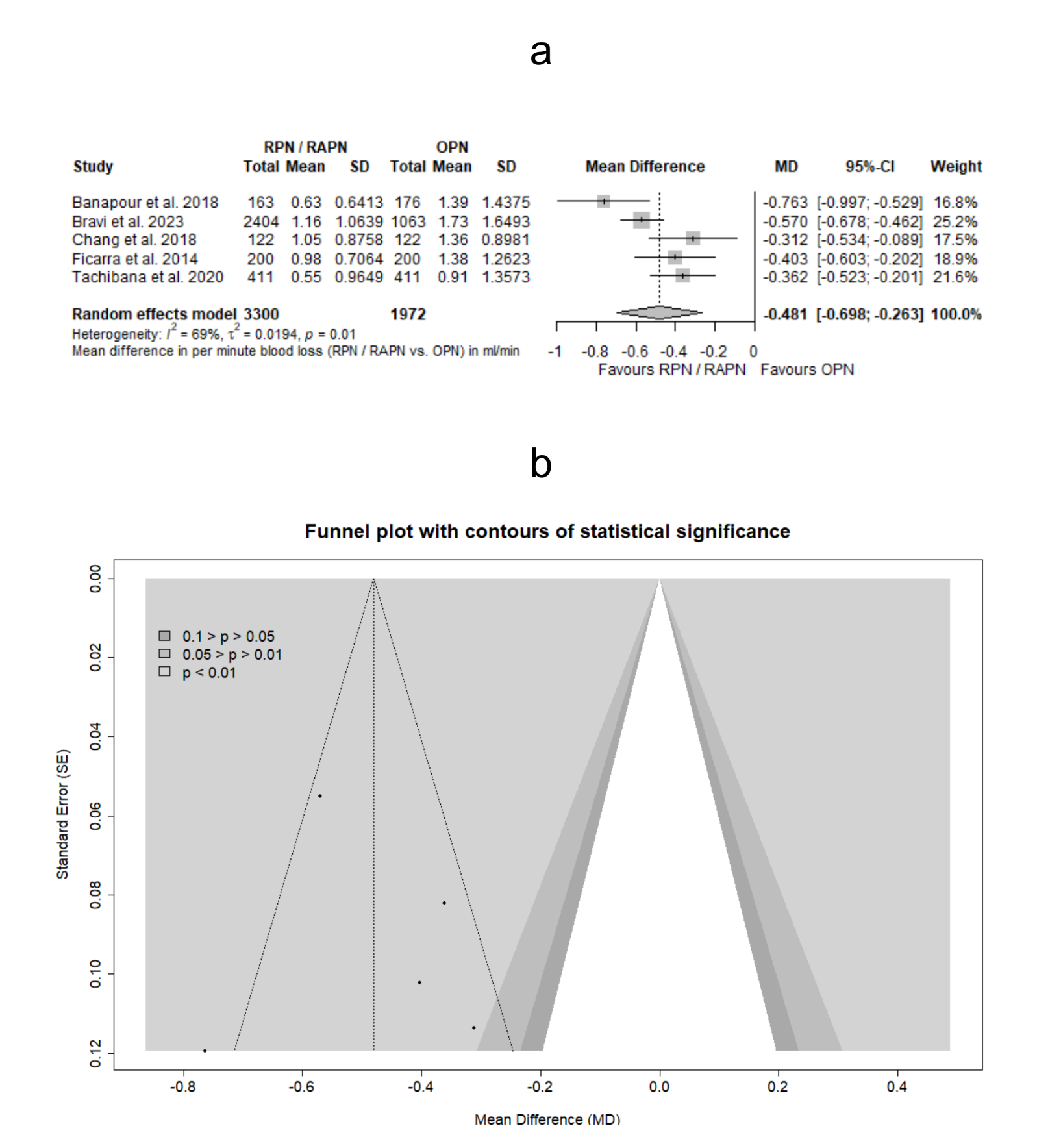


Supplementary Figure 44: Forest plot showing the estimation of the mean difference in Q (MD_Q_) along with its CI_95%_ in RPN/RAPN vs. OPN, for multicenter studies with patient matching, isolated at the fourth level of the sensitivity analysis (a). Funnel plot with contours of statistical significance, for the assessment of publication bias in the respective study set (b).


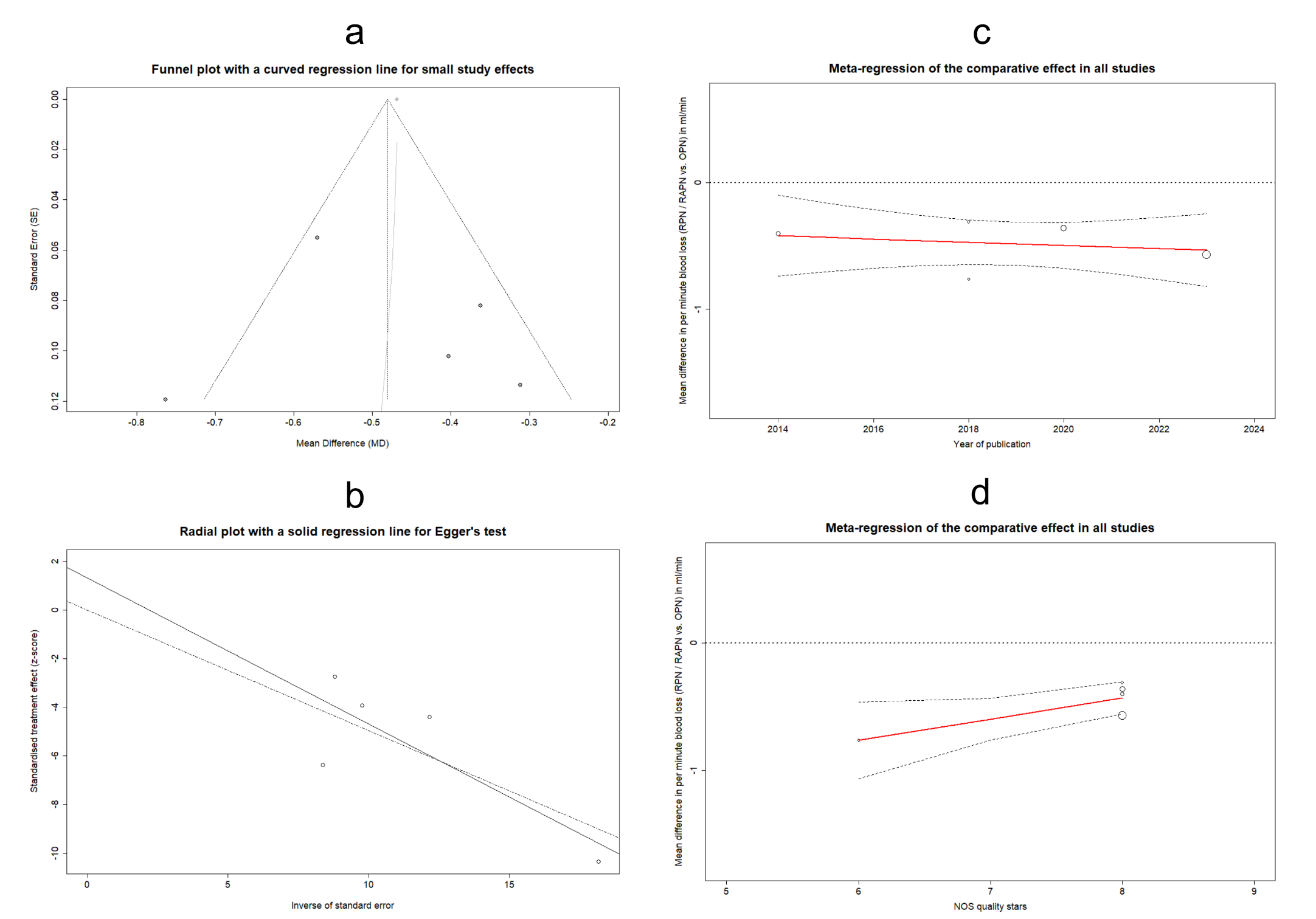


Supplementary Figure 45: Funnel plot presenting the publication bias assessment in multicenter studies with patient matching, incorporating a curved regression line to investigate for small study effects (a). Radial plot complemented with an integrated regression line, to assess the significance of publication bias using the Egger's test (b). Meta-regression analysis plots showing the change in the comparative effect (MD_Q_) between RPN/RAPN vs. OPN, along with the CI_95%_, in the respective study set, using as moderator the publication year (c) and the NOS quality score (d).


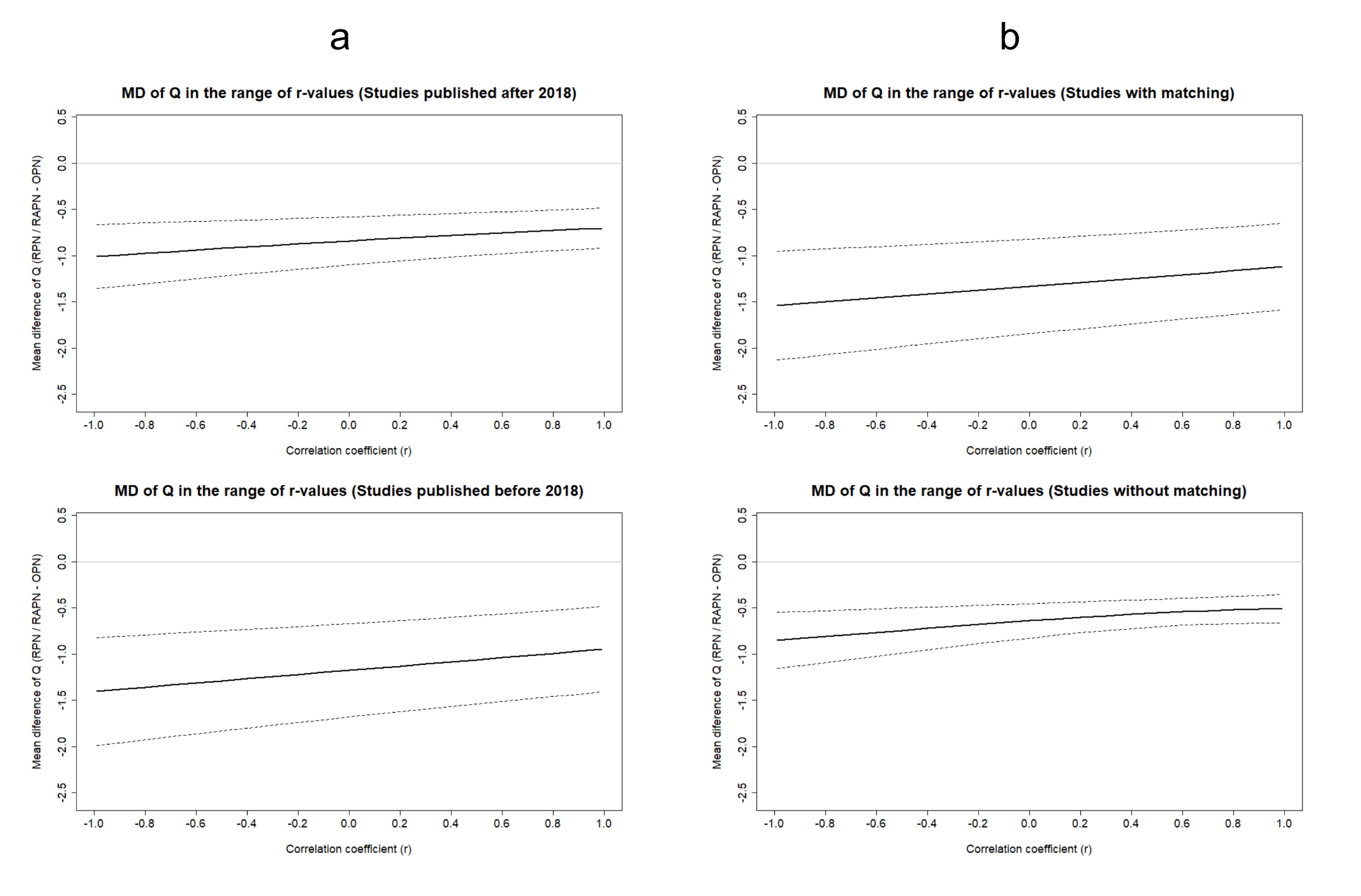


Supplementary Figure 46: Plots depicting the variation in the comparative effect (MD_Q_) between RPN/RAPN and OPN, along with its CI_95%_, for consecutive r-values, at the fifth level of the sensitivity analysis, by publication year (a) and patient matching (b).


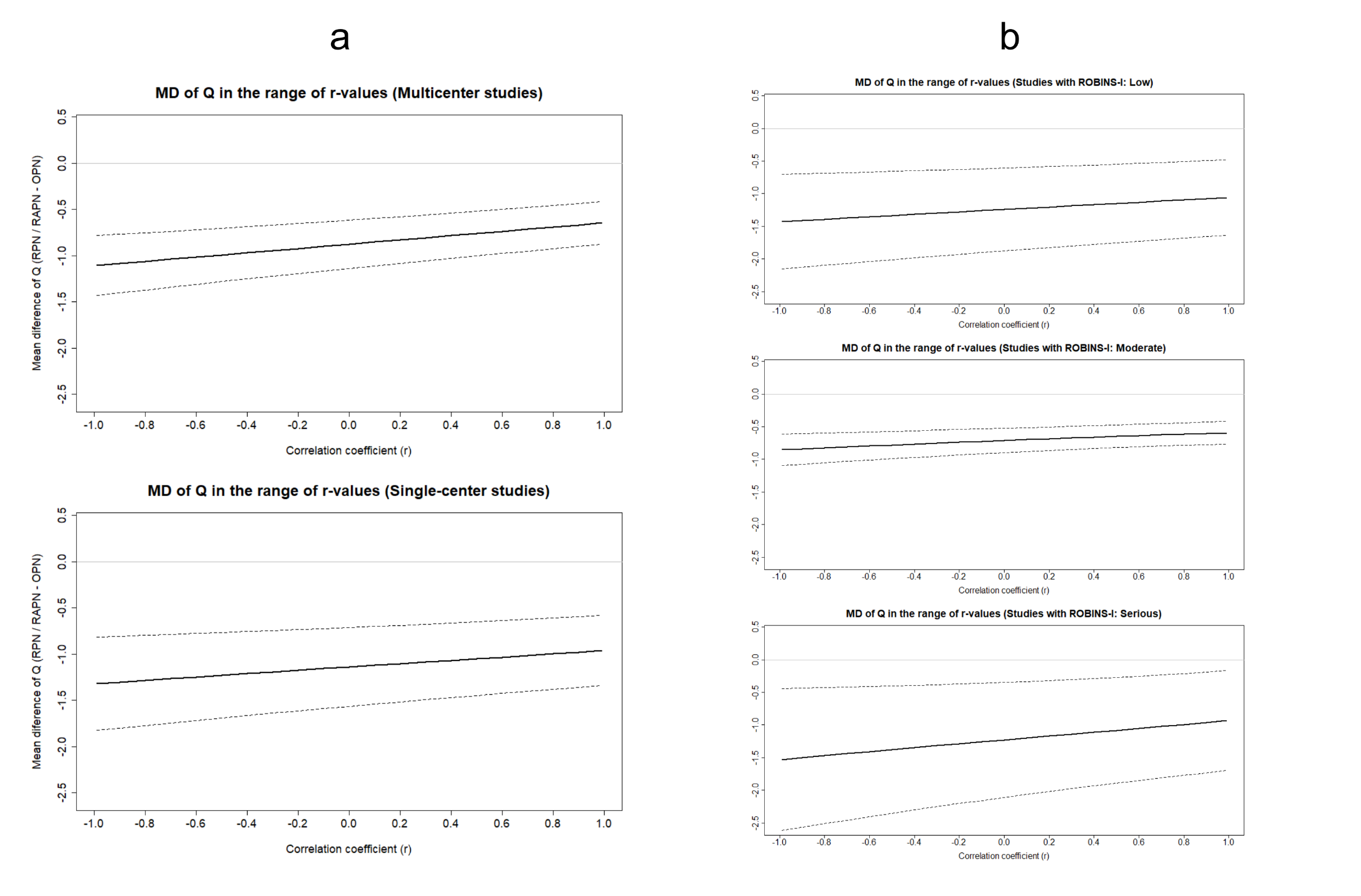


Supplementary Figure 47: Plots depicting the variation in the comparative effect (MD_Q_) between RPN/RAPN and OPN, along with its CI_95%_, for consecutive r-values, at the fifth level of the sensitivity analysis, by the number of referral centers involved (a) and ROBINS-I class (b).
