# Supplementary material for: Comparing robotic and open partial nephrectomy under the prism of surgical precision: a meta-analysis of the average blood loss rate as a novel variable
Source: J Robot Surg. 2024 Aug 7;18(1):313. doi: 10.1007/s11701-024-02060-z (PMC11306375; doi:10.1007/s11701-024-02060-z)
Supplement: Supplementary file 4 — Supplementary file4 (DOCX 4983 KB) [file 11701_2024_2060_MOESM4_ESM.docx]

SENSITIVITY ANALYSIS FOREST PLOTS

(POOLED STUDIES)

*
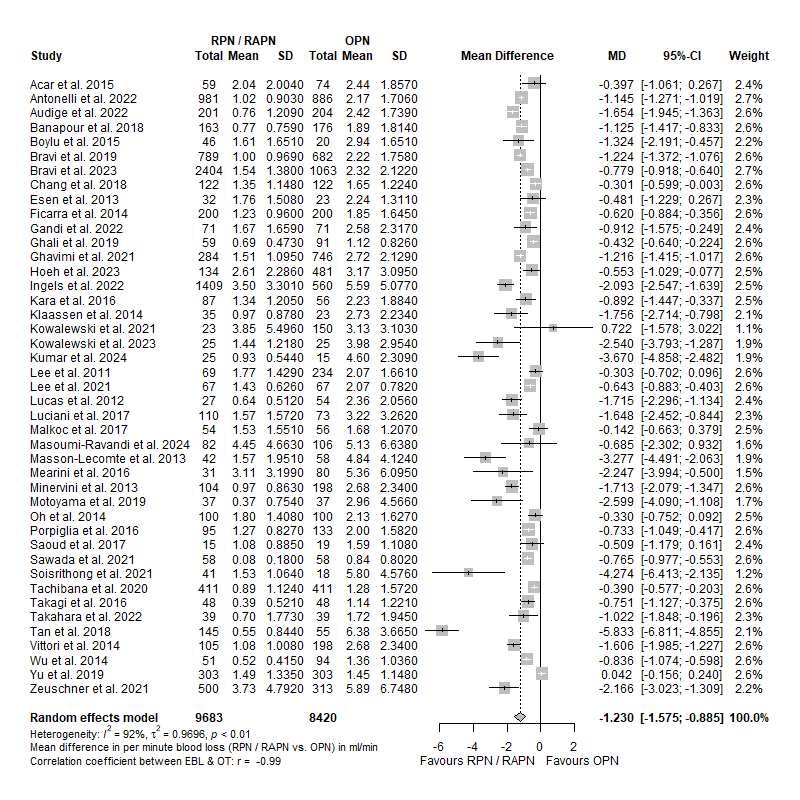

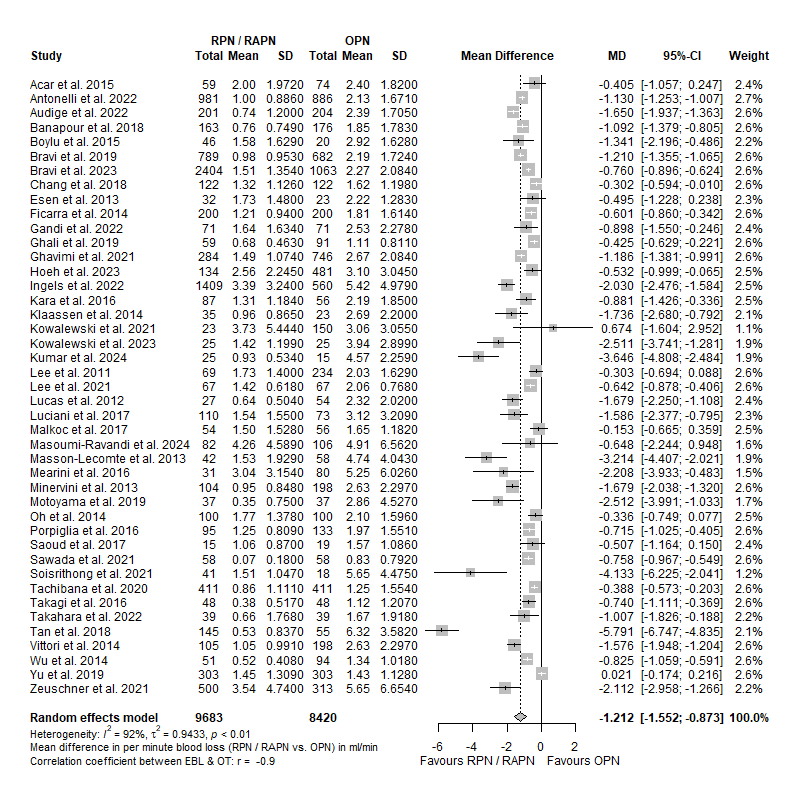

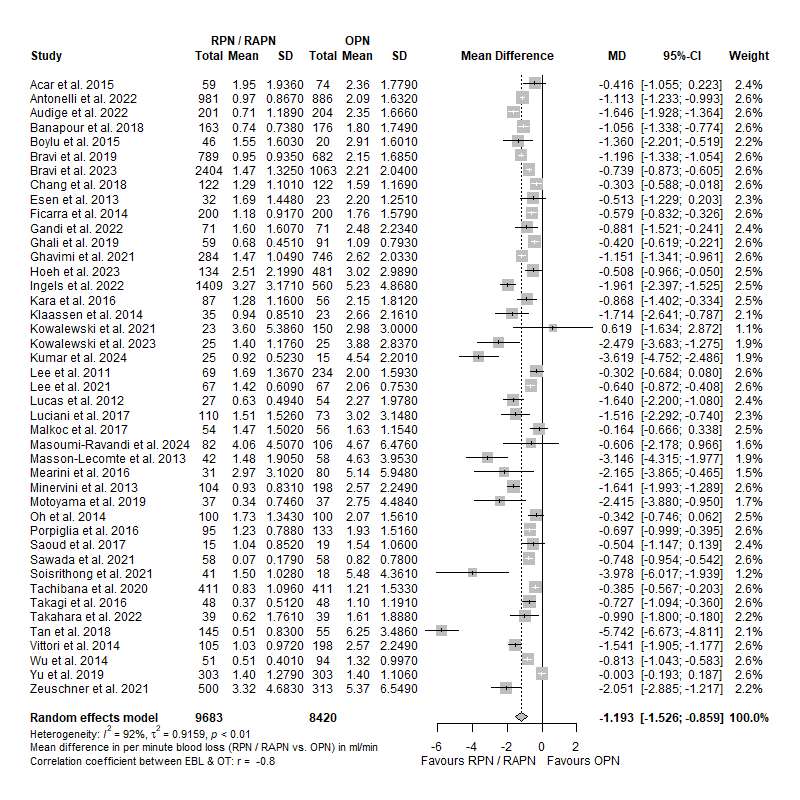

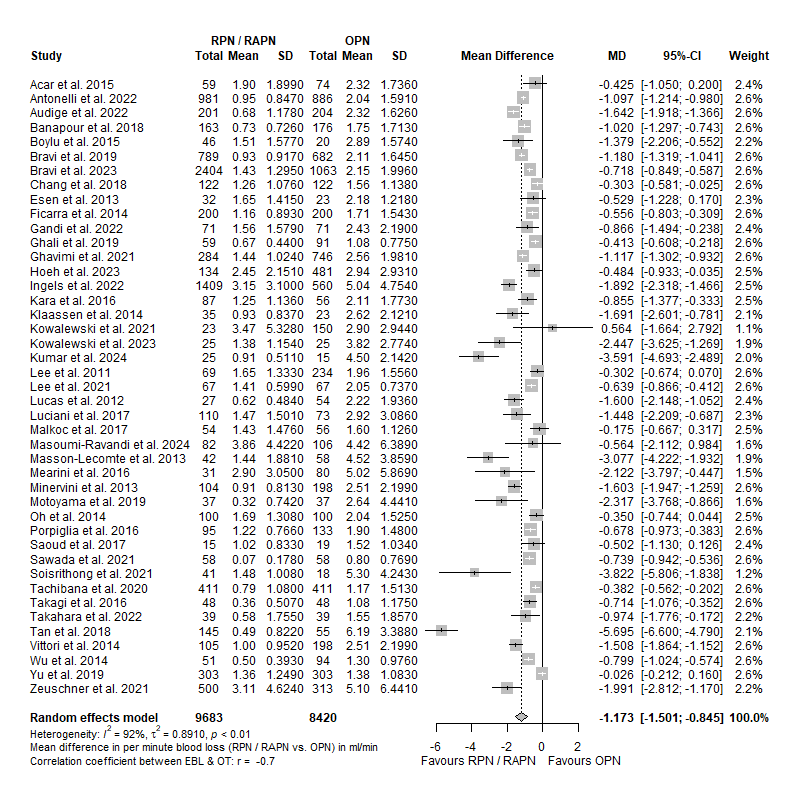

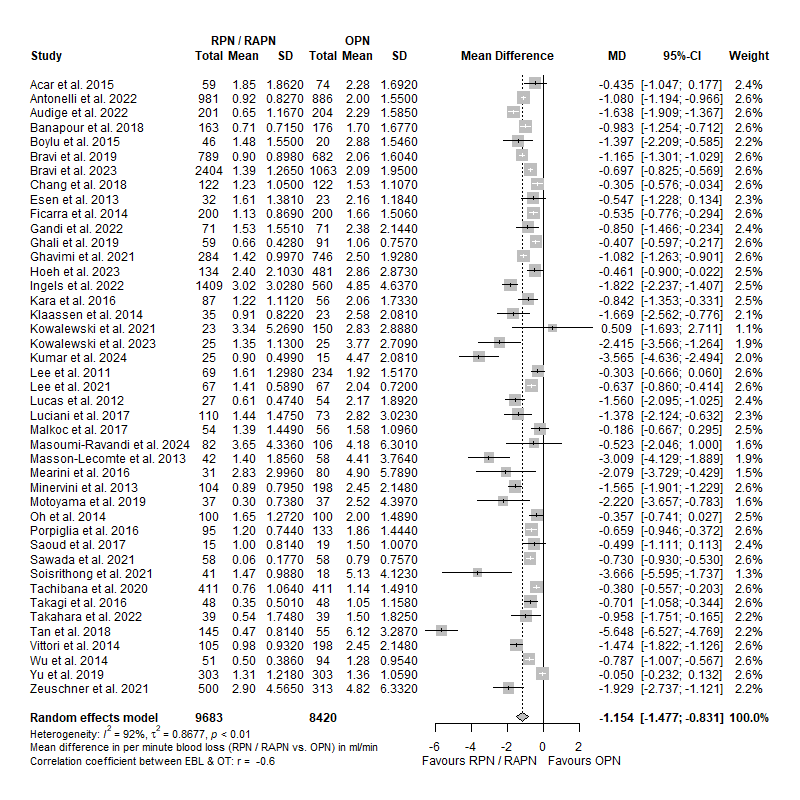

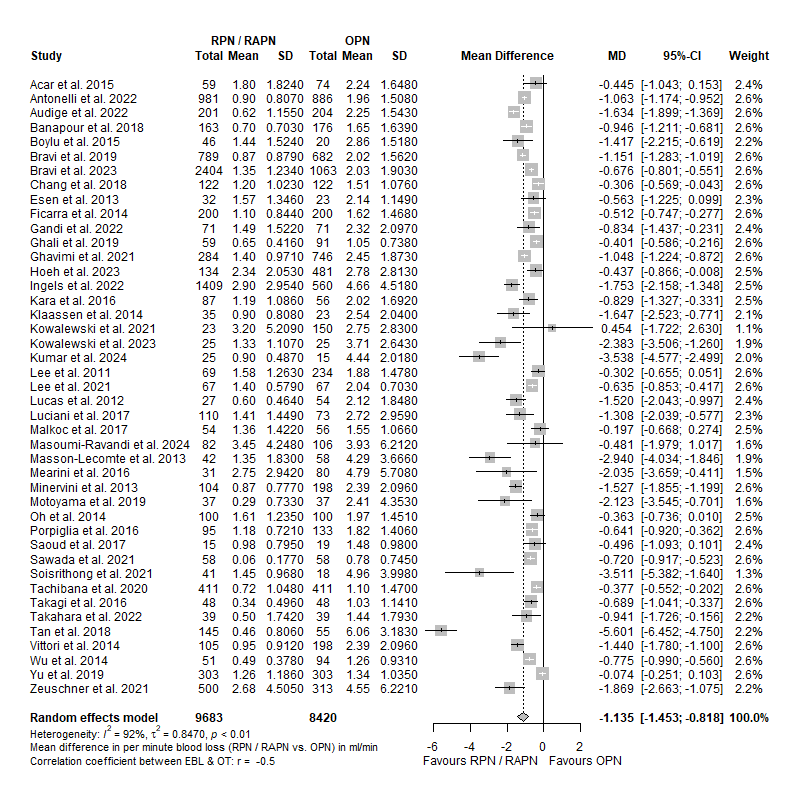

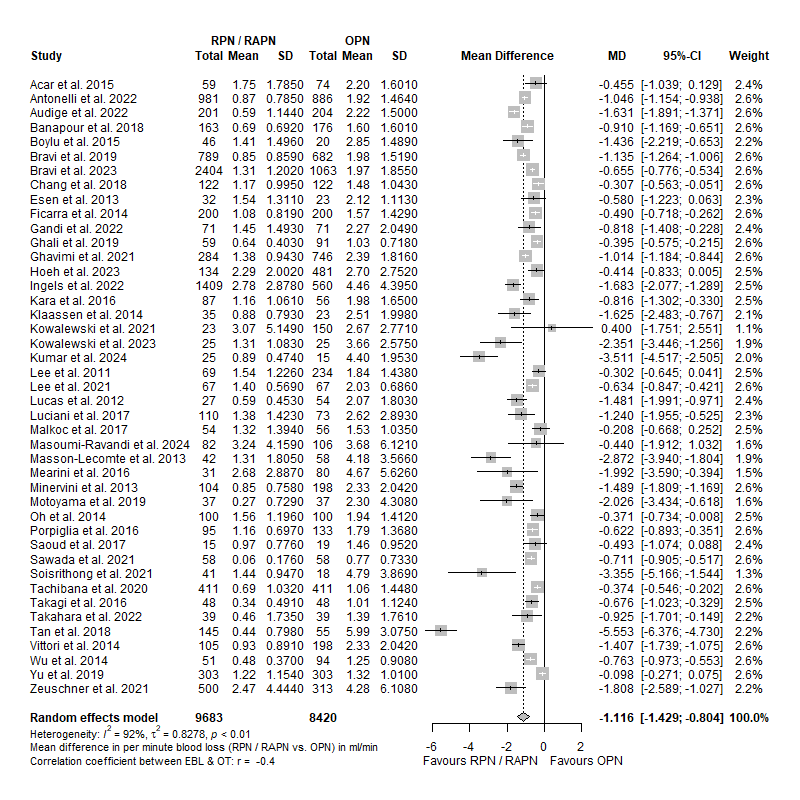

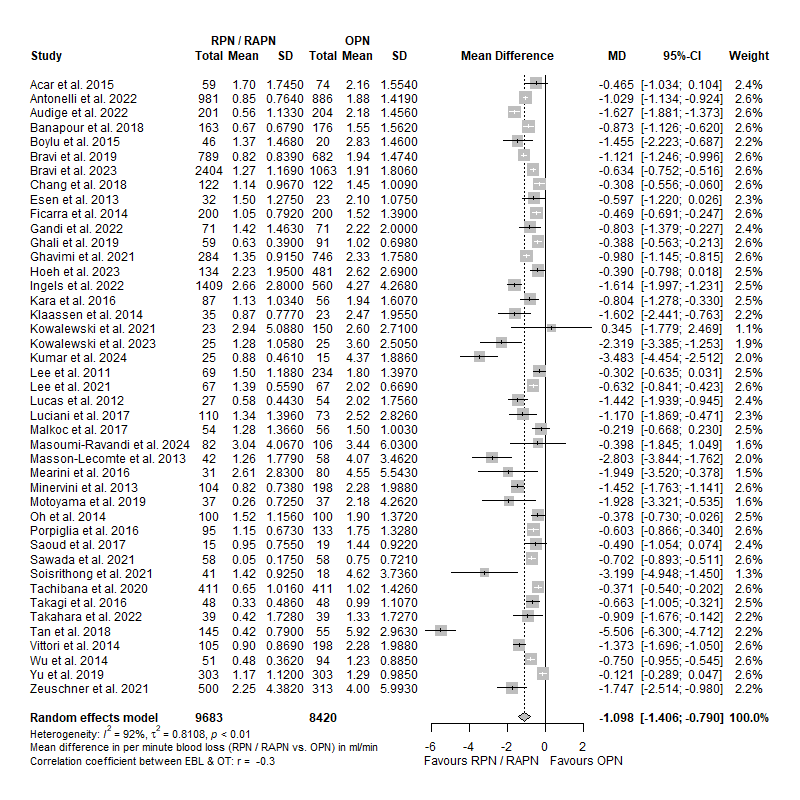

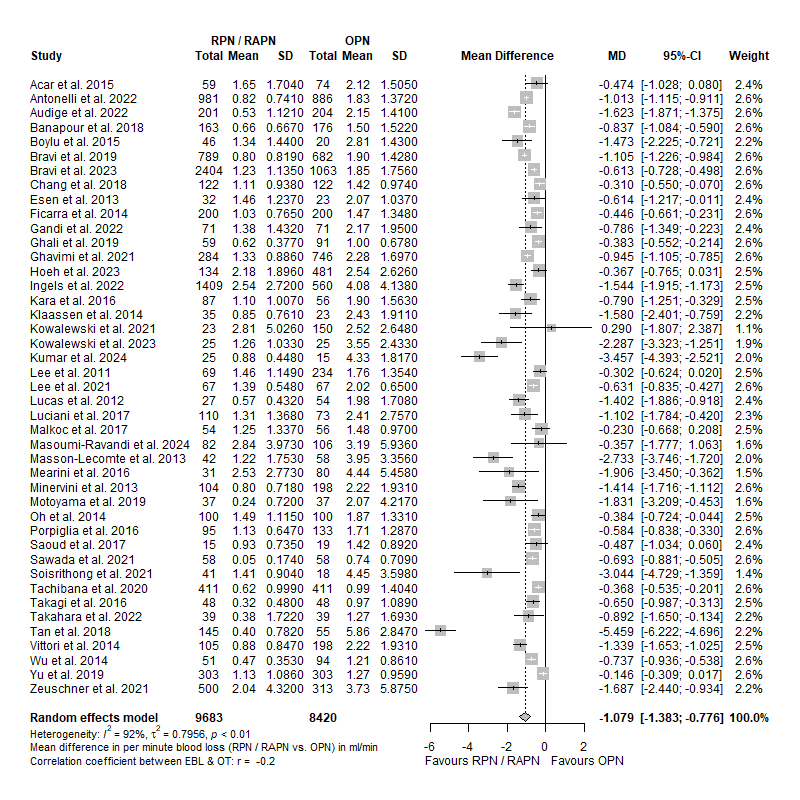

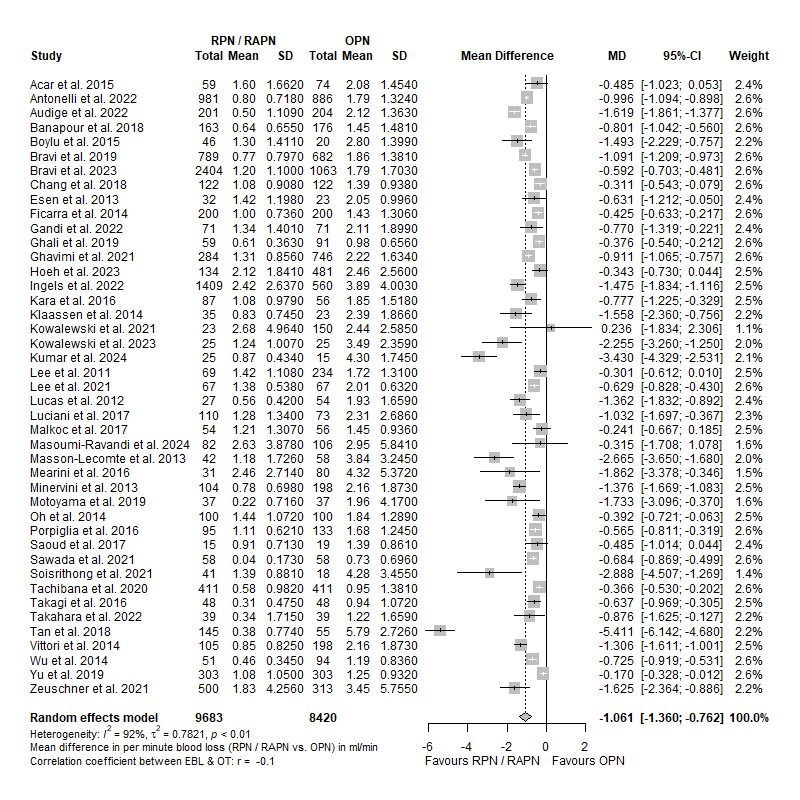

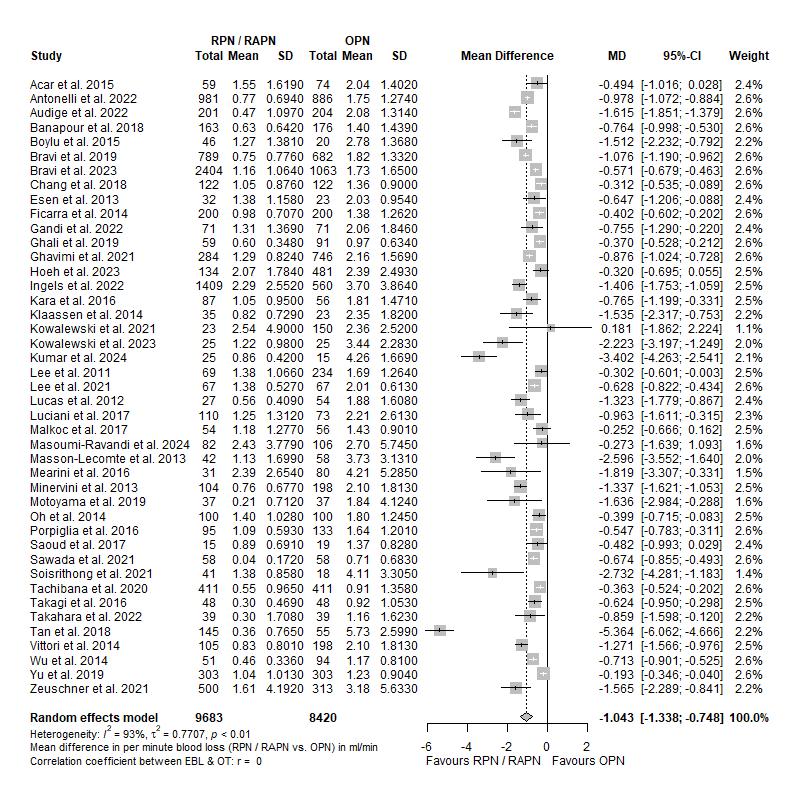

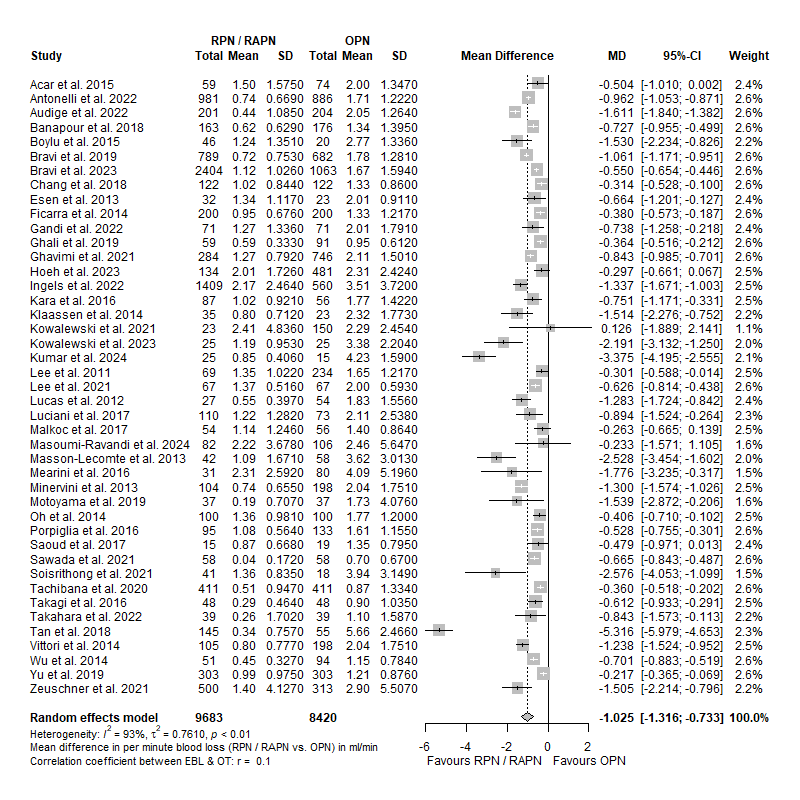

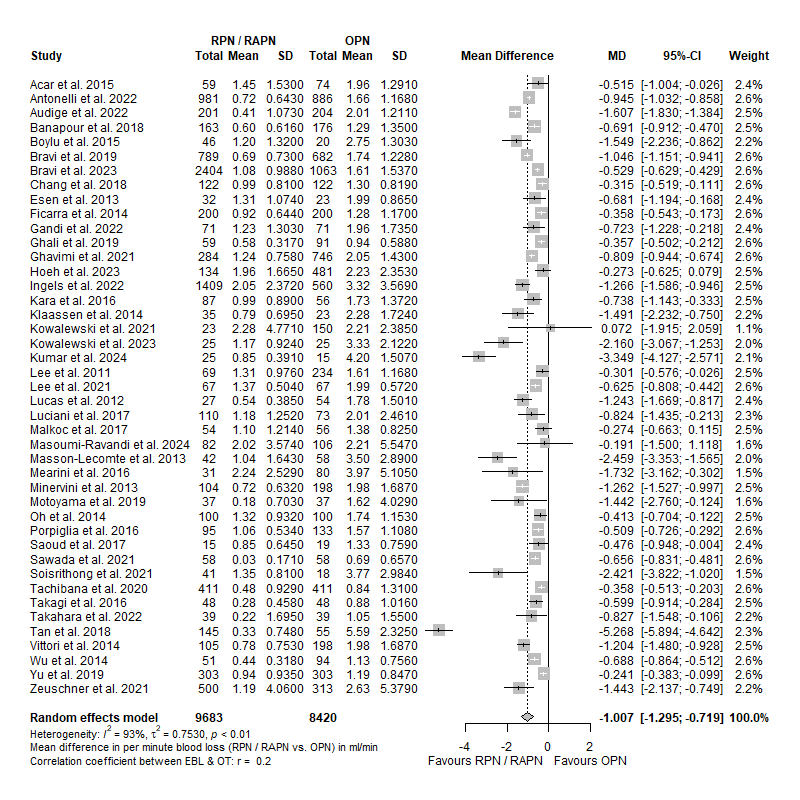

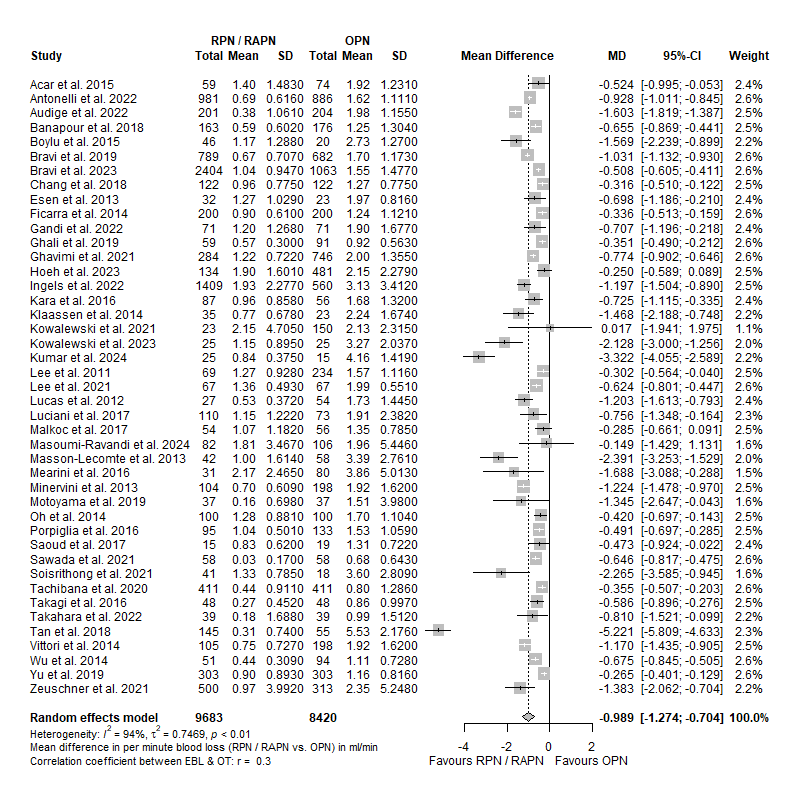

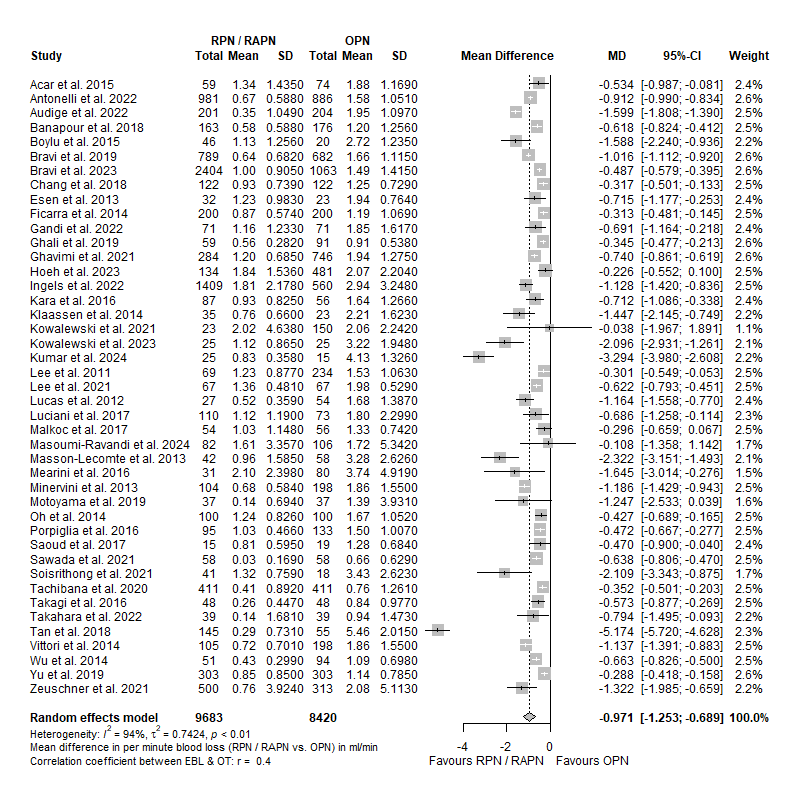

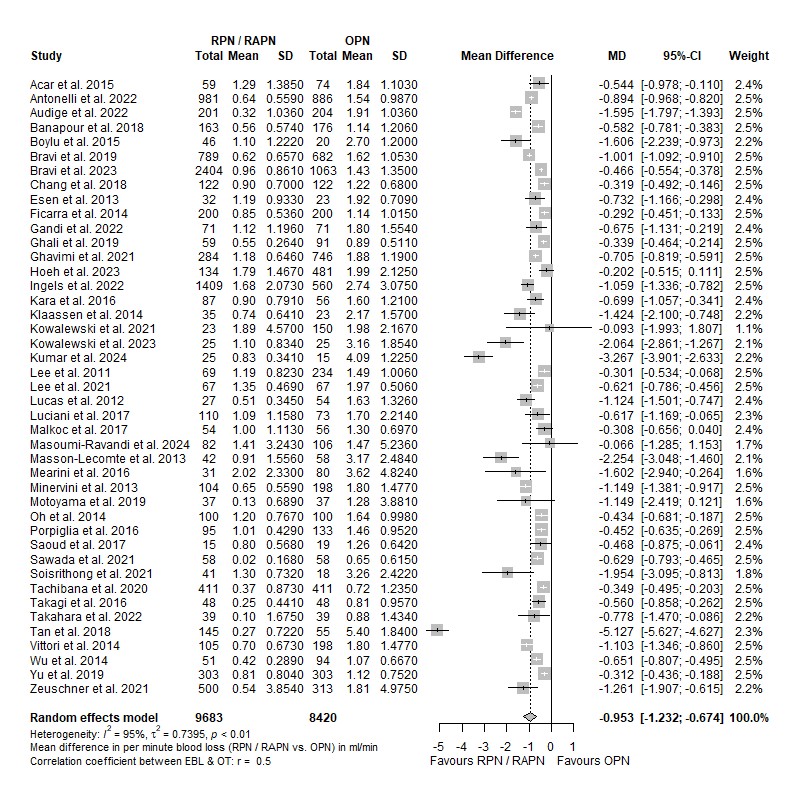

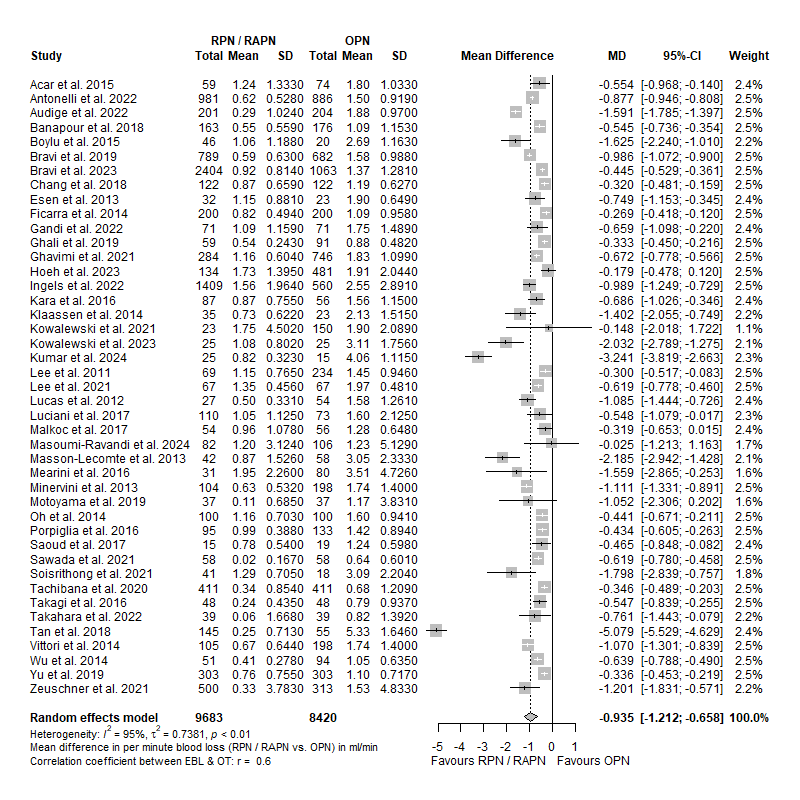

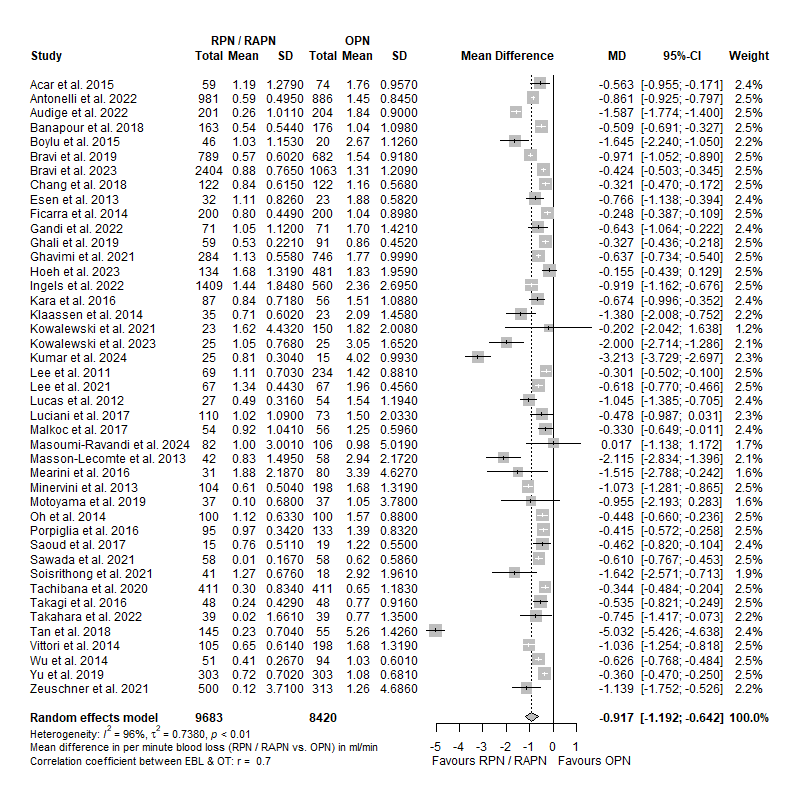

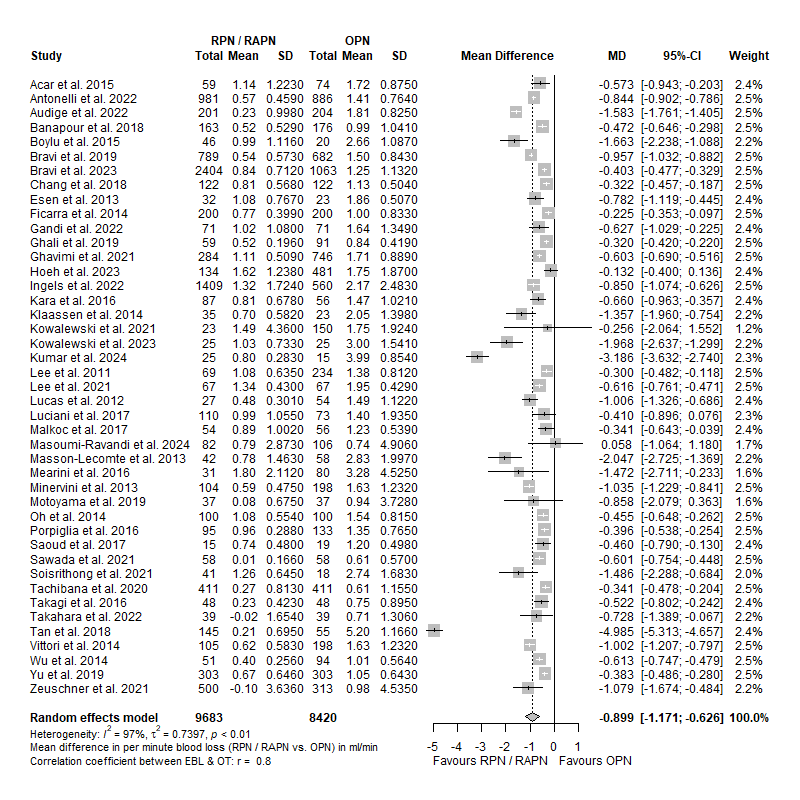

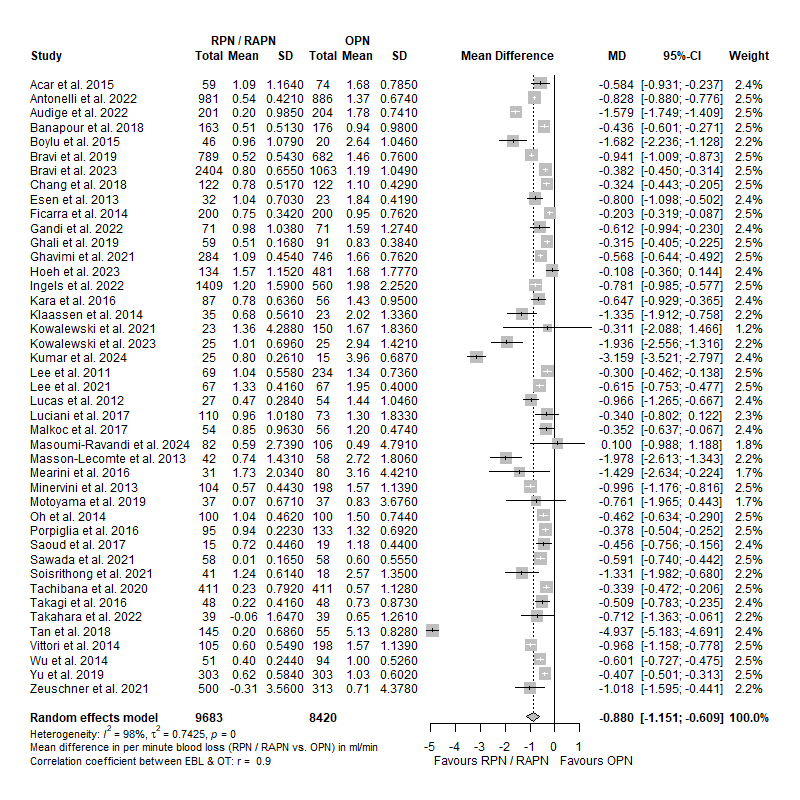

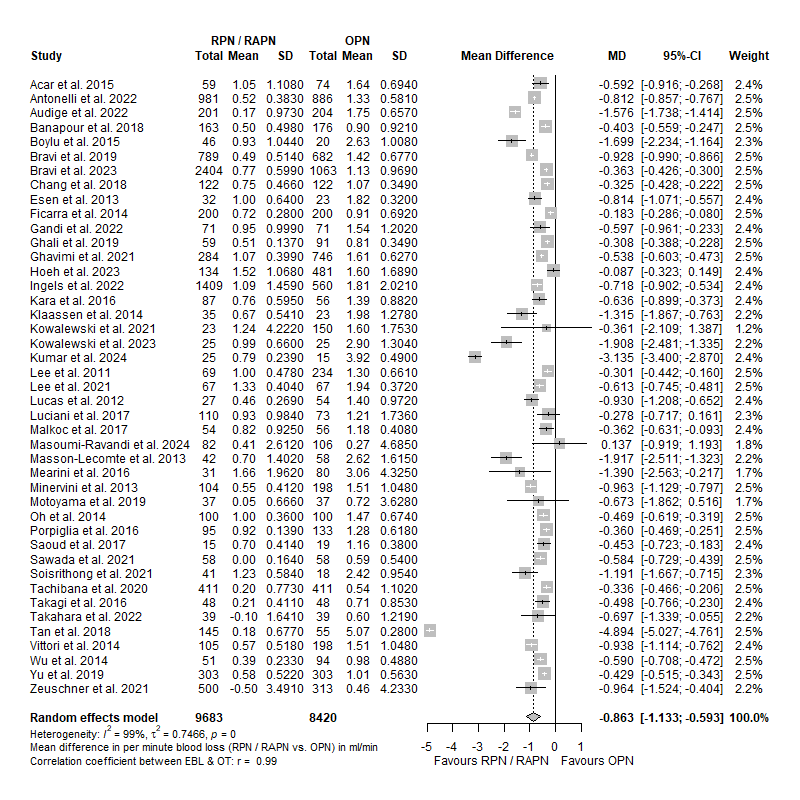
*

Supplementary SA Forest plots: Successive forest plots depicting the stepwise MD_Q_ assessment in pooled studies, for the gradual transition of r from -0.99 to +0.99. Abbreviations: EBL: estimated blood loss, OT: operative time, r: Pearson’s correlation coefficient between EBL and OT.

SENSITIVITY ANALYSIS FOREST PLOTS

(SUBGROUPS BY PUBLICATION YEAR)

*
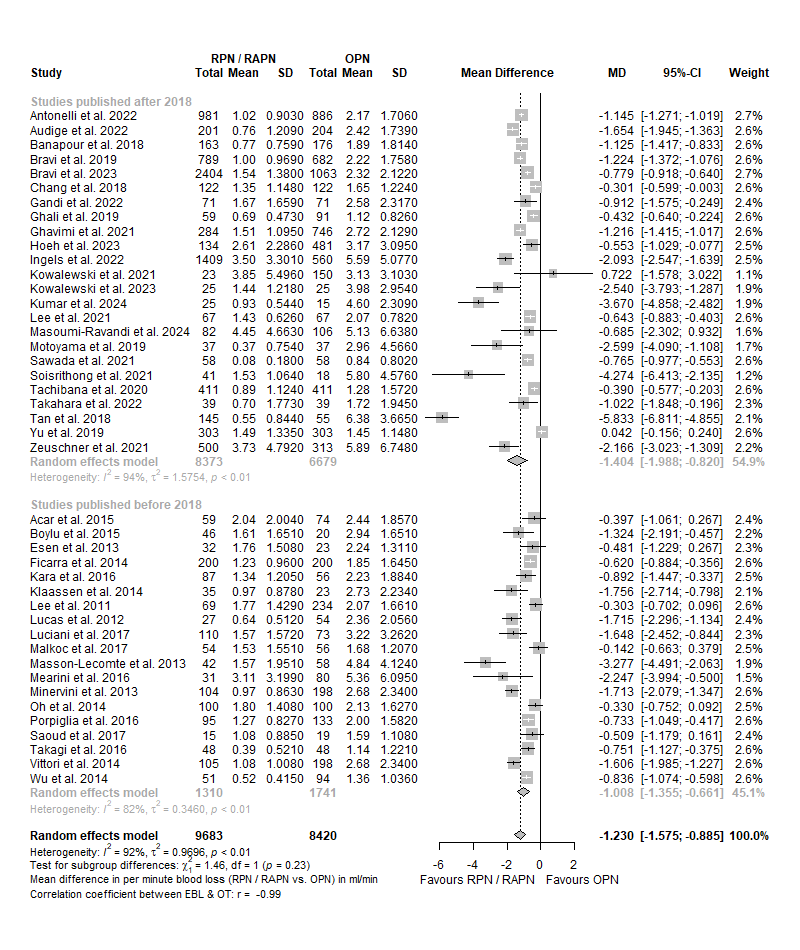

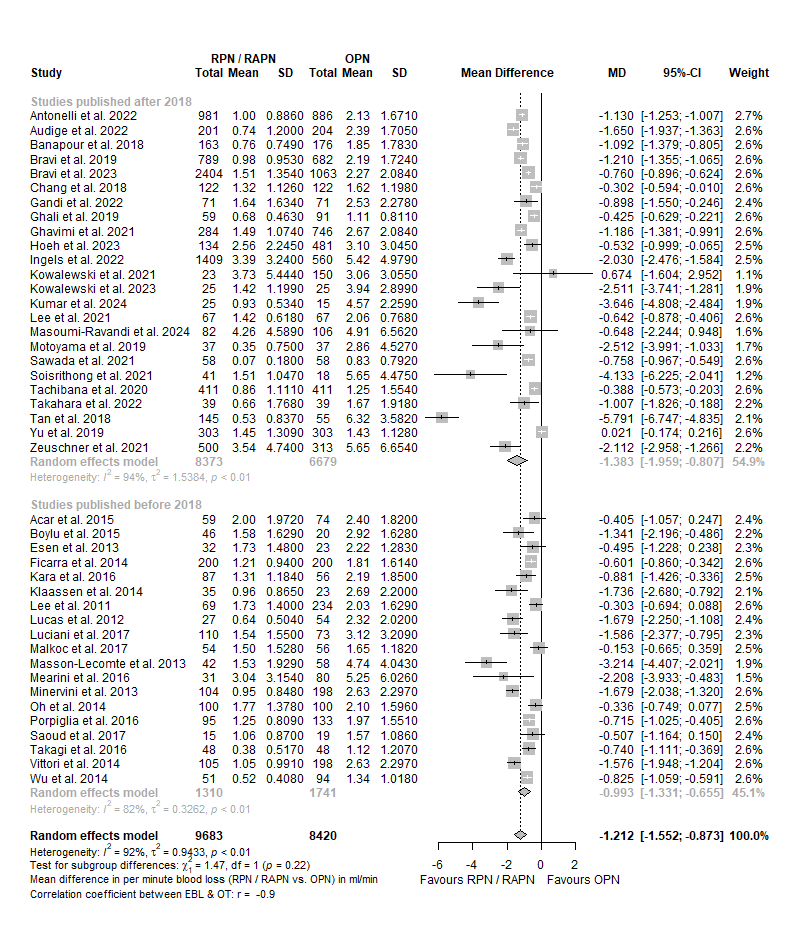

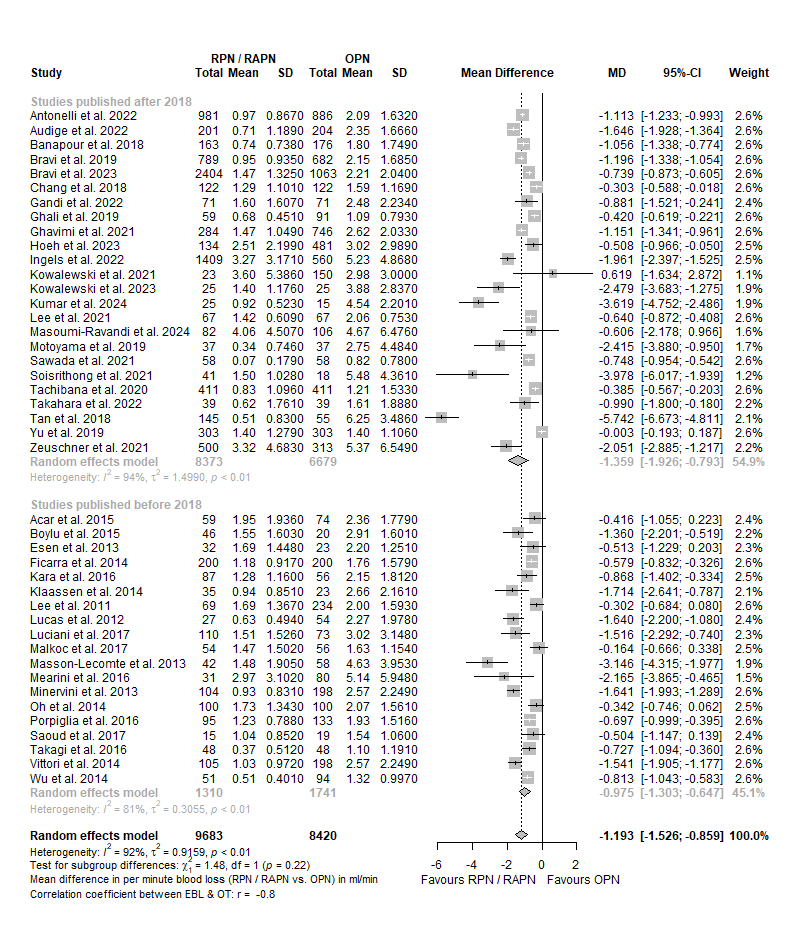

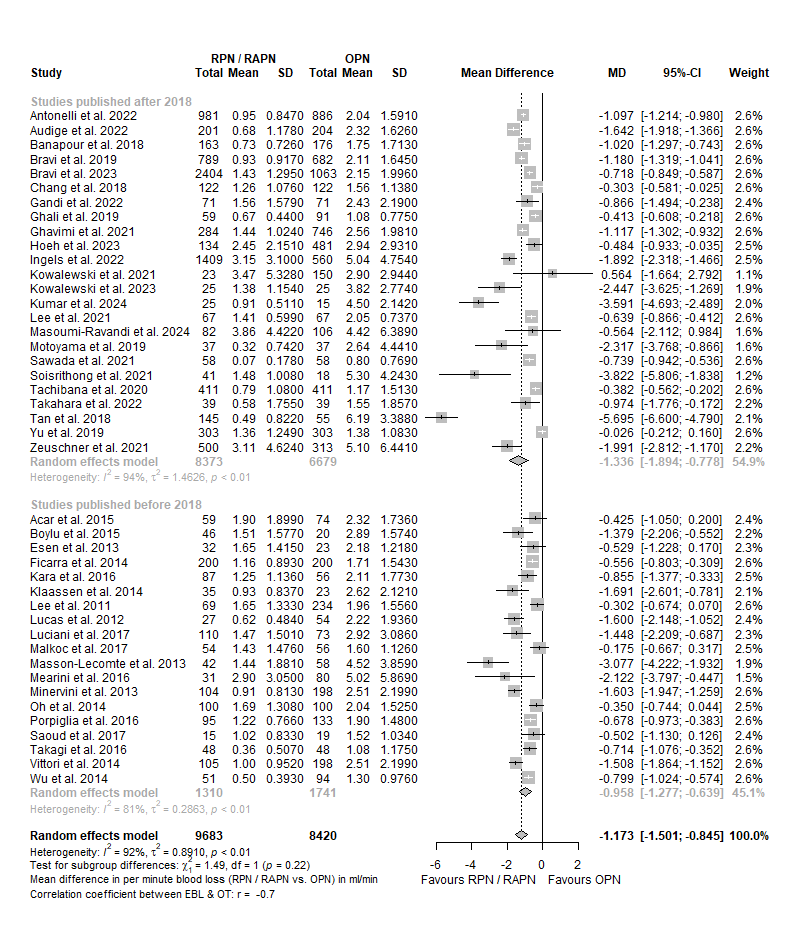

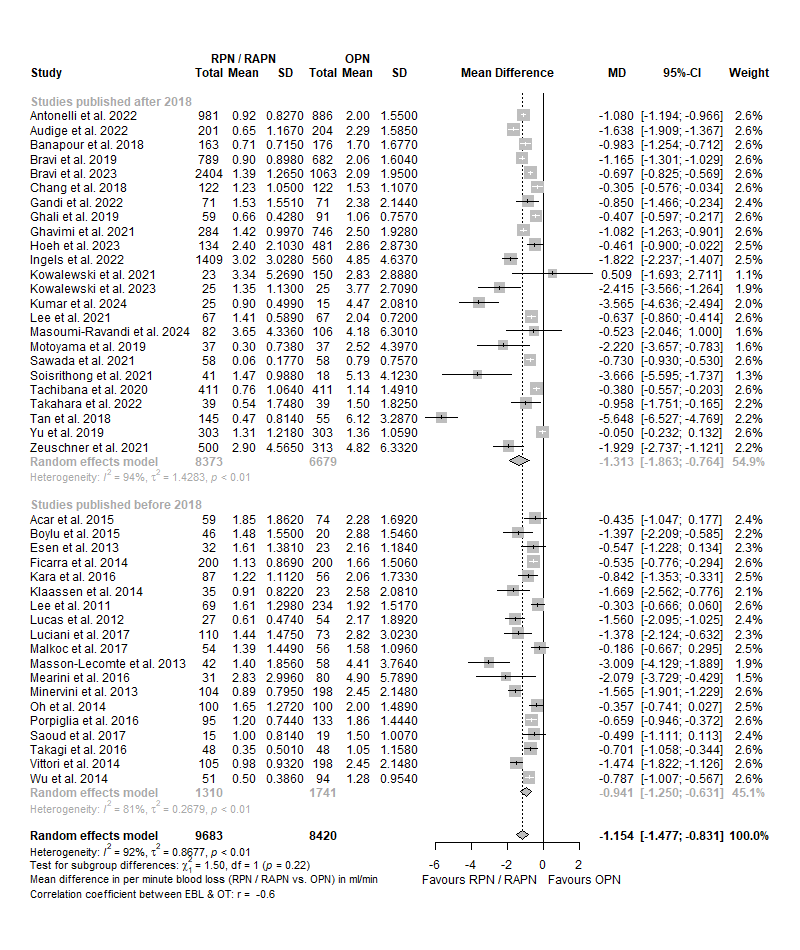

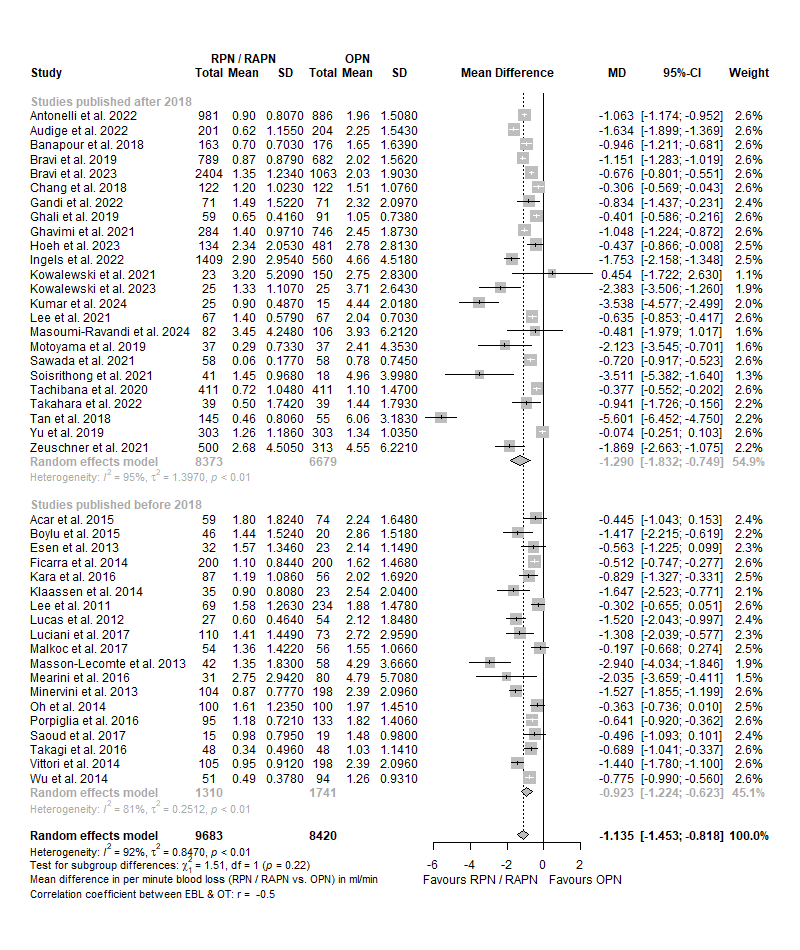

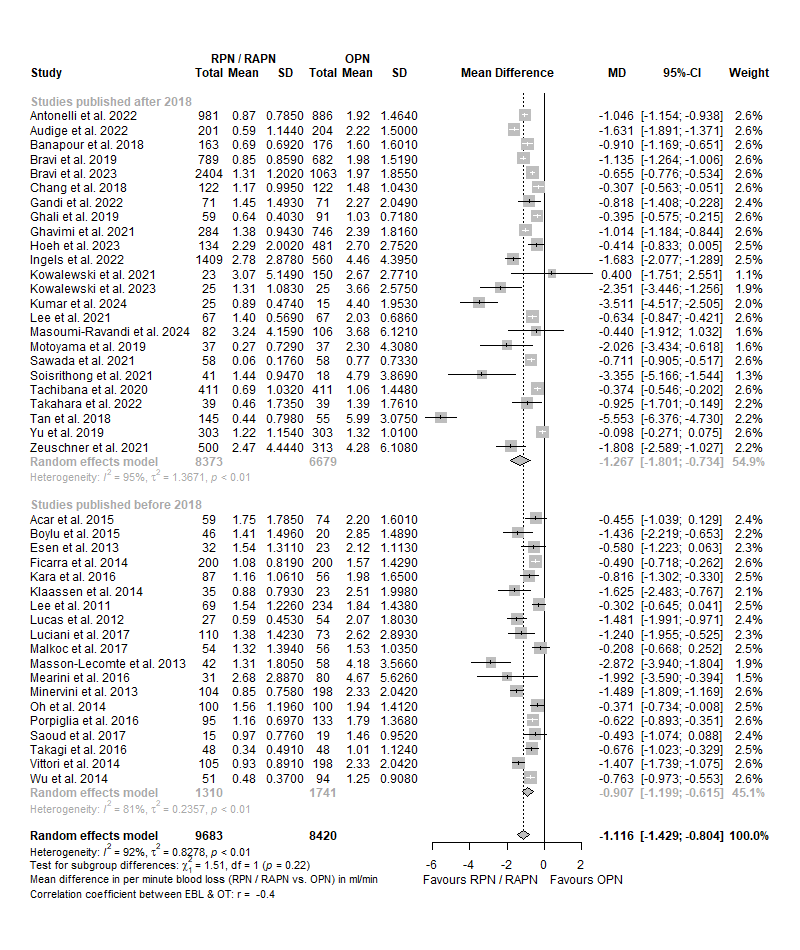

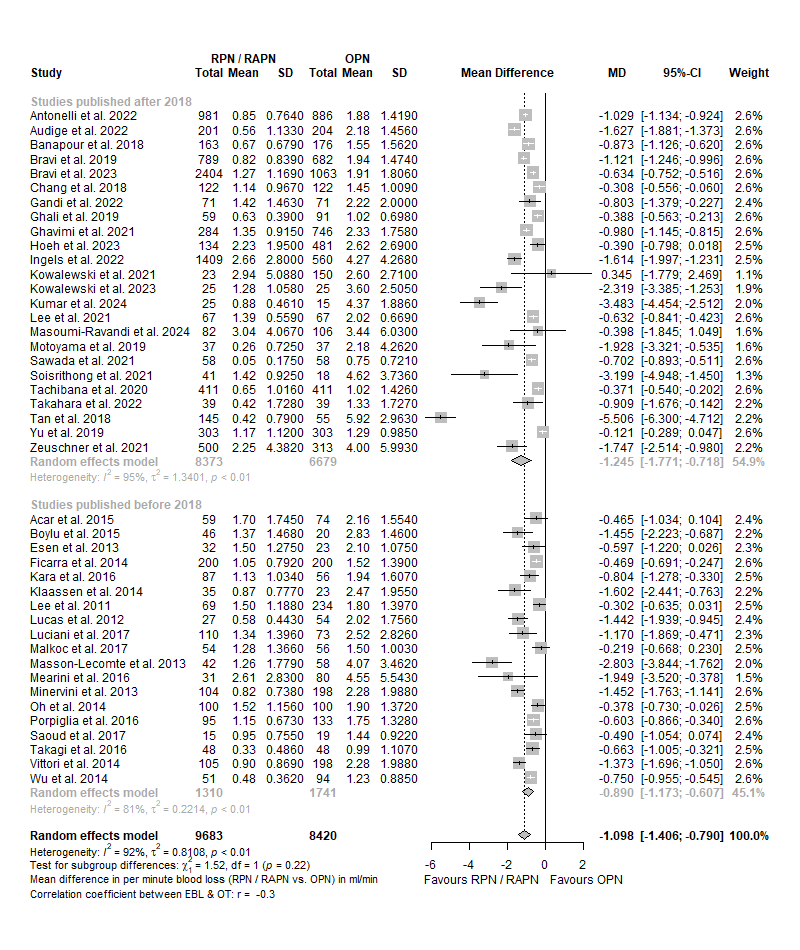

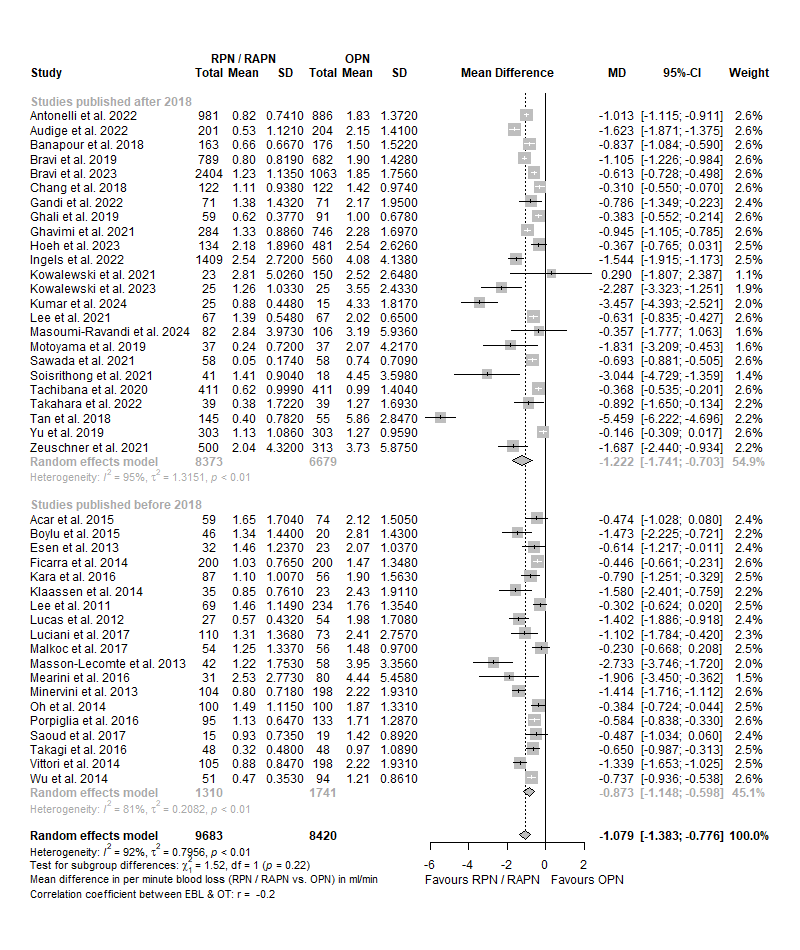

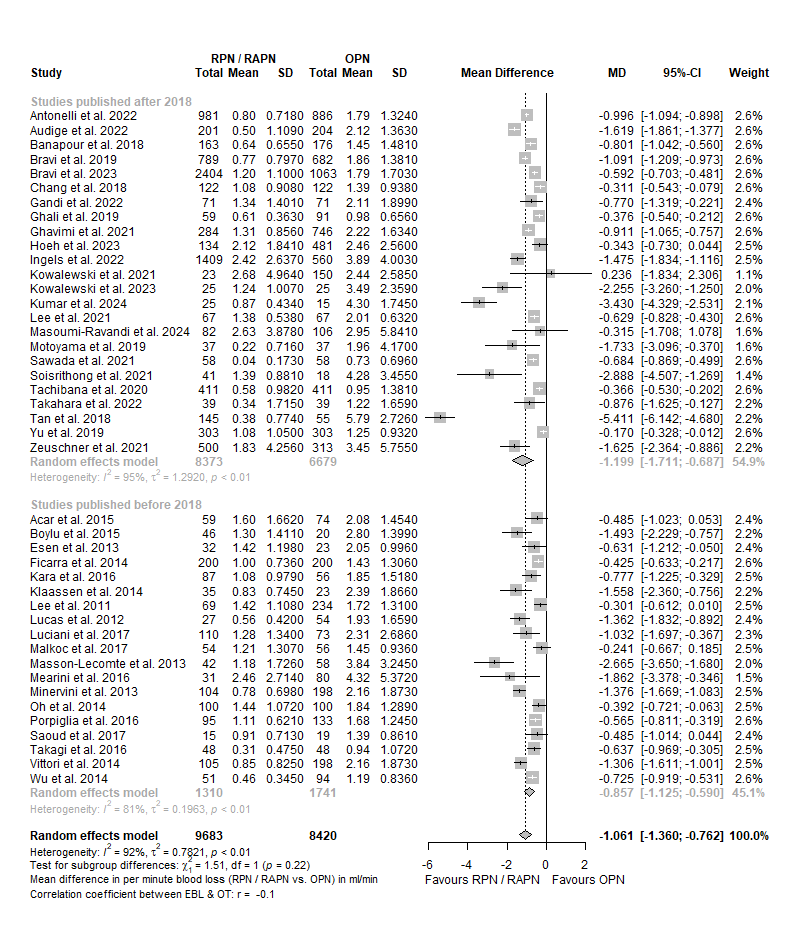

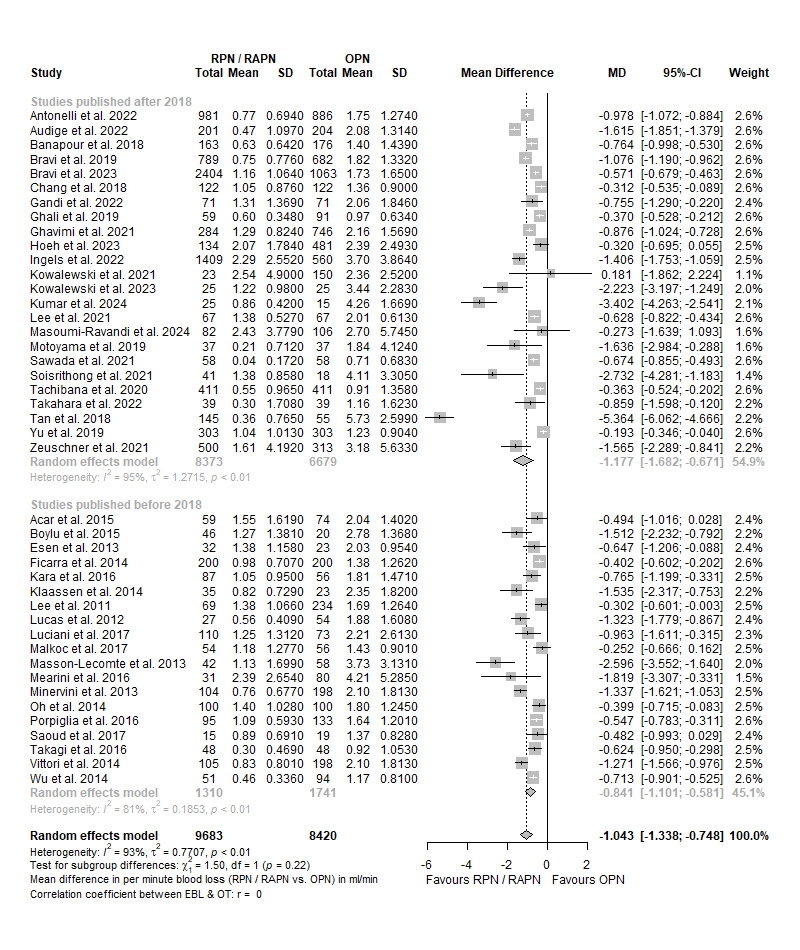

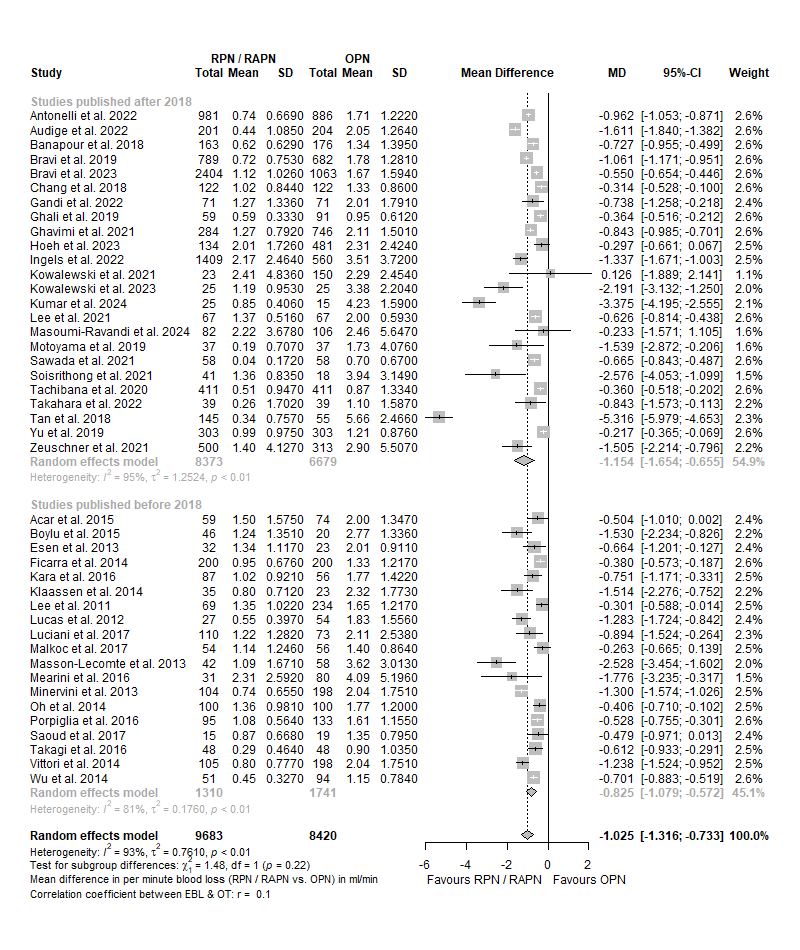

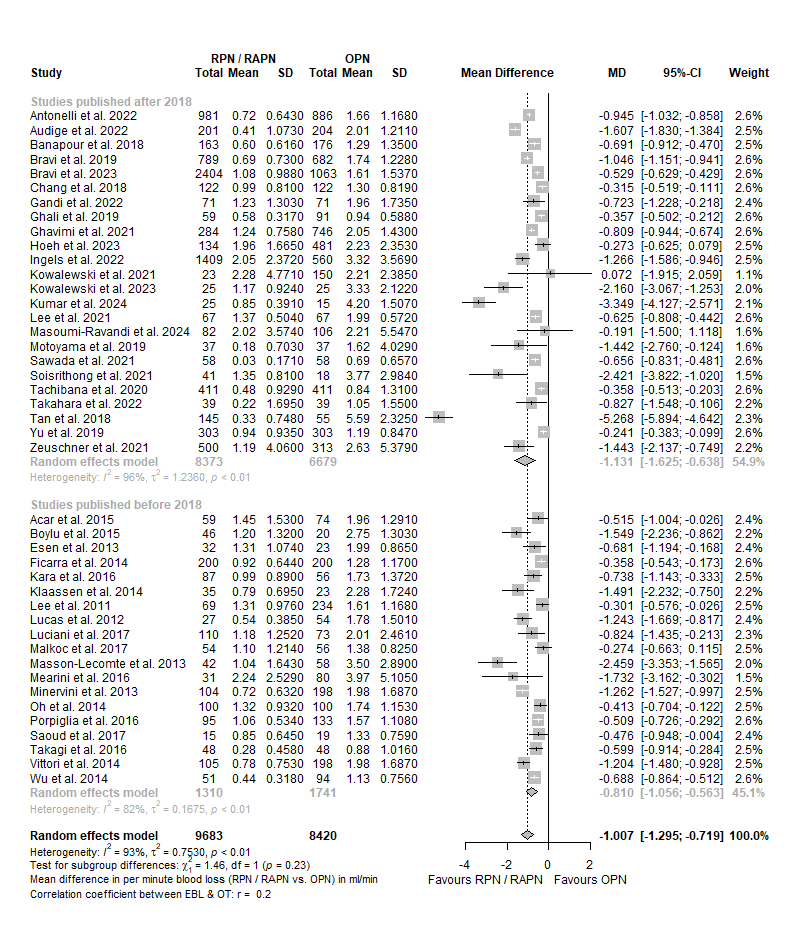

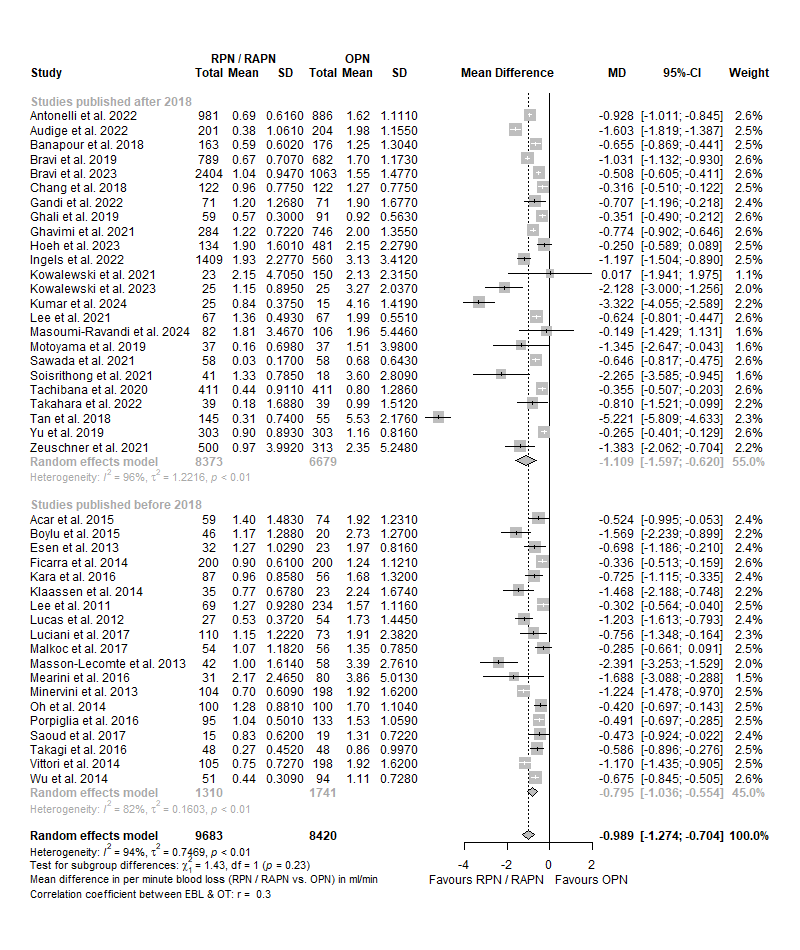

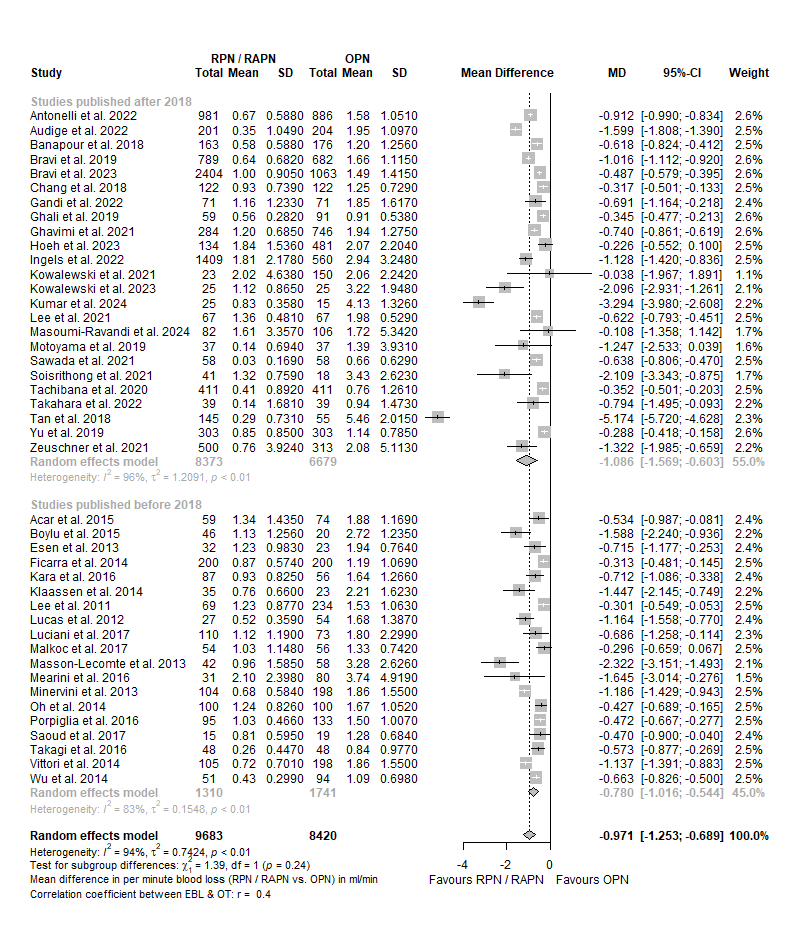

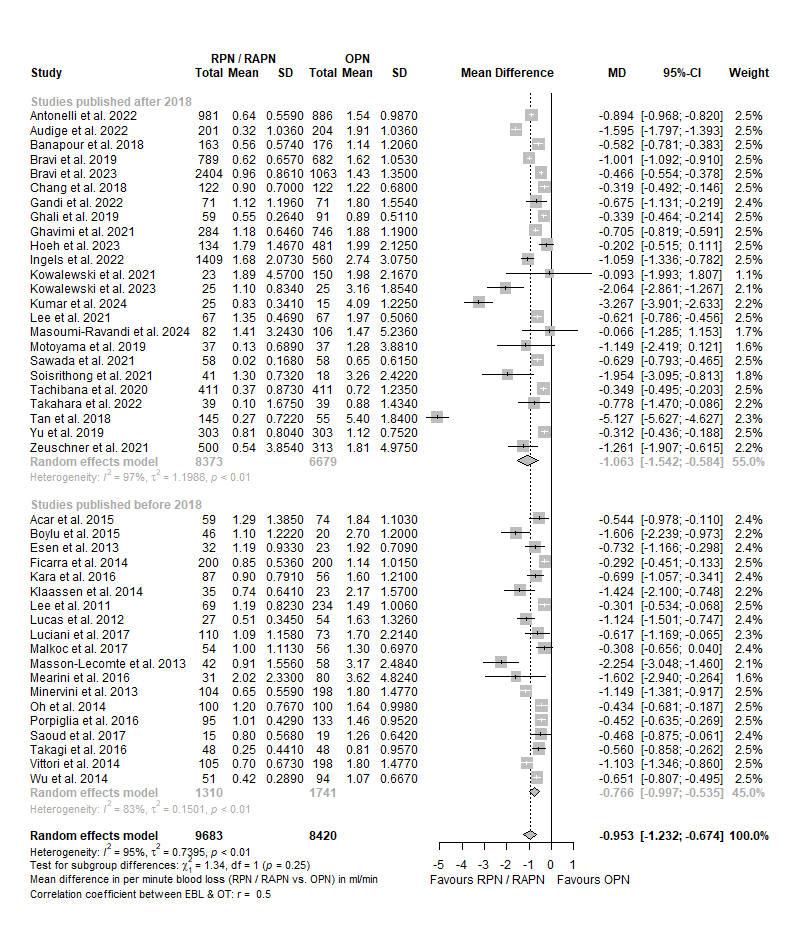

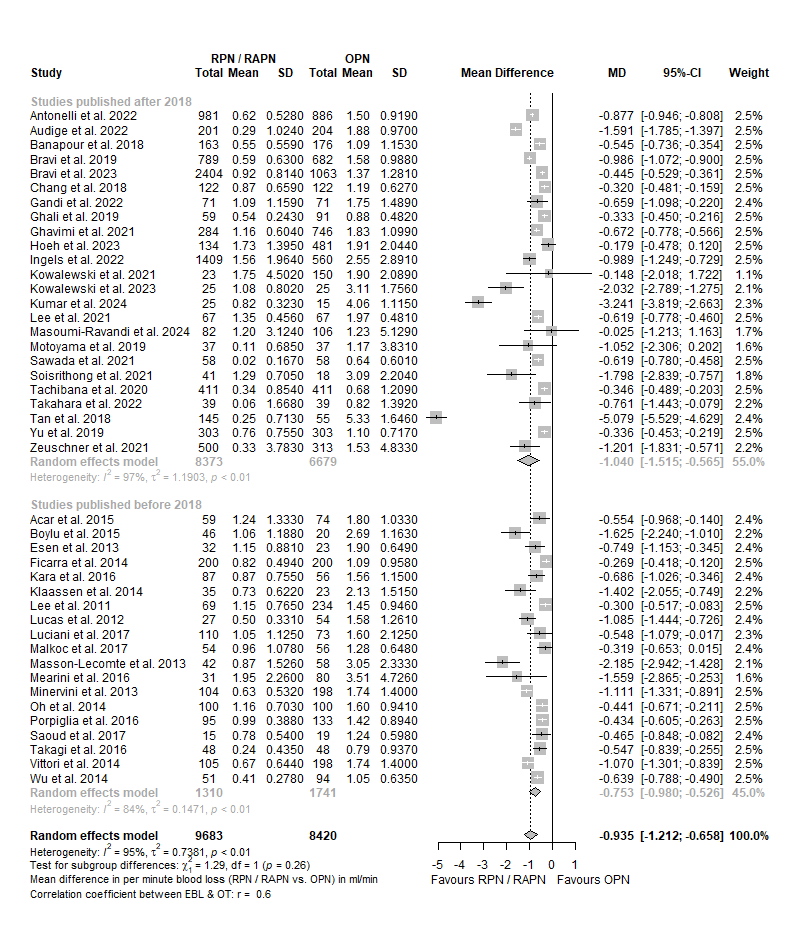

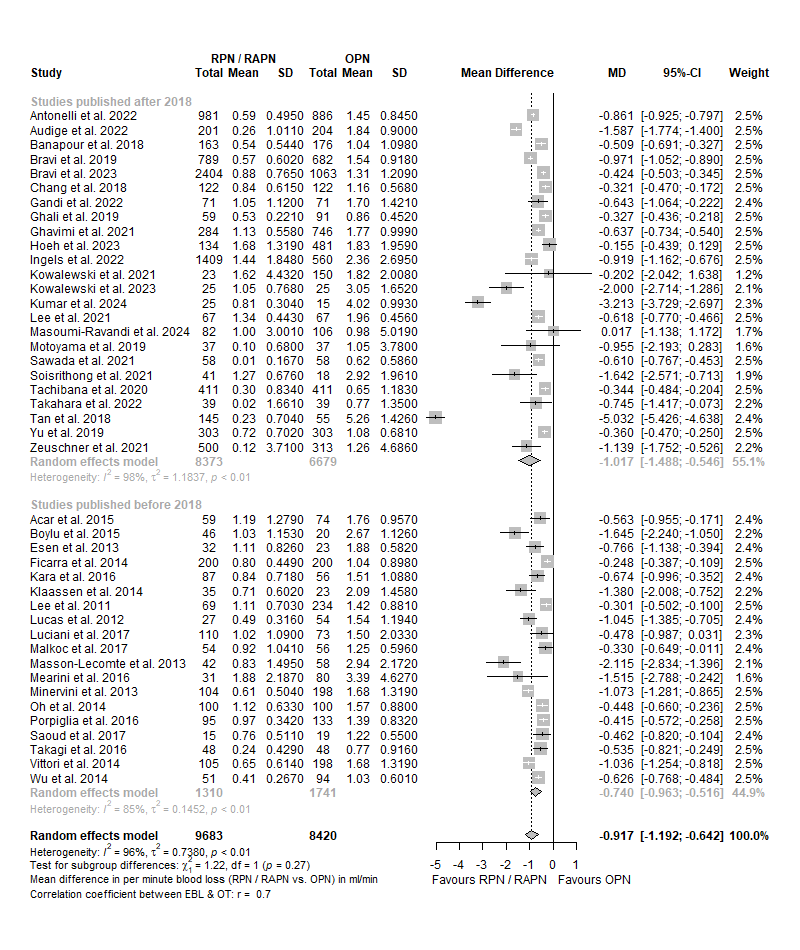

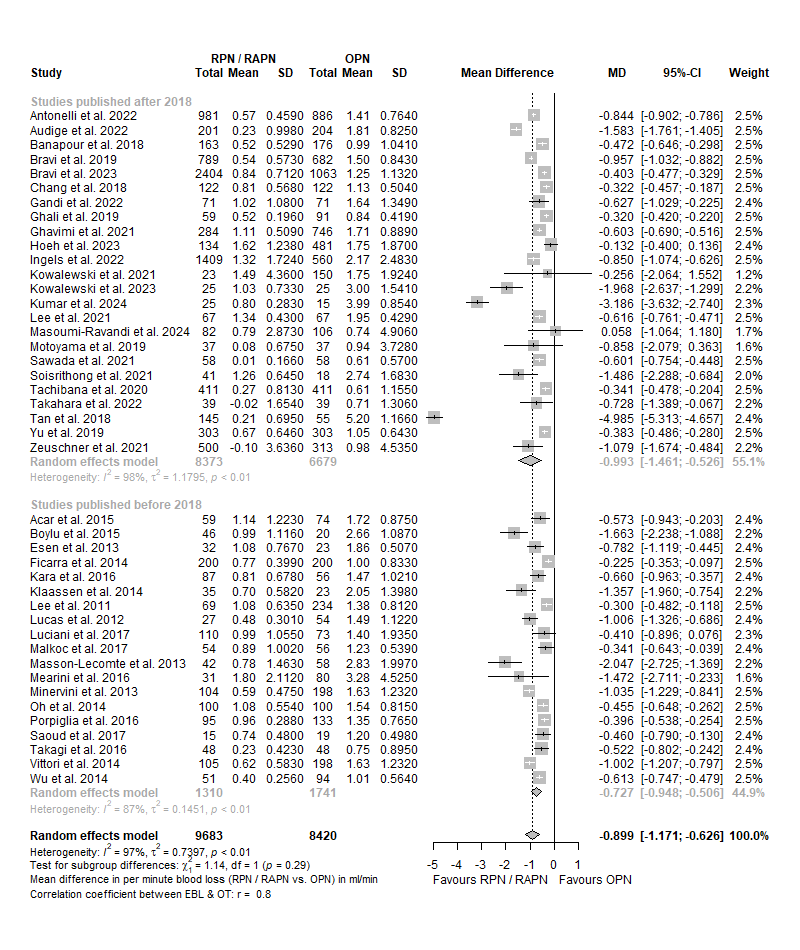

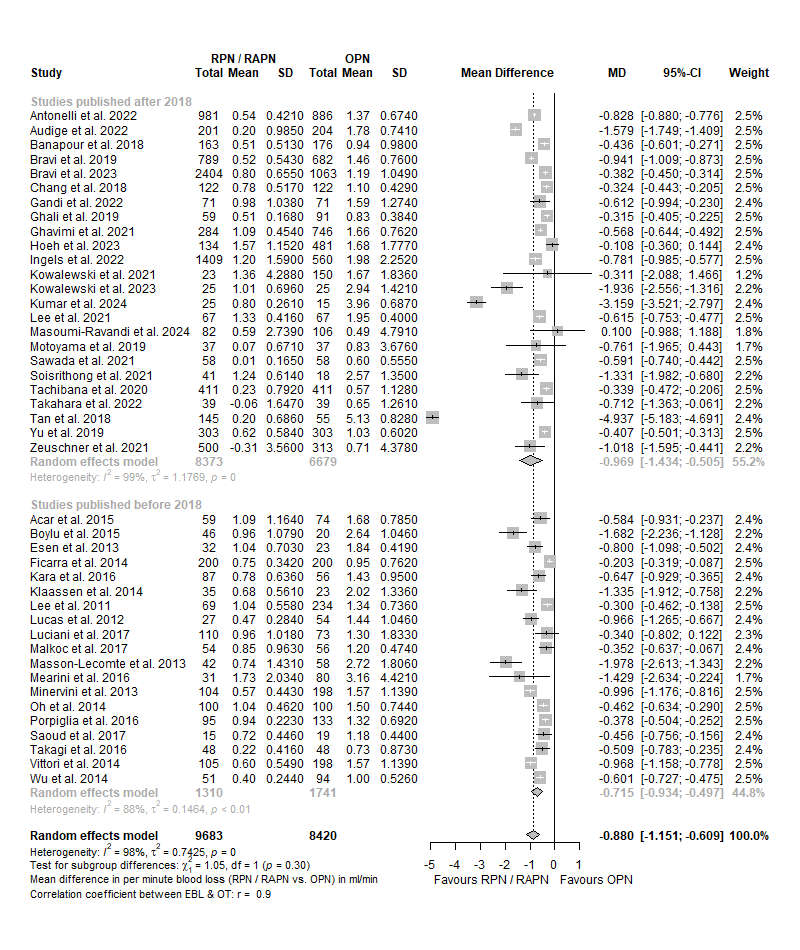

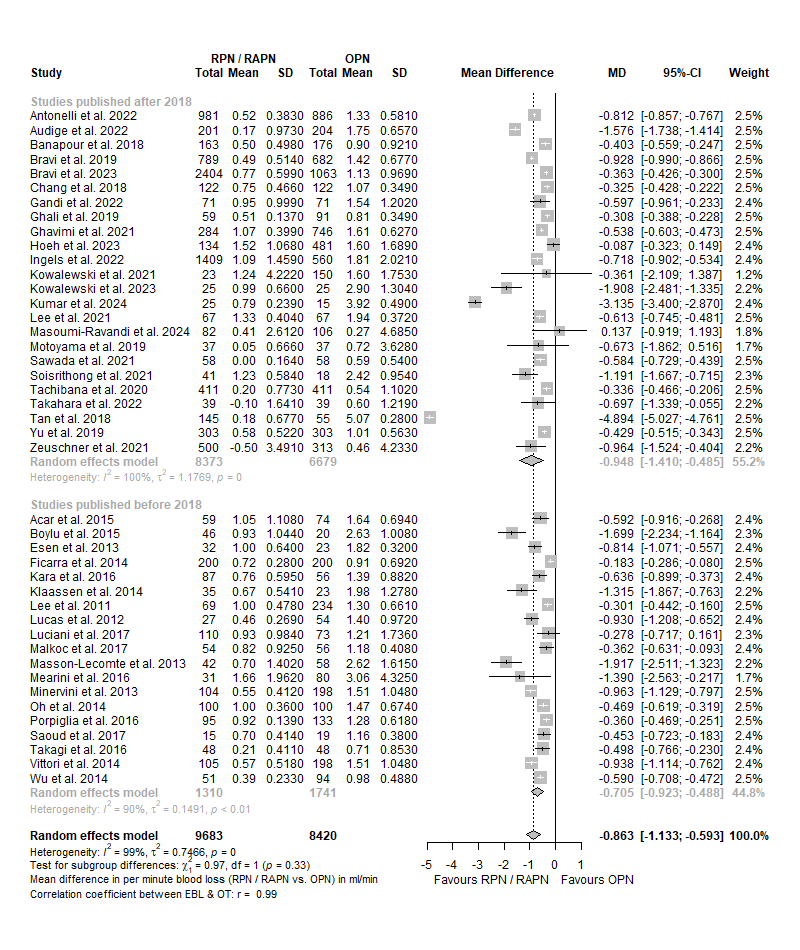
*

Supplementary SA Forest plots: Successive forest plots depicting the stepwise MD_Q_ assessment in studies published post- or pre-2018, for the gradual transition of r from -0.99 to +0.99. Abbreviations: EBL: estimated blood loss, OT: operative time, r: Pearson’s correlation coefficient between EBL and OT.

SENSITIVITY ANALYSIS FOREST PLOTS

(SUBGROUPS BY PATIENT MATCHING)

*
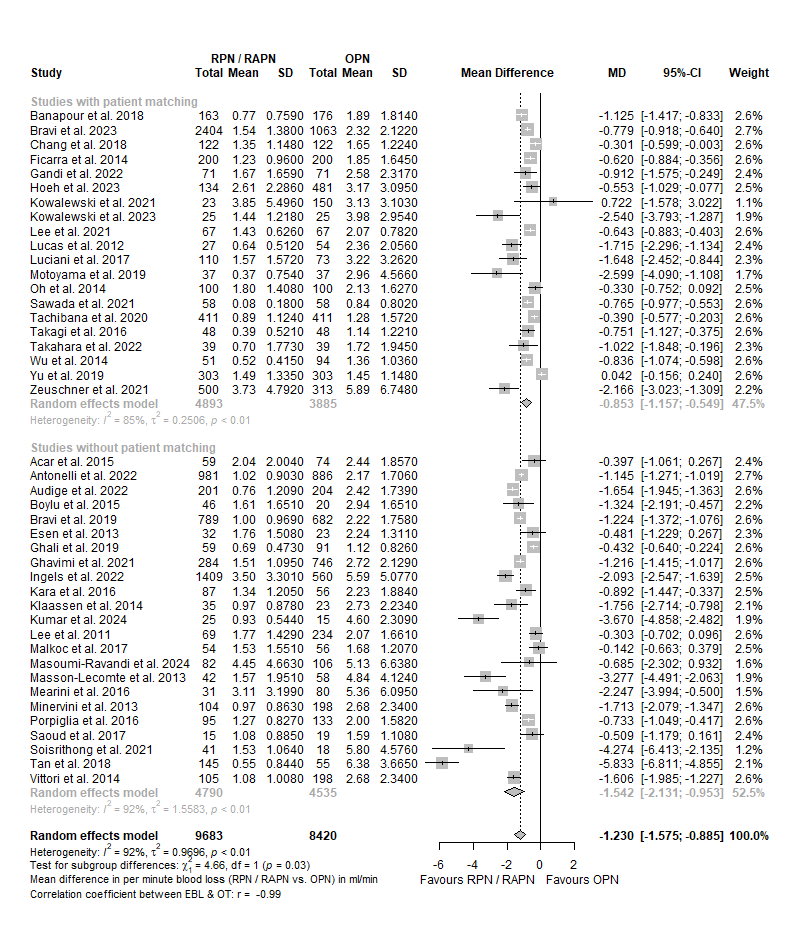

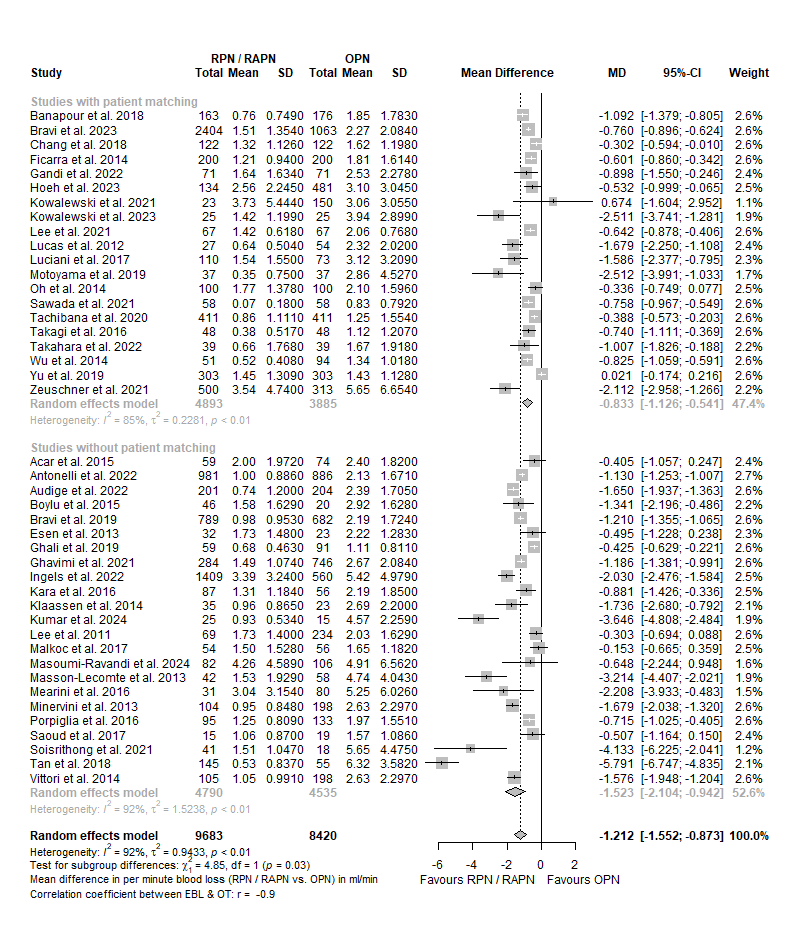

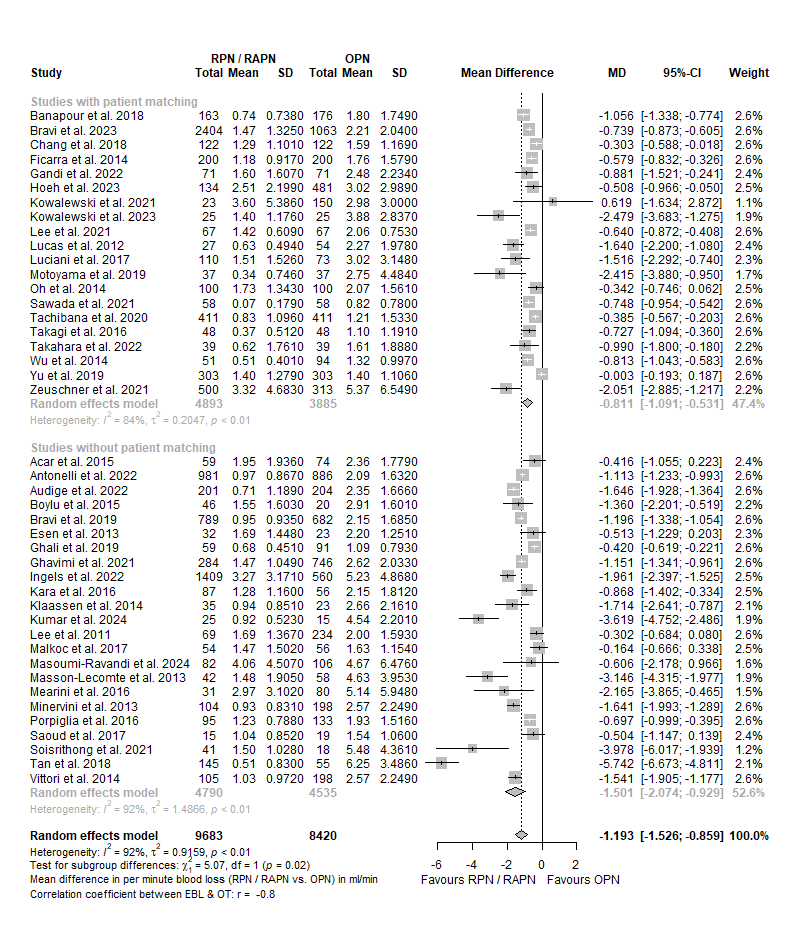

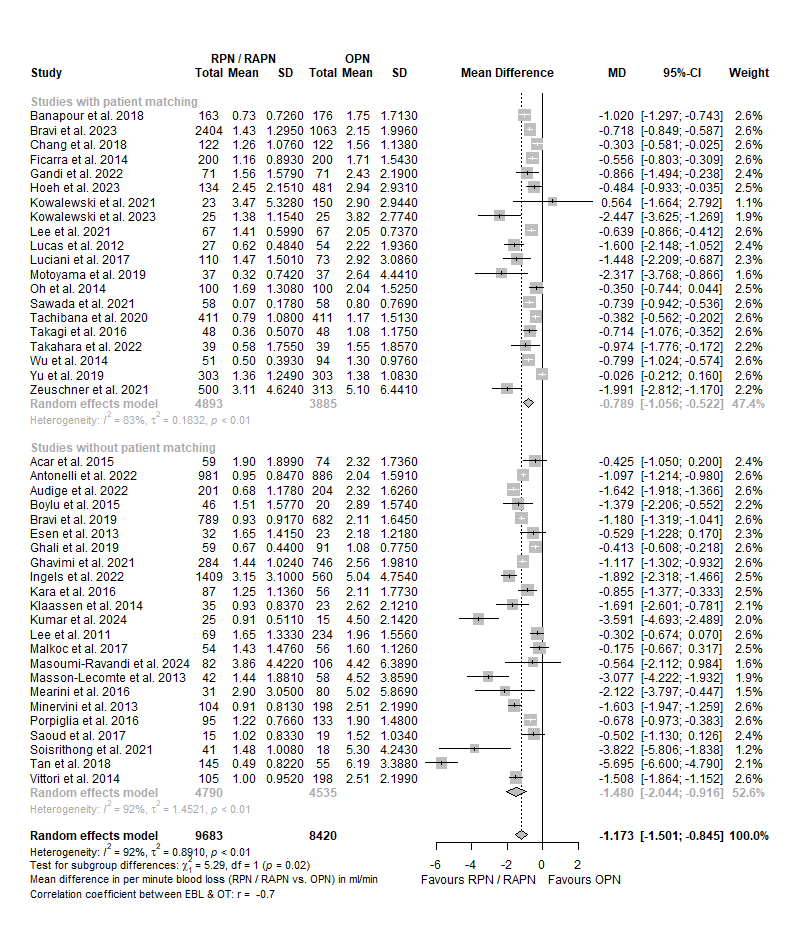

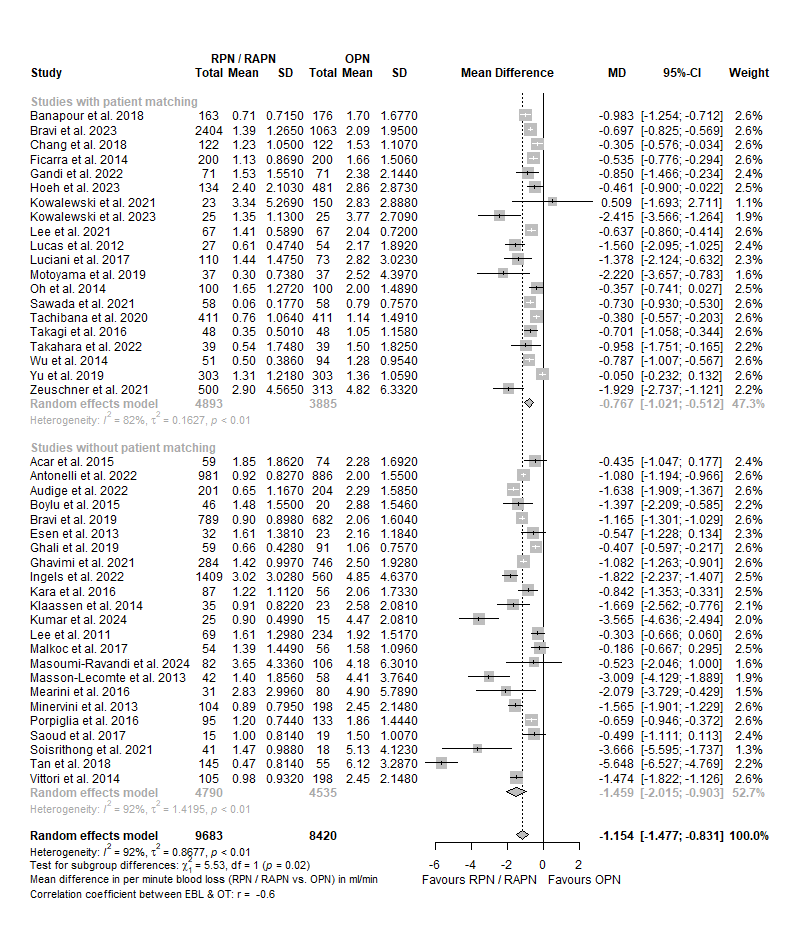

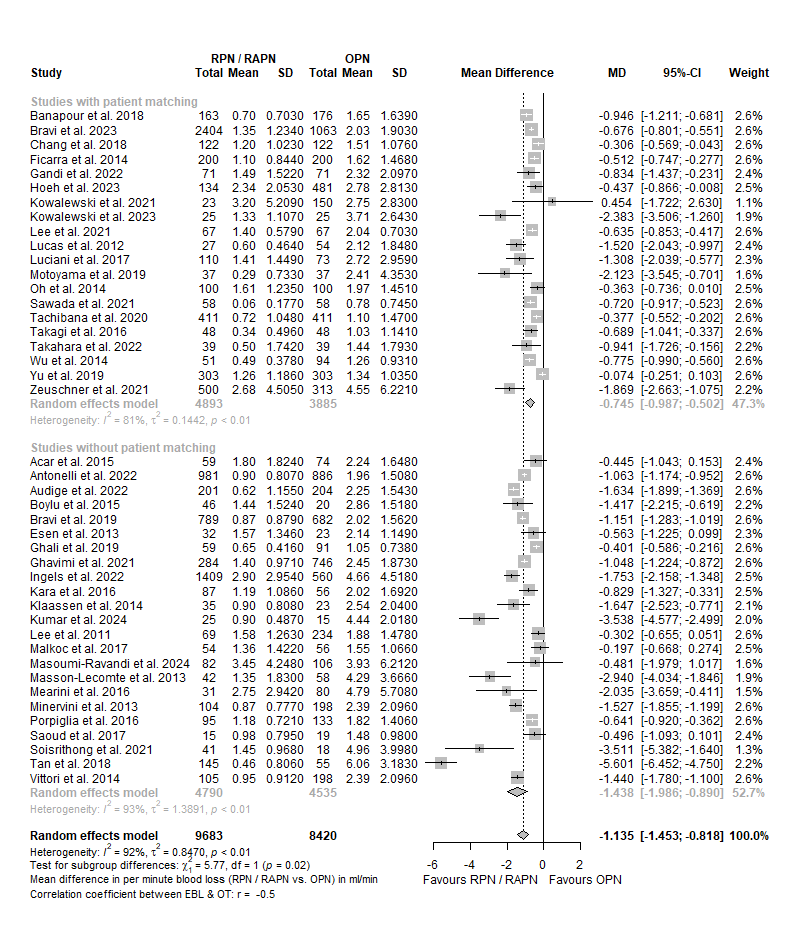

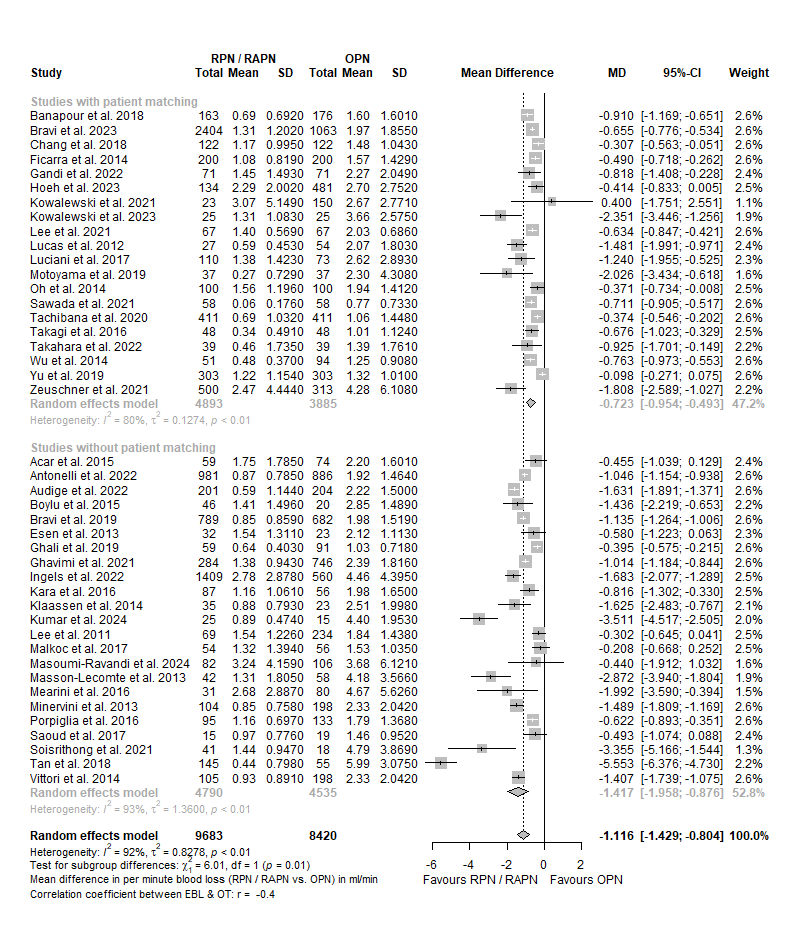

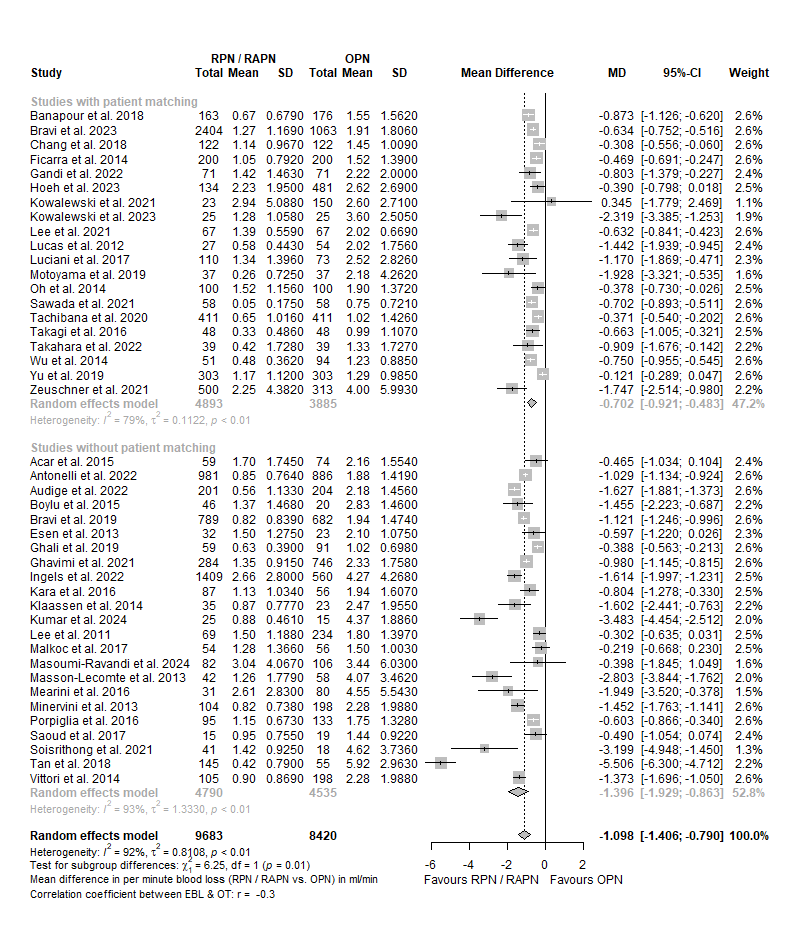

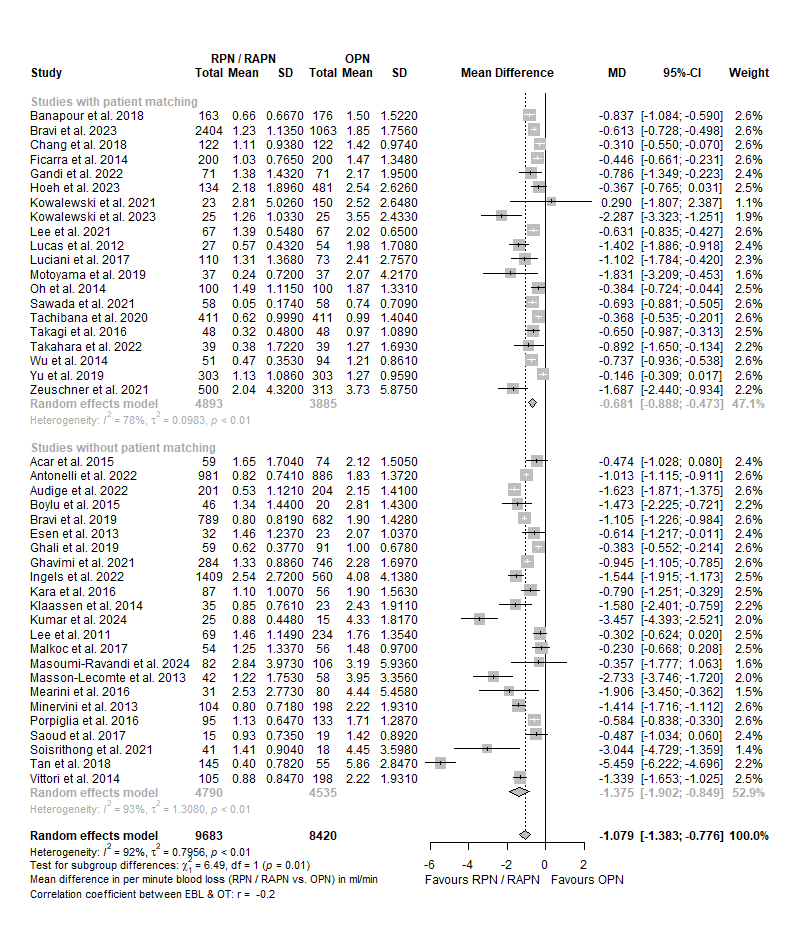

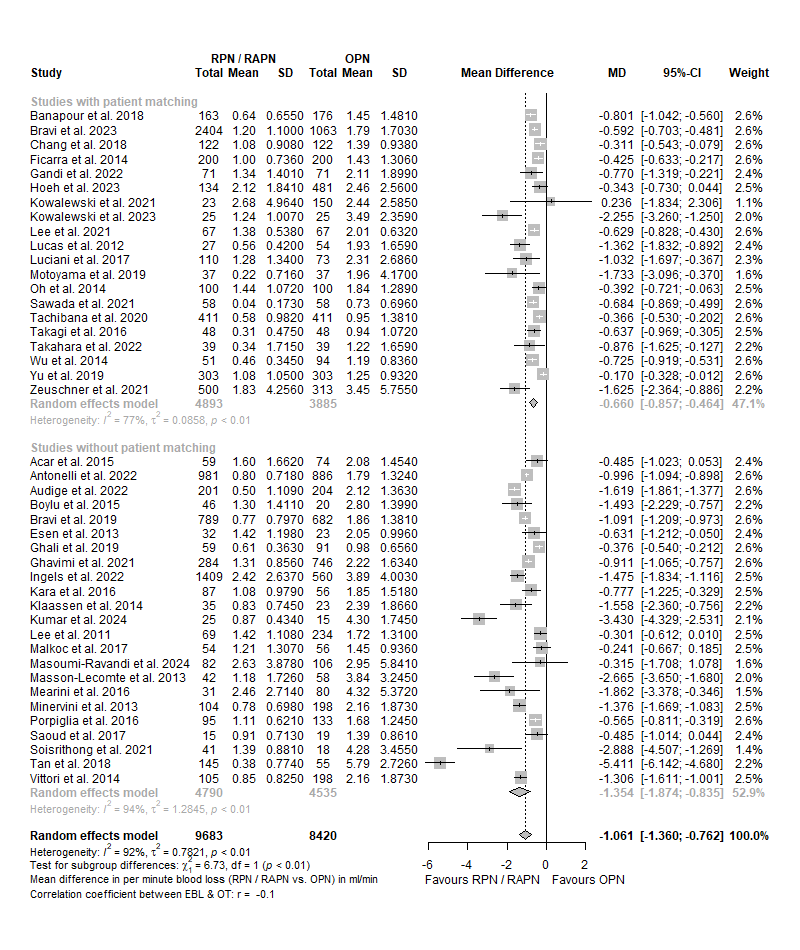

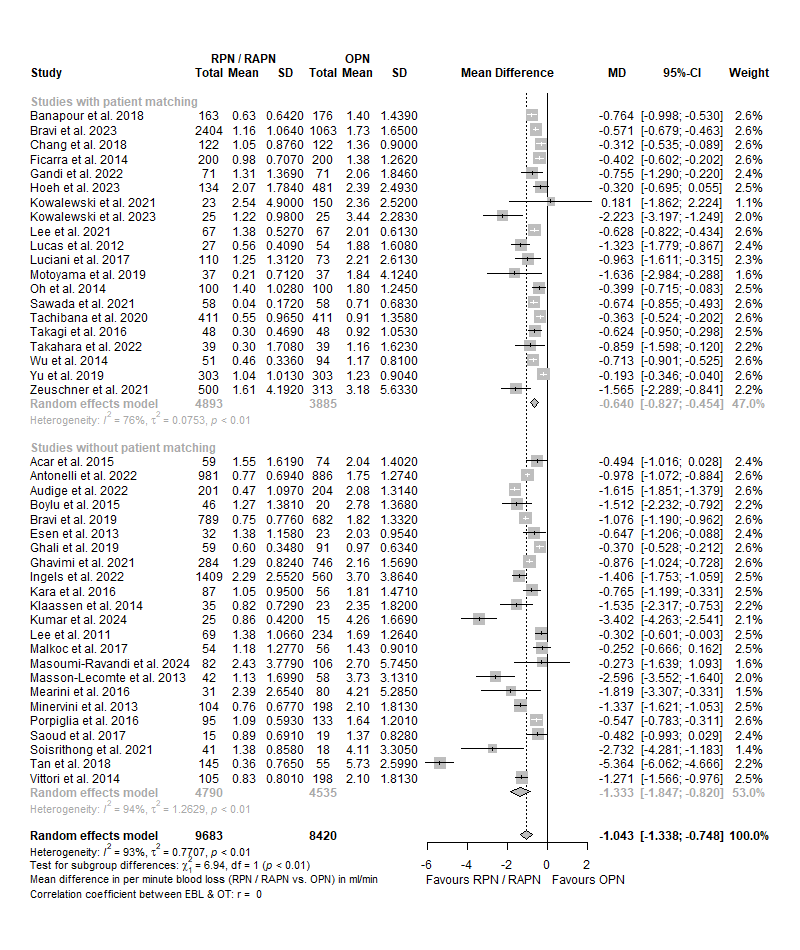

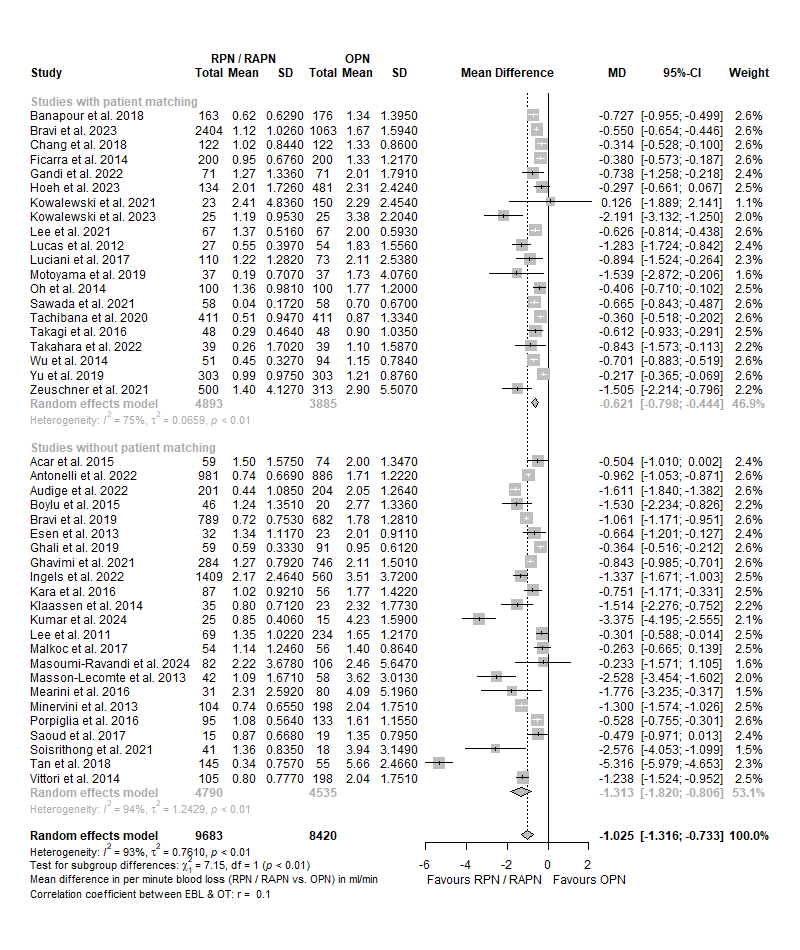

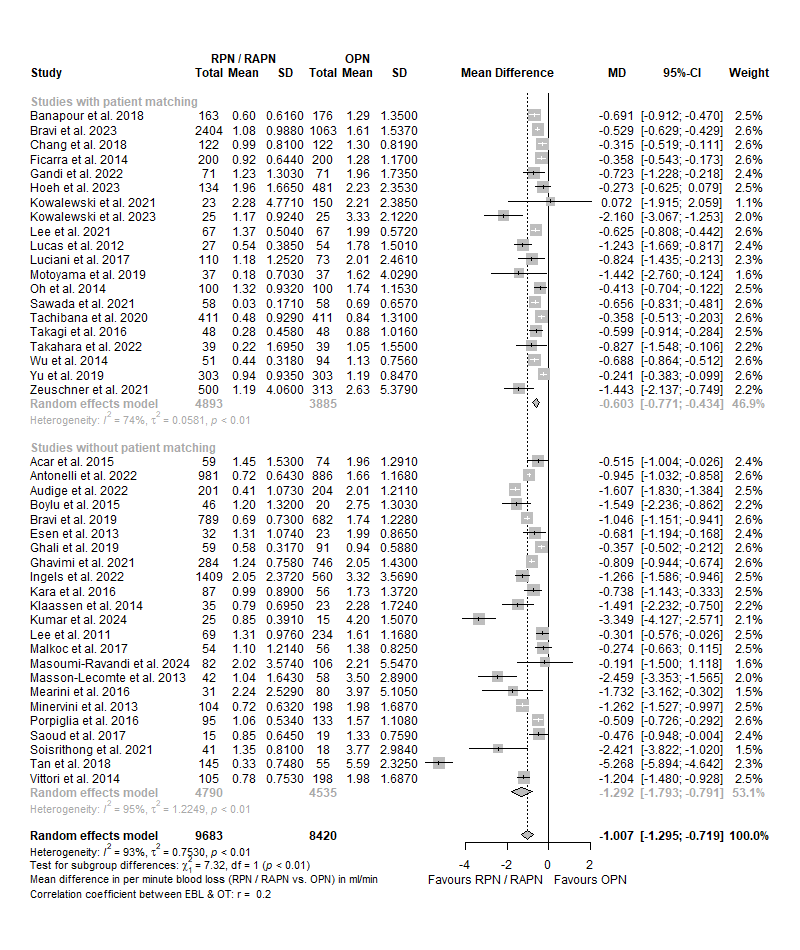

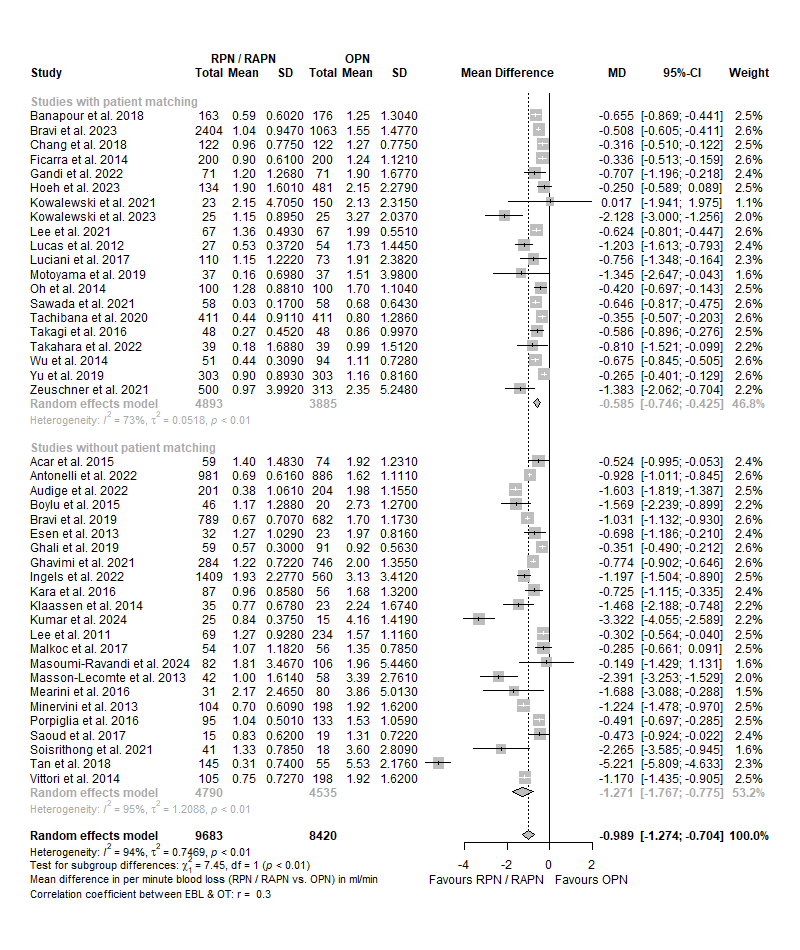

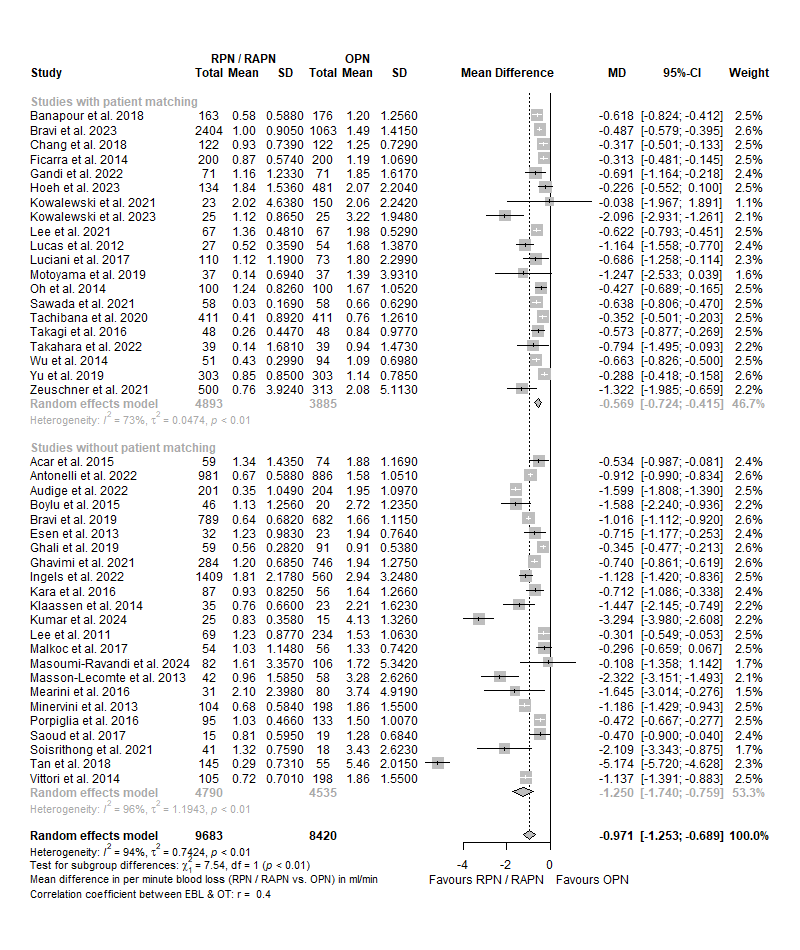

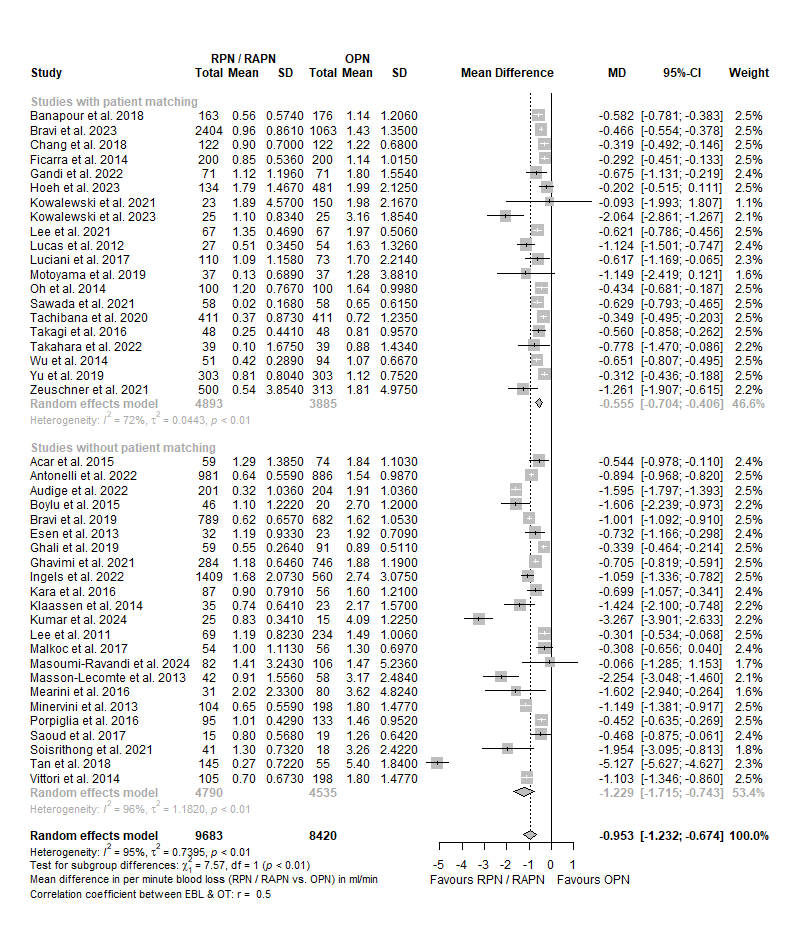

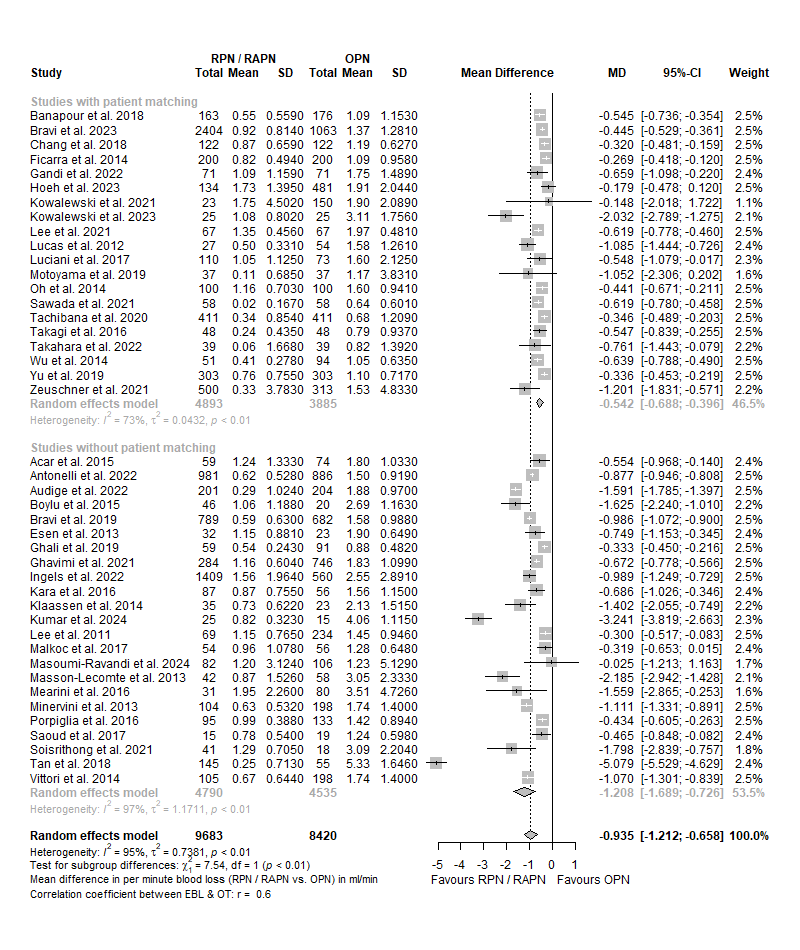

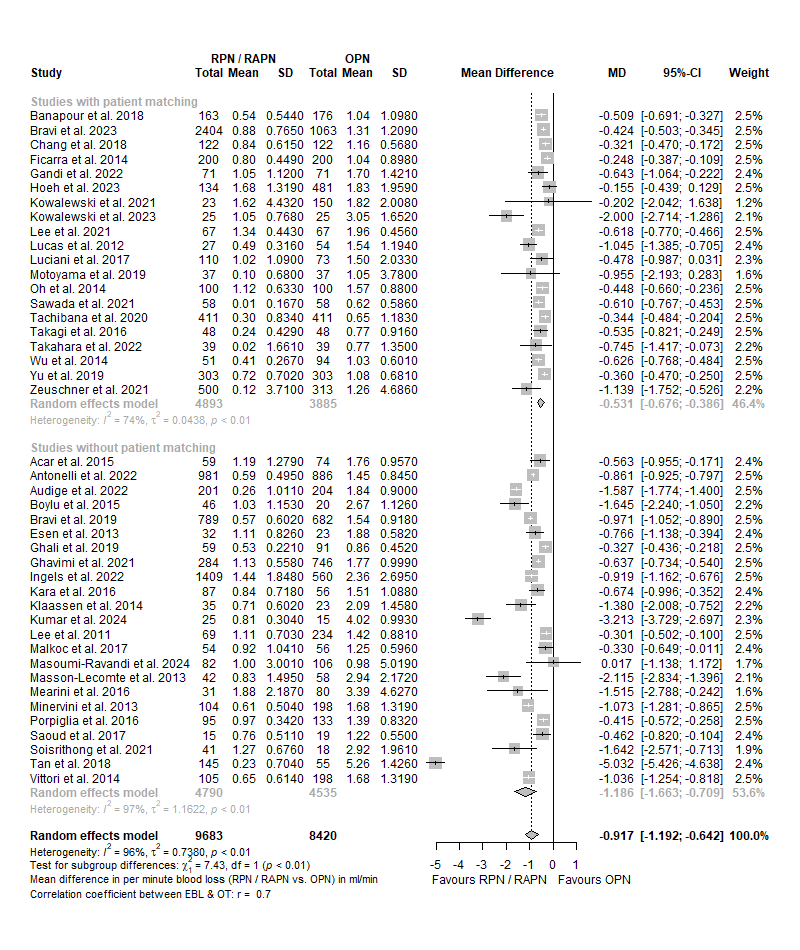

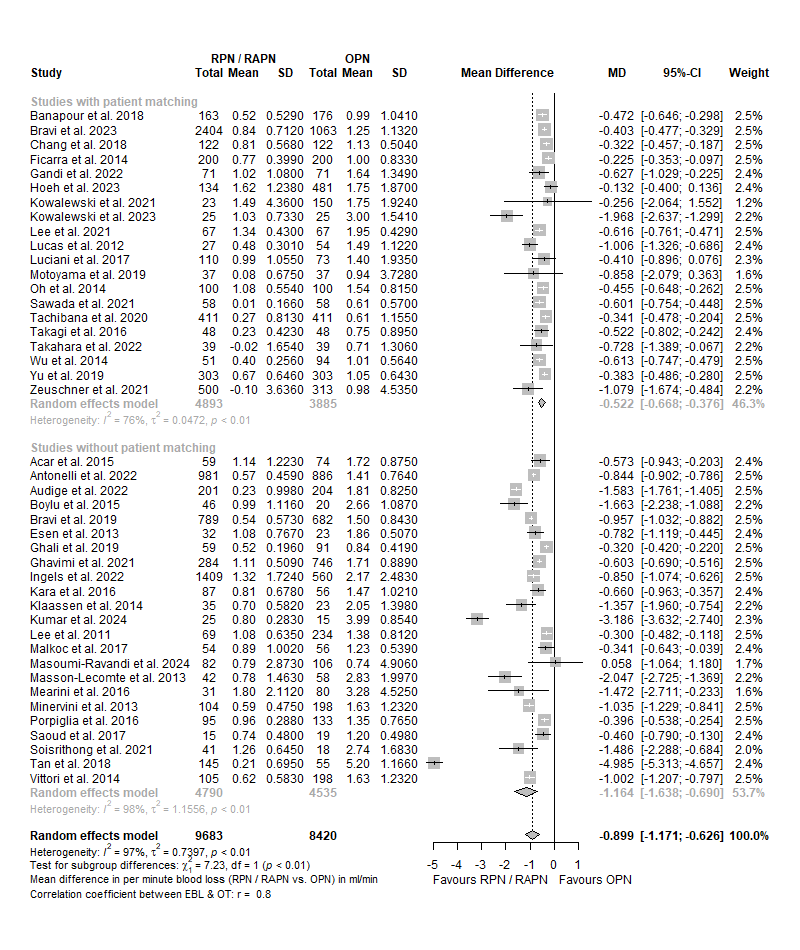

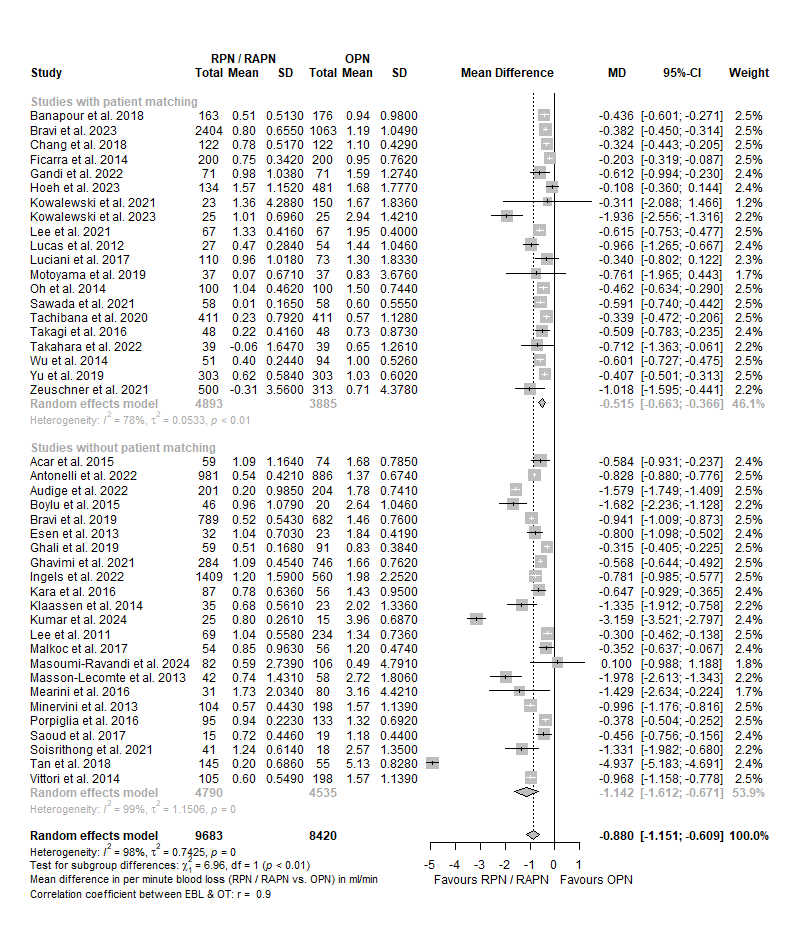

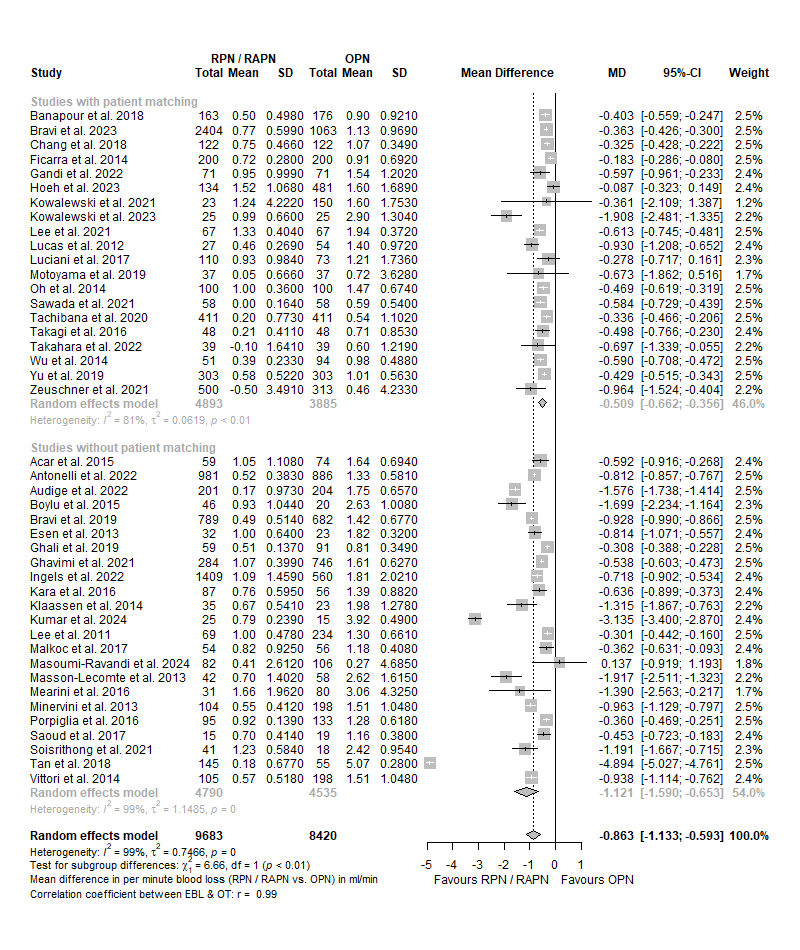
*

Supplementary SA Forest plots: Successive forest plots depicting the stepwise MD_Q_ assessment in studies with or without patient matching, for the gradual transition of r from -0.99 to +0.99. Abbreviations: EBL: estimated blood loss, OT: operative time, r: Pearson’s correlation coefficient between EBL and OT.

SENSITIVITY ANALYSIS FOREST PLOTS

(SUBGROUPS BY REFERRAL CENTER COUNT)

*
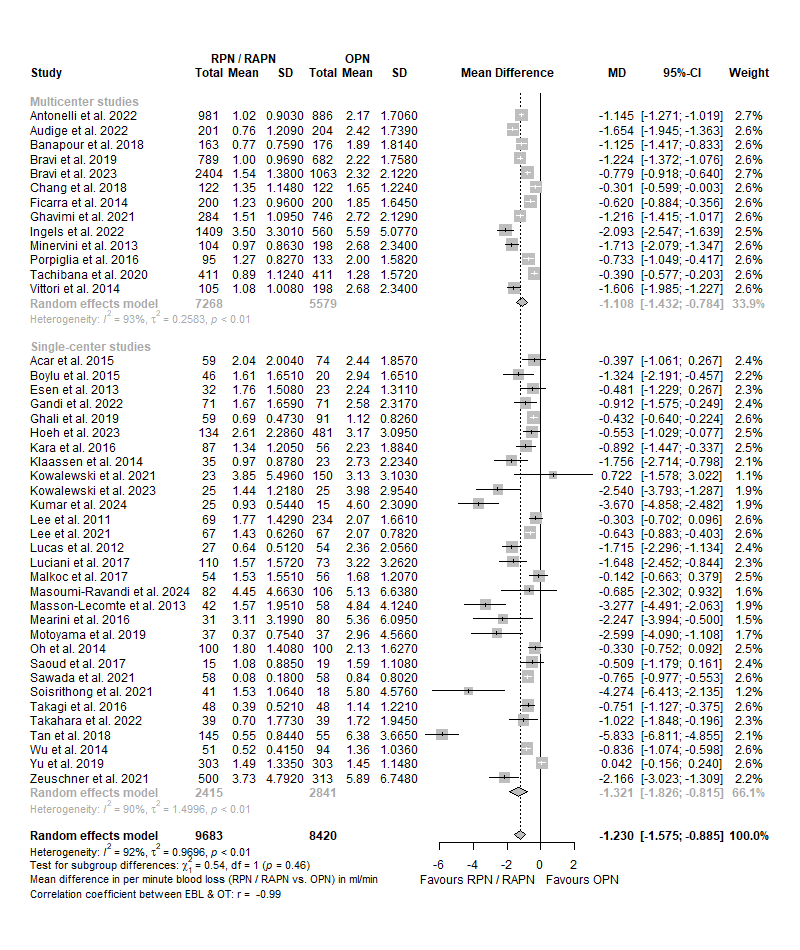

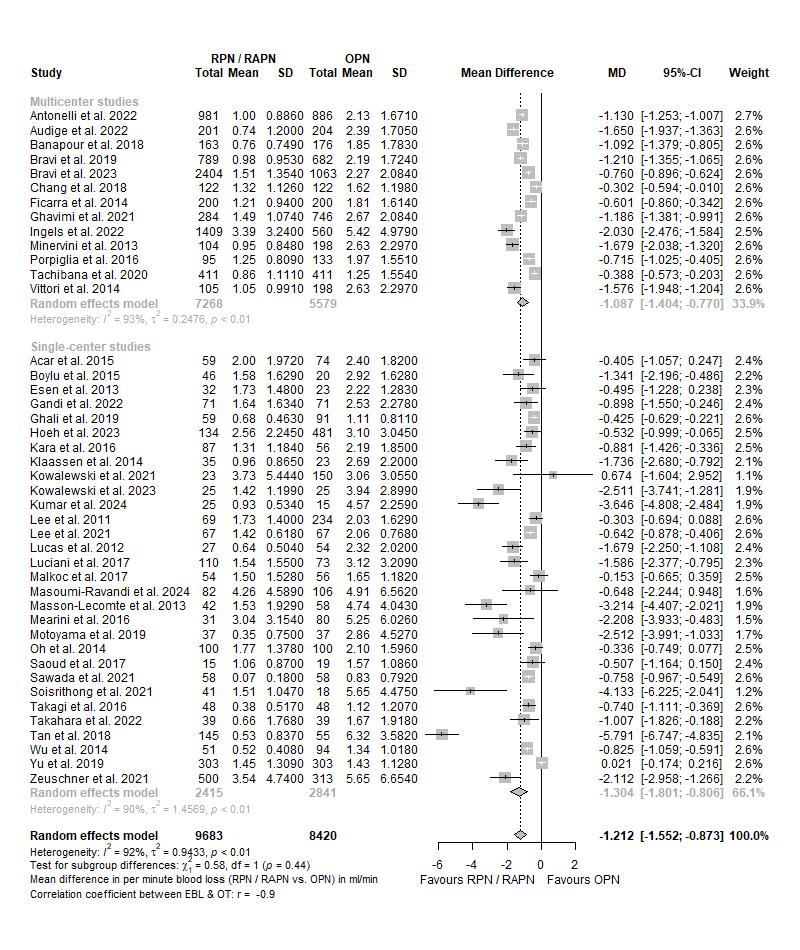

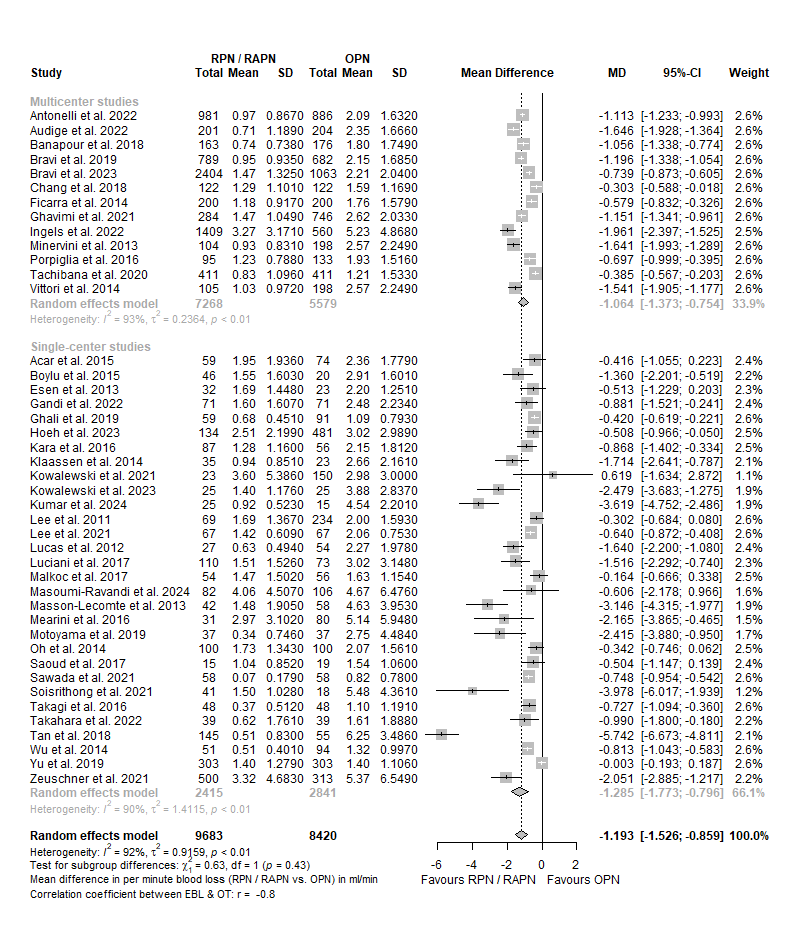

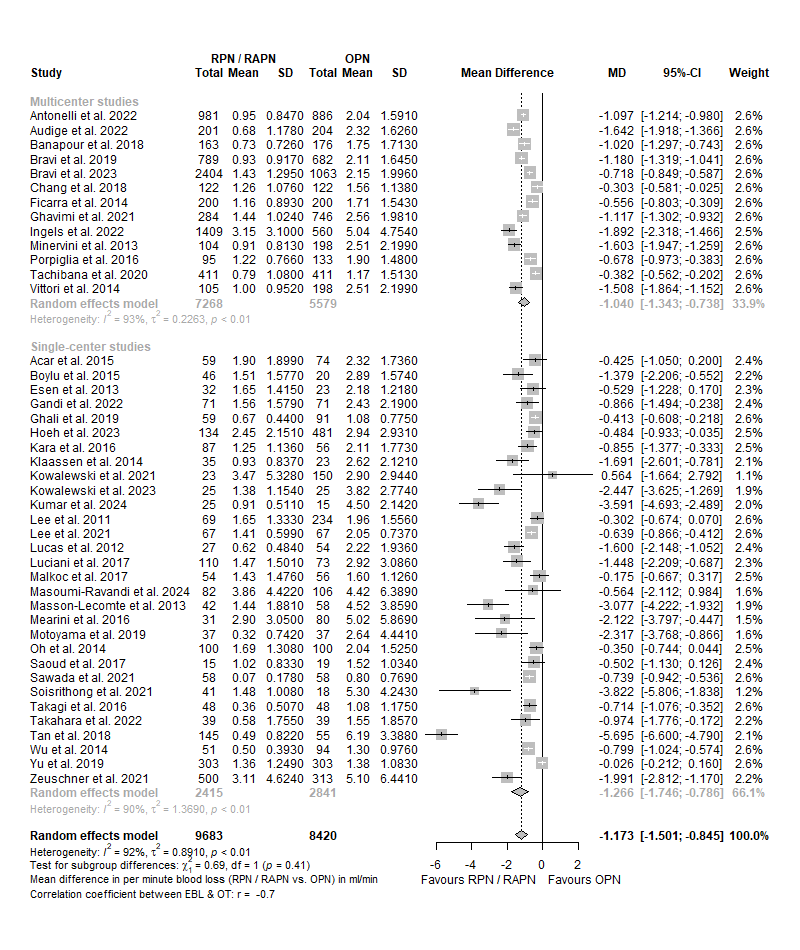

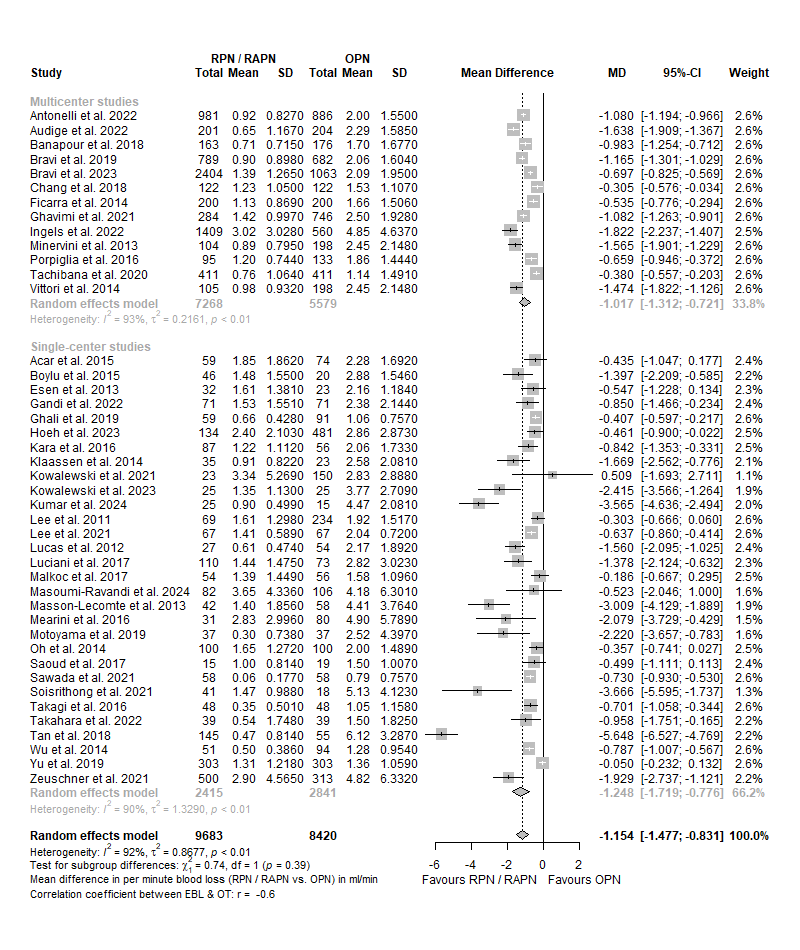

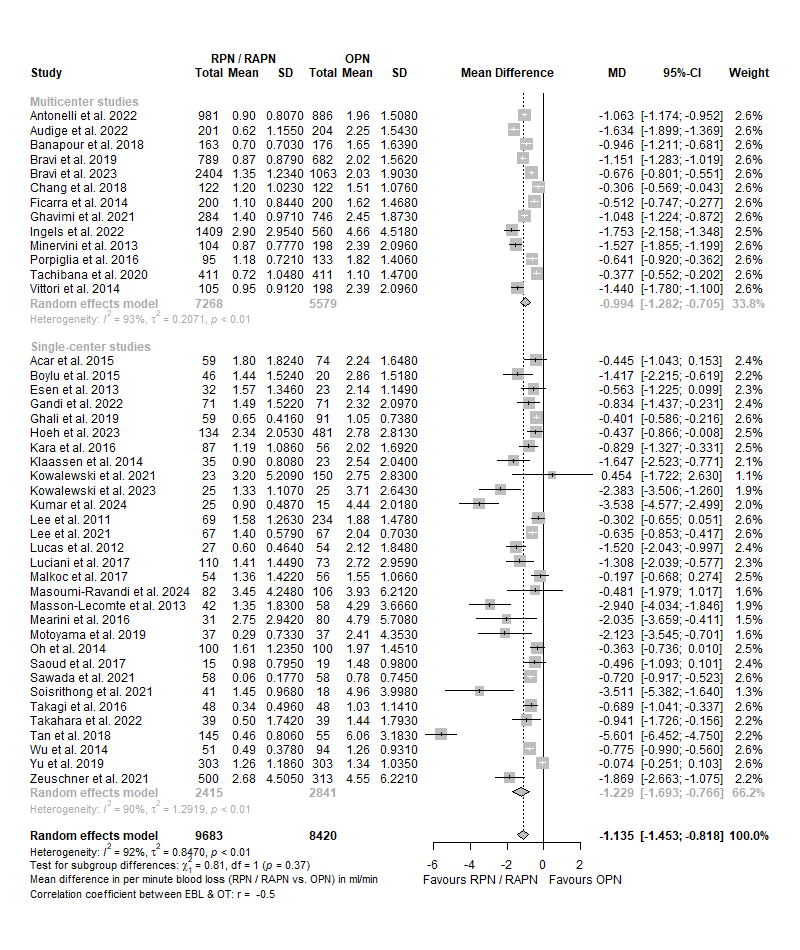

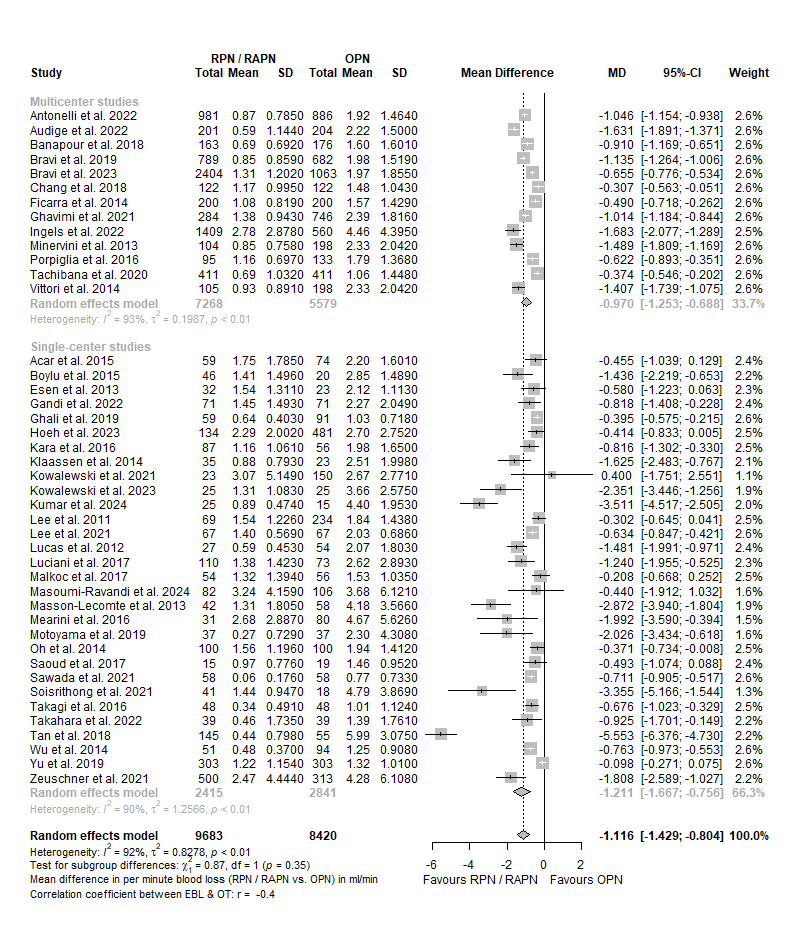

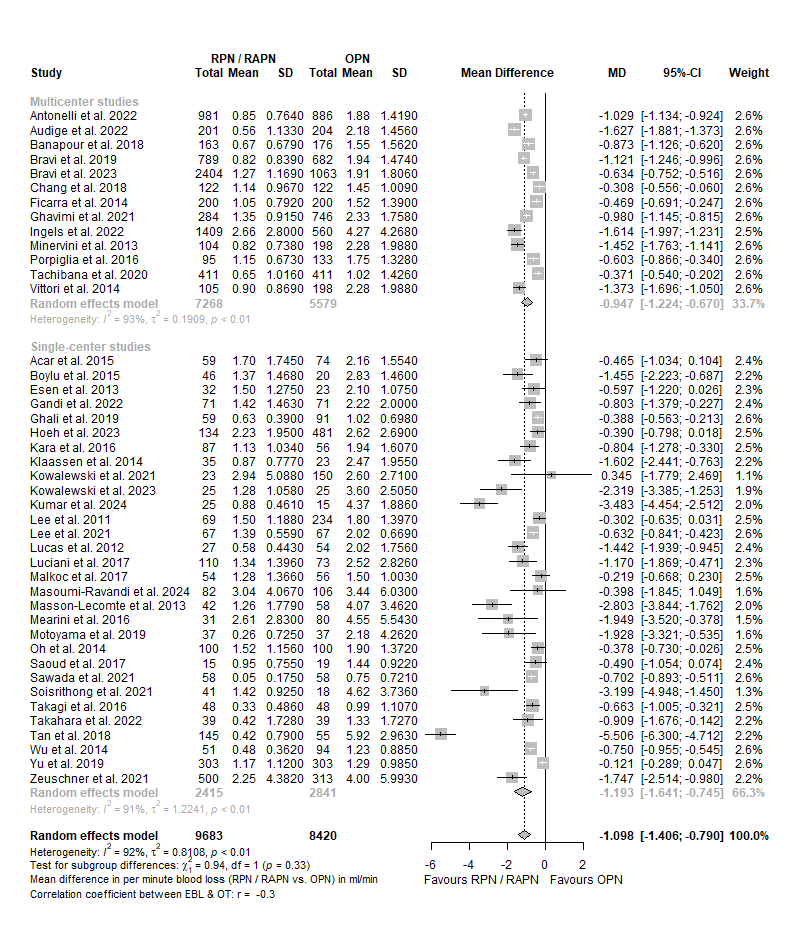

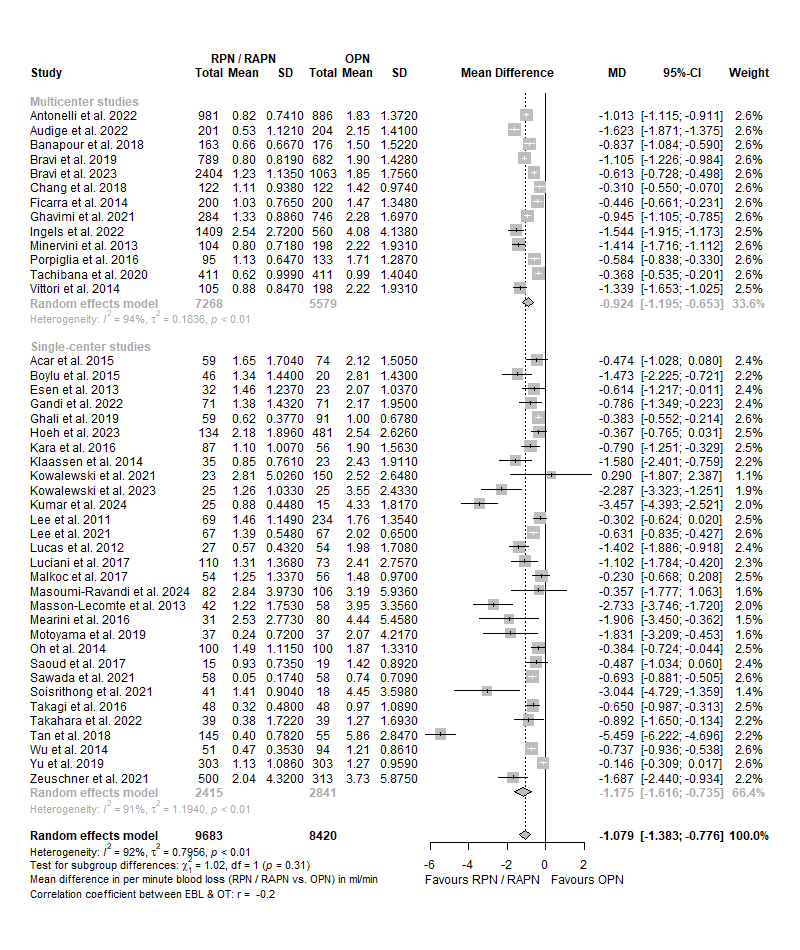

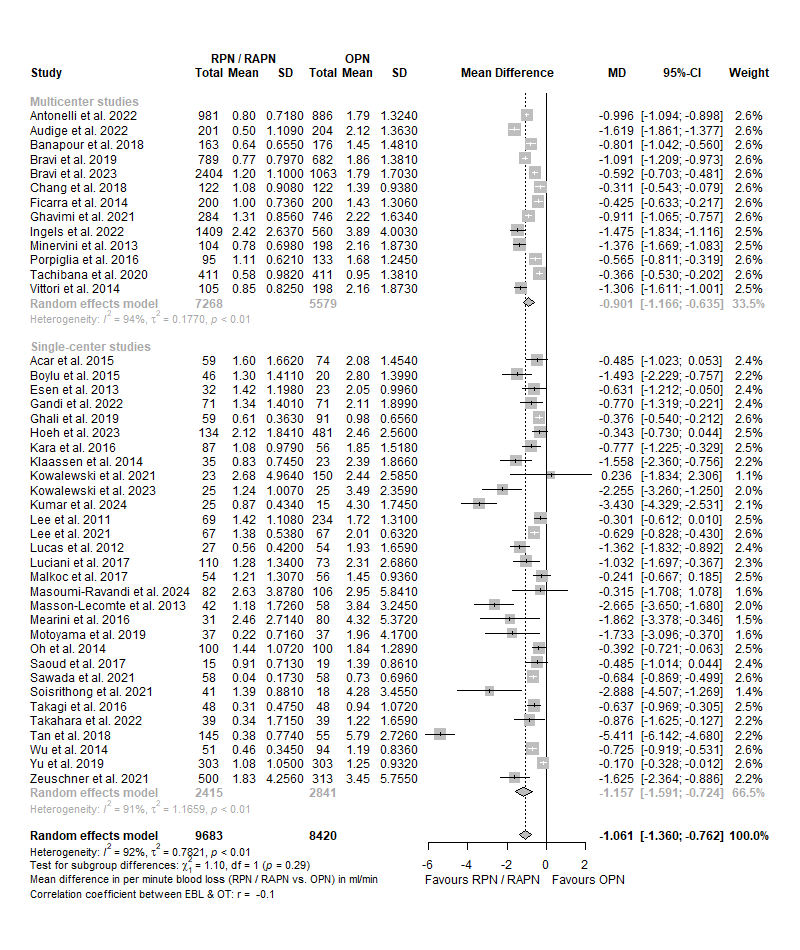

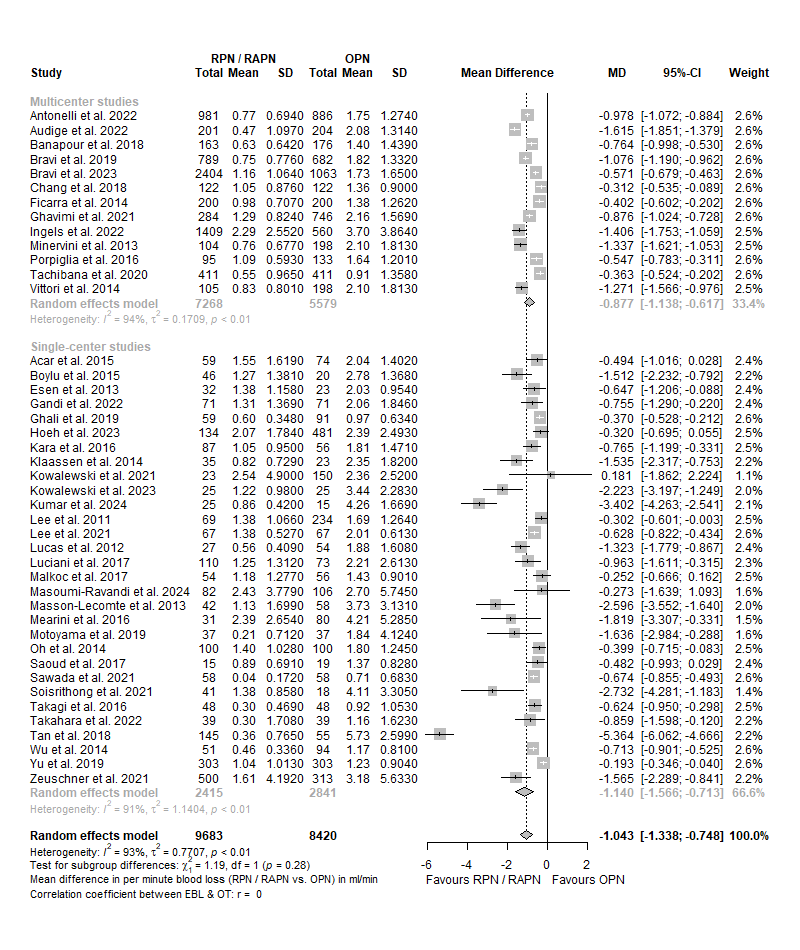

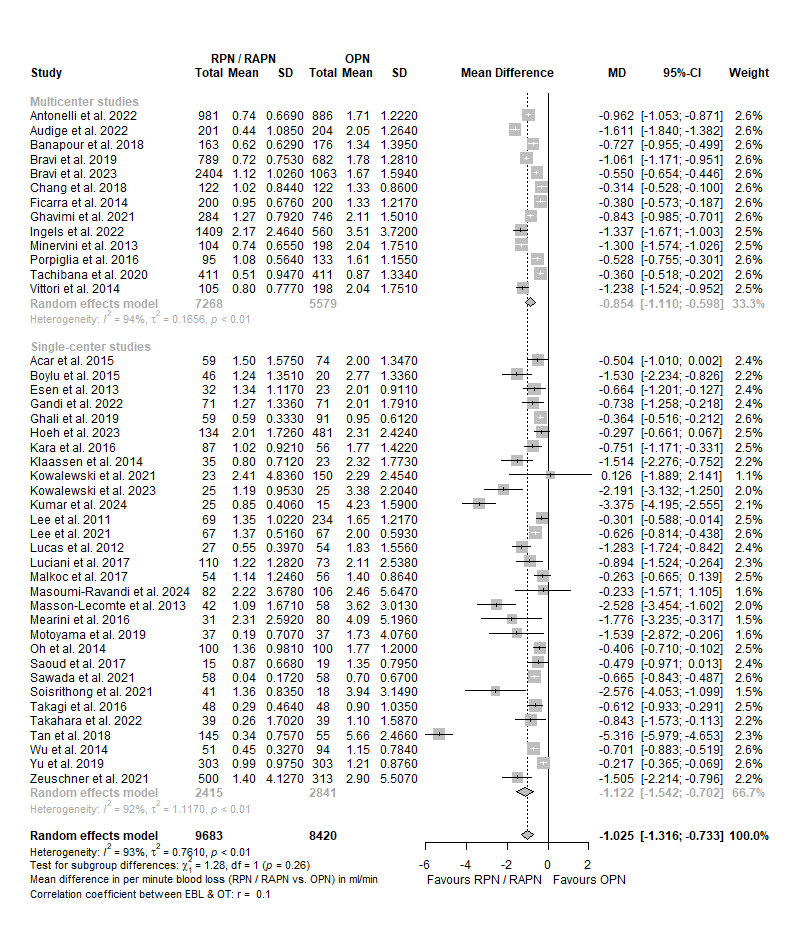

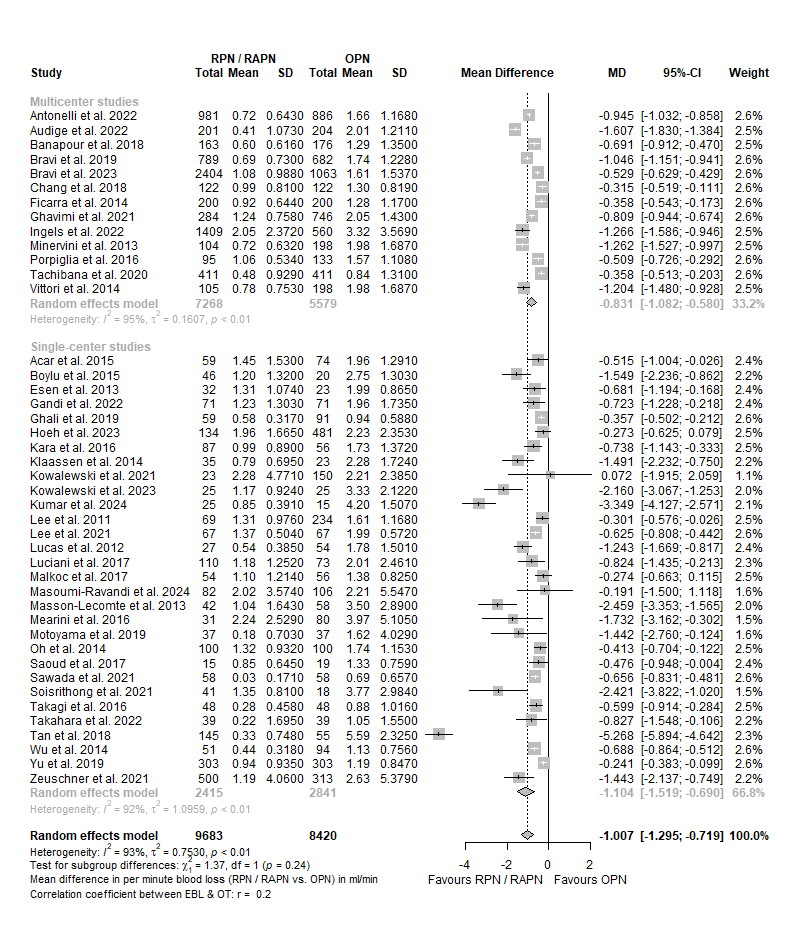

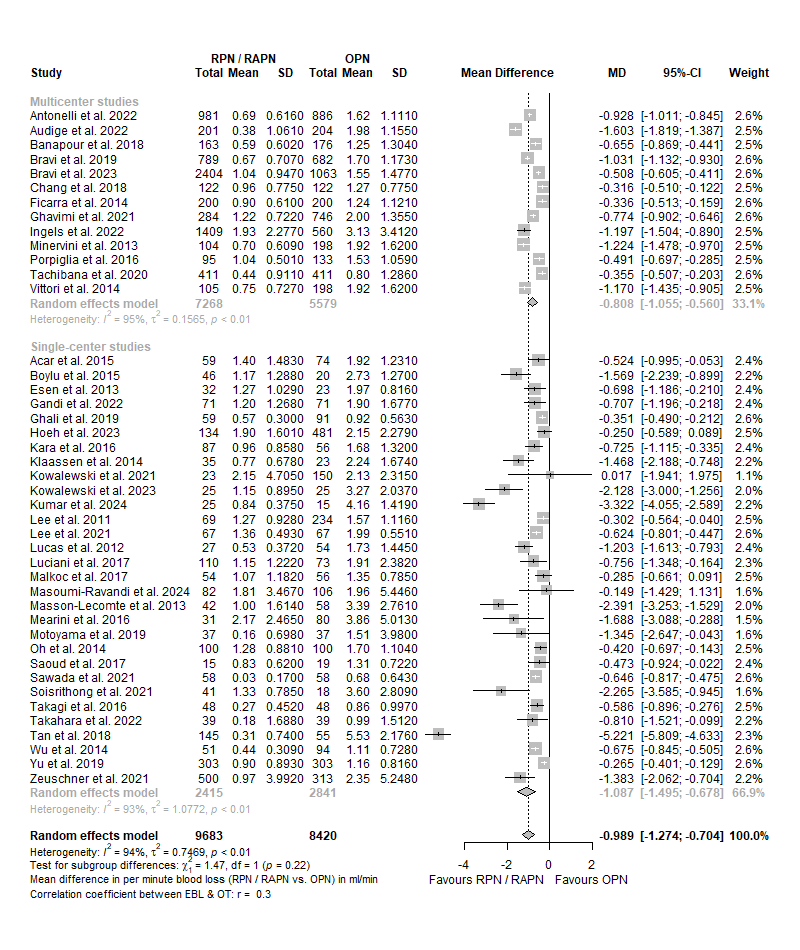

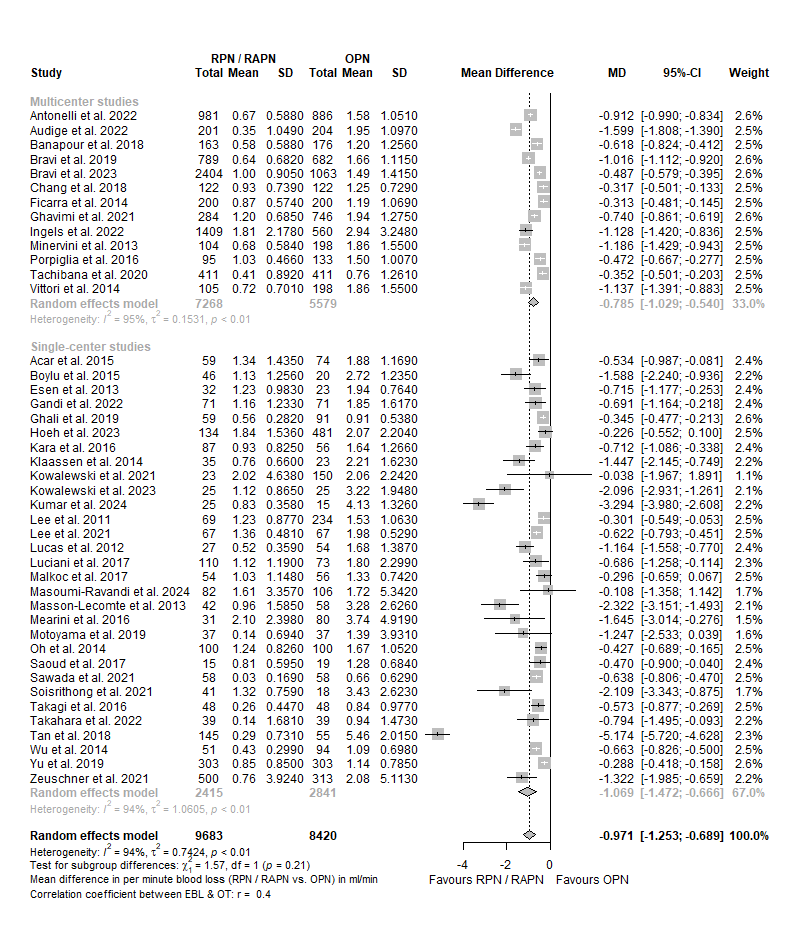

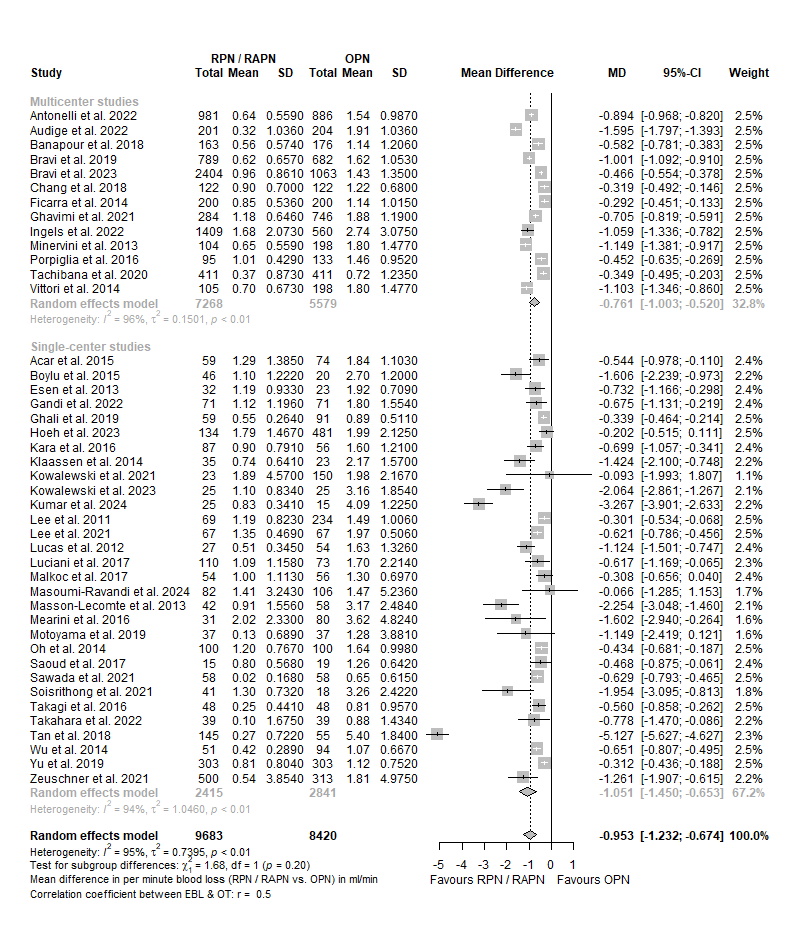

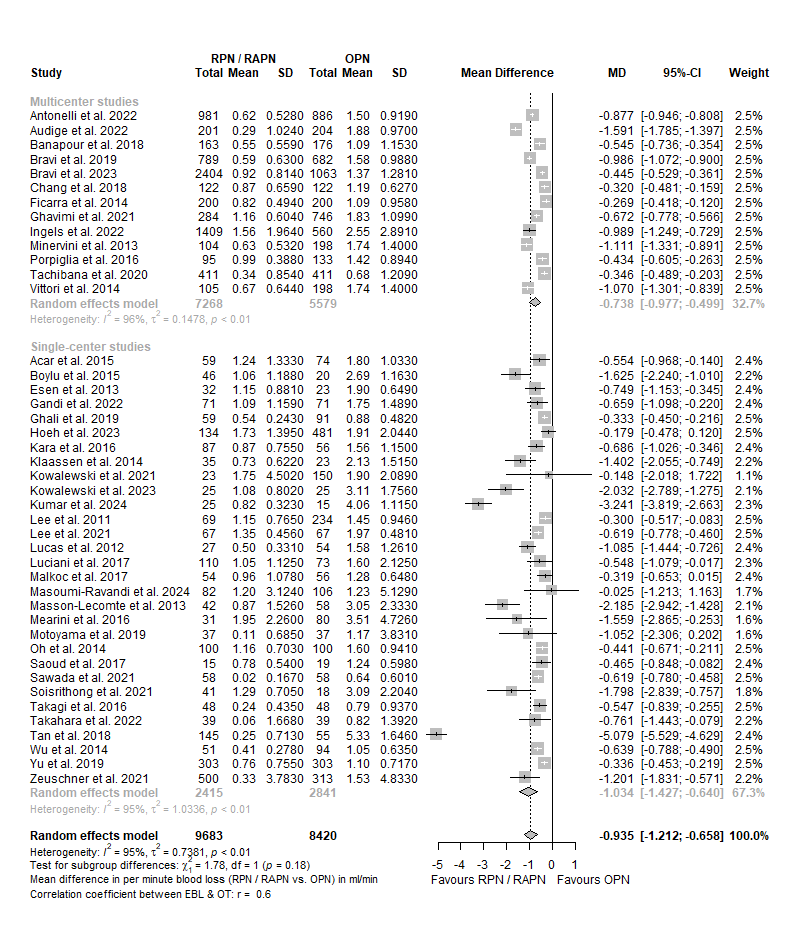

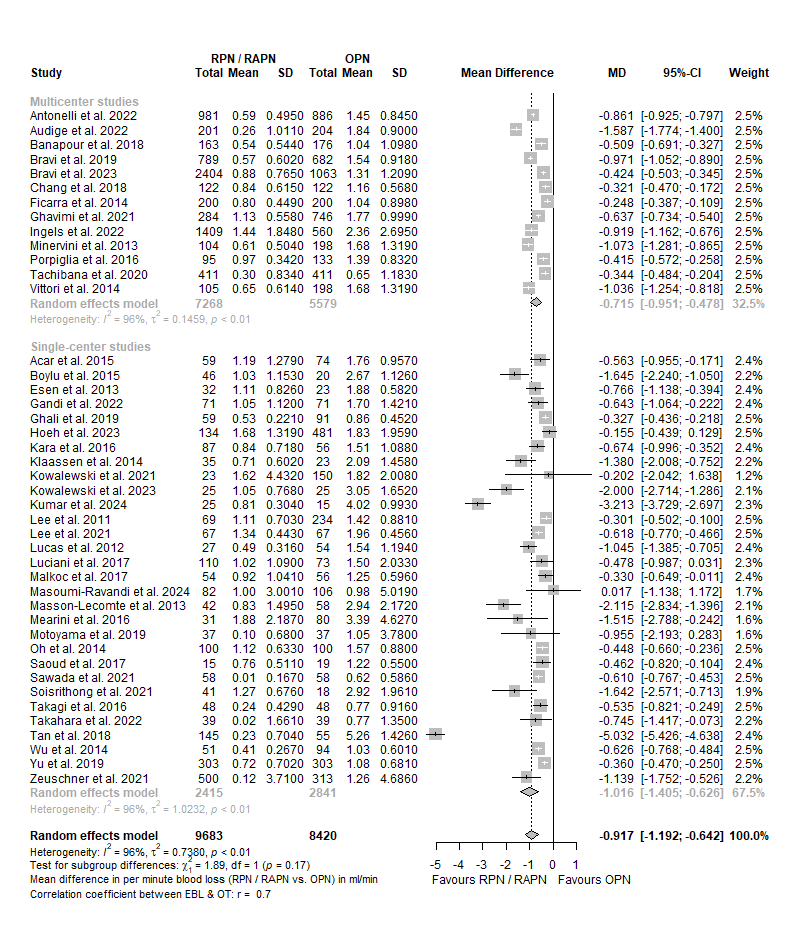

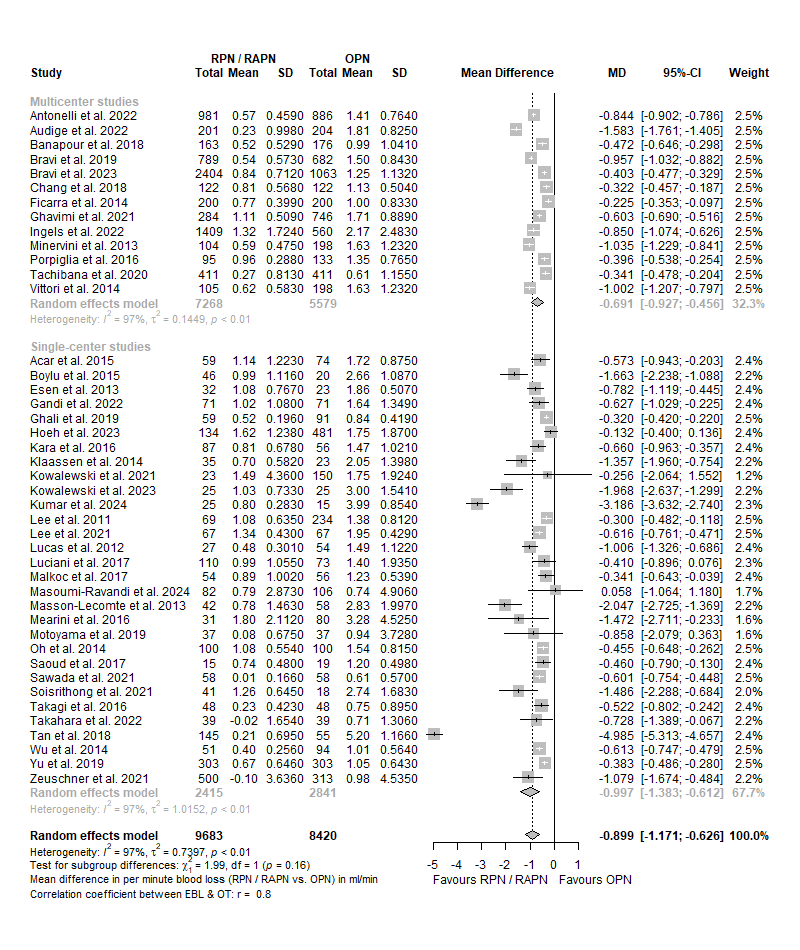

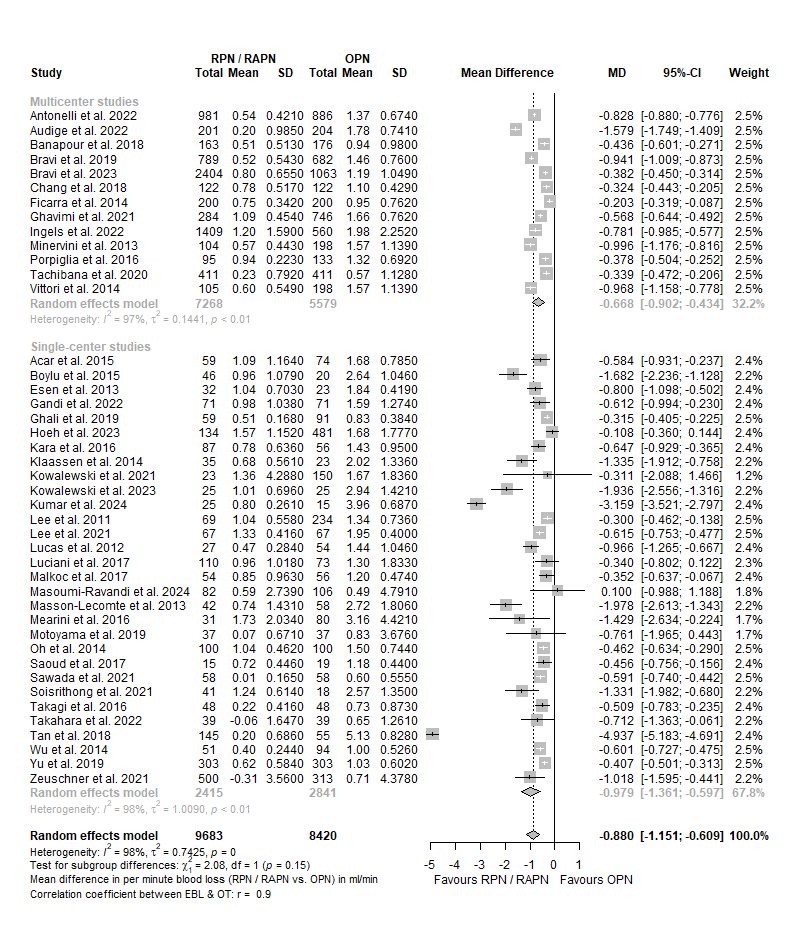

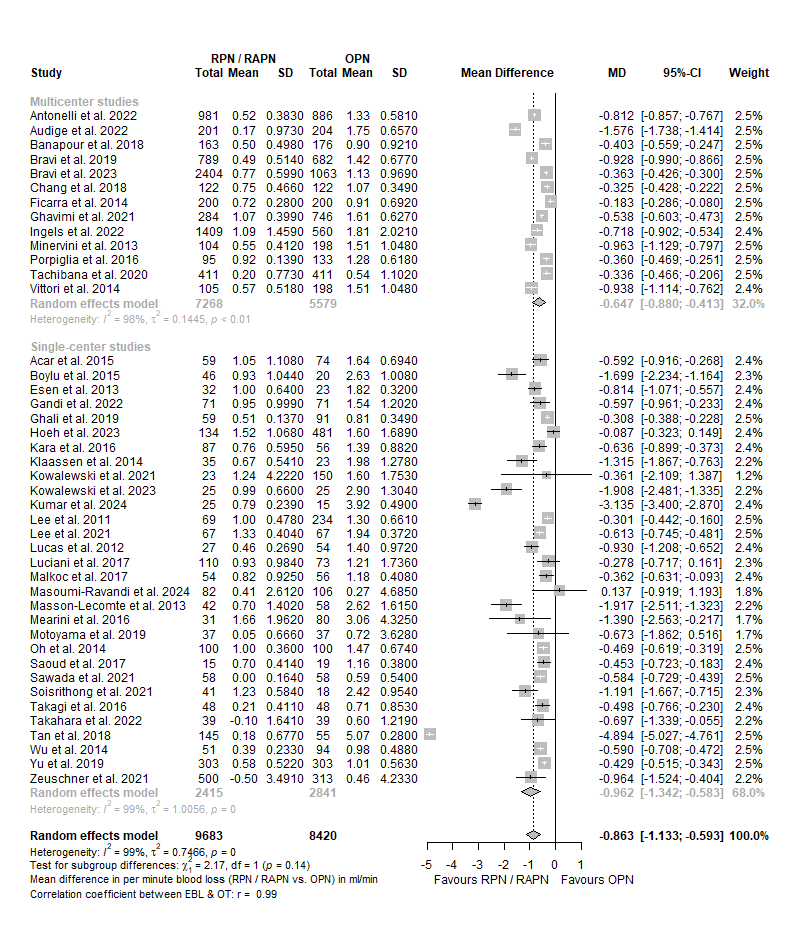
*

Supplementary SA Forest plots: Successive forest plots depicting the stepwise MD_Q_ assessment in multi- or single-center studies, for the gradual transition of r from -0.99 to +0.99. Abbreviations: EBL: estimated blood loss, OT: operative time, r: Pearson’s correlation coefficient between EBL and OT.

SENSITIVITY ANALYSIS FOREST PLOTS

(SUBGROUPS BY ROBINS-I CLASS)

*
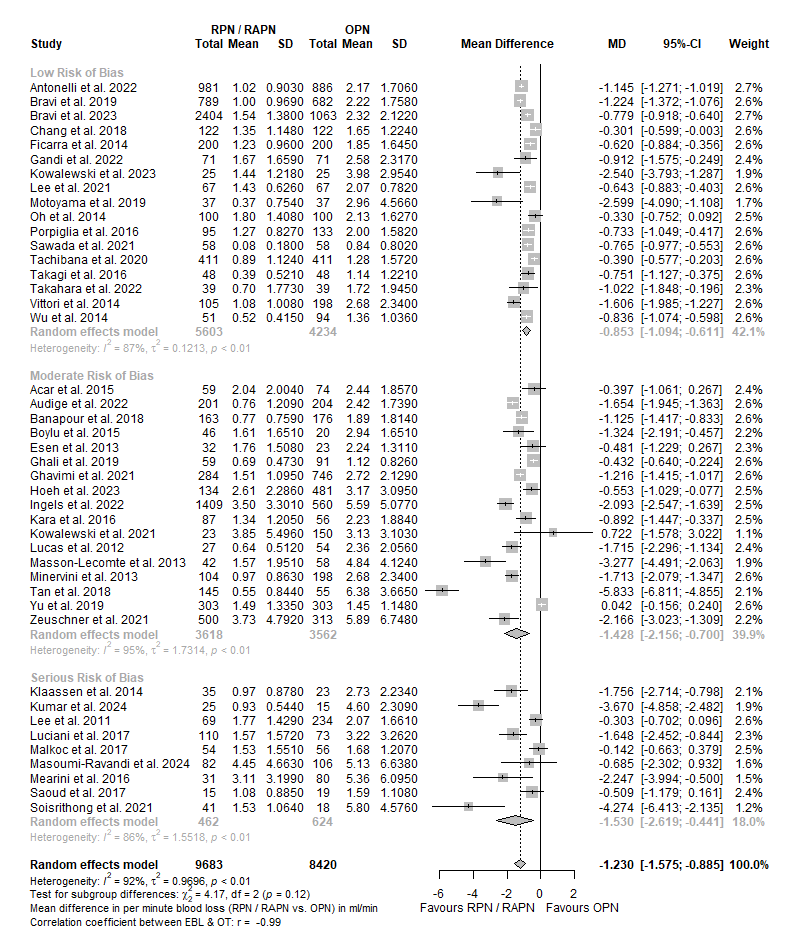

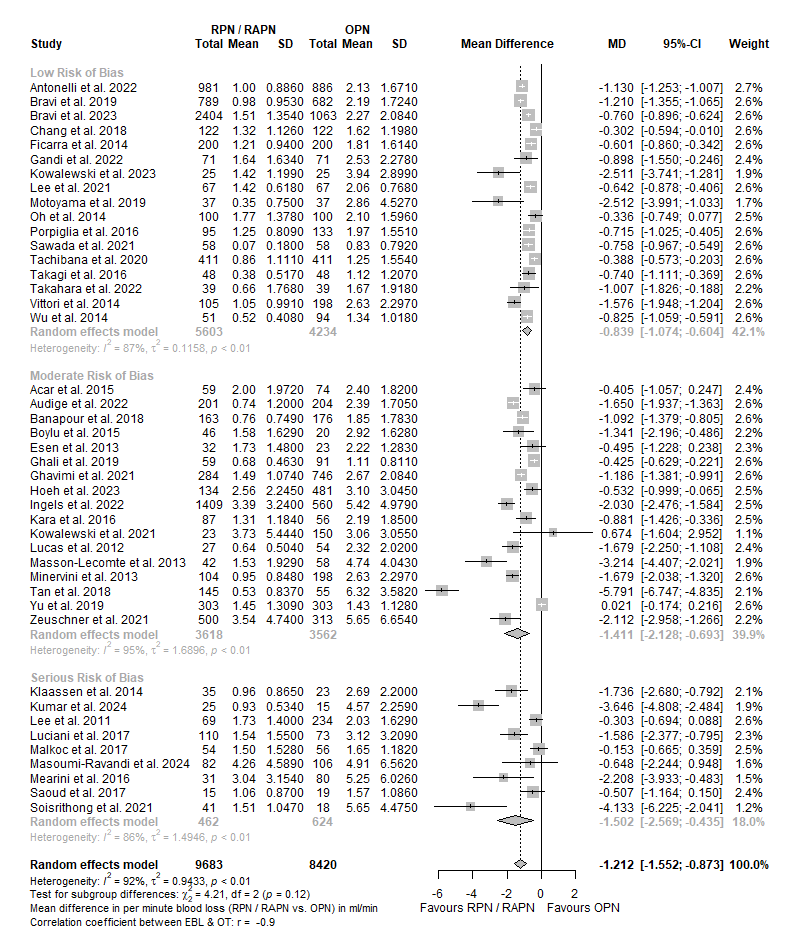

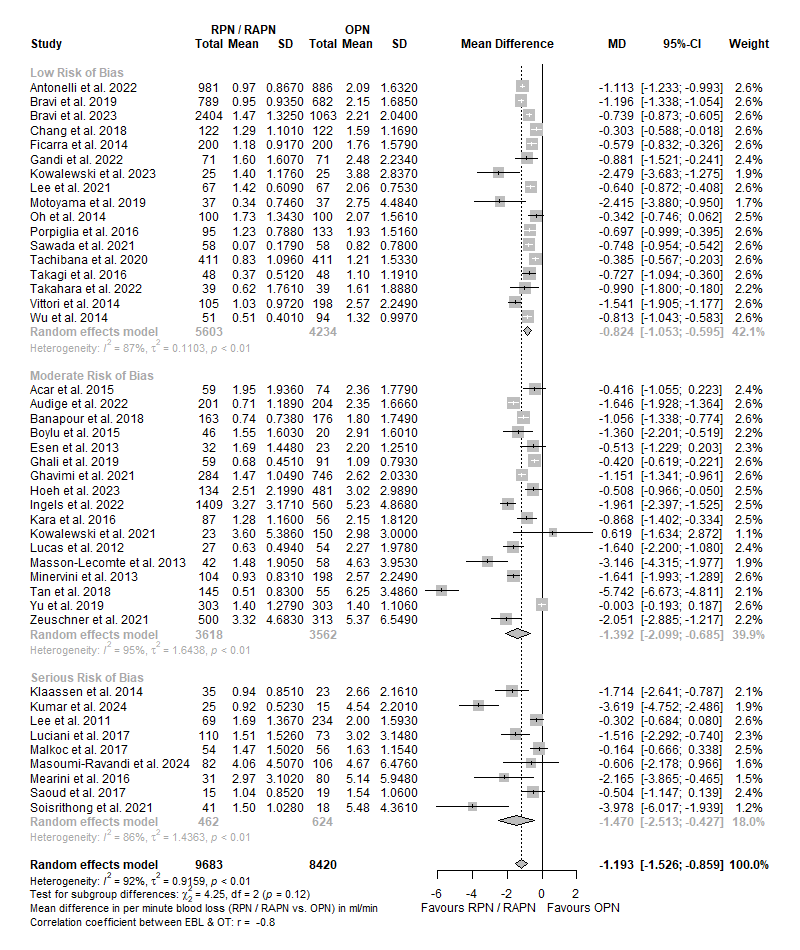

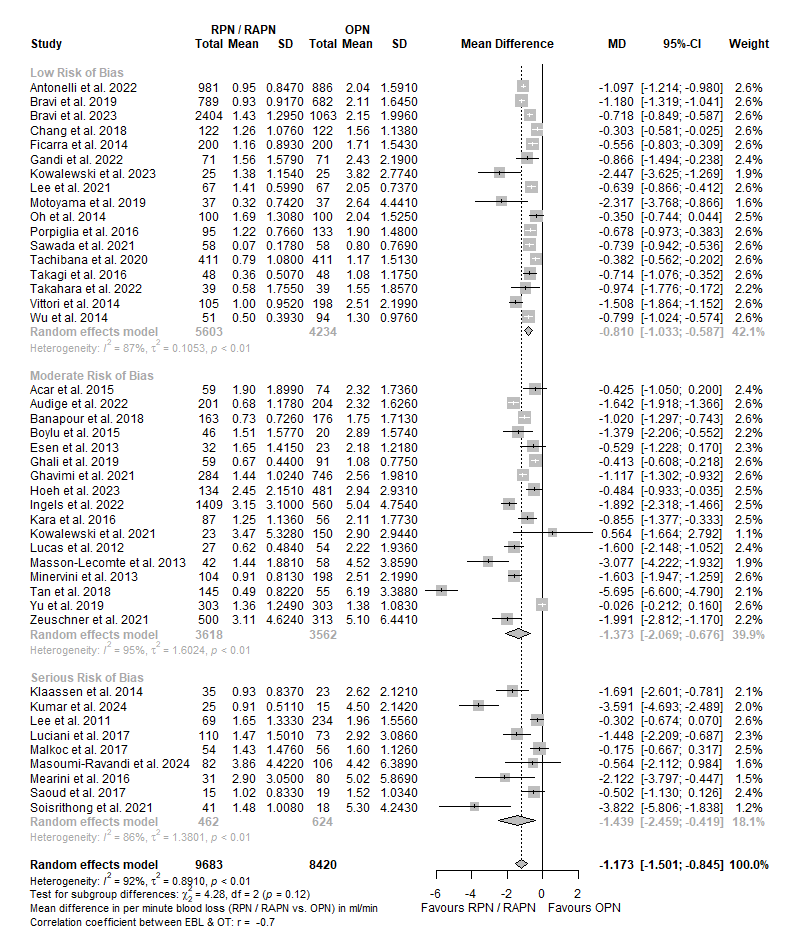

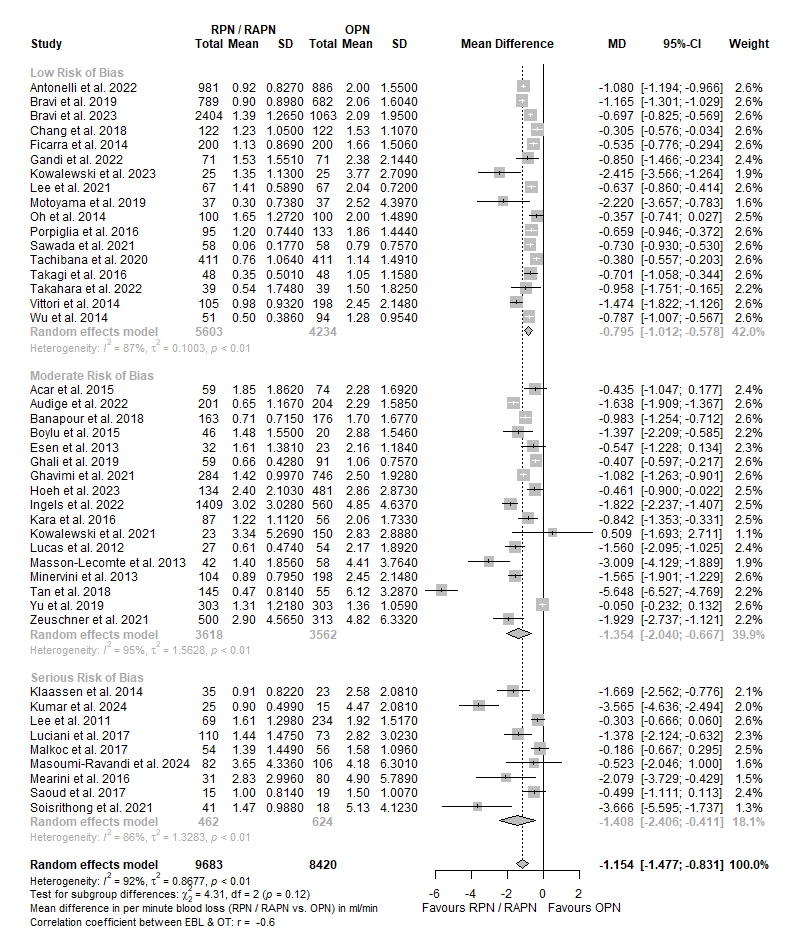

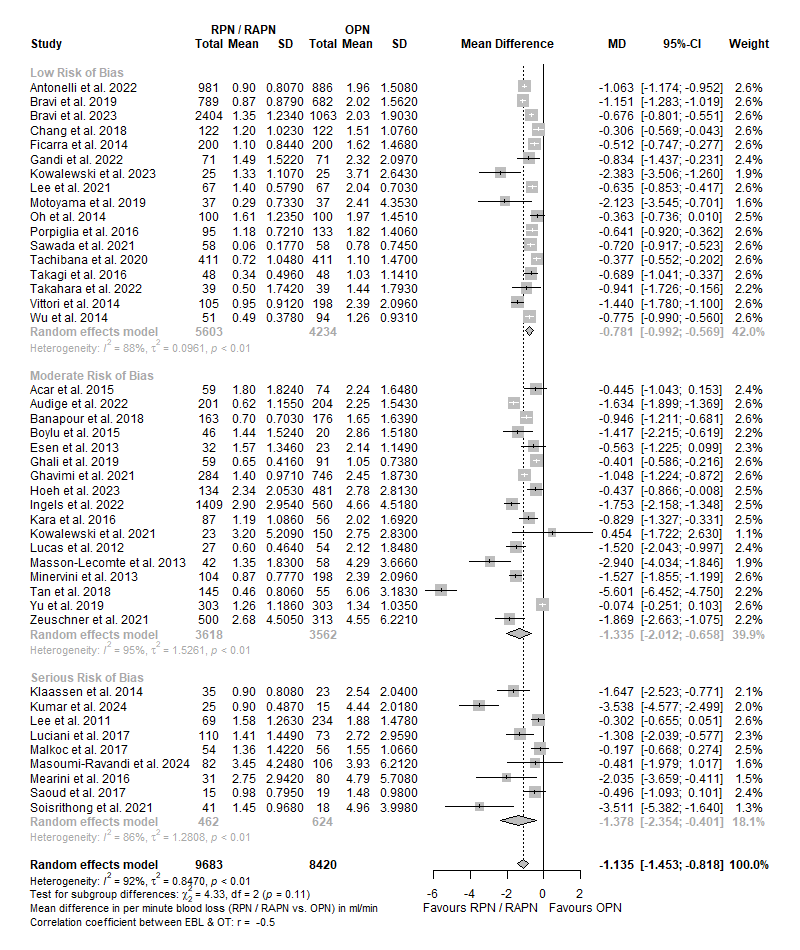

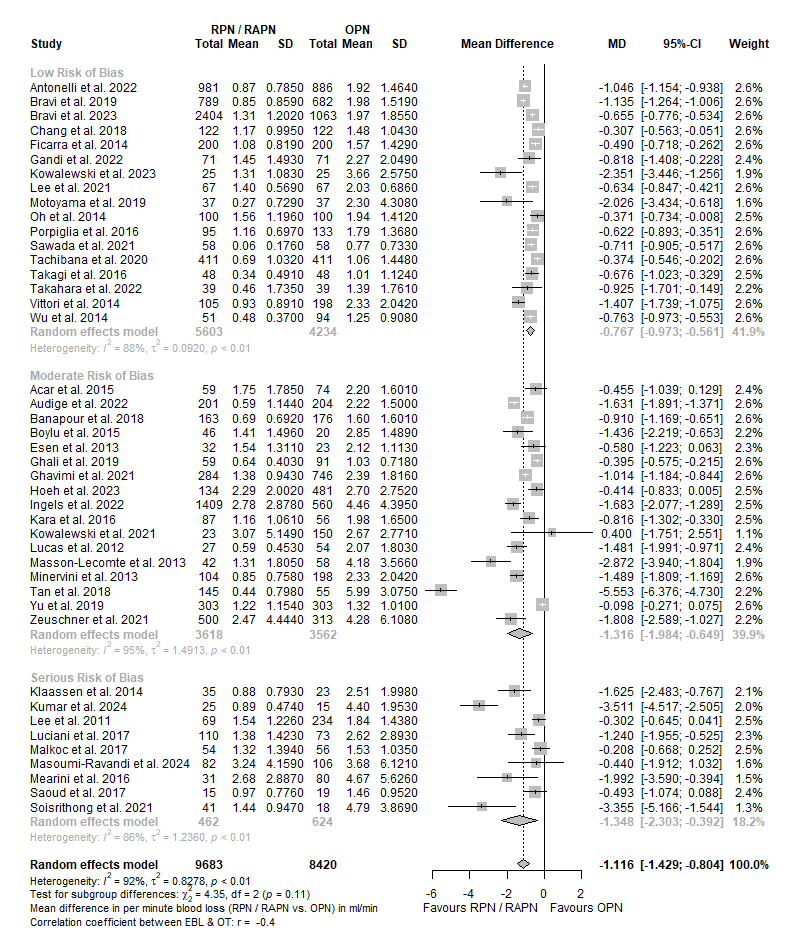

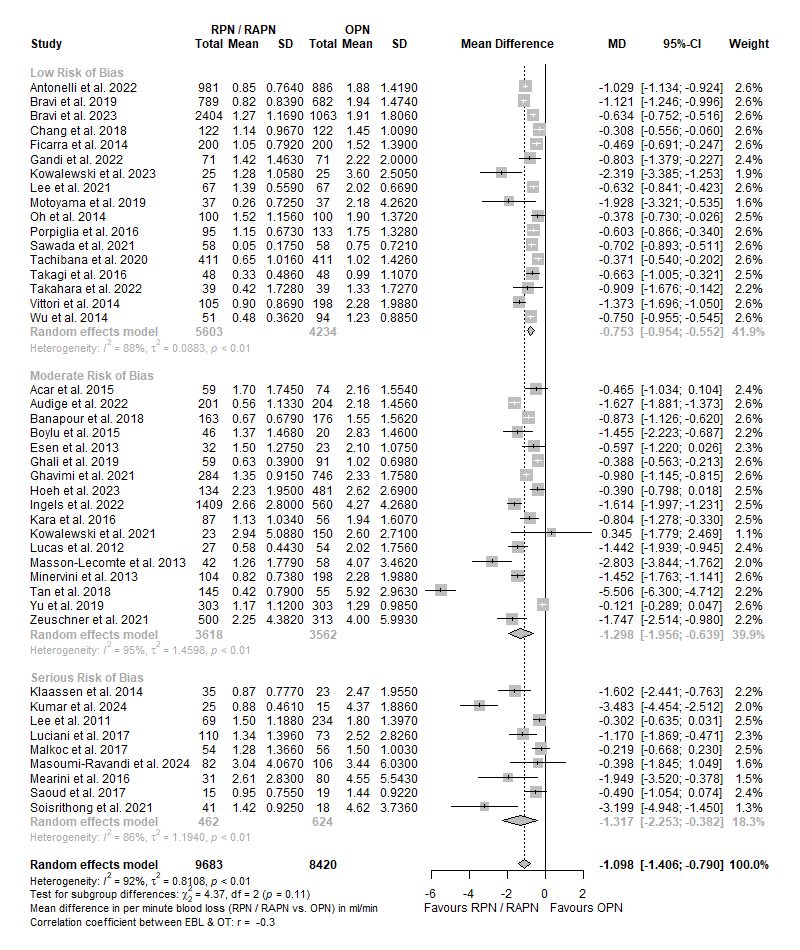

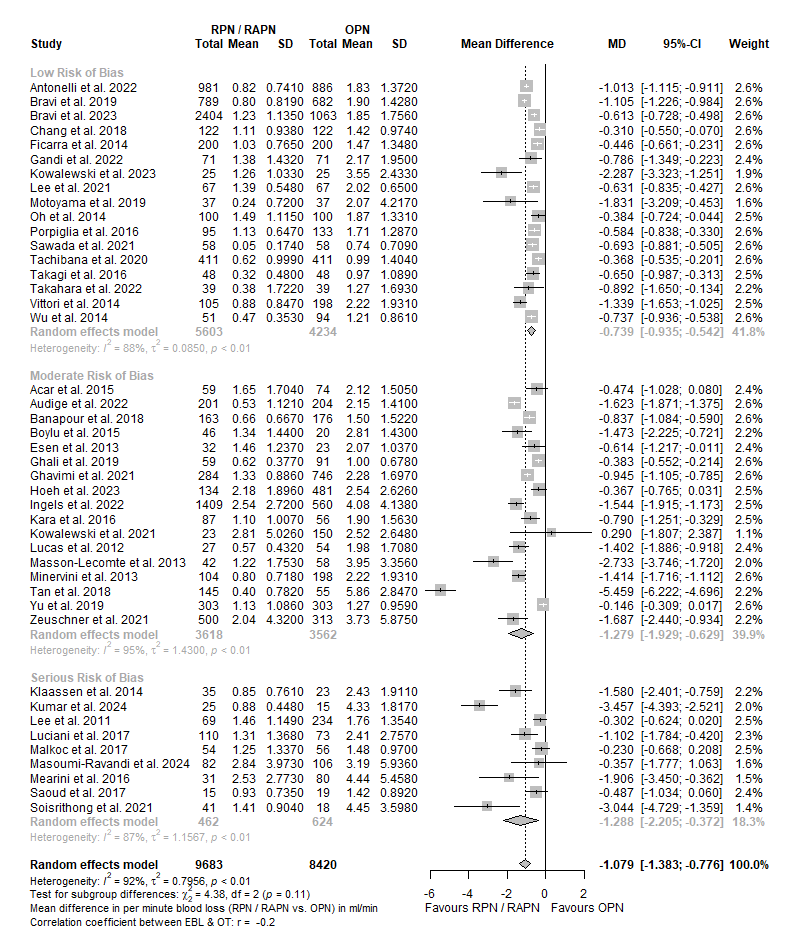

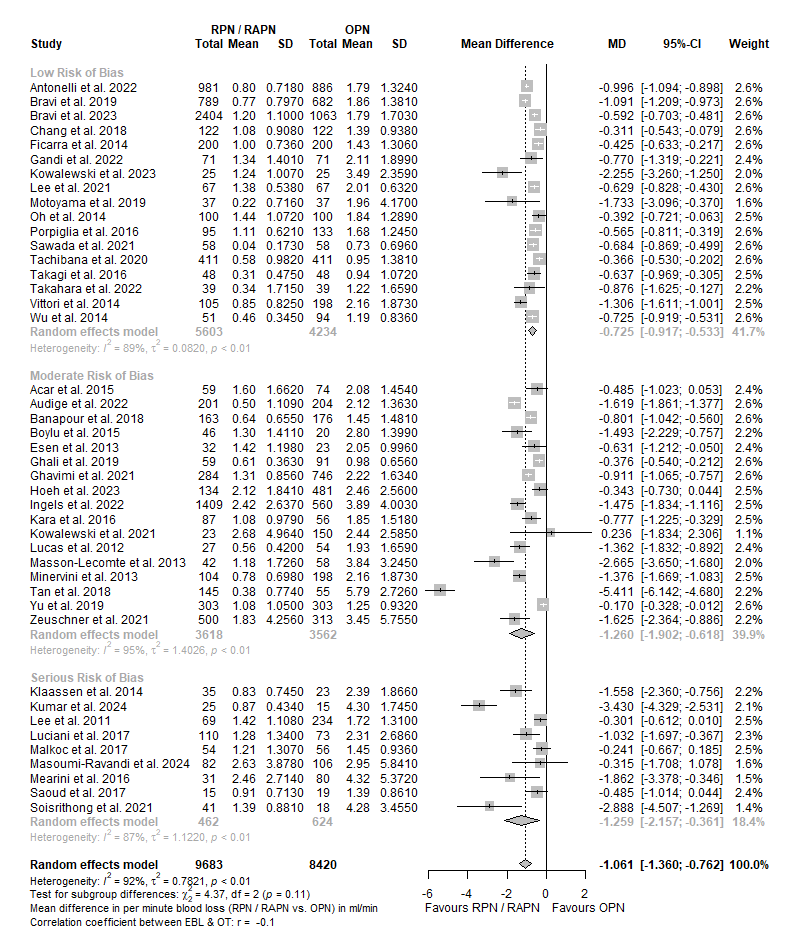

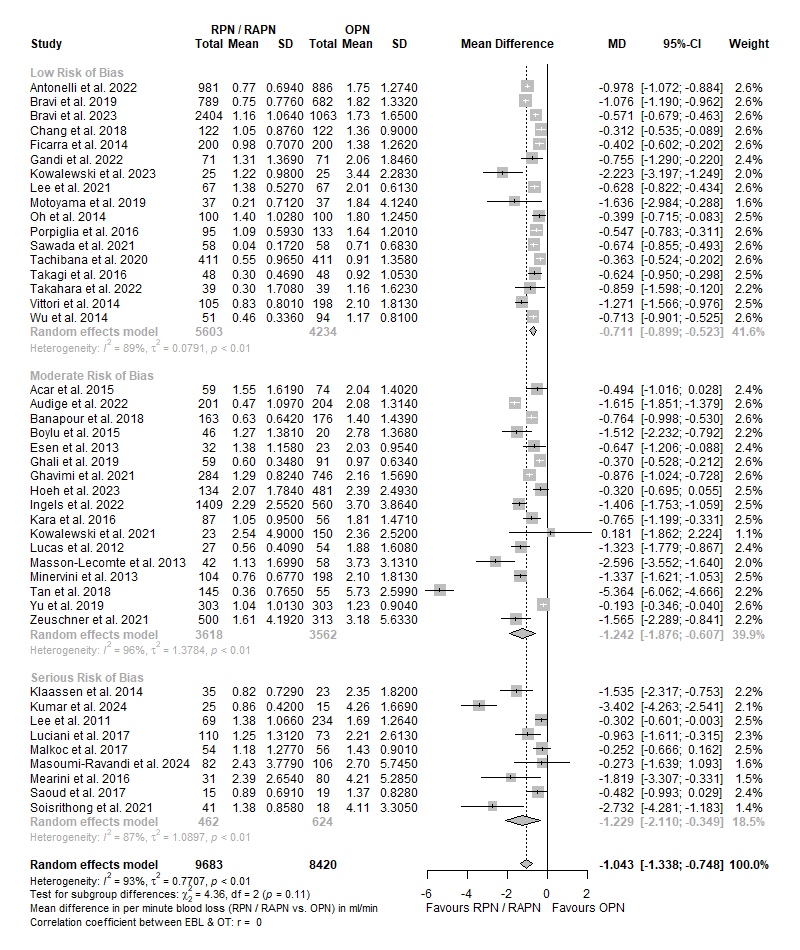

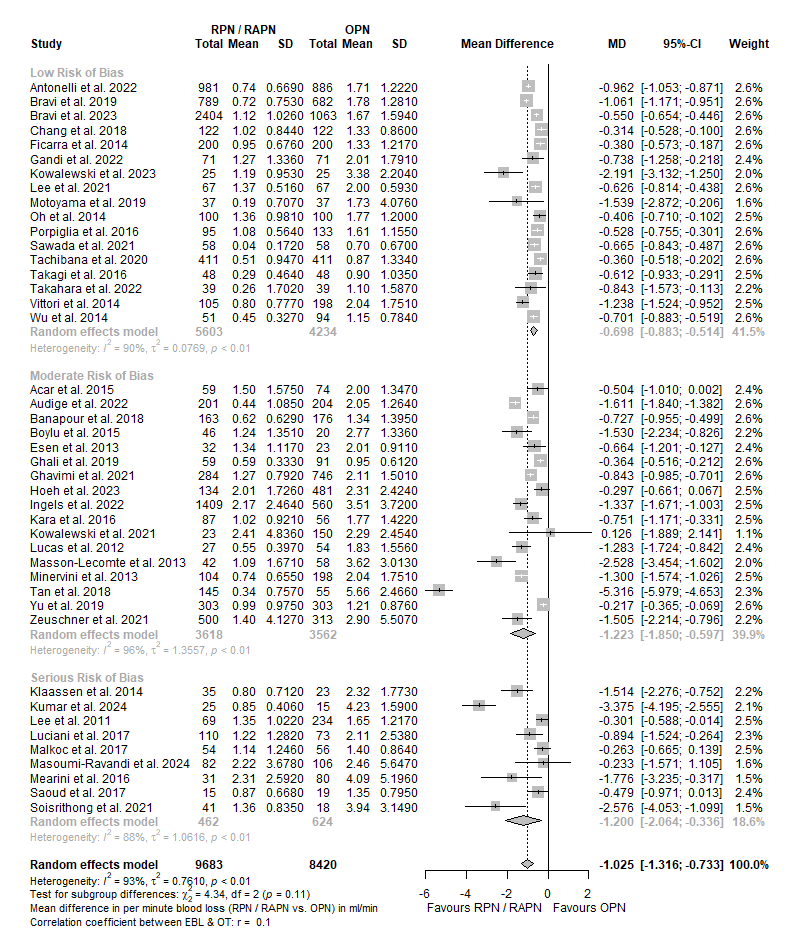

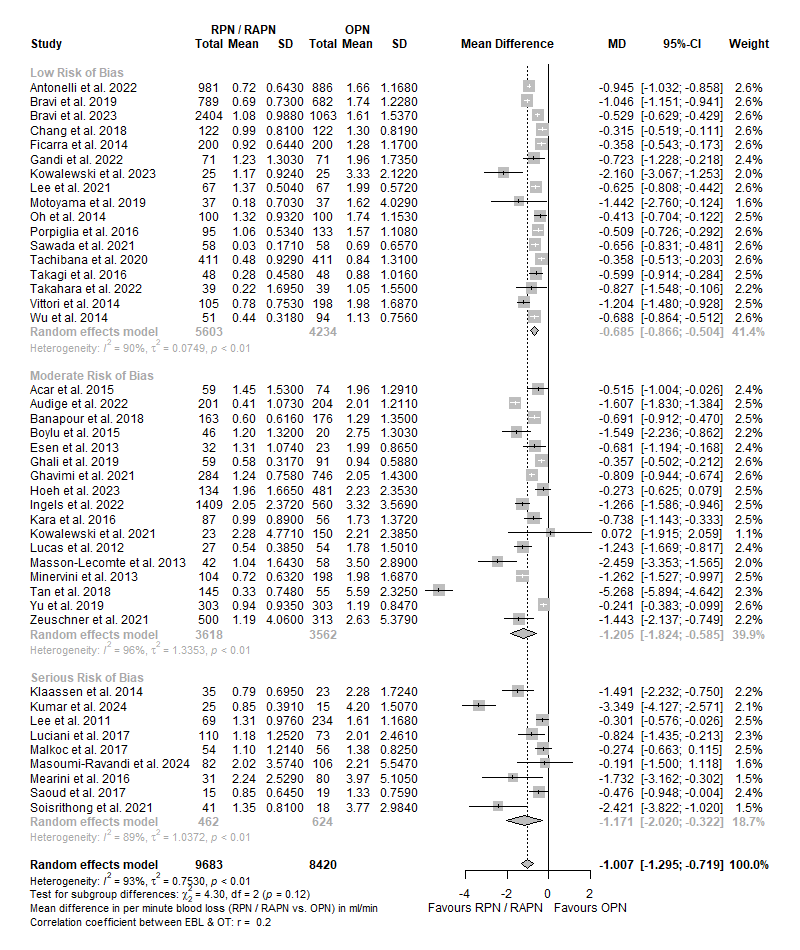

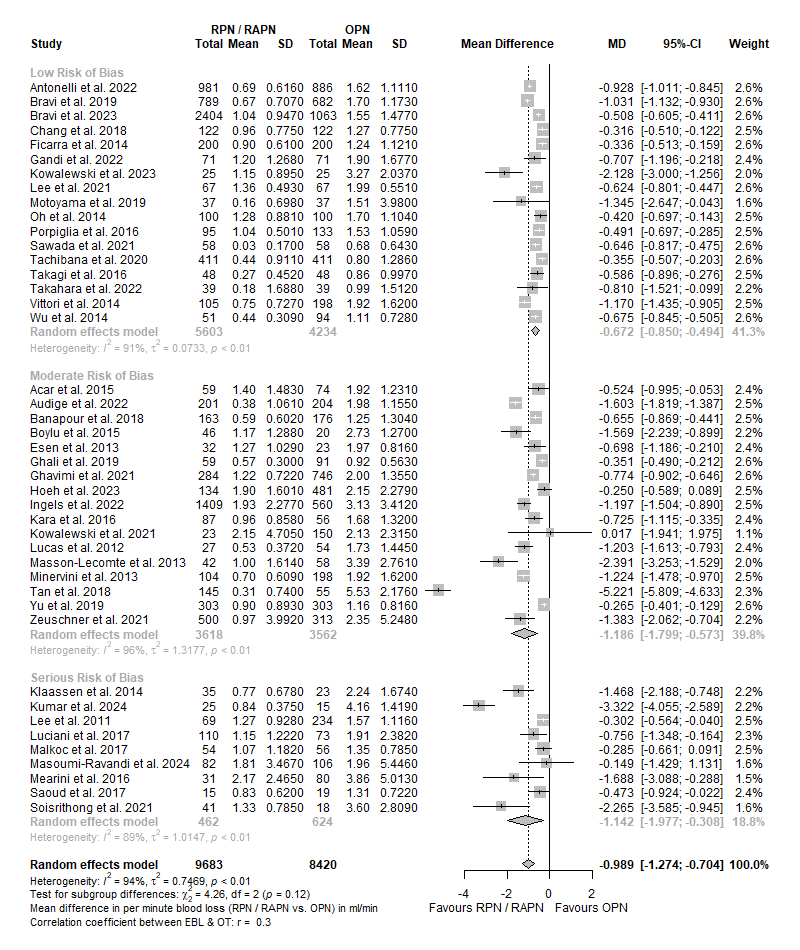

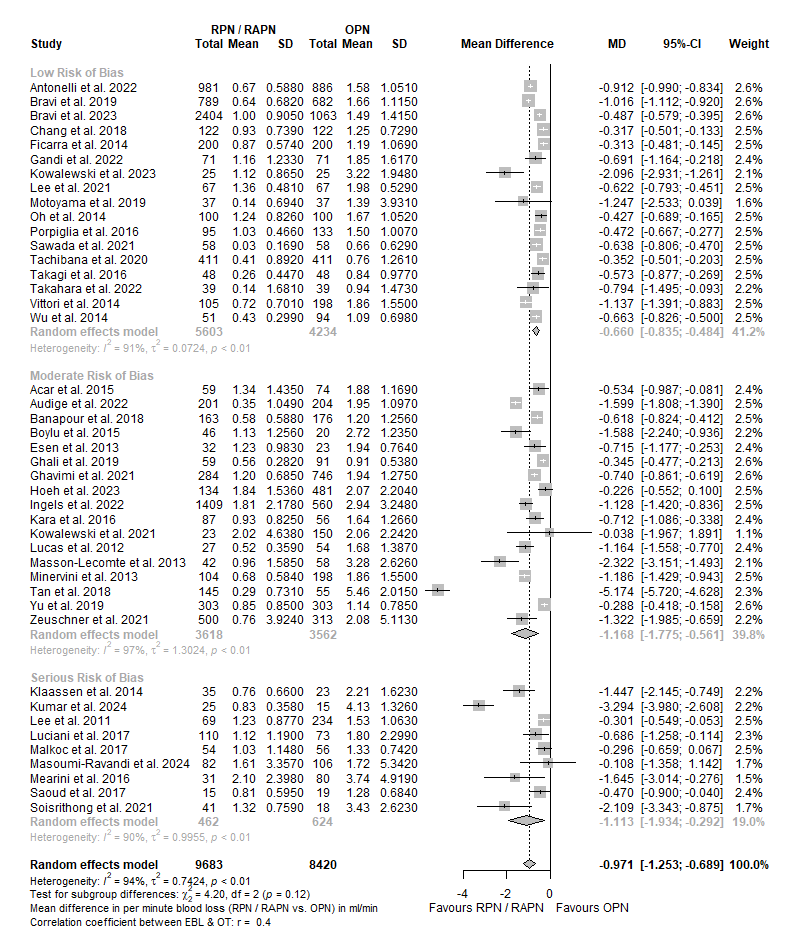

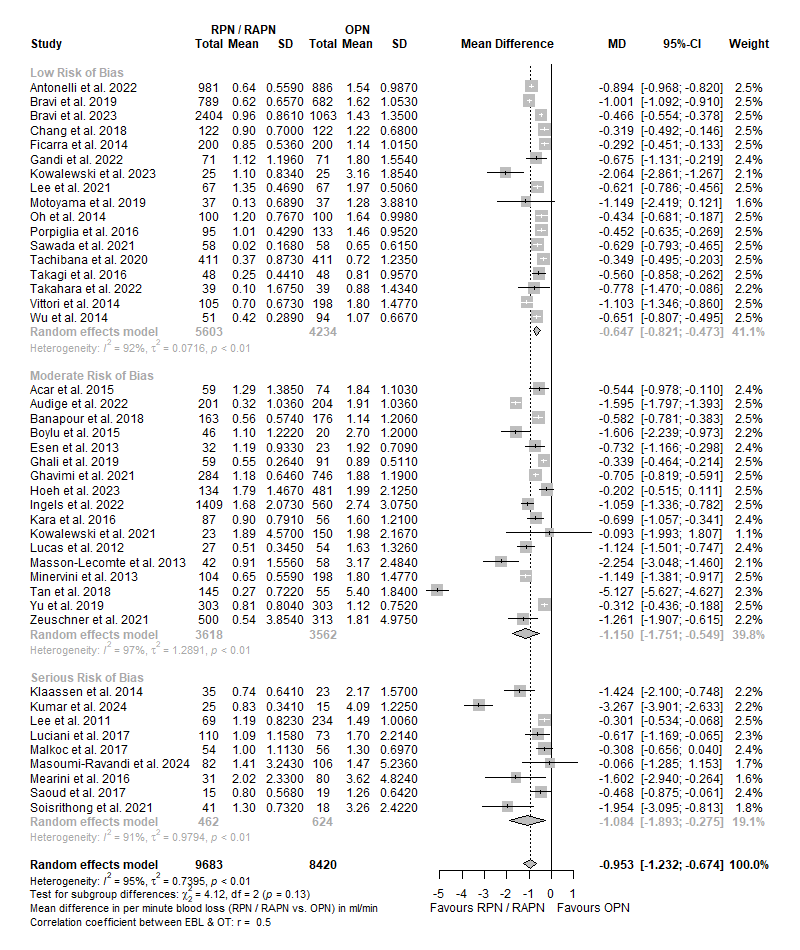
*

Supplementary SA Forest plots: Successive forest plots depicting the stepwise MD_Q_ assessment in studies with low, moderate, or serious ROB, for the gradual transition of r from -0.99 to +0.99. Abbreviations: EBL: estimated blood loss, OT: operative time, r: Pearson’s correlation coefficient between EBL and OT, ROB: risk of bias according to the ROBINS-I tool, ROBINS-I: Risk of Bias In Non-randomized Studies of Interventions.
